# miRNAs may play a major role in the control of gene expression in key pathobiological processes in Chagas disease cardiomyopathy

**DOI:** 10.1371/journal.pntd.0008889

**Published:** 2020-12-22

**Authors:** Laurie Laugier, Ludmila Rodrigues Pinto Ferreira, Frederico Moraes Ferreira, Sandrine Cabantous, Amanda Farage Frade, Joao Paulo Nunes, Rafael Almeida Ribeiro, Pauline Brochet, Priscila Camillo Teixeira, Ronaldo Honorato Barros Santos, Edimar A. Bocchi, Fernando Bacal, Darlan da Silva Cândido, Vanessa Escolano Maso, Helder I. Nakaya, Jorge Kalil, Edecio Cunha-Neto, Christophe Chevillard

**Affiliations:** 1 Aix Marseille Université, Génétique et Immunologie des Maladies Parasitaires, Unité Mixte de Recherche S906, Marseille, France; INSERM, U906, Marseille, France; 2 Laboratory of Immunology, Heart Institute (InCor), University of São Paulo, School of Medicine, São Paulo, Brazil; 3 Division of Clinical Immunology and Allergy, University of São Paulo, School of Medicine, São Paulo, Brazil; 4 Institute for Investigation in Immunology (iii), INCT, São Paulo, Brazil; 5 Aix Marseille Université, TAGC Theories and Approaches of Genomic Complexity, Inserm, INSERM, UMR_1090, Marseille, France; 6 Division of Transplantation, Heart Institute (InCor), University of São Paulo, School of Medicine, São Paulo, Brazil; 7 Department of Pathophysiology and Toxicology, School of Pharmaceutical Sciences, University of São Paulo, São Paulo, Brazil; 8 Scientific Platform Pasteur, University of São Paulo, São Paulo, Brazil; University of Georgia, UNITED STATES

## Abstract

Chronic Chagas disease cardiomyopathy (CCC), an especially aggressive inflammatory dilated cardiomyopathy caused by lifelong infection with the protozoan *Trypanosoma cruzi*, is a major cause of cardiomyopathy in Latin America. Although chronic myocarditis may play a major pathogenetic role, little is known about the molecular mechanisms responsible for its severity. The aim of this study is to study the genes and microRNAs expression in tissues and their connections in regards to the pathobiological processes. To do so, we integrated for the first time global microRNA and mRNA expression profiling from myocardial tissue of CCC patients employing pathways and network analyses. We observed an enrichment in biological processes and pathways associated with the immune response and metabolism. IFNγ, TNF and NFkB were the top upstream regulators. The intersections between differentially expressed microRNAs and differentially expressed target mRNAs showed an enrichment in biological processes such as Inflammation, inflammation, Th1/IFN-γ-inducible genes, fibrosis, hypertrophy, and mitochondrial/oxidative stress/antioxidant response. MicroRNAs also played a role in the regulation of gene expression involved in the key cardiomyopathy-related processes fibrosis, hypertrophy, myocarditis and arrhythmia. Significantly, a discrete number of differentially expressed microRNAs targeted a high number of differentially expressed mRNAs (>20) in multiple processes. Our results suggest that miRNAs orchestrate expression of multiple genes in the major pathophysiological processes in CCC heart tissue. This may have a bearing on pathogenesis, biomarkers and therapy.

## Author summary

Chronic Chagas disease cardiomyopathy (CCC), an aggressive dilated cardiomyopathy caused by *Trypanosoma cruzi*, is a major cause of cardiomyopathy in Latin America. Little is known about the molecular mechanisms responsible for its severity. Authors study the possible role of microRNAs in the regulation of gene expression in relevant pathways and pathobiological processes. Differentially expressed genes (DEGs) and differentially expressed miRNAs (DEMs) -small RNAs that can regulate gene expression—associated to severe cardiomyopathy development. The inflammatory mediator Interferon-γ was the most likely inducer of gene expression in CCC, and most genes belonged to the immune response, fibrosis, hypertrophy and mitochondrial metabolism. A discrete number of differentially expressed mRNAs targeted a high number of differentially expressed mRNAs in multiple processes. Moreover, several pathways had multiple targets regulated by microRNAs, suggesting synergic effect. Results suggest that microRNAs orchestrate expression of multiple genes in the major pathophysiological processes in CCC heart tissue.

## Introduction

Chagas disease is a major public health problem in Latin America, resulting from lifelong infection with the protozoan parasite *Trypanosoma cruzi*. Up to 30 years after acute infection, approximately 30% of the 6 million infected people eventually develop chronic Chagas cardiomyopathy (CCC), a life-threatening inflammatory dilated cardiomyopathy [1,2]. Most other *T*. *cruzi*-infected patients will remain asymptomatic for life (60%) or develop digestive disease, which causes less deaths (approx. 10%) [1]. Chagas disease is the most common cause of non-ischemic cardiomyopathy in Latin America, causing approximately 10,000 deaths/year, mainly due to heart failure and severe arrhythmia/sudden death [1]. Migration turned Chagas disease into a global health problem, with an estimated 400,000 infected persons living in nonendemic countries, mainly the United States and Europe. Current anti–*T*. *cruzi* drugs have shown to be unable to block progression toward CCC [3].

After acute infection, parasitism is partially controlled by the immune response, and low-grade parasite persistence fuels the systemic production of inflammatory cytokines like IFN-γ and TNF-α, which is more intense in CCC than ASY patients [4–6]. CCC is characterized by a monocyte and T cell-rich myocarditis [7,8] with cardiomyocyte damage and hypertrophy, and prominent fibrosis; *T*. *cruzi* parasites are very scarce. IFN-γ producing Th1 cells accumulate in the myocardium of CCC patients [4,9,10] in response to locally produced chemokine ligands CXCL9 and CCL5 [11]. Accordingly, IFN-γ was found to be the most highly expressed cytokine mRNA in CCC myocardium using a 13-cytokine panel [12]. Both heart-crossreactive [13] and *T*. *cruzi*-specific T cells [14] have been found in CCC heart tissue, and both and may play a role in the myocarditis of CCC. Together, evidence suggests that myocarditis and IFNγ signaling plays a major pathogenic role in CCC development and severity (reviewed in [2,15]), although downstream events leading to the heart disease phenotype are still obscure.

CCC has a worse prognosis than cardiomyopathies of non-inflammatory etiology, like ischemic or idiopathic dilated cardiomyopathy (DCM) [15]. Our group has shown that the myocardial gene expression profiles in CCC patients are profoundly different from those of both heart donors and DCM patients as assessed the “Cardiochip” cDNA microarray encoding ca. 11,000 expressed sequence tags (EST) cDNAs expressed in cardiovascular tissue [16]. Indeed, 15% of all genes specifically upregulated in CCC myocardium were found to be IFN-γ-inducible, indicating a strong IFN-γ transcriptional signature. This suggested that the increased aggressiveness of CCC could be related at least in part to activation of IFN-γ-dependent genes and pathways. Significantly, systemic overexpression of IFNγ in transgenic mice causes a TNFα-dependent inflammatory dilated cardiomyopathy [17,18]. Likewise, immunohistological signs of inflammation in suspected myocarditis of postviral etiology is associated with a poor prognosis [19], and sustained and long-term inflammation plays a role in worsening cardiac hypertrophy and chronic heart failure [20]. Mitochondrial dysfunction and oxidative stress have been associated with the pathogenesis of dilated cardiomyopathy and heart failure [21,22]. Indeed, our group has observed altered levels of 16S mitochondrial RNA as well as mRNA encoding mitochondrial proteins [16] and reduced levels of mitochondrial energy metabolism enzymes in that were specific to CCC [23]. Myocardial mitochondrial dysfunction and oxidative stress have been identified and explored in murine models of CCC (Reviewed in [24]). However, the determinants of expression of the majority of differentially expressed genes in CCC- as well as their roles in the key pathogenic roles of hypertrophy, fibrosis, arrhythmia and myocarditis—still remained mostly unknown.

MicroRNAs (miRNAs), short non-coding RNAs (18 to 24 nucleotides), are post-transcriptional regulators critically involved in a multitude of biological processes by modulating protein expression of up to 60% of the genes. miRNAs act by hybridizing with complementary sequences the 3'untranslated region (UTR) of mRNAs, exerting a downregulatory effect through direct degradation of the target mRNA and/or translational repression. MiRNAs also play a key role in multiple disorders, including cardiovascular disease [25]. Modulation of miRNA expression can profoundly alter disease phenotypes, and miRNA-based therapeutics has already entered clinical trials [26]. Our group recently studied the mRNA and miRNA transcriptome in the myocardium of mice acutely infected by *T*. *cruzi* [27], and we have previously shown that expression of muscle-enriched miRNAs ("myoMiRs", including miR-1 and miR-133) is downmodulated in CCC myocardium, suggesting miRNA may control expression of pathogenetically relevant genes in CCC [28]. We raised the hypothesis that mRNA expression and pathways/processes may to be heavily influenced by miRNA expression. To comprehensively address this issue, we performed an integrative genome-wide analysis of the role of miRNA in global gene expression in CCC.

## Methods

### Ethics statement

The protocol was approved by the Institutional Review Board of the University of São Paulo, School of Medicine and written informed consent was obtained from the patients. All experimental methods comply with the Helsinki declaration.

### Patients and sample collection

Human left ventricular free wall heart tissue was obtained from end-stage heart failure patients at the moment of heart transplantation. Patients with CCC presented positive *T*. *cruzi* serology and typical heart conduction abnormalities (right bundle branch block and/or left anterior division hemiblock) and had a histopathological diagnosis of myocarditis, fibrosis and hypertrophy. Left ventricular free wall samples were also obtained from hearts of organ donors, which were not used for transplantation due to size mismatch with available recipients. (n = 4) All left ventricular free wall heart tissue samples were cleared from pericardium and fat and quickly frozen in liquid nitrogen and stored at −80°C.

### RNA Extraction and RT-PCR

Myocardium samples (20–30 mg) were crushed with ceramic beads (CK14, diameter 1.4 mm) in 350 μl of RLT lysis buffer supplemented with 3.5 μl of β-mercapto-ethanol. Total RNA for mRNA expression profilling was extracted with the RNeasy Mini Kit (Qiagen, Courtaboeuf, France) adapted with Trizol. RNA quality and quantity was measured with a 2100 Bioanalyser. Total RNA (1μg), with a RIN > 7, was reverse-transcribed with the high Capacity cDNA Reverse Transcription Kit (ThermoFisher Scientific, Saint Aubin, France).

### Whole human genome expression analysis

Whole genome expression analysis was done on SurePrint G3 Human GeneExpression v1 8x60K arrays (Agilent Technologies, Les Ulis, France) following the manufacturer's protocol. Gene expression data were previously deposited in the GEO database (GSE84796 and GSE111544). Microarray analyses and signal normalization were done with GeneSpring software (11.5.1), T test with adjustment for false discovery rate with the Benjamini-Hochberg method. Genes were considered differentially expressed if adjusted P values were <0.05 and absolute fold change >2.0. In order to validate the microarray results, quantitative real-time PCR, from 20ng of cDNA, was performed with the ABI 7900HT thermocycler and TaqMan Universal PCR Master Mix (Applied Biosystems, Life Technologies). The Student's T test was used to identify differentially expressed genes between CCC and controls by TaqMan RT-qPCR. Gene expression data used in this manuscript are the same ones that used in one of previous work on methylation analysis.

### Principal component analysis, network and pathways analysis

Principal component analysis (PCA) analysis was performed using all differentially expressed genes and the variance expression of the number of standard deviations from mean overall samples. Canonical pathways analysis, networks analysis and Upstream regulator analysis, and classification of differentially genes belonging to pathways and biological processes were performed with Ingenuity Pathway Analysis (IPA, Qiagen Redwood City, CA, USA). We also classified genes in additional relevant pathobiological processes and pathways such as inflammation, IFNγ-modulated genes/Th1 response, extracellular matrix, fibrosis, hypertrophy, contractility of heart, hypertrophy, arrhythmia, oxidative stress/antioxidant response, mitochondria, and mitochondria-related genes using IPA Knowledge Base (IKB) gene lists, which were in some cases merged with other published gene lists. The IFNγ-dependent/Th1 response gene list was merged with published IFNγ-induced/repressed gene lists [29], and the oxidative stress gene list was merged with Nrf2-modulated genes [30]. The NF-kB-modulated gene list was obtained from Yang et al. [31]. The mitochondrial gene list was a combination of all genes contained in the Mitochondrion Gene Ontology term and Mitocarta 2.0 [32]. Deconvolution of immune and cardiac cell types was performed by comparing the differentially expressed genes with the ARCHS4 tissue database in the EnrichR tool [33] (Adjusted P-value < 0.001). The network of cell types representing the genes shared by different tissues was constructed using the Cytoscape tool [34]. Prediction of differentially expressed miRNA-mRNA target relationships was performed with the IPA Knowledge Base. We selected high predicted or experimentally validated miRNA-target relationships.

### Quantitative miRNA expression profiling

RNA and cDNA were obtained from human myocardial samples as previously described [35]. MiRNA profiling experiments were done for 754 miRNAs using pre-printed TLDA microfluidic cards (Human MicroRNA Card Set v3.0), according to the manufacturer's protocols and as described [35]. Raw TLDA data files were pre-processed with threshold and baseline corrections for each sample (automatic baseline and threshold set to 0.3) with assessment of each amplification plot on SDS 2.3 software (ThermoFisher). Cycle threshold (Ct) values from quantitative real time PCR data were imported, normalized and tested for statistical significance with the HTqPCR Bioconductor package [36]. Samples data quality and outliers removal were assessed with the arrayQualityMetrics Bioconductor package [37]. Distribution of the samples Ct values were normalized against the endogenous control RNU48-001006. Differentially expressed miRNAs (DEMs) were determined using a wrapper function from the Bioconductor package LIMMA [38]. Variance filtering was applied and, for each miRNA, up to two failed reads per group were accepted for partial coefficients calculation. Resulting p-values were submitted to false discovery rate adjustment according to the Benjamini-Hochberg method and the statistical significance threshold was defined as p-value ≤ 0.05, with an absolute fold change cutoff ≥ 1.5.

### miRNA target gene interaction analysis

miRNA-target gene interaction analysis was done with performed with Ingenuity Pathway Analysis (IPA, Qiagen Redwood City, CA, USA). Analysis was done in three steps. First of all, for each DEM, we extracted from IPA database all the reported target genes (high predicted or experimentally observed). Then, among all these targets we kept only the ones that were differentially expressed (DEGs) in our gene expression analysis. Finally, we kept only the targets presenting an inverse pairing expression. In the analysis we included DEMs with an absolute fold change over 1.5 and DEGs with an absolute fold change over 2.0.

## Results

Information on the subjects studied in this paper is available in **Table 1**

**Table 1:** Characteristics of the human left ventricular free wall heart tissue samples used in this study.

Project Number	Form	EF	Age	Sex	Transcriptome Analysis	qRT-PCR validation	MiRnome Analysis
EBS	CCC	0.12	32	M	x	x	x
NSR	CCC	0.15	49	F	x	x	
MGS	CCC	0.20	61	F		x	
BHAN	CCC	0.20	15	M		x	
SCS	CCC	0.17	59	M	x	x	x
ECA	CCC	0.19	32	F		x	
VTL	CCC	0.19	41	M		x	
APA	CCC	0.20	60	F	x	x	
MCRS	CCC	0.20	45	F	x	x	x
MERS	CCC	0.20	39	F		x	
MSS	CCC	0.20	46	F		x	
GMS	CCC	0.20	58	M		x	
ISM	CCC	0.20	39	M		x	
OMG	CCC	0.21	49	M		x	
MAP	CCC	0.23	50	F	x	x	
EPG	CCC	0.23	41	M		x	
JRJ	CCC	0.23	51	M		x	
LRJ	CCC	0.25	66	F		x	
HBO	CCC	0.25	36	M	x	x	x
PMG	CCC	0.29	57	M	x	x	x
ABG	CCC	0.30	64	F		x	
ZMC	CCC	0.36	54	F	x	x	x
JAB	CCC	0.55	41	M		x	
AAF2	CCC	0.64	60	M	x	x	x
JMS	CCC	0.66	50	M		x	
LAL	CCC	0.29	39	M		x	x
EMBT	control		25	M	x	x	
LO	control		46	M	x	x	
ESS	control		22	M	x	x	x
ZFS	control		x	M	x	x	x
FJR	control		28	M	x	x	x
MBFM	control		17	M	x	x	x
3557	control				x	x	

We found 1535 genes to be differentially expressed (DEG) between CCC and control myocardium, of which 1105 (72%) are upregulated, while 430 (28%) genes are downregulated in CCC (**S1 Table)**. To validate the microarray results, we performed qPCR of 44 differentially expressed genes on the same samples (independent extractions) used for the microarray study, plus 16 new CCC samples. The confirmation rate was 86%, and only for 6 genes (ABRA, CDC42, ESRRA, GPD1, NFATC2, TGFBR2), the expression patterns were not confirmed. The gene specific qRT-PCR results, including fold change and p values, were previously described ([39], S2 Table).

**Fig 1:**
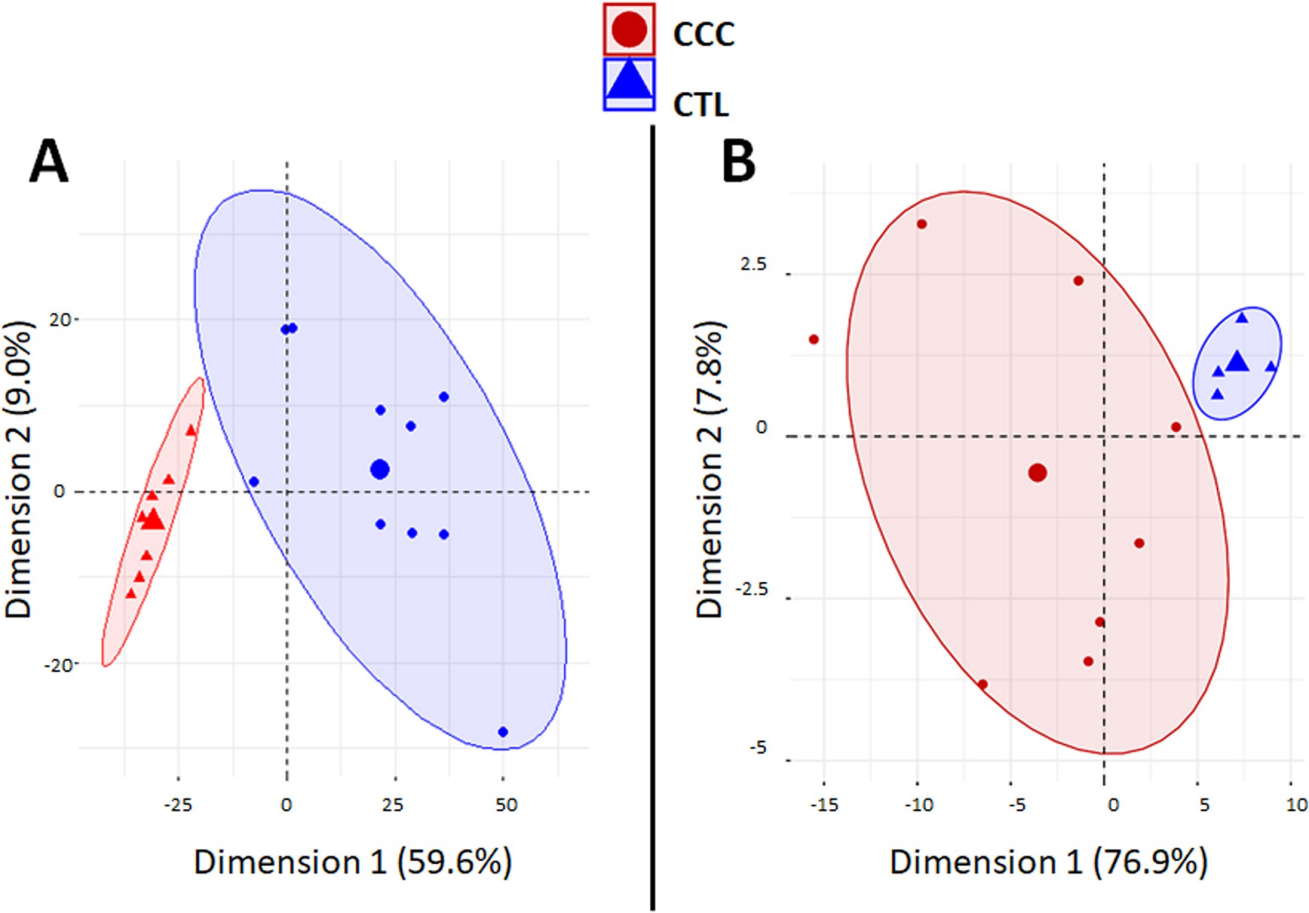
Principal component analysis (PCA) plots. Principal component analysis (PCA) plot of samples was performed based (A) on 1535 differentially expressed genes (DEGs) between CCC and controls. The two main principal components have the largest possible variance (68.8%). The 1535 DEGs had an equal contribution to the first component (ranging from 6.0E-3% to 0.1%). For the second component, 25 DEGs had a contribution over 0.25% (GAB3, WBSCR27, LOC100130930, C1orf35, ISLR2, SLC25A34, NOTCH2, TSPAN32, ATP1A1OS, C11orf65, ZNF214, APCDD1, C1QTNF6, RANBP17, MNS1, APBB3, ANGPTL1, BEND6, LTB, MMP9, ITGB2, PIK3R1, NOTCH2NL, TRMT5 and XLOC_005730); (B) or on 80 differentially expressed miRNAs (DEMs) between CCC and controls. The two main components explain 84.7% of the variance. For the first component, the 80 DEMS had an equal contribution (ranging from 0.24% to1.74%). For the second component, even if all the DEMs contribute, six of the DEMs have a main contribution (hsa-miR-155: 12.8%; hsa-miR-146a: 9.0%; hsa-miR-302d: 8.8%; hsa-miR-378: 8.1%; hsa-miR-486: 7.0%; and hsa-miR-221: 5.2%).

**Fig 2:**
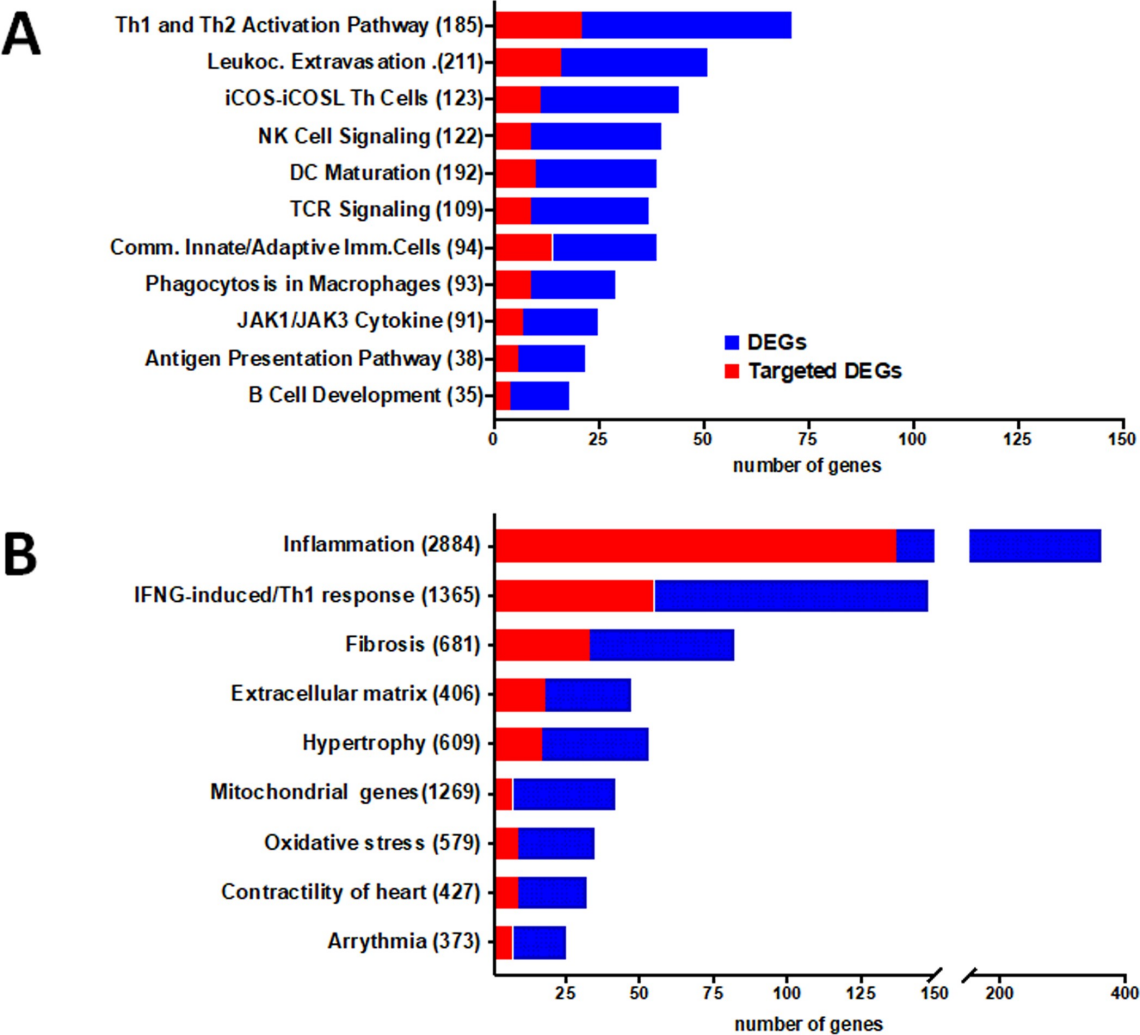
DEGs and DEM-targeted DEGs present in relevant canonical pathways and pathophysiological processes. The stacked bar chart displays the number of DEG (blue) and DEM-targeted DEGs (red) present in each pathway. **A.** Ingenuity Pathway Analysis (IPA) canonical pathways representative of the most significantly enriched in the heart of CCC patients. **B.** DEG and DEM-targeted DEGs in specific biological processes relevant for the CCC pathogenesis. The numerical value in the parentheses in front of each pathway name represents the total number of genes in that pathway/process.

A PCA analysis based on the all differentially expressed genes (DEGs) showed clustering of samples from each group in distinct areas of the plot (**Fig 1A**), confirming that CCC myocardial gene expression patterns were substantially different from controls. A heatmap based on DEGs confirmed the good clustering (**S1A Fig**). Likewise, differential clustering of CCC and control miRNAs also confirmed miRNA expression patterns are distinct in CCC and controls (**Fig 1B**). IPA canonical pathways analysis showed that the most enriched pathways are mainly immune-related, such as Th1 and Th2 T cells, dendritic cells/antigen presentation, leukocyte extravasation, NK and B cells; this is consistent with the high number of upregulated genes from the incoming inflammatory cells present in CCC but not in control heart tissue (**Fig 2A). S2 Table** depicts the DEGs belonging to all significant IPA canonical pathways. **Fig 2B** shows the number of genes in each pathobiological process relevant for the disease such as inflammation, IFNγ-modulated genes/Th1 response, extracellular matrix, fibrosis, contractility of heart, hypertrophy, arrhythmia, oxidative stress/antioxidant response, and mitochondria-related genes. The number of DEGs for each process is described on **Fig 2B**. **S3 Table** contains the complete list of DEGs belonging to each pathobiological process. As expected, inflammation and IFNγ-dependent/Th1 response processes show the highest number of DEGs (361 and 148, respectively), followed by fibrosis (82) and hypertrophy (53). Of interest, we found a significant number of DEGs belonging to mitochondria and oxidative stress functions/processes (42 and 35, respectively). Some DEGs are shared by several biological functions/processes (**S4 and S5 Tables)**. IFNγ-dependent DEGs were found in all other 8 processes, ranging from 9% to 40% of genes in the other processes; those represented 104 inflammation, 33 fibrosis, 18 hypertrophy, 8 contractility and 7 mitochondrial genes.

**Fig 3:**
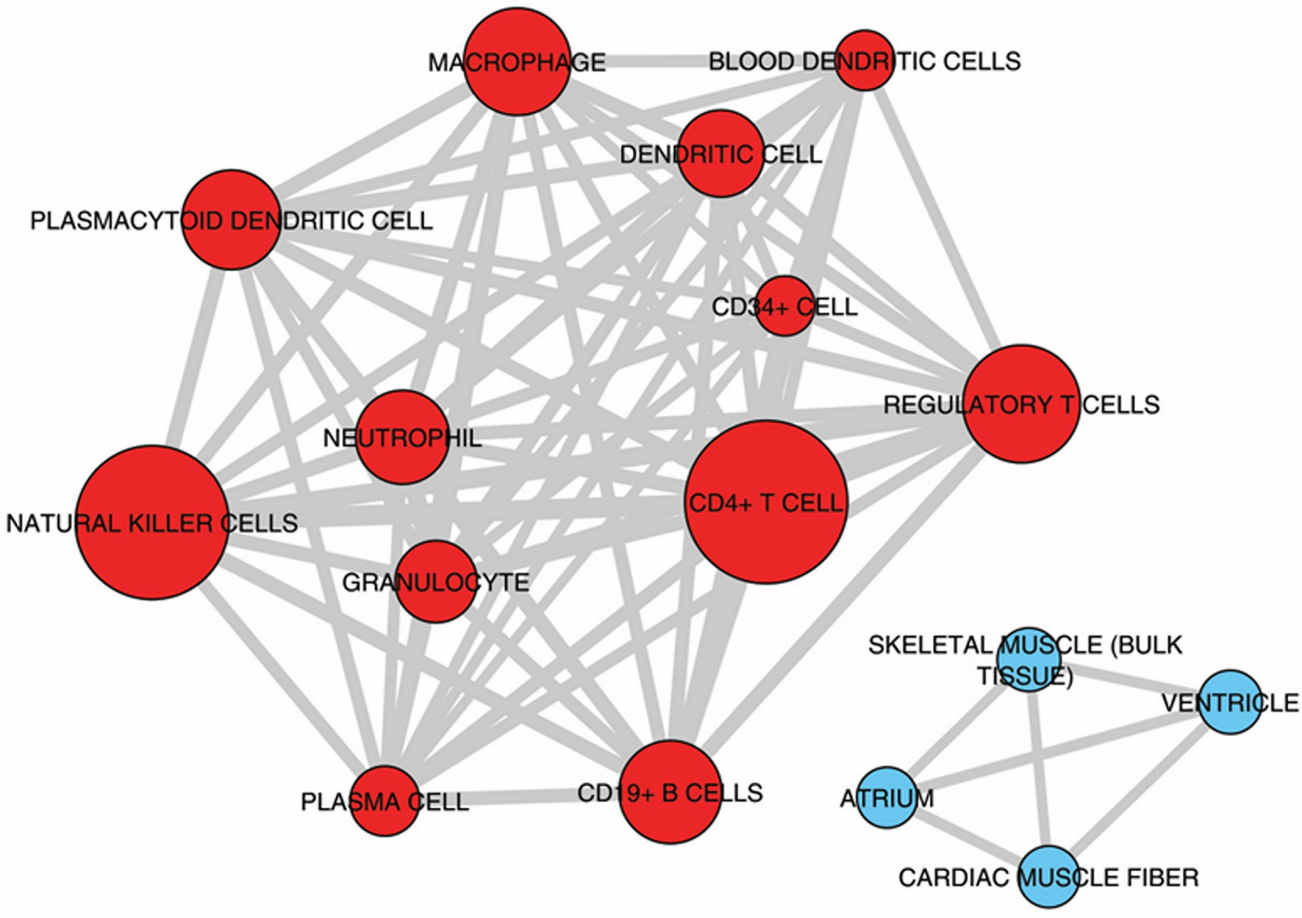
Enrichment analysis of cell subset and tissue signatures. Signatures of different tissues and cell types from ARCHS4 tissue database enriched with up-regulated (red nodes) or down-regulated (blue nodes) genes compared to controls (Adjusted P-value < 0.001). The width of edges (connecting lines) is proportional to the number of genes shared by two signatures. The size of nodes is proportional to the -log10 Adjusted P-value.

Among these processes inflammation may be specific to CCC as shown in previous gene expression studies [11,12]. For the other processes, they have been also described in dilated cardiomyopathies of other etiologies. Upstream regulator analysis performed by IPA examines how many targets of each given transcriptional regulator are present in the DEGs—as well as the direction of change–based on the literature and IPA knowledge base; putative regulators are ranked according to overlap with expected targets and directionality (z-score). It indicated that IFNγ is the top upstream regulator, followed by other cytokines like TNFα, IL-18 and EBI3/IL27Rβ chain, the chemokines CCL5 and CXCL10, the transcription factors NF-kB and Ap1, and the PI3K enzyme (**Table 2**). **S6 Table** shows the 27 cytokines and chemokines upregulated in CCC heart tissue. Significantly, the 7 most upregulated among them were chemokines, including chemokine ligands of CCR5 (CCL5, CCL4) and CXCR3 CXCL9 and CXCL10). Multiple cytokines and chemokines that were top upstream regulators like IFNγ, CCL5, CXCL10, IL-18, IL-7, EBI3/IL-27b and IL-4 were found to be upregulated to different degrees in CCC myocardium. Deconvolution of immune cell type profiles in CCC myocardium revealed an enrichment of gene expression signatures of CD4+ T cells, NK cells, B cells/plasma cells, dendritic cells, plasmacytoid dendritic cells, regulatory T cells and granulocytes (red; **Fig 3**). This indicates that these cell types infiltrate the myocardium of CCC patients. Conversely, genes down-regulated in CCC myocardium when compared to controls were enriched with signatures of cardiac muscle cells (blue; **Fig 3**). This result is most likely a consequence of reduced representation of cardiac mRNAs in CCC myocardium that was replaced by inflammatory cells.

**Table 2:** Upstream regulator analysis in CCC myocardium.

Upstream Regulator	Molecule	Activation z-score	p-value of overlap
IFNG	cytokine	7.891	2.38E-24
TNF	cytokine	6.494	2.19E-12
IL18	cytokine	4.583	8.95E-14
NFkB (complex)	complex	4.465	5.54E-06
CD40LG	cytokine	4.287	4.10E-15
TCR	complex	3.832	1.80E-26
BCR (complex)	complex	3.771	1.27E-15
IL7	cytokine	3.466	6.32E-18
TET2	enzyme	3.357	4.04E-03
IL4	cytokine	3.082	7.43E-23
EBI3 (IL27Rβ chain IL27RB)	cytokine	2.785	2.13E-05
Fcer1	complex	2.72	3.35E-05
CXCL10	cytokine	2.581	3.15E-04
Ap1	complex	2.454	5.70E-03
CCL5	cytokine	2.432	5.92E-03
U1 snRNP	complex	2.384	1.24E-06
TNFSF14 (LIGHT)	cytokine	2.224	9.77E-03
PI3K (complex)	complex	2.007	3.62E-04
Collagen type I	complex	2.000	3.96E-01

Regarding microRNA analysis, it was performed on 8 CCC and 4 control myocardial samples contained in the mRNA transcriptome experiment (10 CCC and 7 control myocardial samples). 754 human miRNAs were screened on the heart samples and among them, 210 miRNAs were detected in every sample; these were quantified in each tissue sample. **S7 Table** shows the expression levels and statistical significance of the 210 expressed miRNAs. Based on their expression values, we have found that 80 out of 210 miRNAs were differentially expressed (DEMs) (absolute FC ≥1.5, p<0.05 without correction). A PCA analysis based on the all differentially expressed miRNAs (**Fig 1B**) and a heatmap (**S1B Fig**) confirmed that miRNA patterns were substantially different from controls. After correction for multiple testing, only miR-146a-000468 (p = 6,9E-03) and miR-155-002623 (p = 6,9E-03) remain significantly altered. However, the list of the 80 miRNAs obtained without correction for multiple testing seems to be relevant as it contains miR-1 (p = 5,0E-03), miR-133a (p = 1,4E-02) and miR-133b (p = 1.3E-2) that we previously observed as under-expressed in CCC samples as compared to controls [28]. MiR-208a, which was also previously described to be under expressed in CCC samples in the same study [28], is borderline in the present study (p = 5,5E-02).

As the controls were younger than CCC we performed some PCA analyses restricted on CCC including DEG information (**S2A Fig**) or DEM information (**S2B Fig**) then we overlaid the age of the patients. No obvious correlation was detected. We performed some spearman correlation tests 9 DEGs were age correlated and none of the DEM were age correlated (**S8 Table**). So, the age may not act as a confounding factor. Similarlly, we did a PCA analysis on cases and controls taking into account DEG information or DEM information and the sex of the patients. No specific clustering was detected (**S3A and S3B Fig**). For each DEG and DEM we made Student’s t tests between the male and female patients and no association were detected.

In order to identify putative miRNA-target gene interactions among DEMs and DEGs, we performed inverse expression pairing of DEMs (80) and DEGs (1535). A total of 571 miRNA-mRNA interactions involving 67 DEMs and 396 DEGs were found by IPA**. S9 Table** depicts all observed DEM-DEG interactions. **Fig 2A** shows the number of DEGs in the most important canonical pathways and the fraction that is targeted by DEMs. The proportion of DEGs targeted by DEMs in each depicted canonical pathway varies from 24% to 62%. A similar analysis done on DEM-DEG interactions in the 9 key pathobiological processes, which also indicated that a substantial proportion of DEGs (16.7–40.8%) are targeted by DEMs. Apart from the inflammation (38%) and IFNγ-induced genes (37.2%), pathobiological processes with the highest number of DEM-targeted DEGs are fibrosis (40.2%), extracellular matrix (38.2%) and hypertrophy (32.1%) processes (**Fig 2B**). **S10 Table** shows all DEM-DEG interactions classified according to biological process. In order to validate the specificity of the DEM-DEG targeting, we simulated the targeting of the DEGs with 80 miRNAs that were not differentially expressed in CCC myocardium (non-DEMs) as compared to 80 DEMs using the IPA miRNA target filter function, again focusing only high predicted and experimentally observed targets. Comparing the number of target DEGs in the top 6 IPA canonical pathways that were shared by DEMs and non-DEMs, we found a 50% higher number of DEG targets from DEM than DEG targets of the simulated non-DEMs (p<0.00001, chi-square). This suggests that the pairing of DEG targets with DEM was not random.

**Fig 4:**
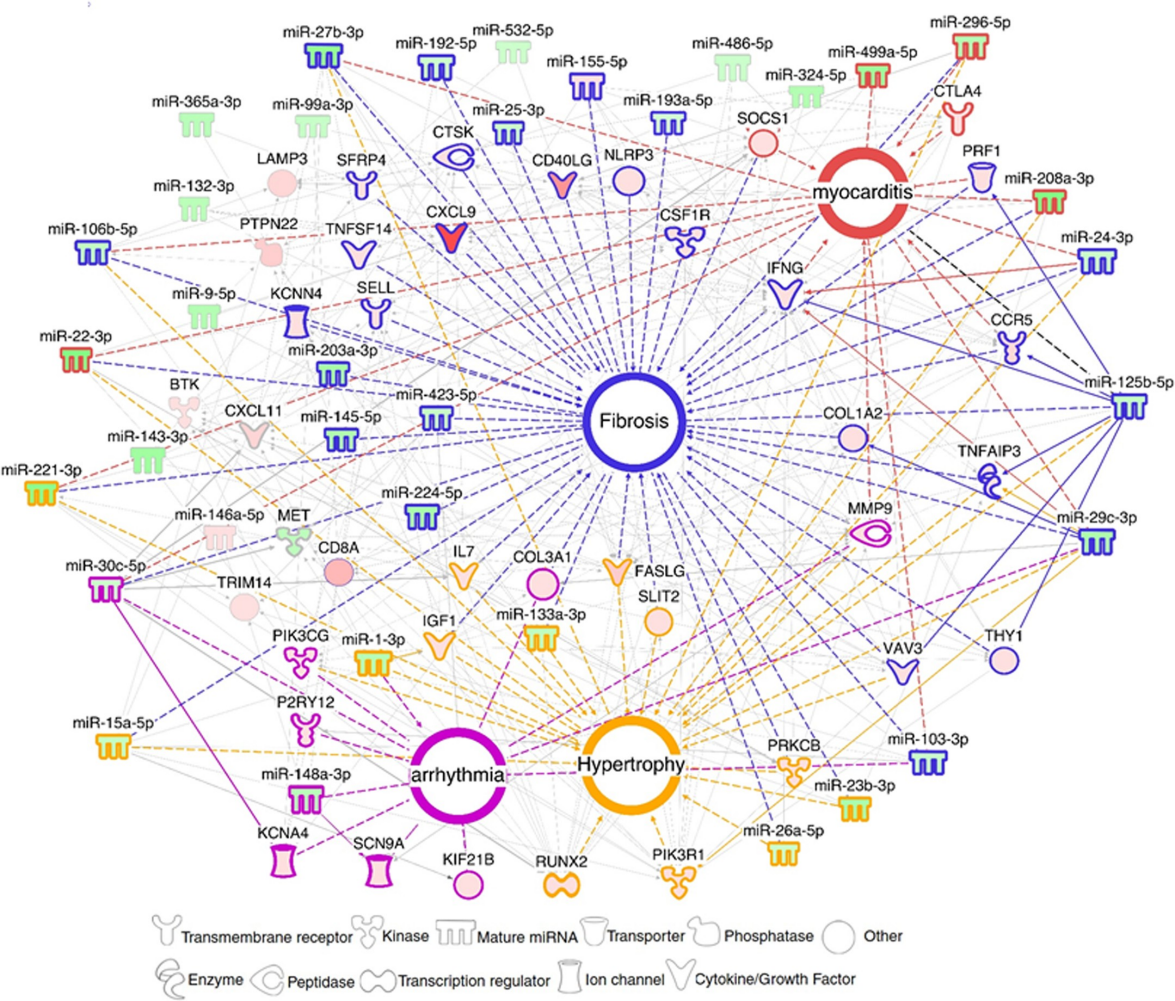
DEM-DEG network related to the main Chagas disease pathobiological features. Networks with DEMs and DEGs related to myocarditis, fibrosis, arrhythmia and hypertrophy were built using IPA software. Each built network contains molecules represented as nodes, and the biological relationship between two nodes is represented as an edge (line). All edges (connecting lines) are interactions supported by at least one reference from the literature or from canonical information stored in the IPA Ingenuity Knowledge Base (IKB). Full lines are direct interactions, dotted lines are indirect interactions. Upregulated microRNAs and mRNAs are colored in hues of red, and downregulated molecules are colored in hues of green according to the intensity of expression. Each node shape represents one type of molecule.

We found that 5 miRNAs (hsa-miR-125b-5p, hsa-miR-15a-5p, hsa-miR-296-5p, hsa-miR-29c-3p and hsa-miR-103a-3p) each regulate more than twenty DEGs; moreover, each of them affects at least 6 of the 9 biological functions and processes analyzed (**S11 Table**). Moreover, several of these “master” miRNAs targeted multiple genes belonging to a given process at the same time, suggesting a synergistic action. A network built with DEM-DEG targets around the important pathobiological processes, myocarditis, fibrosis, hypertrophy and arrhythmia disclosed a strong focus on fibrosis, and several miRNAs and targets participated in various processes (summarized on **Fig 4)**. We validated the expression of 38 genes belonging to the four pathobiological processes with real time RTqPCR in a larger set of CCC samples.

## Discussion

To assess the role of miRNAs in regulating gene expression in CCC myocardium, we performed an integrative genome-wide analysis of miRNAs and mRNA expression in CCC myocardium samples and performed network and pathways analysis. We identified 1535 differentially expressed genes (DEGs) and 80 differentially expressed miRNAs (DEMs). We found that both miRNAs and mRNA expression profiles discriminated CCC from control samples. Pathways analysis disclosed an enrichment in inflammation, Th1/IFN-γ-inducible genes, genes belonging to fibrosis, hypertrophy, and mitochondrial/oxidative stress/antioxidant response. Our results corroborated that IFN-γ is the key cytokine modulating transcriptional changes in CCC myocardium and affecting all other studied pathobiological processes, and cell type deconvolution indicated the presence of novel immune cell types that had not yet been disclosed by immunohistochemistry. Our data also suggest that a significant number of differentially expressed microRNAs target differentially expressed genes; moreover, a few microRNAs may potentially regulate simultaneously multiple genes in key pathways and pathogenetically relevant processes. Our paper is the first to indicate that miRNAs may play a role in promoting major transcriptome changes in human inflammatory cardiomyopathy.

Our results pointed out IFN-γ is the top gene expression regulator with ca. 10% of DEGs being modulatable by it, in all pathobiological processes. Indeed, several studies have shown a negative impact of IFN-γ on the myocardium, leading to reduced contractility, release of chemokines and increased production of atrial natriuretic factor [40–42]. IFN-γ-induced cardiac fibrosis with increased fibroblast proliferation, production of hyaluronan and metalloproteinases 2 and 9 has also been demonstrated [43–46]. The role of IFN-γ, TNF-α and NF-kB as top upregulators are also in line with data in genetically modified murine models. Mice transgenic to IFN-γ developed a TNF-α-dependent inflammatory dilated cardiomyopathy with fibrosis and heart failure [18], and a very similar phenotype was developed by mice constitutively expressing active IKK2 [47]. Mechanistically, IFN-γ induces TNF-α and potentiates TNF-α-mediated NF-kB signaling and upregulation of NOS2 [48,49], leading to cardiomyocyte contractile dysfunction and apoptosis [Sun, 1998 #110]. This is mediated at least in part by NADPH-and NOS2-dependent production of reactive oxygen and nitrogen species (ROS and RNS, respectively), with oxidative and nitrosative stress [40,50]. IFN-γ -induced RNS leads to inhibition of mitochondrial oxidative metabolism [51] and ATP depletion in cardiomyocytes [52] with ensuing mitochondrial dysfunction. Of interest, 169 DEGs, or ca 10% of DEGS are potentially modulated by NF-kB in CCC myocardium. Our data point towards IFN-γ and NF-kB-mediated signaling as a major player in Chagas cardiomyopathy; we believe they may have a central role in orchestrating the molecular processes that contribute to heart failure. It is noteworthy that IFN-γ may also act through modulation of miRNA expression in CCC myocardium. IFN-γ down-regulates 5 miRNAs (27b, 92a, 99a, 99b, 101) [53,54] which found to be downregulated DEMs in CCC myocardium.

Previous immunohistochemistry studies on CCC heart tissue haves identified a few cell types in the inflammatory infiltrate. Most cells were CD68+ macropages, CD4 and CD8+ T cells, with fewer B cells, NK cells, and TGF-beta-expressing cells [7,8,10]. In addition to these cell types, the deconvolution of immune cell type transcriptional profiles shown here provided evidence for the presence of plasmacytoid dentritic cells, regulatory cells, plasma cells and granulocytes. Many of the genes in the NK signature are shared with CD8+ cytotoxic T cells which are abundant in CCC heart tissue [8]; this has previously been observed in transcriptome profiling of peripheral blood from CCC patients [27], and we believe our finding in myocardium represents an NK/CD8+ T cell cytotoxicity signature. Regarding regulatory T cells, we had previously found a low expression of CTLA-4 mRNA in CCC heart tissue, suggesting a small component of CTLA-4+ T regs [12].

A significant proportion of the DEGs in the pathways and processes we studied (15–62%) were targeted by differentially expressed miRNA (DEM). We found that some DEMs had unusually high numbers of target DEGs. Five downmodulated DEMs (hsa-miR-15a-5p, hsa-miR-29c-3p, hsa-miR-103a-3p, hsa-miR-125b-5p and hsa-miR-296-5p) each targeted at least 20 DEGs involved in 6 or more studied pathobiological processes. Their functions in the context of heart disease and inflammation are described below. Downmodulated miR-15a may targets 23 upregulated DEGs involved in 5 out of the 8 processes described above. Previous data indicate that the miR-15 family is involved in TGFβ-pathway inhibition [55] while miR-15 antagonists induce fibrosis and hypertrophy [56], which are important features of Chagas disease. Our study also revealed down regulation of hsa-miR-29c-3p, associated with 21 upregulated target DEGs involved in all processes described above. The miR-29 family members target several genes related to extracellular matrix and fibrosis [57–59], and miR29b was shown to be inhibited by the myocardial infarction associated transcript (MIAT) [29], a long non-coding RNA overexpressed in CCC patients’ myocardium [60].

Downmodulated miR-103 targets 20 upregulated DEGs, involved in 6 out of the 8 processes previously described. MiR-103 is overexpressed in the myocardium of heart failure patients [61], and attenuates cardiomyocyte hypertrophy by a mechanism that partially relies on reducing cardiac autophagy [62]. Down modulated miR-125b targets 23 upregulated DEGs in 6 out of the 8 processes described above. The miR-125 family members negatively regulate the expression of TNF-α, reducing ischemia/reperfusion damage [63] also reducing chemokine RANTES (CCL5), which is highly expressed in our CCC samples. Lastly, down modulated miR-296 targets 22 upregulated DEGs. MiR-296 is linked to fibrosis, and is similarly downregulated in hypertensive patients [64].

Only two miRNAs (miR-155-5p and miR-146a-5p) were found to be upregulated in CCC patients. miR-155 has been shown to increase the global Nrf2 transcriptional response by targeting translation of the transcriptional regulator BACH1 [65], indicating that this miRNA may have an impact on oxidative stress in CCC hearts and its upregulation may have occurred as a compensatory mechanism to intense oxidative stress. This hypothesis will require biological validations. The Nrf2 pathway and HMOX1 have been reported to play a role in "tissue tolerance"—the ability of resist pathogen, inflammation, or oxidative stress-mediated damage during infection or inflammation [66,67] and we have found that HMOX2, a homologous Nrf2-induced gene involved in the antioxidant response, is down regulated in CCC myocardium. miR-146a is expressed in multiple cardiac cell types [68] and was found to be increased upon induction of cardiotoxicity and to inhibit proteins involved in heart regeneration (ErbB-2 and -4) [69,70], suggesting that overexpression of this miRNA may have a negative impact on cardiac function. On the other hand, miR-146a was shown to be induced by NF-κB and to create an anti-inflammatory feedback loop by inhibiting NF-κB-induced proinflammatory cytokine production and inflammatory cell migration into the myocardium [71,72] promoting Treg suppressor function [73], suggesting that this miRNA also presents cardioprotective effects. However, we have shown here and elsewhere [11,12] that Th1 inflammatory cytokines and chemokines are highly expressed in CCC heart tissue and very few Treg are detectable in CCC myocardium [10–12]. Like miR-155, it is possible that it is upregulated as a failed attempt to modulate inflammation.

Our study was performed in whole heart tissue, containing several cell types, including cardiomyocytes, fibroblasts, endothelial and infiltrating inflammatory cells. We must thus keep in mind that results reflect the composite of mRNA and microRNA content of each cell type with its respective contribution. Most of the RNA will come from cardiomyocytes, but inflammatory cell RNA will readily show up, since control tissue is free from inflammatory infiltrates, showing at most passenger leukocytes that are much less numerous. At any event, our results suggest that, by targeting multiple genes in relevant pathogenic disease pathways and processes, miRNAs can exert a combined regulatory effect that may be stronger than the effect of a single DEM-DEG interaction. In addition, we found a small number of key "high-ranking" differentially expressed miRNAs—those with the highest number of targets, overlapping with those with multiple targets involved in several pathological processes. Our data identified specific molecular features in key pathogenic processes. Further investigation and validation of the more important miRNA-mRNA interactions involved in fibrosis, oxidative stress, and mitochondrial processes may reveal important insights into the pathogenesis of CCC and may translate in the identification of novel therapeutic targets. Our findings may have a bearing on myocarditis and inflammatory cardiomyopathy of distinct etiologies as well as to IFN-γ mediated age-related myocardial inflammation and functional decline [74] as recently described.

## Supporting information

S1 Table List of differentially expressed genes on human heart biopsies from end stage patients or organ donors. (PDF)

S2 Table Description of the canonical pathways containing the DEGs in CCC myocardium. (PDF)

S3 Table DEGs associated to biological functions and processes in CCC myocardium. (PDF)

S4 Table Number of DEGs shared between several pathobiological functions or processes in CCC myocardium. (PDF)

S5 Table DEGs shared between several pathobiological functions or processes in CCC myocardium. (PDF)

S6 Table Cytokines and chemokines differentially expressed in CCC heart tissue. (PDF)

S7 Table DEMs in CCC myocardium. (PDF)

S8 Table Correlation between DEG/DEM and the age of the patients. (PDF)

S9 Table DEM-DEG interactions in CCC myocardium. (PDF)

S10 Table DEM-DEG interaction in each pathobiological function or process in CCC myocardium. (PDF)

S11 Table Five DEMs that control a large number of DEGS and processes in CCC myocardium. (PDF)

S1 Fig Unsupervised hierarchical clustering done on patients with severe chronic Chagas disease cardiomyopathy (CCC) and controls. **A.** Unsupervised hierarchical clustering based on the 1535 DEGs. **B.** Unsupervised hierarchical clustering based on the 80 DEMs.
(TIF)

S2 Fig Principal component analysis (PCA) plots taking into account the age of the cases. Principal component analysis (PCA) plot of samples was performed based **A.** on 1535 differentially expressed genes (DEGs) between CCC and controls. **B.** on 80 differentially expressed miRNAs (DEMs) between CCC and controls. Each plot was generated only on cases and the age of of the patients was overlaid.
(TIF)

S3 Fig Principal component analysis (PCA) plots taking into account the sex of the cases and controls. Principal component analysis (PCA) plot of samples was performed based **A.** on 1535 differentially expressed genes (DEGs) between CCC and controls. **B.** on 80 differentially expressed miRNAs (DEMs) between CCC and controls. On each plot cases and controls are indicated according to their sex.
(TIF)
