# Supplementary material for: miRNAs may play a major role in the control of gene expression in key pathobiological processes in Chagas disease cardiomyopathy
Source: PLoS Negl Trop Dis. 2020 Dec 22;14(12):e0008889. doi: 10.1371/journal.pntd.0008889 (PMC7787679; doi:10.1371/journal.pntd.0008889)
Supplement: S1 Table — (PDF) [file pntd.0008889.s001.pdf]

S1 table. List of differentially expressed genes on human heart biopsies from end stage patients or organ donors.

Gene Symbol	Probe Name	p (Corr)	Fold Change	Gene Name	Description
IGJ	A_23_P167168	1.91E-06	72.66	immunoglobulin J polypeptide, linker protein for immunoglobulin alpha and mu polypeptides	Homo sapiens immunoglobulin J polypeptide, linker protein for immunoglobulin alpha and mu polypeptides (IGJ), mRNA [NM_144646]
IGLL1	A_24_P239076	1.95E-06	132.21	immunoglobulin lambda-like polypeptide 1	Homo sapiens immunoglobulin lambda-like polypeptide 1 (IGLL1), transcript variant 1, mRNA [NM_020070]
LINC00426	A_33_P3333960	1.95E-06	61.03	long intergenic non-protein coding RNA 426	Homo sapiens long intergenic non-protein coding RNA 426 (LINC00426), non-coding RNA [NR_024464]
TBC1D10C	A_33_P3328559	1.95E-06	30.33	TBC1 domain family, member 10C	Homo sapiens TBC1 domain family, member 10C (TBC1D10C), mRNA [NM_198517]
FCRL6	A_33_P3285734	1.95E-06	24.44	Fc receptor-like 6	Homo sapiens Fc receptor-like 6 (FCRL6), mRNA [NM_001004310]
LOC96610	A_33_P3376958	2.77E-06	144.01	BMS1 homolog, ribosome assembly protein (yeast) pseudogene	Homo sapiens BMS1 homolog, ribosome assembly protein (yeast) pseudogene (LOC96610), non-coding RNA [NR_027293]
LOC100653210	A_33_P3424612	3.28E-06	84.58	ig kappa chain V-III region VG-like	immunoglobulin kappa variable 3-11 [Source:HGNC Symbol;Acc:5815] [ENST00000483158]
KLRC4	A_23_P218058	3.28E-06	17.81	killer cell lectin-like receptor subfamily C, member 4	Homo sapiens killer cell lectin-like receptor subfamily C, member 4 (KLRC4), mRNA [NM_013431]
SCML4	A_33_P3387691	3.48E-06	226.38	sex comb on midleg-like 4 (Drosophila)	Homo sapiens sex comb on midleg-like 4 (Drosophila) (SCML4), mRNA [NM_198081]
RHOH	A_23_P58132	3.50E-06	36.81	ras homolog gene family, member H	Homo sapiens ras homolog gene family, member H (RHOH), mRNA [NM_004310]

CTSW	A_24_P396167	3.69E-06	21.47	cathepsin W	Homo sapiens cathepsin W (CTSW), mRNA [NM_001335]
GFI1	A_23_P257365	3.78E-06	40.11	growth factor independent 1 transcription repressor	Homo sapiens growth factor independent 1 transcription repressor (GFI1), transcript variant 1, mRNA [NM_005263]
EOMES	A_24_P97374	3.78E-06	27.45	eomesodermin	Homo sapiens eomesodermin (EOMES), mRNA [NM_005442]
ITGAL	A_23_P206806	3.91E-06	32.61	integrin, alpha L (antigen CD11A (p180), lymphocyte function-associated antigen 1; alpha polypeptide)	Homo sapiens integrin, alpha L (antigen CD11A (p180), lymphocyte function-associated antigen 1; alpha polypeptide) (ITGAL), transcript variant 1, mRNA [NM_002209]
ZNF683	A_33_P3208970	4.19E-06	68.08	zinc finger protein 683	Homo sapiens zinc finger protein 683 (ZNF683), transcript variant 1, mRNA [NM_001114759]
FCRL5	A_23_P201211	4.19E-06	64.45	Fc receptor-like 5	Homo sapiens Fc receptor-like 5 (FCRL5), transcript variant 1, mRNA [NM_031281]
SAMD3	A_23_P93524	4.30E-06	22.92	sterile alpha motif domain containing 3	Homo sapiens sterile alpha motif domain containing 3 (SAMD3), transcript variant 1, mRNA [NM_001017373]
TMC8	A_23_P346093	4.30E-06	16.67	transmembrane channel-like 8	Homo sapiens transmembrane channel-like 8 (TMC8), mRNA [NM_152468]
IGLL5	A_33_P3379039	4.38E-06	653.81	immunoglobulin lambda-like polypeptide 5	Homo sapiens immunoglobulin lambda-like polypeptide 5 (IGLL5), transcript variant 1, mRNA [NM_001178126]
GZMH	A_23_P128993	4.40E-06	69.72	granzyme H (cathepsin G-like 2, protein h-CCPX)	Homo sapiens granzyme H (cathepsin G-like 2, protein h-CCPX) (GZMH), mRNA [NM_033423]
ITK	A_23_P354151	4.87E-06	31.17	IL2-inducible T-cell kinase	Homo sapiens IL2-inducible T-cell kinase (ITK), mRNA [NM_005546]
UBASH3A	A_23_P6293	4.87E-06	22.83	ubiquitin associated and SH3 domain containing A	Homo sapiens ubiquitin associated and SH3 domain containing A (UBASH3A),

					transcript variant 1, mRNA [NM_018961]
ZAP70	A_24_P169234	5.21E-06	16.56	zeta-chain (TCR) associated protein kinase 70kDa	Homo sapiens zeta-chain (TCR) associated protein kinase 70kDa (ZAP70), transcript variant 1, mRNA [NM_001079]
SKAP1	A_23_P100730	5.44E-06	41.14	src kinase associated phosphoprotein 1	Homo sapiens src kinase associated phosphoprotein 1 (SKAP1), transcript variant 1, mRNA [NM_003726]
FBXL16	A_23_P406385	6.11E-06	29.65	F-box and leucine-rich repeat protein 16	Homo sapiens F-box and leucine-rich repeat protein 16 (FBXL16), mRNA [NM_153350]
NKG7	A_23_P119042	6.11E-06	12.41	natural killer cell group 7 sequence	Homo sapiens natural killer cell group 7 sequence (NKG7), mRNA [NM_005601]
CD3G	A_23_P98410	6.26E-06	33.30	CD3g molecule, gamma (CD3-TCR complex)	Homo sapiens CD3g molecule, gamma (CD3-TCR complex) (CD3G), mRNA [NM_000073]
CCL4	A_23_P207564	6.28E-06	20.66	chemokine (C-C motif) ligand 4	Homo sapiens chemokine (C-C motif) ligand 4 (CCL4), transcript variant 1, mRNA [NM_002984]
THEMIS	A_33_P3462422	6.89E-06	32.90	thymocyte selection associated	Homo sapiens thymocyte selection associated (THEMIS), transcript variant 1, mRNA [NM_001164685]
LOC100128420	A_32_P77102	6.99E-06	70.74	uncharacterized LOC100128420	Homo sapiens uncharacterized LOC100128420 (LOC100128420), transcript variant 1, non-coding RNA [NR_038461]
MCOLN2	A_23_P23639	7.09E-06	24.06	mucolipin 2	Homo sapiens mucolipin 2 (MCOLN2), mRNA [NM_153259]
MZB1	A_23_P84596	7.92E-06	97.01	marginal zone B and B1 cell-specific protein	Homo sapiens marginal zone B and B1 cell-specific protein (MZB1), mRNA [NM_016459]
GPR171	A_23_P253317	8.83E-06	21.65	G protein-coupled receptor 171	Homo sapiens G protein-coupled receptor 171 (GPR171), mRNA

					[NM_013308]
JAKMIP1	A_33_P3221114	8.83E-06	16.84	janus kinase and microtubule interacting protein 1	Homo sapiens janus kinase and microtubule interacting protein 1 (JAKMIP1), transcript variant 2, mRNA [NM_144720]
AMICA1	A_24_P192914	8.84E-06	19.44	adhesion molecule, interacts with CXADR antigen 1	Homo sapiens adhesion molecule, interacts with CXADR antigen 1 (AMICA1), transcript variant 2, mRNA [NM_153206]
MIAT	A_19_P00315524	9.00E-06	38.39		Homo sapiens myocardial infarction associated transcript (non-protein coding) (MIAT), transcript variant 1, non-coding RNA [NR_003491]
KLRC3	A_23_P128281	9.02E-06	14.55	killer cell lectin-like receptor subfamily C, member 3	Homo sapiens killer cell lectin-like receptor subfamily C, member 3 (KLRC3), transcript variant 2, mRNA [NM_007333]
CD6	A_23_P311875	9.05E-06	40.35	CD6 molecule	Homo sapiens CD6 molecule (CD6), mRNA [NM_006725]
TRAF3IP3	A_33_P3281403	9.94E-06	30.74	TRAF3 interacting protein 3	Homo sapiens TRAF3 interacting protein 3 (TRAF3IP3), mRNA [NM_025228]
PRKCQ	A_23_P1374	1.05E-05	16.80	protein kinase C, theta	Homo sapiens protein kinase C, theta (PRKCQ), transcript variant 1, mRNA [NM_006257]
KLRC1	A_23_P151046	1.23E-05	36.25	killer cell lectin-like receptor subfamily C, member 1	Homo sapiens killer cell lectin-like receptor subfamily C, member 1 (KLRC1), transcript variant 1, mRNA [NM_002259]
CD69	A_33_P3241021	1.27E-05	20.02	CD69 molecule	CD69 molecule [Source:HGNC Symbol;Acc:1694] [ENST00000416624]
TBX21	A_23_P141555	1.37E-05	16.40	T-box 21	Homo sapiens T-box 21 (TBX21), mRNA [NM_013351]
SLAMF6	A_33_P3303857	1.42E-05	15.64	SLAM family member 6	Homo sapiens SLAM family member 6

					(SLAMF6), transcript variant 1, mRNA [NM_001184714]
RUNX3	A_33_P3221748	1.48E-05	16.78	runt-related transcription factor 3	Homo sapiens runt-related transcription factor 3 (RUNX3), transcript variant 1, mRNA [NM_001031680]
IKZF1	A_33_P3842556	1.50E-05	17.42	IKAROS family zinc finger 1 (Ikaros)	Homo sapiens IKAROS family zinc finger 1 (Ikaros) (IKZF1), transcript variant 1, mRNA [NM_006060]
CXCR3	A_23_P114299	1.52E-05	38.47	chemokine (C-X-C motif) receptor 3	Homo sapiens chemokine (C-X-C motif) receptor 3 (CXCR3), transcript variant 1, mRNA [NM_001504]
FASLG	A_23_P369815	1.52E-05	17.71	Fas ligand (TNF superfamily, member 6)	Homo sapiens Fas ligand (TNF superfamily, member 6) (FASLG), mRNA [NM_000639]
CD8A	A_32_P163247	1.55E-05	25.01	CD8a molecule	Homo sapiens CD8a molecule (CD8A), transcript variant 1, mRNA [NM_001768]
STYK1	A_23_P13822	1.55E-05	14.72	serine/threonine/tyrosine kinase 1	Homo sapiens serine/threonine/tyrosine kinase 1 (STYK1), mRNA [NM_018423]
RNF212	A_33_P3273919	1.59E-05	-4.29	ring finger protein 212	Homo sapiens cDNA FLJ42587 fis, clone BRACE3009377. [AK124578]
CD96	A_23_P44155	1.69E-05	40.67	CD96 molecule	Homo sapiens CD96 molecule (CD96), transcript variant 1, mRNA [NM_198196]
ZNF831	A_32_P206479	1.71E-05	37.35	zinc finger protein 831	Homo sapiens zinc finger protein 831 (ZNF831), mRNA [NM_178457]
ZBP1	A_23_P259141	1.90E-05	22.02	Z-DNA binding protein 1	Homo sapiens Z-DNA binding protein 1 (ZBP1), transcript variant 1, mRNA [NM_030776]
MATK	A_23_P50678	1.90E-05	13.61	megakaryocyte-associated tyrosine kinase	Homo sapiens megakaryocyte-associated tyrosine kinase (MATK), transcript variant 1, mRNA [NM_139355]
sept-06	A_33_P3243554	1.90E-05	3.04	septin 6	Homo sapiens septin 6 (SEPT6), transcript variant V, mRNA [NM_145802]

IL2RG	A_23_P148473	1.92E-05	14.87	interleukin 2 receptor, gamma	Homo sapiens interleukin 2 receptor, gamma (IL2RG), mRNA [NM_000206]
C8orf80	A_33_P3246593	1.95E-05	17.49	chromosome 8 open reading frame 80	Homo sapiens chromosome 8 open reading frame 80 (C8orf80), mRNA [NM_001010906]
LY9	A_24_P324674	2.01E-05	12.66	lymphocyte antigen 9	Homo sapiens lymphocyte antigen 9 (LY9), transcript variant 1, mRNA [NM_002348]
CSF2RA	A_23_P501985	2.14E-05	6.06	colony stimulating factor 2 receptor, alpha, low-affinity (granulocyte-macrophage)	Homo sapiens colony stimulating factor 2 receptor, alpha, low-affinity (granulocyte-macrophage) (CSF2RA), transcript variant 6, mRNA [NM_172249]
POU2AF1	A_23_P312920	2.73E-05	105.43	POU class 2 associating factor 1	Homo sapiens POU class 2 associating factor 1 (POU2AF1), mRNA [NM_006235]
CD2	A_23_P161076	2.74E-05	37.71	CD2 molecule	Homo sapiens CD2 molecule (CD2), mRNA [NM_001767]
MAP4K1	A_23_P5002	2.74E-05	11.78	mitogen-activated protein kinase kinase kinase kinase 1	Homo sapiens mitogen-activated protein kinase kinase kinase kinase 1 (MAP4K1), transcript variant 1, mRNA [NM_001042600]
TNIP3	A_23_P386478	2.81E-05	3.77	TNFAIP3 interacting protein 3	Homo sapiens TNFAIP3 interacting protein 3 (TNIP3), transcript variant 1, mRNA [NM_024873]
IL21R	A_24_P227927	2.90E-05	15.69	interleukin 21 receptor	Homo sapiens interleukin 21 receptor (IL21R), transcript variant 2, mRNA [NM_181078]
ACAP1	A_23_P107336	2.91E-05	15.58	ArfGAP with coiled-coil, ankyrin repeat and PH domains 1	Homo sapiens ArfGAP with coiled-coil, ankyrin repeat and PH domains 1 (ACAP1), mRNA [NM_014716]
SLAMF7	A_24_P353638	2.95E-05	40.95	SLAM family member 7	Homo sapiens SLAM family member 7 (SLAMF7), mRNA [NM_021181]
SH2D3A	A_33_P3254320	2.95E-05	13.17	SH2 domain containing 3A	Homo sapiens SH2 domain containing 3A

					(SH2D3A), mRNA [NM_005490]
IL7R	A_23_P404494	3.05E-05	49.73	interleukin 7 receptor	Homo sapiens interleukin 7 receptor (IL7R), mRNA [NM_002185]
CCL5	A_23_P152838	3.17E-05	77.89	chemokine (C-C motif) ligand 5	Homo sapiens chemokine (C-C motif) ligand 5 (CCL5), mRNA [NM_002985]
CRTAM	A_23_P305092	3.17E-05	27.81	cytotoxic and regulatory T cell molecule	Homo sapiens cytotoxic and regulatory T cell molecule (CRTAM), mRNA [NM_019604]
PVRIG	A_33_P3351745	3.20E-05	16.05	poliovirus receptor related immunoglobulin domain containing	Homo sapiens poliovirus receptor related immunoglobulin domain containing (PVRIG), mRNA [NM_024070]
CD247	A_23_P34676	3.23E-05	17.93	CD247 molecule	Homo sapiens CD247 molecule (CD247), transcript variant 1, mRNA [NM_198053]
CXorf65	A_32_P101352	3.65E-05	16.71	chromosome X open reading frame 65	Homo sapiens chromosome X open reading frame 65 (CXorf65), transcript variant 1, mRNA [NM_001025265]
CYTIP	A_23_P90626	3.82E-05	12.10	cytohesin 1 interacting protein	Homo sapiens cytohesin 1 interacting protein (CYTIP), mRNA [NM_004288]
CD8B	A_23_P357881	3.82E-05	18.67	CD8b molecule	Homo sapiens CD8b molecule (CD8B), transcript variant 4, mRNA [NM_172102]
ITGB7	A_23_P76529	3.82E-05	7.28	integrin, beta 7	Homo sapiens integrin, beta 7 (ITGB7), mRNA [NM_000889]
LOC100505585	A_33_P3290089	3.88E-05	14.23	uncharacterized LOC100505585	PREDICTED: Homo sapiens hypothetical LOC100505585 (LOC100505585), miscRNA [XR_109536]
CXCR6	A_23_P109913	4.35E-05	45.73	chemokine (C-X-C motif) receptor 6	Homo sapiens chemokine (C-X-C motif) receptor 6 (CXCR6), mRNA [NM_006564]
CD48	A_32_P175934	4.49E-05	25.69	CD48 molecule	Homo sapiens CD48 molecule (CD48), mRNA [NM_001778]
CXCR4	A_23_P102000	4.57E-05	15.05	chemokine (C-X-C motif) receptor 4	Homo sapiens chemokine (C-X-C motif) receptor 4 (CXCR4), transcript variant 1, mRNA [NM_001008540]
KLRK1	A_24_P193093	4.57E-05	14.54	killer cell lectin-like receptor	Homo sapiens killer cell lectin-like

				subfamily K, member 1	receptor subfamily K, member 1 (KLRK1), mRNA [NM_007360]
KLRD1	A_23_P204208	5.02E-05	16.50	killer cell lectin-like receptor subfamily D, member 1	Homo sapiens killer cell lectin-like receptor subfamily D, member 1 (KLRD1), transcript variant 1, mRNA [NM_002262]
LAG3	A_23_P116942	5.18E-05	13.11	lymphocyte-activation gene 3	Homo sapiens lymphocyte-activation gene 3 (LAG3), mRNA [NM_002286]
KIAA0125	A_23_P140190	5.24E-05	23.21	KIAA0125	Homo sapiens KIAA0125 (KIAA0125), non-coding RNA [NR_026800]
PTPRCAP	A_33_P3295056	5.28E-05	15.10	protein tyrosine phosphatase, receptor type, C-associated protein	Homo sapiens protein tyrosine phosphatase, receptor type, C-associated protein (PTPRCAP), mRNA [NM_005608]
AMPD1	A_23_P51787	5.67E-05	32.64	adenosine monophosphate deaminase 1	Homo sapiens adenosine monophosphate deaminase 1 (AMPD1), transcript variant 1, mRNA [NM_000036]
SH2D1A	A_33_P3283619	5.71E-05	11.64	SH2 domain containing 1A	Homo sapiens SH2 domain containing 1A (SH2D1A), transcript variant 2, mRNA [NM_001114937]
CD40LG	A_33_P3250680	5.81E-05	37.87	CD40 ligand	Homo sapiens CD40 ligand (CD40LG), mRNA [NM_000074]
AOAH	A_23_P145718	6.05E-05	21.84	acyloxyacyl hydrolase (neutrophil)	Homo sapiens acyloxyacyl hydrolase (neutrophil) (AOAH), transcript variant 1, mRNA [NM_001637]
PTPN7	A_23_P201778	6.19E-05	14.81	protein tyrosine phosphatase, non-receptor type 7	Homo sapiens protein tyrosine phosphatase, non-receptor type 7 (PTPN7), transcript variant 2, mRNA [NM_080588]
PBX4	A_23_P90419	6.25E-05	43.68	pre-B-cell leukemia homeobox 4	Homo sapiens pre-B-cell leukemia homeobox 4 (PBX4), transcript variant 1, mRNA [NM_025245]
SYTL1	A_33_P3266744	6.25E-05	6.67	synaptotagmin-like 1	Homo sapiens synaptotagmin-like 1

					(SYTL1), transcript variant 2, mRNA [NM_032872]
DEF6	A_23_P321913	6.37E-05	13.18	differentially expressed in FDCP 6 homolog (mouse)	Homo sapiens differentially expressed in FDCP 6 homolog (mouse) (DEF6), mRNA [NM_022047]
CD27	A_23_P48088	6.67E-05	14.86	CD27 molecule	Homo sapiens CD27 molecule (CD27), mRNA [NM_001242]
PRDM1	A_23_P350451	6.82E-05	26.79	PR domain containing 1, with ZNF domain	Homo sapiens PR domain containing 1, with ZNF domain (PRDM1), transcript variant 1, mRNA [NM_001198]
PTPN22	A_33_P3241984	7.35E-05	16.75	protein tyrosine phosphatase, non-receptor type 22 (lymphoid)	Homo sapiens protein tyrosine phosphatase, non-receptor type 22 (lymphoid) (PTPN22), transcript variant 1, mRNA [NM_015967]
S100Z	A_24_P12136	7.63E-05	6.91	S100 calcium binding protein Z	Homo sapiens S100 calcium binding protein Z (S100Z), mRNA [NM_130772]
TARP	A_33_P3225625	8.29E-05	14.67	TCR gamma alternate reading frame protein	Homo sapiens TCR gamma alternate reading frame protein (TARP), nuclear gene encoding mitochondrial protein, transcript variant 1, mRNA [NM_001003799]
CD244	A_23_P85453	8.60E-05	11.50	CD244 molecule, natural killer cell receptor 2B4	Homo sapiens CD244 molecule, natural killer cell receptor 2B4 (CD244), transcript variant 1, mRNA [NM_016382]
MEI1	A_23_P211561	8.73E-05	28.99	meiosis inhibitor 1	Homo sapiens meiosis inhibitor 1 (MEI1), mRNA [NM_152513]
PARP8	A_23_P121898	9.04E-05	4.98	poly (ADP-ribose) polymerase family, member 8	Homo sapiens poly (ADP-ribose) polymerase family, member 8 (PARP8), transcript variant 2, mRNA [NM_024615]
RAB39B	A_24_P408603	9.24E-05	14.79	RAB39B, member RAS oncogene family	Homo sapiens RAB39B, member RAS oncogene family (RAB39B), mRNA [NM_171998]
IL2RB	A_24_P203000	9.29E-05	21.42	interleukin 2 receptor, beta	Homo sapiens interleukin 2 receptor,

					beta (IL2RB), mRNA [NM_000878]
LAX1	A_23_P438	9.78E-05	17.61	lymphocyte transmembrane adaptor 1	Homo sapiens lymphocyte transmembrane adaptor 1 (LAX1), transcript variant 1, mRNA [NM_017773]
LINC00256B	A_32_P99347	1.04E-04	5.22	long intergenic non-protein coding RNA 256B	Homo sapiens long intergenic non-protein coding RNA 256B (LINC00256B), non-coding RNA [NR_024376]
BAG1	A_33_P3290919	1.04E-04	-2.36	BCL2-associated athanogene	Homo sapiens BCL2-associated athanogene (BAG1), transcript variant 1, mRNA [NM_001172415]
CD3E	A_23_P416747	1.13E-04	7.28	CD3e molecule, epsilon (CD3-TCR complex)	Homo sapiens CD3e molecule, epsilon (CD3-TCR complex) (CD3E), mRNA [NM_000733]
CD84	A_24_P362193	1.14E-04	11.12	CD84 molecule	Homo sapiens CD84 molecule (CD84), transcript variant 2, mRNA [NM_003874]
ITGA4	A_23_P56505	1.14E-04	14.34	integrin, alpha 4 (antigen CD49D, alpha 4 subunit of VLA-4 receptor)	Homo sapiens integrin, alpha 4 (antigen CD49D, alpha 4 subunit of VLA-4 receptor) (ITGA4), mRNA [NM_000885]
RAB37	A_33_P3314594	1.21E-04	4.45	RAB37, member RAS oncogene family	Homo sapiens RAB37, member RAS oncogene family (RAB37), transcript variant 3, mRNA [NM_175738]
sept-01	A_23_P21057	1.21E-04	24.25	septin 1	Homo sapiens septin 1 (SEPT1), mRNA [NM_052838]
TRAT1	A_23_P212568	1.24E-04	18.76	T cell receptor associated transmembrane adaptor 1	Homo sapiens T cell receptor associated transmembrane adaptor 1 (TRAT1), mRNA [NM_016388]
DENND1C	A_23_P78608	1.30E-04	8.57	DENN/MADD domain containing 1C	Homo sapiens DENN/MADD domain containing 1C (DENND1C), mRNA [NM_024898]
SP140	A_24_P328504	1.30E-04	5.50	SP140 nuclear body protein	Homo sapiens SP140 nuclear body protein (SP140), transcript variant 1, mRNA [NM_007237]
LOC606724	A_33_P3242614	1.34E-04	11.05	coronin, actin binding protein,	Homo sapiens coronin, actin binding

				1A pseudogene	protein, 1A pseudogene (LOC606724), non-coding RNA [NR_002454]
CCL17	A_23_P26325	1.40E-04	18.55	chemokine (C-C motif) ligand 17	Homo sapiens chemokine (C-C motif) ligand 17 (CCL17), mRNA [NM_002987]
EXOC3L4	A_33_P3360972	1.40E-04	8.50	exocyst complex component 3-like 4	Homo sapiens exocyst complex component 3-like 4 (EXOC3L4), mRNA [NM_001077594]
BCL11B	A_23_P205738	1.45E-04	13.71	B-cell CLL/lymphoma 11B (zinc finger protein)	Homo sapiens B-cell CLL/lymphoma 11B (zinc finger protein) (BCL11B), transcript variant 1, mRNA [NM_138576]
LCK	A_33_P3382746	1.53E-04	10.23	lymphocyte-specific protein tyrosine kinase	Homo sapiens lymphocyte-specific protein tyrosine kinase (LCK), transcript variant 2, mRNA [NM_005356]
PRF1	A_23_P1473	1.53E-04	6.10	perforin 1 (pore forming protein)	Homo sapiens perforin 1 (pore forming protein) (PRF1), transcript variant 1, mRNA [NM_005041]
IL16	A_23_P61057	1.53E-04	5.71	interleukin 16	Homo sapiens interleukin 16 (IL16), transcript variant 1, mRNA [NM_004513]
SIT1	A_23_P43369	1.59E-04	13.19	signaling threshold regulating transmembrane adaptor 1	Homo sapiens signaling threshold regulating transmembrane adaptor 1 (SIT1), mRNA [NM_014450]
FLJ32255	A_19_P00320759	1.68E-04	4.12	uncharacterized LOC643977	PREDICTED: Homo sapiens hypothetical LOC643977 (FLJ32255), miscRNA [XR_108575]
S1PR4	A_33_P3281273	1.78E-04	7.14	sphingosine-1-phosphate receptor 4	Homo sapiens sphingosine-1-phosphate receptor 4 (S1PR4), mRNA [NM_003775]
CARD11	A_23_P82324	1.80E-04	7.34	caspase recruitment domain family, member 11	Homo sapiens caspase recruitment domain family, member 11 (CARD11), mRNA [NM_032415]
D4S234E	A_33_P3259135	1.81E-04	-13.28	DNA segment on chromosome 4 (unique) 234 expressed sequence	Homo sapiens DNA segment on chromosome 4 (unique) 234 expressed sequence (D4S234E), transcript variant 1, mRNA [NM_014392]

SCARNA17	A_32_P37592	1.85E-04	3.66	small Cajal body-specific RNA 17	Homo sapiens small Cajal body-specific RNA 17 (SCARNA17), guide RNA [NR_003003]
PYHIN1	A_24_P139604	2.09E-04	9.96	pyrin and HIN domain family, member 1	Homo sapiens pyrin and HIN domain family, member 1 (PYHIN1), transcript variant 4, mRNA [NM_198930]
EVI2B	A_23_P66694	2.10E-04	9.97	ecotropic viral integration site 2B	Homo sapiens ecotropic viral integration site 2B (EVI2B), mRNA [NM_006495]
IFRD2	A_23_P92132	2.11E-04	-2.10	interferon-related developmental regulator 2	Homo sapiens interferon-related developmental regulator 2 (IFRD2), mRNA [NM_006764]
CD3D	A_33_P3375541	2.51E-04	5.87	CD3d molecule, delta (CD3-TCR complex)	Homo sapiens CD3d molecule, delta (CD3-TCR complex) (CD3D), transcript variant 1, mRNA [NM_000732]
AIM1	A_23_P70785	2.51E-04	5.75	absent in melanoma 1	Homo sapiens absent in melanoma 1 (AIM1), mRNA [NM_001624]
SIRPG	A_23_P28857	2.55E-04	9.35	signal-regulatory protein gamma	Homo sapiens signal-regulatory protein gamma (SIRPG), transcript variant 1, mRNA [NM_018556]
GBP5	A_23_P74290	2.62E-04	17.44	guanylate binding protein 5	Homo sapiens guanylate binding protein 5 (GBP5), transcript variant 1, mRNA [NM_052942]
TAGAP	A_24_P354724	2.66E-04	17.92	T-cell activation RhoGTPase activating protein	Homo sapiens T-cell activation RhoGTPase activating protein (TAGAP), transcript variant 2, mRNA [NM_054114]
HCST	A_23_P39386	2.66E-04	9.82	hematopoietic cell signal transducer	Homo sapiens hematopoietic cell signal transducer (HCST), transcript variant 1, mRNA [NM_014266]
AIM2	A_32_P44394	2.70E-04	34.08	absent in melanoma 2	Homo sapiens absent in melanoma 2 (AIM2), mRNA [NM_004833]
SMPD3	A_23_P163567	2.70E-04	19.07	sphingomyelin phosphodiesterase 3, neutral membrane (neutral	Homo sapiens sphingomyelin phosphodiesterase 3, neutral membrane (neutral sphingomyelinase II) (SMPD3),

				sphingomyelinase II)	mRNA [NM_018667]
CASS4	A_23_P91283	2.71E-04	3.87	Cas scaffolding protein family member 4	Homo sapiens Cas scaffolding protein family member 4 (CASS4), transcript variant 2, mRNA [NM_020356]
GZMB	A_23_P117602	2.73E-04	7.28	granzyme B (granzyme 2, cytotoxic T-lymphocyte-associated serine esterase 1)	Homo sapiens granzyme B (granzyme 2, cytotoxic T-lymphocyte-associated serine esterase 1) (GZMB), mRNA [NM_004131]
TRIM4	A_24_P68247	2.74E-04	2.56	tripartite motif containing 4	Homo sapiens tripartite motif containing 4 (TRIM4), transcript variant alpha, mRNA [NM_033017]
PNOC	A_23_P253321	2.79E-04	23.28	prepronociceptin	Homo sapiens prepronociceptin (PNOC), mRNA [NM_006228]
HSH2D	A_23_P153372	2.87E-04	19.02	hematopoietic SH2 domain containing	Homo sapiens hematopoietic SH2 domain containing (HSH2D), mRNA [NM_032855]
CST7	A_23_P68601	2.90E-04	10.67	cystatin F (leukocystatin)	Homo sapiens cystatin F (leukocystatin) (CST7), mRNA [NM_003650]
AGXT2L1	A_23_P257231	2.96E-04	-10.91	alanine-glyoxylate aminotransferase 2-like 1	Homo sapiens alanine-glyoxylate aminotransferase 2-like 1 (AGXT2L1), transcript variant 1, mRNA [NM_031279]
IKZF3	A_23_P376060	3.09E-04	14.07	IKAROS family zinc finger 3 (Aiolos)	Homo sapiens IKAROS family zinc finger 3 (Aiolos) (IKZF3), transcript variant 1, mRNA [NM_012481]
CDH9	A_23_P92999	3.09E-04	-4.13	cadherin 9, type 2 (T1-cadherin)	Homo sapiens cadherin 9, type 2 (T1-cadherin) (CDH9), mRNA [NM_016279]
CD37	A_24_P82749	3.12E-04	9.60	CD37 molecule	Homo sapiens CD37 molecule (CD37), transcript variant 1, mRNA [NM_001774]
TSC22D3	A_33_P3237634	3.12E-04	4.90	TSC22 domain family, member 3	Homo sapiens TSC22 domain family, member 3 (TSC22D3), transcript variant 2, mRNA [NM_004089]
MYB	A_23_P31073	3.17E-04	11.47	v-myb myeloblastosis viral oncogene homolog (avian)	Homo sapiens v-myb myeloblastosis viral oncogene homolog (avian) (MYB),

					transcript variant 2, mRNA [NM_005375]
DOCK10	A_23_P16722	3.21E-04	6.54	dedicator of cytokinesis 10	Homo sapiens dedicator of cytokinesis 10 (DOCK10), mRNA [NM_014689]
CASP8	A_24_P148499	3.27E-04	6.20	caspase 8, apoptosis-related cysteine peptidase	Homo sapiens caspase 8, apoptosis-related cysteine peptidase (CASP8), transcript variant E, mRNA [NM_033358]
TTC14	A_23_P212511	3.32E-04	2.15	tetratricopeptide repeat domain 14	Homo sapiens tetratricopeptide repeat domain 14 (TTC14), transcript variant 2, mRNA [NM_001042601]
PQLC3	A_23_P131375	3.34E-04	2.74	PQ loop repeat containing 3	Homo sapiens PQ loop repeat containing 3 (PQLC3), mRNA [NM_152391]
BIN2	A_23_P162386	3.35E-04	11.46	bridging integrator 2	Homo sapiens bridging integrator 2 (BIN2), mRNA [NM_016293]
BTK	A_23_P137139	3.38E-04	7.84	Bruton agammaglobulinemia tyrosine kinase	Homo sapiens Bruton agammaglobulinemia tyrosine kinase (BTK), mRNA [NM_000061]
LOC153684	A_33_P3323760	3.39E-04	3.45	uncharacterized LOC153684	Homo sapiens uncharacterized LOC153684 (LOC153684), non-coding RNA [NR_015447]
PSTPIP1	A_23_P48997	3.49E-04	5.22	proline-serine-threonine phosphatase interacting protein 1	Homo sapiens proline-serine-threonine phosphatase interacting protein 1 (PSTPIP1), mRNA [NM_003978]
PIWIL4	A_23_P427760	3.50E-04	3.07	piwi-like 4 (Drosophila)	Homo sapiens piwi-like 4 (Drosophila) (PIWIL4), mRNA [NM_152431]
CNTRL	A_33_P3257554	3.51E-04	3.30	centriolin	Homo sapiens centriolin (CNTRL), mRNA [NM_007018]
RASAL3	A_23_P79069	3.53E-04	7.13	RAS protein activator like 3	Homo sapiens RAS protein activator like 3 (RASAL3), mRNA [NM_022904]
CLNK	A_33_P3267989	3.57E-04	12.15	cytokine-dependent hematopoietic cell linker	Homo sapiens cytokine-dependent hematopoietic cell linker (CLNK), mRNA [NM_052964]
SMAP2	A_23_P406330	3.83E-04	3.91	small ArfGAP2	Homo sapiens small ArfGAP2 (SMAP2), transcript variant 1, mRNA [NM_022733]

CXCL9	A_23_P18452	3.94E-04	64.05	chemokine (C-X-C motif) ligand 9	Homo sapiens chemokine (C-X-C motif) ligand 9 (CXCL9), mRNA [NM_002416]
PTPRC	A_23_P12392	4.01E-04	11.10	protein tyrosine phosphatase, receptor type, C	Homo sapiens protein tyrosine phosphatase, receptor type, C (PTPRC), transcript variant 4, mRNA [NM_080923]
CLEC9A	A_33_P3319022	4.01E-04	5.85	C-type lectin domain family 9, member A	Homo sapiens C-type lectin domain family 9, member A (CLEC9A), mRNA [NM_207345]
LRRC17	A_23_P253958	4.01E-04	5.44	leucine rich repeat containing 17	Homo sapiens leucine rich repeat containing 17 (LRRC17), transcript variant 2, mRNA [NM_005824]
PRKCB	A_23_P420281	4.05E-04	10.04	protein kinase C, beta	Homo sapiens protein kinase C, beta (PRKCB), transcript variant 2, mRNA [NM_002738]
FGD3	A_24_P153840	4.17E-04	4.91	FYVE, RhoGEF and PH domain containing 3	Homo sapiens FYVE, RhoGEF and PH domain containing 3 (FGD3), transcript variant 2, mRNA [NM_033086]
AVEN	A_23_P100074	4.23E-04	-2.25	apoptosis, caspase activation inhibitor	Homo sapiens apoptosis, caspase activation inhibitor (AVEN), mRNA [NM_020371]
RUNX2	A_32_P161762	4.37E-04	5.98	runt-related transcription factor 2	Homo sapiens runt-related transcription factor 2 (RUNX2), transcript variant 3, mRNA [NM_004348]
XCL1	A_24_P45476	4.38E-04	19.99	chemokine (C motif) ligand 1	Homo sapiens chemokine (C motif) ligand 1 (XCL1), mRNA [NM_002995]
PRAM1	A_33_P3330608	4.45E-04	5.24	PML-RARA regulated adaptor molecule 1	Homo sapiens PML-RARA regulated adaptor molecule 1 (PRAM1), mRNA [NM_032152]
CCDC88C	A_24_P289170	4.45E-04	5.06	coiled-coil domain containing 88C	Homo sapiens coiled-coil domain containing 88C (CCDC88C), mRNA [NM_001080414]
EMB	A_24_P684186	4.62E-04	6.63	embigin	Homo sapiens embigin (EMB), mRNA [NM_198449]

C2	A_33_P3404601	4.68E-04	15.72	complement component 2	Homo sapiens complement component 2 (C2), transcript variant 3, mRNA [NM_001178063]
FBLIM1	A_23_P337934	4.68E-04	-2.54	filamin binding LIM protein 1	Homo sapiens filamin binding LIM protein 1 (FBLIM1), transcript variant 1, mRNA [NM_017556]
GYLTL1B	A_23_P104617	4.95E-04	-2.55	glycosyltransferase-like 1B	Homo sapiens glycosyltransferase-like 1B (GYLTL1B), mRNA [NM_152312]
C1orf105	A_23_P46584	5.24E-04	-6.89	chromosome 1 open reading frame 105	Homo sapiens chromosome 1 open reading frame 105 (C1orf105), mRNA [NM_139240]
CD5	A_33_P3298990	5.35E-04	20.57	CD5 molecule	Homo sapiens CD5 molecule (CD5), mRNA [NM_014207]
SUSD3	A_23_P401076	5.47E-04	7.21	sushi domain containing 3	Homo sapiens sushi domain containing 3 (SUSD3), mRNA [NM_145006]
LOC439949	A_32_P232559	5.76E-04	12.66	uncharacterized LOC439949	Homo sapiens uncharacterized LOC439949 (LOC439949), transcript variant 1, non-coding RNA [NR_036502]
APBB1IP	A_24_P232365	5.77E-04	6.16	amyloid beta (A4) precursor protein-binding, family B, member 1 interacting protein	Homo sapiens amyloid beta (A4) precursor protein-binding, family B, member 1 interacting protein (APBB1IP), mRNA [NM_019043]
CNIH2	A_24_P203315	5.77E-04	-3.34	cornichon homolog 2 (Drosophila)	Homo sapiens cornichon homolog 2 (Drosophila) (CNIH2), mRNA [NM_182553]
TRIM34	A_23_P124190	5.77E-04	2.72	tripartite motif containing 34	Homo sapiens tripartite motif containing 34 (TRIM34), transcript variant 3, mRNA [NM_130390]
KLRB1	A_23_P99275	5.82E-04	17.68	killer cell lectin-like receptor subfamily B, member 1	Homo sapiens killer cell lectin-like receptor subfamily B, member 1 (KLRB1), mRNA [NM_002258]
CAMK4	A_23_P250347	5.83E-04	6.16	calcium/calmodulin-dependent protein kinase IV	Homo sapiens calcium/calmodulin-dependent protein kinase IV (CAMK4),

					mRNA [NM_001744]
DOCK2	A_24_P408704	5.87E-04	10.60	dedicator of cytokinesis 2	Homo sapiens dedicator of cytokinesis 2 (DOCK2), mRNA [NM_004946]
TXNDC3	A_23_P259611	5.87E-04	10.56	thioredoxin domain containing 3 (spermatozoa)	Homo sapiens thioredoxin domain containing 3 (spermatozoa) (TXNDC3), mRNA [NM_016616]
TRERF1	A_23_P59022	6.07E-04	4.43	transcriptional regulating factor 1	Homo sapiens transcriptional regulating factor 1 (TRERF1), mRNA [NM_033502]
LOC100130899	A_33_P3240747	6.12E-04	-4.43	uncharacterized LOC100130899	Homo sapiens uncharacterized LOC100130899 (LOC100130899), non-coding RNA [NR_039988]
ATP2A2	A_24_P141786	6.23E-04	-3.34	ATPase, Ca++ transporting, cardiac muscle, slow twitch 2	Homo sapiens ATPase, Ca++ transporting, cardiac muscle, slow twitch 2 (ATP2A2), transcript variant a, mRNA [NM_001681]
TMEM79	A_33_P3320272	6.23E-04	2.31	transmembrane protein 79	Homo sapiens transmembrane protein 79 (TMEM79), transcript variant 1, mRNA [NM_032323]
LOC728978	A_32_P52153	6.27E-04	-4.98	uncharacterized LOC728978	Homo sapiens uncharacterized LOC728978 (LOC728978), non-coding RNA [NR_038453]
LOC727869	A_32_P110016	6.29E-04	2.33	uncharacterized LOC727869	PREDICTED: Homo sapiens hypothetical LOC727869 (LOC727869), miscRNA [XR_111211]
ZNF219	A_24_P35891	6.29E-04	-2.08	zinc finger protein 219	Homo sapiens zinc finger protein 219 (ZNF219), transcript variant 1, mRNA [NM_016423]
RGS1	A_23_P97141	6.34E-04	19.41	regulator of G-protein signaling 1	Homo sapiens regulator of G-protein signaling 1 (RGS1), mRNA [NM_002922]
RGL4	A_23_P306941	6.64E-04	14.09	ral guanine nucleotide dissociation stimulator-like 4	Homo sapiens ral guanine nucleotide dissociation stimulator-like 4 (RGL4), mRNA [NM_153615]
SNX10	A_24_P98109	6.69E-04	9.76	sorting nexin 10	Homo sapiens sorting nexin 10 (SNX10),

					transcript variant 2, mRNA [NM_013322]
NCF1	A_23_P42746	6.69E-04	9.42	neutrophil cytosolic factor 1	Homo sapiens neutrophil cytosolic factor 1 (NCF1), mRNA [NM_000265]
TRIM14	A_23_P216655	6.69E-04	6.84	tripartite motif containing 14	Homo sapiens tripartite motif containing 14 (TRIM14), transcript variant 1, mRNA [NM_014788]
FCN1	A_23_P157875	6.76E-04	13.46	ficolin (collagen/fibrinogen domain containing) 1	Homo sapiens ficolin (collagen/fibrinogen domain containing) 1 (FCN1), mRNA [NM_002003]
SV2C	A_33_P3314301	6.88E-04	-3.53	synaptic vesicle glycoprotein 2C	Homo sapiens synaptic vesicle glycoprotein 2C (SV2C), mRNA [NM_014979]
CORO1A	A_23_P106761	6.92E-04	4.87	coronin, actin binding protein, 1A	Homo sapiens coronin, actin binding protein, 1A (CORO1A), transcript variant 2, mRNA [NM_007074]
NDC80	A_23_P50108	6.95E-04	5.66	NDC80 homolog, kinetochore complex component (S. cerevisiae)	Homo sapiens NDC80 homolog, kinetochore complex component (S. cerevisiae) (NDC80), mRNA [NM_006101]
IL23A	A_23_P76078	7.08E-04	3.23	interleukin 23, alpha subunit p19	Homo sapiens interleukin 23, alpha subunit p19 (IL23A), mRNA [NM_016584]
CCR2	A_23_P212354	7.09E-04	12.92	chemokine (C-C motif) receptor 2	Homo sapiens chemokine (C-C motif) receptor 2 (CCR2), transcript variant A, mRNA [NM_001123041]
OXER1	A_33_P3222203	7.28E-04	2.62	oxoeicosanoid (OXE) receptor 1	Homo sapiens oxoeicosanoid (OXE) receptor 1 (OXER1), mRNA [NM_148962]
RBPMS2	A_23_P100056	7.53E-04	-3.15	RNA binding protein with multiple splicing 2	Homo sapiens RNA binding protein with multiple splicing 2 (RBPMS2), mRNA [NM_194272]
CCR5	A_23_P412321	7.62E-04	13.49	chemokine (C-C motif) receptor 5	Homo sapiens chemokine (C-C motif) receptor 5 (CCR5), transcript variant A,

					mRNA [NM_000579]
PPP1R1A	A_24_P13285	7.70E-04	-4.29	protein phosphatase 1, regulatory (inhibitor) subunit 1A	Homo sapiens protein phosphatase 1, regulatory (inhibitor) subunit 1A (PPP1R1A), mRNA [NM_006741]
TRAF1	A_24_P89891	7.95E-04	5.10	TNF receptor-associated factor 1	Homo sapiens TNF receptor-associated factor 1 (TRAF1), transcript variant 1, mRNA [NM_005658]
LOC100130000	A_33_P3245480	7.95E-04	4.03	phosphodiesterase 4D interacting protein pseudogene	Homo sapiens phosphodiesterase 4D interacting protein pseudogene (LOC100130000), non-coding RNA [NR_033189]
CCDC88B	A_24_P50950	7.97E-04	7.07	coiled-coil domain containing 88B	coiled-coil domain containing 88B [Source:HGNC Symbol;Acc:26757] [ENST00000492980]
LOC401847	A_24_P101642	8.00E-04	37.25	ig heavy chain V-III region VH26-like	PREDICTED: Homo sapiens ig heavy chain V-III region VH26-like (LOC401847), mRNA [XM_001718104]
CHDH	A_23_P357185	8.00E-04	-3.08	choline dehydrogenase	Homo sapiens choline dehydrogenase (CHDH), nuclear gene encoding mitochondrial protein, mRNA [NM_018397]
BLK	A_23_P31725	8.11E-04	12.33	B lymphoid tyrosine kinase	Homo sapiens B lymphoid tyrosine kinase (BLK), mRNA [NM_001715]
C14orf182	A_24_P263910	8.11E-04	7.04	chromosome 14 open reading frame 182	chromosome 14 open reading frame 182 [Source:HGNC Symbol;Acc:27503] [ENST00000529902]
HMHA1	A_33_P3318414	8.16E-04	7.70	histocompatibility (minor) HA-1	Homo sapiens histocompatibility (minor) HA-1 (HMHA1), mRNA [NM_012292]
ZBTB32	A_23_P131024	8.20E-04	9.13	zinc finger and BTB domain containing 32	Homo sapiens zinc finger and BTB domain containing 32 (ZBTB32), mRNA [NM_014383]
ARHGDIB	A_23_P151075	8.26E-04	2.75	Rho GDP dissociation inhibitor (GDI) beta	Homo sapiens Rho GDP dissociation inhibitor (GDI) beta (ARHGDIB), mRNA

					[NM_001175]
RTN1	A_23_P140290	8.38E-04	7.61	reticulon 1	Homo sapiens reticulon 1 (RTN1), transcript variant 1, mRNA [NM_021136]
LGALS2	A_23_P120902	8.49E-04	43.40	lectin, galactoside-binding, soluble, 2	Homo sapiens lectin, galactoside-binding, soluble, 2 (LGALS2), mRNA [NM_006498]
TNFRSF17	A_23_P37736	8.57E-04	18.53	tumor necrosis factor receptor superfamily, member 17	Homo sapiens tumor necrosis factor receptor superfamily, member 17 (TNFRSF17), mRNA [NM_001192]
RLTPR	A_33_P3294222	8.57E-04	4.07	RGD motif, leucine rich repeats, tropomodulin domain and proline-rich containing	Homo sapiens RGD motif, leucine rich repeats, tropomodulin domain and proline-rich containing (RLTPR), mRNA [NM_001013838]
DUSP13	A_23_P104471	8.57E-04	-2.99	dual specificity phosphatase 13	Homo sapiens dual specificity phosphatase 13 (DUSP13), transcript variant 1, mRNA [NM_001007271]
DPEP2	A_23_P118025	8.59E-04	9.69	dipeptidase 2	Homo sapiens dipeptidase 2 (DPEP2), mRNA [NM_022355]
FYB	A_24_P393740	8.71E-04	14.77	FYN binding protein	Homo sapiens FYN binding protein (FYB), transcript variant 1, mRNA [NM_001465]
KIF20B	A_33_P3215239	8.89E-04	2.81	kinesin family member 20B	Homo sapiens kinesin family member 20B (KIF20B), mRNA [NM_016195]
LRRK2	A_33_P3369058	8.92E-04	3.27	leucine-rich repeat kinase 2	Homo sapiens leucine-rich repeat kinase 2 (LRRK2), mRNA [NM_198578]
PLXNC1	A_23_P414958	9.02E-04	4.36	plexin C1	Homo sapiens plexin C1 (PLXNC1), transcript variant 1, mRNA [NM_005761]
STK4	A_24_P94054	9.12E-04	2.48	serine/threonine kinase 4	Homo sapiens serine/threonine kinase 4 (STK4), mRNA [NM_006282]
SLAMF8	A_23_P200138	9.27E-04	13.51	SLAM family member 8	Homo sapiens SLAM family member 8 (SLAMF8), mRNA [NM_020125]
RGS9	A_23_P66881	9.38E-04	9.84	regulator of G-protein signaling 9	Homo sapiens regulator of G-protein signaling 9 (RGS9), transcript variant 1, mRNA [NM_003835]

NBPF10	A_32_P83465	9.44E-04	2.22	neuroblastoma breakpoint family, member 10	Homo sapiens neuroblastoma breakpoint family, member 10 (NBPF10), mRNA [NM_001039703]
LOC388692	A_33_P3352887	9.61E-04	4.39	uncharacterized LOC388692	Homo sapiens uncharacterized LOC388692 (LOC388692), non-coding RNA [NR_027002]
RBM38	A_23_P17430	9.61E-04	-2.45	RNA binding motif protein 38	Homo sapiens RNA binding motif protein 38 (RBM38), transcript variant 1, mRNA [NM_017495]
PTPRU	A_33_P3309491	9.84E-04	-2.62	protein tyrosine phosphatase, receptor type, U	Homo sapiens protein tyrosine phosphatase, receptor type, U (PTPRU), transcript variant 3, mRNA [NM_005704]
GLIS1	A_23_P316612	1.01E-03	6.21	GLIS family zinc finger 1	Homo sapiens GLIS family zinc finger 1 (GLIS1), mRNA [NM_147193]
RAC2	A_23_P218770	1.02E-03	4.14	ras-related C3 botulinum toxin substrate 2 (rho family, small GTP binding protein Rac2)	Homo sapiens ras-related C3 botulinum toxin substrate 2 (rho family, small GTP binding protein Rac2) (RAC2), mRNA [NM_002872]
BATF	A_23_P128974	1.04E-03	13.33	basic leucine zipper transcription factor, ATF-like	Homo sapiens basic leucine zipper transcription factor, ATF-like (BATF), mRNA [NM_006399]
PIK3IP1	A_23_P109488	1.06E-03	5.57	phosphoinositide-3-kinase interacting protein 1	Homo sapiens phosphoinositide-3-kinase interacting protein 1 (PIK3IP1), transcript variant 1, mRNA [NM_052880]
GZMK	A_23_P156218	1.08E-03	31.71	granzyme K (granzyme 3; tryptase II)	Homo sapiens granzyme K (granzyme 3; tryptase II) (GZMK), mRNA [NM_002104]
SELPLG	A_23_P64860	1.09E-03	5.55	selectin P ligand	Homo sapiens selectin P ligand (SELPLG), transcript variant 2, mRNA [NM_003006]
PIK3CG	A_33_P3304170	1.09E-03	5.37	phosphoinositide-3-kinase, catalytic, gamma polypeptide	Homo sapiens phosphoinositide-3-kinase, catalytic, gamma polypeptide (PIK3CG), mRNA [NM_002649]
GLYAT	A_23_P403886	1.09E-03	-2.11	glycine-N-acyltransferase	Homo sapiens glycine-N-acyltransferase (GLYAT), nuclear gene encoding

					mitochondrial protein, transcript variant 2, mRNA [NM_005838]
GZMA	A_33_P3376821	1.10E-03	24.70	granzyme A (granzyme 1, cytotoxic T-lymphocyte-associated serine esterase 3)	Homo sapiens granzyme A (granzyme 1, cytotoxic T-lymphocyte-associated serine esterase 3) (GZMA), mRNA [NM_006144]
FERMT2	A_33_P3344482	1.14E-03	-2.01	fermitin family member 2	Homo sapiens fermitin family member 2 (FERMT2), transcript variant 3, mRNA [NM_001135000]
CLEC12A	A_23_P128470	1.14E-03	4.20	C-type lectin domain family 12, member A	Homo sapiens C-type lectin domain family 12, member A (CLEC12A), transcript variant 1, mRNA [NM_138337]
FAM113B	A_23_P36888	1.14E-03	3.56	family with sequence similarity 113, member B	Homo sapiens family with sequence similarity 113, member B (FAM113B), mRNA [NM_138371]
PIM2	A_24_P379104	1.18E-03	7.45	pim-2 oncogene	Homo sapiens pim-2 oncogene (PIM2), mRNA [NM_006875]
TIFAB	A_33_P3380383	1.23E-03	16.91	TRAF-interacting protein with forkhead-associated domain, family member B	Homo sapiens TRAF-interacting protein with forkhead-associated domain, family member B (TIFAB), mRNA [NM_001099221]
RIMBP3	A_23_P154962	1.28E-03	5.75	RIMS binding protein 3	Homo sapiens RIMS binding protein 3 (RIMBP3), mRNA [NM_015672]
SLA	A_23_P216340	1.32E-03	7.82	Src-like-adaptor	Homo sapiens Src-like-adaptor (SLA), transcript variant 1, mRNA [NM_001045556]
ESRRA	A_23_P1585	1.34E-03	-2.20	estrogen-related receptor alpha	Homo sapiens estrogen-related receptor alpha (ESRRA), mRNA [NM_004451]
LAT	A_23_P44112	1.35E-03	3.64	linker for activation of T cells	Homo sapiens linker for activation of T cells (LAT), transcript variant 1, mRNA [NM_014387]
CDKN3	A_23_P48669	1.38E-03	-2.39	cyclin-dependent kinase inhibitor 3	Homo sapiens cyclin-dependent kinase inhibitor 3 (CDKN3), transcript variant 1,

					mRNA [NM_005192]
TRMU	A_33_P3433156	1.41E-03	-2.17	tRNA 5-methylaminomethyl-2-thiouridylate methyltransferase	Homo sapiens tRNA 5-methylaminomethyl-2-thiouridylate methyltransferase (TRMU), nuclear gene encoding mitochondrial protein, transcript variant 1, mRNA [NM_018006]
WNT10A	A_23_P102117	1.42E-03	3.54	wingless-type MMTV integration site family, member 10A	Homo sapiens wingless-type MMTV integration site family, member 10A (WNT10A), mRNA [NM_025216]
CD52	A_23_P85800	1.44E-03	10.80	CD52 molecule	Homo sapiens CD52 molecule (CD52), mRNA [NM_001803]
FANCA	A_23_P206441	1.44E-03	5.00	Fanconi anemia, complementation group A	Homo sapiens Fanconi anemia, complementation group A (FANCA), transcript variant 1, mRNA [NM_000135]
DENND2D	A_23_P85952	1.46E-03	6.17	DENN/MADD domain containing 2D	Homo sapiens DENN/MADD domain containing 2D (DENND2D), mRNA [NM_024901]
LOC401320	A_33_P3432961	1.46E-03	2.64	uncharacterized LOC401320	Homo sapiens uncharacterized LOC401320 (LOC401320), non-coding RNA [NR_038889]
CD226	A_33_P3413558	1.47E-03	6.47	CD226 molecule	Homo sapiens CD226 molecule (CD226), mRNA [NM_006566]
FURIN	A_23_P3368	1.47E-03	-2.29	furin (paired basic amino acid cleaving enzyme)	Homo sapiens furin (paired basic amino acid cleaving enzyme) (FURIN), mRNA [NM_002569]
C1orf162	A_23_P412562	1.49E-03	4.30	chromosome 1 open reading frame 162	Homo sapiens chromosome 1 open reading frame 162 (C1orf162), mRNA [NM_174896]
FOLH1	A_23_P47616	1.50E-03	3.25	folate hydrolase (prostate-specific membrane antigen) 1	Homo sapiens folate hydrolase (prostate-specific membrane antigen) 1 (FOLH1), transcript variant 1, mRNA [NM_004476]
NLRC3	A_23_P340019	1.51E-03	7.76	NLR family, CARD domain	Homo sapiens NLR family, CARD domain

				containing 3	containing 3 (NLRC3), mRNA [NM_178844]
SNX20	A_24_P13831	1.51E-03	4.30	sorting nexin 20	Homo sapiens sorting nexin 20 (SNX20), transcript variant 1, mRNA [NM_182854]
KBTBD10	A_23_P17190	1.52E-03	-3.90	kelch repeat and BTB (POZ) domain containing 10	Homo sapiens kelch repeat and BTB (POZ) domain containing 10 (KBTBD10), mRNA [NM_006063]
LOC115110	A_33_P3242820	1.52E-03	2.38	uncharacterized LOC115110	Homo sapiens uncharacterized LOC115110 (LOC115110), non-coding RNA [NR_026927]
LOC100130811	A_32_P65589	1.54E-03	13.61	ig heavy chain V-I region V35-like	PREDICTED: Homo sapiens ig heavy chain V-I region V35-like (LOC100130811), mRNA [XM_003403830]
RGS18	A_23_P302550	1.55E-03	7.37	regulator of G-protein signaling 18	Homo sapiens regulator of G-protein signaling 18 (RGS18), mRNA [NM_130782]
MMP25	A_23_P376557	1.56E-03	6.09	matrix metallopeptidase 25	Homo sapiens matrix metallopeptidase 25 (MMP25), mRNA [NM_022468]
TRIM46	A_33_P3390778	1.58E-03	3.58	tripartite motif containing 46	Homo sapiens tripartite motif containing 46 (TRIM46), mRNA [NM_025058]
SLIT2	A_23_P144348	1.59E-03	2.81	slit homolog 2 (Drosophila)	Homo sapiens slit homolog 2 (Drosophila) (SLIT2), mRNA [NM_004787]
MS4A6A	A_23_P36120	1.60E-03	5.41	membrane-spanning 4-domains, subfamily A, member 6A	Homo sapiens membrane-spanning 4-domains, subfamily A, member 6A (MS4A6A), transcript variant 2, mRNA [NM_022349]
ADAM8	A_24_P300777	1.62E-03	6.70	ADAM metallopeptidase domain 8	Homo sapiens ADAM metallopeptidase domain 8 (ADAM8), transcript variant 1, mRNA [NM_001109]
FRZB	A_23_P363778	1.62E-03	5.88	frizzled-related protein	Homo sapiens frizzled-related protein (FRZB), mRNA [NM_001463]

MYO1F	A_23_P142447	1.62E-03	5.46	myosin IF	Homo sapiens myosin IF (MYO1F), mRNA [NM_012335]
PHF21B	A_33_P3280666	1.63E-03	-2.38	PHD finger protein 21B	Homo sapiens PHD finger protein 21B (PHF21B), transcript variant 1, mRNA [NM_138415]
CCL3L3	A_24_P228130	1.68E-03	5.43	chemokine (C-C motif) ligand 3-like 3	Homo sapiens chemokine (C-C motif) ligand 3-like 3 (CCL3L3), mRNA [NM_001001437]
TMPRSS3	A_23_P80162	1.72E-03	2.16	transmembrane protease, serine 3	Homo sapiens transmembrane protease, serine 3 (TMPRSS3), transcript variant D, mRNA [NM_032405]
MPP2	A_33_P3360611	1.74E-03	-2.20	membrane protein, palmitoylated 2 (MAGUK p55 subfamily member 2)	Homo sapiens membrane protein, palmitoylated 2 (MAGUK p55 subfamily member 2) (MPP2), mRNA [NM_005374]
ASCL2	A_32_P171061	1.74E-03	8.34	achaete-scute complex homolog 2 (Drosophila)	Homo sapiens achaete-scute complex homolog 2 (Drosophila) (ASCL2), mRNA [NM_005170]
C9orf3	A_23_P73012	1.74E-03	-2.44	chromosome 9 open reading frame 3	Homo sapiens chromosome 9 open reading frame 3 (C9orf3), transcript variant 2, mRNA [NM_032823]
GTF3A	A_23_P25525	1.74E-03	-2.05	general transcription factor IIIA	Homo sapiens general transcription factor IIIA (GTF3A), mRNA [NM_002097]
DQX1	A_23_P56659	1.75E-03	2.43	DEAQ box RNA-dependent ATPase 1	Homo sapiens DEAQ box RNA-dependent ATPase 1 (DQX1), mRNA [NM_133637]
CCR7	A_23_P343398	1.76E-03	14.70	chemokine (C-C motif) receptor 7	Homo sapiens chemokine (C-C motif) receptor 7 (CCR7), mRNA [NM_001838]
LOC550112	A_19_P00320846	1.79E-03	2.74	uncharacterized LOC550112	Homo sapiens uncharacterized LOC550112 (LOC550112), non-coding RNA [NR_015439]
BLNK	A_33_P3363637	1.79E-03	7.62	B-cell linker	Homo sapiens B-cell linker (BLNK), transcript variant 1, mRNA [NM_013314]
UBD	A_23_P81898	1.84E-03	31.26	ubiquitin D	Homo sapiens ubiquitin D (UBD), mRNA

					[NM_006398]
TXNDC5	A_33_P3270451	1.84E-03	4.66	thioredoxin domain containing 5 (endoplasmic reticulum)	Homo sapiens thioredoxin domain containing 5 (endoplasmic reticulum) (TXNDC5), transcript variant 1, mRNA [NM_030810]
IL10RA	A_23_P203173	1.86E-03	6.28	interleukin 10 receptor, alpha	Homo sapiens interleukin 10 receptor, alpha (IL10RA), transcript variant 1, mRNA [NM_001558]
P2RY12	A_23_P143902	1.88E-03	5.07	purinergic receptor P2Y, G-protein coupled, 12	Homo sapiens purinergic receptor P2Y, G-protein coupled, 12 (P2RY12), transcript variant 1, mRNA [NM_022788]
HNMT	A_23_P56734	1.89E-03	2.54	histamine N-methyltransferase	Homo sapiens histamine N-methyltransferase (HNMT), transcript variant 1, mRNA [NM_006895]
DERL3	A_33_P3724157	1.93E-03	3.64	Der1-like domain family, member 3	Homo sapiens Der1-like domain family, member 3 (DERL3), transcript variant 3, mRNA [NM_198440]
DOCK8	A_32_P181077	1.94E-03	5.51	dedicator of cytokinesis 8	Homo sapiens dedicator of cytokinesis 8 (DOCK8), transcript variant 1, mRNA [NM_203447]
SOCS1	A_23_P420196	1.97E-03	3.93	suppressor of cytokine signaling 1	Homo sapiens suppressor of cytokine signaling 1 (SOCS1), mRNA [NM_003745]
TTYH1	A_23_P50815	1.99E-03	-2.53	tweety homolog 1 (Drosophila)	Homo sapiens tweety homolog 1 (Drosophila) (TTYH1), transcript variant 1, mRNA [NM_020659]
ABI3BP	A_33_P3411975	1.99E-03	4.60	ABI family, member 3 (NESH) binding protein	Homo sapiens ABI family, member 3 (NESH) binding protein (ABI3BP), mRNA [NM_015429]
CD7	A_33_P3226995	1.99E-03	9.00	CD7 molecule	Homo sapiens CD7 molecule (CD7), mRNA [NM_006137]
GVINP1	A_33_P3341616	1.99E-03	7.80	GTPase, very large interferon inducible pseudogene 1	Homo sapiens GTPase, very large interferon inducible pseudogene 1 (GVINP1), non-coding RNA [NR_003945]

AKNA	A_33_P3220047	1.99E-03	7.01	AT-hook transcription factor	Homo sapiens AT-hook transcription factor (AKNA), mRNA [NM_030767]
SLC4A8	A_33_P3419998	1.99E-03	5.81	solute carrier family 4, sodium bicarbonate cotransporter, member 8	Homo sapiens solute carrier family 4, sodium bicarbonate cotransporter, member 8 (SLC4A8), transcript variant 1, mRNA [NM_001039960]
APOBEC3H	A_32_P347617	2.02E-03	7.51	apolipoprotein B mRNA editing enzyme, catalytic polypeptide-like 3H	Homo sapiens apolipoprotein B mRNA editing enzyme, catalytic polypeptide-like 3H (APOBEC3H), transcript variant 2, mRNA [NM_181773]
HEPN1	A_33_P3298120	2.03E-03	-2.09	hepatocellular carcinoma, down-regulated 1	Homo sapiens hepatocellular carcinoma, down-regulated 1 (HEPN1), mRNA [NM_001037558]
EVI2A	A_23_P78092	2.03E-03	6.72	ecotropic viral integration site 2A	Homo sapiens ecotropic viral integration site 2A (EVI2A), transcript variant 1, mRNA [NM_001003927]
SPNS3	A_23_P100963	2.05E-03	4.43	spinster homolog 3 (Drosophila)	Homo sapiens spinster homolog 3 (Drosophila) (SPNS3), mRNA [NM_182538]
PPP2R2B	A_23_P213620	2.06E-03	9.74	protein phosphatase 2, regulatory subunit B, beta	Homo sapiens protein phosphatase 2, regulatory subunit B, beta (PPP2R2B), transcript variant 1, mRNA [NM_004576]
MYH6	A_23_P37167	2.06E-03	-4.92	myosin, heavy chain 6, cardiac muscle, alpha	Homo sapiens myosin, heavy chain 6, cardiac muscle, alpha (MYH6), mRNA [NM_002471]
LOC651536	A_33_P3257486	2.06E-03	4.34	immunoglobulin omega chain-like	immunoglobulin lambda variable 5-45 [Source:HGNC Symbol;Acc:5924] [ENST00000390296]
DLEC1	A_23_P18282	2.06E-03	4.26	deleted in lung and esophageal cancer 1	Homo sapiens deleted in lung and esophageal cancer 1 (DLEC1), transcript variant DLEC1-N1, mRNA [NM_007335]
CLEC4D	A_33_P3352578	2.08E-03	7.89	C-type lectin domain family 4, member D	Homo sapiens C-type lectin domain family 4, member D (CLEC4D), mRNA

					[NM_080387]
LCP2	A_23_P30547	2.08E-03	4.70	lymphocyte cytosolic protein 2 (SH2 domain containing leukocyte protein of 76kDa)	Homo sapiens lymphocyte cytosolic protein 2 (SH2 domain containing leukocyte protein of 76kDa) (LCP2), mRNA [NM_005565]
C6orf97	A_33_P3412087	2.10E-03	3.95	chromosome 6 open reading frame 97	Homo sapiens chromosome 6 open reading frame 97 (C6orf97), mRNA [NM_025059]
FAM105A	A_23_P133438	2.11E-03	5.51	family with sequence similarity 105, member A	Homo sapiens family with sequence similarity 105, member A (FAM105A), mRNA [NM_019018]
XLOC_003482	A_19_P00812050	2.13E-03	2.20		BROAD Institute lincRNA (XLOC_003482), lincRNA [TCONS_00008017]
CD53	A_23_P74547	2.13E-03	8.17	CD53 molecule	Homo sapiens CD53 molecule (CD53), transcript variant 1, mRNA [NM_001040033]
HCLS1	A_23_P73429	2.13E-03	4.97	hematopoietic cell-specific Lyn substrate 1	Homo sapiens hematopoietic cell-specific Lyn substrate 1 (HCLS1), mRNA [NM_005335]
JAKMIP2	A_33_P3255290	2.16E-03	5.44	janus kinase and microtubule interacting protein 2	Homo sapiens janus kinase and microtubule interacting protein 2 (JAKMIP2), mRNA [NM_014790]
MYO5A	A_24_P255218	2.16E-03	2.13	myosin VA (heavy chain 12, myoxin)	Homo sapiens myosin VA (heavy chain 12, myoxin) (MYO5A), transcript variant 1, mRNA [NM_000259]
LOC100132057	A_32_P515431	2.16E-03	5.03	phosphodiesterase 4D interacting protein pseudogene	PREDICTED: Homo sapiens hypothetical LOC100132057 (LOC100132057), miscRNA [XR_110830]
XLOC_008370	A_19_P00315529	2.16E-03	4.61		BROAD Institute lincRNA (XLOC_008370), lincRNA [TCONS_00018105]
ATP10D	A_33_P3311473	2.16E-03	2.89	ATPase, class V, type 10D	Homo sapiens ATPase, class V, type 10D

					(ATP10D), mRNA [NM_020453]
LPAR2	A_23_P101829	2.17E-03	6.31	lysophosphatidic acid receptor 2	Homo sapiens lysophosphatidic acid receptor 2 (LPAR2), mRNA [NM_004720]
ARHGAP30	A_33_P3213910	2.19E-03	3.15	Rho GTPase activating protein 30	Homo sapiens Rho GTPase activating protein 30 (ARHGAP30), transcript variant 2, mRNA [NM_181720]
CENPV	A_23_P313828	2.24E-03	-4.05	centromere protein V	Homo sapiens centromere protein V (CENPV), mRNA [NM_181716]
IL7	A_23_P8961	2.24E-03	6.23	interleukin 7	Homo sapiens interleukin 7 (IL7), transcript variant 1, mRNA [NM_000880]
LRRC8E	A_24_P348203	2.24E-03	-5.59	leucine rich repeat containing 8 family, member E	Homo sapiens leucine rich repeat containing 8 family, member E (LRRC8E), mRNA [NM_025061]
TRPM2	A_24_P27977	2.25E-03	3.77	transient receptor potential cation channel, subfamily M, member 2	Homo sapiens transient receptor potential cation channel, subfamily M, member 2 (TRPM2), transcript variant 1, mRNA [NM_003307]
LOC645769	A_33_P3418992	2.25E-03	-2.22	uncharacterized LOC645769	Homo sapiens cDNA FLJ41797 fis, clone NHNPC2000877. [AK123791]
C16orf54	A_33_P3227443	2.27E-03	11.20	chromosome 16 open reading frame 54	Homo sapiens chromosome 16 open reading frame 54 (C16orf54), mRNA [NM_175900]
SYT7	A_33_P3239185	2.29E-03	-3.28	synaptotagmin VII	Homo sapiens synaptotagmin VII (SYT7), transcript variant 1, mRNA [NM_001252065]
GFRA2	A_33_P3317580	2.30E-03	3.40	GDNF family receptor alpha 2	Homo sapiens GDNF family receptor alpha 2 (GFRA2), transcript variant 1, mRNA [NM_001495]
CORO2A	A_23_P135061	2.34E-03	3.97	coronin, actin binding protein, 2A	Homo sapiens coronin, actin binding protein, 2A (CORO2A), transcript variant 1, mRNA [NM_003389]
BTLA	A_33_P3358923	2.37E-03	8.62	B and T lymphocyte associated	Homo sapiens B and T lymphocyte associated (BTLA), transcript variant 1,

					mRNA [NM_181780]
FGD2	A_24_P140788	2.38E-03	3.67	FYVE, RhoGEF and PH domain containing 2	Homo sapiens FYVE, RhoGEF and PH domain containing 2 (FGD2), mRNA [NM_173558]
LAMA4	A_33_P3391596	2.38E-03	-2.86	laminin, alpha 4	Homo sapiens laminin, alpha 4 (LAMA4), transcript variant 1, mRNA [NM_001105206]
FAM26F	A_23_P7827	2.39E-03	11.76	family with sequence similarity 26, member F	Homo sapiens family with sequence similarity 26, member F (FAM26F), mRNA [NM_001010919]
SLC16A6	A_23_P152791	2.41E-03	6.97	solute carrier family 16, member 6 (monocarboxylic acid transporter 7)	Homo sapiens solute carrier family 16, member 6 (monocarboxylic acid transporter 7) (SLC16A6), transcript variant 2, mRNA [NM_004694]
PPP1CC	A_23_P204423	2.41E-03	-2.02	protein phosphatase 1, catalytic subunit, gamma isozyme	Homo sapiens protein phosphatase 1, catalytic subunit, gamma isozyme (PPP1CC), transcript variant 1, mRNA [NM_002710]
OCIAD2	A_23_P121702	2.45E-03	-2.11	OCIA domain containing 2	Homo sapiens OCIA domain containing 2 (OCIAD2), transcript variant 1, mRNA [NM_001014446]
IPCEF1	A_33_P3397995	2.45E-03	6.41	interaction protein for cytohesin exchange factors 1	Homo sapiens interaction protein for cytohesin exchange factors 1 (IPCEF1), transcript variant 1, mRNA [NM_001130699]
ANGPT1	A_23_P216023	2.48E-03	4.04	angiopoietin 1	Homo sapiens angiopoietin 1 (ANGPT1), transcript variant 1, mRNA [NM_001146]
NELL2	A_23_P10025	2.51E-03	9.52	NEL-like 2 (chicken)	Homo sapiens NEL-like 2 (chicken) (NELL2), transcript variant 2, mRNA [NM_006159]
TNNI2	A_23_P24784	2.51E-03	3.58	troponin I type 2 (skeletal, fast)	Homo sapiens troponin I type 2 (skeletal, fast) (TNNI2), transcript variant 1, mRNA [NM_003282]

GRIK3	A_33_P3246553	2.51E-03	-2.13	glutamate receptor, ionotropic, kainate 3	Homo sapiens glutamate receptor, ionotropic, kainate 3 (GRIK3), mRNA [NM_000831]
SASH3	A_24_P237443	2.52E-03	7.32	SAM and SH3 domain containing 3	Homo sapiens SAM and SH3 domain containing 3 (SASH3), mRNA [NM_018990]
ACP5	A_23_P142075	2.54E-03	7.31	acid phosphatase 5, tartrate resistant	Homo sapiens acid phosphatase 5, tartrate resistant (ACP5), transcript variant 4, mRNA [NM_001611]
LRMP	A_23_P98910	2.54E-03	4.73	lymphoid-restricted membrane protein	Homo sapiens lymphoid-restricted membrane protein (LRMP), transcript variant 1, mRNA [NM_006152]
SCARNA2	A_33_P3273584	2.54E-03	3.25	small Cajal body-specific RNA 2	Homo sapiens small Cajal body-specific RNA 2 (SCARNA2), guide RNA [NR_003023]
RTN4RL1	A_23_P432056	2.56E-03	-5.18	reticulon 4 receptor-like 1	Homo sapiens reticulon 4 receptor-like 1 (RTN4RL1), mRNA [NM_178568]
C2orf89	A_23_P255896	2.64E-03	2.82	chromosome 2 open reading frame 89	Homo sapiens chromosome 2 open reading frame 89 (C2orf89), mRNA [NM_001080824]
P2RY14	A_24_P165864	2.69E-03	2.65	purinergic receptor P2Y, G-protein coupled, 14	Homo sapiens purinergic receptor P2Y, G-protein coupled, 14 (P2RY14), transcript variant 2, mRNA [NM_014879]
SP140L	A_23_P337753	2.76E-03	3.30	SP140 nuclear body protein-like	Homo sapiens SP140 nuclear body protein-like (SP140L), mRNA [NM_138402]
PTK2	A_33_P3416707	2.81E-03	-3.30	PTK2 protein tyrosine kinase 2	PTK2 protein tyrosine kinase 2 [Source:HGNC Symbol;Acc:9611] [ENST00000342207]
GALNT6	A_33_P3215768	2.82E-03	7.89	UDP-N-acetyl-alpha-D-galactosamine:polypeptide N-acetylgalactosaminyltransferase 6 (GalNAc-T6)	Homo sapiens UDP-N-acetyl-alpha-D-galactosamine:polypeptide N-acetylgalactosaminyltransferase 6 (GalNAc-T6) (GALNT6), mRNA

					[NM_007210]
LCN9	A_33_P3423931	2.84E-03	-3.44	lipocalin 9	Homo sapiens lipocalin 9 (LCN9), mRNA [NM_001001676]
ERP27	A_23_P139687	2.85E-03	4.08	endoplasmic reticulum protein 27	Homo sapiens endoplasmic reticulum protein 27 (ERP27), mRNA [NM_152321]
EGLN1	A_23_P343935	2.86E-03	-2.08	egl nine homolog 1 (C. elegans)	Homo sapiens egl nine homolog 1 (C. elegans) (EGLN1), mRNA [NM_022051]
LOC100131096	A_24_P857624	2.87E-03	3.09	uncharacterized LOC100131096	Homo sapiens uncharacterized LOC100131096 (LOC100131096), non-coding RNA [NR_040071]
GRAP2	A_24_P10884	2.89E-03	2.39	GRB2-related adaptor protein 2	Homo sapiens GRB2-related adaptor protein 2 (GRAP2), mRNA [NM_004810]
GPR18	A_23_P14165	2.91E-03	5.53	G protein-coupled receptor 18	Homo sapiens G protein-coupled receptor 18 (GPR18), transcript variant 1, mRNA [NM_005292]
SEMA4D	A_24_P261169	3.00E-03	8.82	sema domain, immunoglobulin domain (Ig), transmembrane domain (TM) and short cytoplasmic domain, (semaphorin) 4D	Homo sapiens sema domain, immunoglobulin domain (Ig), transmembrane domain (TM) and short cytoplasmic domain, (semaphorin) 4D (SEMA4D), transcript variant 1, mRNA [NM_006378]
PTGDR	A_23_P393777	3.00E-03	5.17	prostaglandin D2 receptor (DP)	Homo sapiens prostaglandin D2 receptor (DP) (PTGDR), mRNA [NM_000953]
LAIR2	A_23_P209129	3.00E-03	4.39	leukocyte-associated immunoglobulin-like receptor 2	Homo sapiens leukocyte-associated immunoglobulin-like receptor 2 (LAIR2), transcript variant 1, mRNA [NM_002288]
SLC9A9	A_32_P46214	3.00E-03	2.84	solute carrier family 9 (sodium/hydrogen exchanger), member 9	Homo sapiens solute carrier family 9 (sodium/hydrogen exchanger), member 9 (SLC9A9), mRNA [NM_173653]
FAM117B	A_32_P195401	3.00E-03	2.99	family with sequence similarity 117, member B	Homo sapiens family with sequence similarity 117, member B (FAM117B), mRNA [NM_173511]
GPR160	A_23_P167005	3.01E-03	2.68	G protein-coupled receptor 160	Homo sapiens G protein-coupled

					receptor 160 (GPR160), mRNA [NM_014373]
ERMN	A_23_P102017	3.07E-03	4.17	ermin, ERM-like protein	Homo sapiens ermin, ERM-like protein (ERMN), transcript variant 2, mRNA [NM_020711]
SIGLEC10	A_23_P208182	3.08E-03	9.70	sialic acid binding Ig-like lectin 10	Homo sapiens sialic acid binding Ig-like lectin 10 (SIGLEC10), transcript variant 1, mRNA [NM_033130]
WDR54	A_23_P68072	3.12E-03	2.65	WD repeat domain 54	Homo sapiens WD repeat domain 54 (WDR54), mRNA [NM_032118]
H2AFZ	A_24_P41570	3.12E-03	-2.08	H2A histone family, member Z	Homo sapiens H2A histone family, member Z (H2AFZ), mRNA [NM_002106]
WDFY4	A_32_P30905	3.13E-03	7.47	WDFY family member 4	Homo sapiens WDFY family member 4 (WDFY4), mRNA [NM_020945]
PTK2B	A_33_P3351536	3.13E-03	3.35	PTK2B protein tyrosine kinase 2 beta	Homo sapiens PTK2B protein tyrosine kinase 2 beta (PTK2B), transcript variant 1, mRNA [NM_173174]
ATXN7L1	A_32_P179740	3.15E-03	2.35	ataxin 7-like 1	Homo sapiens ataxin 7-like 1 (ATXN7L1), transcript variant 1, mRNA [NM_020725]
TRIM59	A_32_P72341	3.16E-03	4.76	tripartite motif containing 59	Homo sapiens tripartite motif containing 59 (TRIM59), mRNA [NM_173084]
CYSLTR1	A_23_P22660	3.23E-03	2.78	cysteinyl leukotriene receptor 1	Homo sapiens cysteinyl leukotriene receptor 1 (CYSLTR1), mRNA [NM_006639]
NCR3	A_23_P251881	3.25E-03	12.32	natural cytotoxicity triggering receptor 3	Homo sapiens natural cytotoxicity triggering receptor 3 (NCR3), transcript variant 1, mRNA [NM_147130]
BTN3A1	A_33_P3388466	3.27E-03	3.96	butyrophilin, subfamily 3, member A1	Homo sapiens butyrophilin, subfamily 3, member A1 (BTN3A1), transcript variant 1, mRNA [NM_007048]
ARAP2	A_32_P83784	3.28E-03	4.85	ArfGAP with RhoGAP domain, ankyrin repeat and PH domain 2	Homo sapiens ArfGAP with RhoGAP domain, ankyrin repeat and PH domain 2 (ARAP2), mRNA [NM_015230]

CSF1R	A_23_P110791	3.28E-03	4.70	colony stimulating factor 1 receptor	Homo sapiens colony stimulating factor 1 receptor (CSF1R), mRNA [NM_005211]
LY75	A_23_P334173	3.35E-03	7.26	lymphocyte antigen 75	Homo sapiens lymphocyte antigen 75 (LY75), mRNA [NM_002349]
SPEF2	A_33_P3407400	3.35E-03	4.95	sperm flagellar 2	Homo sapiens sperm flagellar 2 (SPEF2), transcript variant 2, mRNA [NM_144722]
CD79A	A_23_P107735	3.38E-03	-4.04	CD79a molecule, immunoglobulin-associated alpha	Homo sapiens CD79a molecule, immunoglobulin-associated alpha (CD79A), transcript variant 1, mRNA [NM_001783]
CD59	A_24_P784765	3.38E-03	-2.38	CD59 molecule, complement regulatory protein	Homo sapiens CD59 molecule, complement regulatory protein (CD59), transcript variant 1, mRNA [NM_203330]
GNMT	A_23_P7957	3.40E-03	-6.18	glycine N-methyltransferase	Homo sapiens glycine N-methyltransferase (GNMT), mRNA [NM_018960]
SRCIN1	A_32_P201530	3.40E-03	5.01	SRC kinase signaling inhibitor 1	Homo sapiens SRC kinase signaling inhibitor 1 (SRCIN1), mRNA [NM_025248]
SLC9B1	A_23_P415611	3.40E-03	4.12	solute carrier family 9, subfamily B (cation proton antiporter 2), member 1	Homo sapiens solute carrier family 9, subfamily B (cation proton antiporter 2), member 1 (SLC9B1), nuclear gene encoding mitochondrial protein, transcript variant 1, mRNA [NM_139173]
MET	A_23_P359245	3.40E-03	-2.49	met proto-oncogene (hepatocyte growth factor receptor)	Homo sapiens met proto-oncogene (hepatocyte growth factor receptor) (MET), transcript variant 2, mRNA [NM_000245]
C17orf87	A_33_P3379268	3.42E-03	5.19	chromosome 17 open reading frame 87	Homo sapiens chromosome 17 open reading frame 87 (C17orf87), mRNA [NM_207103]
GTPBP6	A_24_P358381	3.43E-03	-2.21	GTP binding protein 6 (putative)	Homo sapiens GTP binding protein 6 (putative) (GTPBP6), mRNA

					[NM_012227]
LOC100128670	A_33_P3366754	3.46E-03	9.75	uncharacterized LOC100128670	Homo sapiens cDNA FLJ44998 fis, clone BRAWH3010833. [AK126945]
E2F8	A_23_P35871	3.46E-03	-5.34	E2F transcription factor 8	Homo sapiens E2F transcription factor 8 (E2F8), mRNA [NM_024680]
CCL28	A_23_P503072	3.46E-03	4.51	chemokine (C-C motif) ligand 28	Homo sapiens chemokine (C-C motif) ligand 28 (CCL28), mRNA [NM_148672]
ANO9	A_24_P8109	3.46E-03	3.44	anoctamin 9	Homo sapiens anoctamin 9 (ANO9), mRNA [NM_001012302]
OSBPL7	A_33_P3358740	3.46E-03	2.34	oxysterol binding protein-like 7	Homo sapiens oxysterol binding protein-like 7 (OSBPL7), transcript variant 1, mRNA [NM_145798]
LINC00239	A_32_P176911	3.47E-03	7.26	long intergenic non-protein coding RNA 239	Homo sapiens long intergenic non-protein coding RNA 239 (LINC00239), non-coding RNA [NR_026774]
IRF4	A_23_P214360	3.49E-03	5.96	interferon regulatory factor 4	Homo sapiens interferon regulatory factor 4 (IRF4), transcript variant 1, mRNA [NM_002460]
NCK2	A_33_P3268564	3.49E-03	3.01	NCK adaptor protein 2	Homo sapiens NCK adaptor protein 2 (NCK2), transcript variant 3, mRNA [NM_001004722]
C12orf35	A_23_P98930	3.49E-03	2.53	chromosome 12 open reading frame 35	Homo sapiens chromosome 12 open reading frame 35 (C12orf35), mRNA [NM_018169]
PIK3R5	A_23_P66543	3.50E-03	2.70	phosphoinositide-3-kinase, regulatory subunit 5	Homo sapiens phosphoinositide-3-kinase, regulatory subunit 5 (PIK3R5), transcript variant 2, mRNA [NM_014308]
LCP1	A_23_P204847	3.51E-03	7.53	lymphocyte cytosolic protein 1 (L-plastin)	Homo sapiens lymphocyte cytosolic protein 1 (L-plastin) (LCP1), mRNA [NM_002298]
SLC5A10	A_33_P3210418	3.52E-03	-2.08	solute carrier family 5 (sodium/glucose cotransporter), member 10	Homo sapiens solute carrier family 5 (sodium/glucose cotransporter), member 10 (SLC5A10), transcript variant

					1, mRNA [NM_152351]
NPPA	A_33_P3323699	3.56E-03	-2.12	natriuretic peptide A	natriuretic peptide A [Source:HGNC Symbol;Acc:7939] [ENST00000376476]
GPC6	A_23_P371682	3.56E-03	4.05	glypican 6	Homo sapiens glypican 6 (GPC6), mRNA [NM_005708]
PSD4	A_23_P68121	3.61E-03	4.38	pleckstrin and Sec7 domain containing 4	Homo sapiens pleckstrin and Sec7 domain containing 4 (PSD4), mRNA [NM_012455]
GPR126	A_24_P411749	3.61E-03	3.73	G protein-coupled receptor 126	Homo sapiens G protein-coupled receptor 126 (GPR126), transcript variant a1, mRNA [NM_020455]
LOC100506047	A_19_P00320407	3.66E-03	3.10		PREDICTED: Homo sapiens hypothetical LOC100506047 (LOC100506047), miscRNA [XR_109976]
C12orf75	A_32_P163089	3.69E-03	4.71	chromosome 12 open reading frame 75	Homo sapiens chromosome 12 open reading frame 75 (C12orf75), mRNA [NM_001145199]
DLEU1	A_23_P151337	3.80E-03	-2.01	deleted in lymphocytic leukemia 1 (non-protein coding)	Homo sapiens deleted in lymphocytic leukemia 1 (non-protein coding) (DLEU1), non-coding RNA [NR_002605]
MS4A4E	A_33_P3402269	3.82E-03	4.57	membrane-spanning 4-domains, subfamily A, member 4E	PREDICTED: Homo sapiens membrane-spanning 4-domains, subfamily A, member 4E (MS4A4E), mRNA [XM_003119183]
CCDC65	A_23_P47904	3.82E-03	3.14	coiled-coil domain containing 65	Homo sapiens coiled-coil domain containing 65 (CCDC65), mRNA [NM_033124]
CTLA4	A_33_P3396139	3.83E-03	10.68	cytotoxic T-lymphocyte-associated protein 4	Homo sapiens cytotoxic T-lymphocyte-associated protein 4 (CTLA4), transcript variant 1, mRNA [NM_005214]
FAM65B	A_24_P941359	3.83E-03	4.27	family with sequence similarity 65, member B	Homo sapiens family with sequence similarity 65, member B (FAM65B), transcript variant 1, mRNA [NM_014722]

PARVG	A_23_P250413	3.85E-03	5.10	parvin, gamma	Homo sapiens parvin, gamma (PARVG), transcript variant 1, mRNA [NM_022141]
ITGAD	A_23_P129665	3.86E-03	13.92	integrin, alpha D	Homo sapiens integrin, alpha D (ITGAD), mRNA [NM_005353]
LIG1	A_23_P39116	3.86E-03	2.20	ligase I, DNA, ATP-dependent	Homo sapiens ligase I, DNA, ATP-dependent (LIG1), mRNA [NM_000234]
UTP11L	A_23_P11774	3.86E-03	-2.03	UTP11-like, U3 small nucleolar ribonucleoprotein, (yeast)	Homo sapiens UTP11-like, U3 small nucleolar ribonucleoprotein, (yeast) (UTP11L), mRNA [NM_016037]
BHLHE22	A_32_P75581	3.86E-03	7.48	basic helix-loop-helix family, member e22	Homo sapiens basic helix-loop-helix family, member e22 (BHLHE22), mRNA [NM_152414]
PLD4	A_23_P88222	3.94E-03	2.85	phospholipase D family, member 4	Homo sapiens phospholipase D family, member 4 (PLD4), mRNA [NM_138790]
LOC389602	A_19_P00317444	4.10E-03	-3.02	uncharacterized LOC389602	PREDICTED: Homo sapiens hypothetical LOC389602 (LOC389602), miscRNA [XR_108709]
ZNF226	A_23_P395555	4.11E-03	2.05	zinc finger protein 226	Homo sapiens zinc finger protein 226 (ZNF226), transcript variant 1, mRNA [NM_001032372]
DPT	A_23_P200741	4.12E-03	3.50	dermatopontin	Homo sapiens dermatopontin (DPT), mRNA [NM_001937]
DAPP1	A_23_P255444	4.13E-03	7.74	dual adaptor of phosphotyrosine and 3-phosphoinositides	Homo sapiens dual adaptor of phosphotyrosine and 3-phosphoinositides (DAPP1), mRNA [NM_014395]
APOBEC3D	A_23_P132316	4.16E-03	3.51	apolipoprotein B mRNA editing enzyme, catalytic polypeptide-like 3D	Homo sapiens apolipoprotein B mRNA editing enzyme, catalytic polypeptide-like 3D (APOBEC3D), mRNA [NM_152426]
SCGB1D1	A_23_P127781	4.17E-03	-8.22	secretoglobin, family 1D, member 1	Homo sapiens secretoglobin, family 1D, member 1 (SCGB1D1), mRNA [NM_006552]

GTSF1	A_33_P3388192	4.17E-03	6.75	gametocyte specific factor 1	Homo sapiens gametocyte specific factor 1 (GTSF1), mRNA [NM_144594]
LST1	A_24_P103469	4.17E-03	4.52	leukocyte specific transcript 1	Homo sapiens leukocyte specific transcript 1 (LST1), transcript variant 1, mRNA [NM_007161]
FAM160A1	A_33_P3381318	4.17E-03	-3.72	family with sequence similarity 160, member A1	Homo sapiens family with sequence similarity 160, member A1 (FAM160A1), mRNA [NM_001109977]
ARRDC5	A_32_P30271	4.18E-03	5.65	arrestin domain containing 5	Homo sapiens arrestin domain containing 5 (ARRDC5), mRNA [NM_001080523]
FUT4	A_33_P3286536	4.21E-03	2.24	fucosyltransferase 4 (alpha (1,3) fucosyltransferase, myeloid-specific)	Homo sapiens fucosyltransferase 4 (alpha (1,3) fucosyltransferase, myeloid-specific) (FUT4), mRNA [NM_002033]
LINC00158	A_23_P132057	4.26E-03	3.16	long intergenic non-protein coding RNA 158	Homo sapiens long intergenic non-protein coding RNA 158 (LINC00158), non-coding RNA [NR_024027]
BMP7	A_23_P68487	4.33E-03	-15.87	bone morphogenetic protein 7	Homo sapiens bone morphogenetic protein 7 (BMP7), mRNA [NM_001719]
CEBPE	A_33_P3387991	4.33E-03	3.87	CCAAT/enhancer binding protein (C/EBP), epsilon	Homo sapiens CCAAT/enhancer binding protein (C/EBP), epsilon (CEBPE), mRNA [NM_001805]
EAF2	A_23_P252201	4.34E-03	2.68	ELL associated factor 2	Homo sapiens ELL associated factor 2 (EAF2), mRNA [NM_018456]
RAB30	A_24_P167338	4.34E-03	2.04	RAB30, member RAS oncogene family	Homo sapiens RAB30, member RAS oncogene family (RAB30), mRNA [NM_014488]
NAAA	A_33_P3297217	4.34E-03	2.14	N-acylethanolamine acid amidase	Homo sapiens N-acylethanolamine acid amidase (NAAA), transcript variant 2, mRNA [NM_001042402]
C20orf85	A_23_P314835	4.37E-03	-2.17	chromosome 20 open reading frame 85	Homo sapiens chromosome 20 open reading frame 85 (C20orf85), mRNA [NM_178456]

C5orf20	A_23_P81441	4.39E-03	11.06	chromosome 5 open reading frame 20	Homo sapiens chromosome 5 open reading frame 20 (C5orf20), mRNA [NM_130848]
ADORA3	A_23_P137931	4.42E-03	6.42	adenosine A3 receptor	Homo sapiens adenosine A3 receptor (ADORA3), transcript variant 2, mRNA [NM_000677]
FER1L4	A_23_P80048	4.44E-03	9.84	fer-1-like 4 (C. elegans) pseudogene	Homo sapiens fer-1-like 4 (C. elegans) pseudogene (FER1L4), non-coding RNA [NR_024377]
TIGIT	A_33_P3235213	4.44E-03	5.92	T cell immunoreceptor with Ig and ITIM domains	Homo sapiens T cell immunoreceptor with Ig and ITIM domains (TIGIT), mRNA [NM_173799]
NHLRC3	A_32_P129950	4.47E-03	2.03	NHL repeat containing 3	Homo sapiens NHL repeat containing 3 (NHLRC3), transcript variant 1, mRNA [NM_001012754]
LOC283710	A_33_P3336587	4.48E-03	9.71	uncharacterized LOC283710	Homo sapiens uncharacterized LOC283710 (LOC283710), mRNA [NM_001243538]
CXorf21	A_23_P62227	4.48E-03	5.02	chromosome X open reading frame 21	Homo sapiens chromosome X open reading frame 21 (CXorf21), mRNA [NM_025159]
GCNT1	A_33_P3282489	4.48E-03	3.98	glucosaminyl (N-acetyl) transferase 1, core 2	Homo sapiens glucosaminyl (N-acetyl) transferase 1, core 2 (GCNT1), transcript variant 1, mRNA [NM_001097634]
CDCA2	A_23_P385861	4.48E-03	-7.81	cell division cycle associated 2	Homo sapiens cell division cycle associated 2 (CDCA2), mRNA [NM_152562]
FAM72D	A_32_P151800	4.48E-03	5.50	family with sequence similarity 72, member D	Homo sapiens family with sequence similarity 72, member D (FAM72D), mRNA [NM_207418]
OLFML1	A_23_P147665	4.52E-03	2.35	olfactomedin-like 1	Homo sapiens olfactomedin-like 1 (OLFML1), mRNA [NM_198474]
QPCT	A_24_P71468	4.54E-03	4.32	glutaminyl-peptide	Homo sapiens glutaminyl-peptide

				cyclotransferase	cyclotransferase (QPCT), mRNA [NM_012413]
PPYR1	A_24_P364042	4.54E-03	-3.92	pancreatic polypeptide receptor 1	Homo sapiens pancreatic polypeptide receptor 1 (PPYR1), mRNA [NM_005972]
KCNJ15	A_33_P3267532	4.56E-03	-2.43	potassium inwardly-rectifying channel, subfamily J, member 15	Homo sapiens potassium inwardly-rectifying channel, subfamily J, member 15 (KCNJ15), transcript variant 1, mRNA [NM_170736]
NRK	A_33_P3248405	4.58E-03	10.13	Nik related kinase	Nik related kinase [Source:HGNC Symbol;Acc:25391] [ENST00000536164]
NUP98	A_23_P308032	4.59E-03	-2.18	nucleoporin 98kDa	Homo sapiens nucleoporin 98kDa (NUP98), transcript variant 3, mRNA [NM_005387]
RAB27B	A_23_P107612	4.62E-03	2.96	RAB27B, member RAS oncogene family	Homo sapiens RAB27B, member RAS oncogene family (RAB27B), mRNA [NM_004163]
PIM3	A_23_P61398	4.62E-03	-2.46	pim-3 oncogene	Homo sapiens pim-3 oncogene (PIM3), mRNA [NM_001001852]
KNTC1	A_23_P136817	4.63E-03	2.35	kinetochore associated 1	Homo sapiens kinetochore associated 1 (KNTC1), mRNA [NM_014708]
TSPAN5	A_33_P3325349	4.63E-03	3.48	tetraspanin 5	Homo sapiens tetraspanin 5 (TSPAN5), mRNA [NM_005723]
RHOBTB1	A_33_P3348288	4.64E-03	2.02	Rho-related BTB domain containing 1	Homo sapiens Rho-related BTB domain containing 1 (RHOBTB1), transcript variant 4, mRNA [NM_001242359]
EDN2	A_23_P312150	4.71E-03	-8.18	endothelin 2	Homo sapiens endothelin 2 (EDN2), mRNA [NM_001956]
KRTAP4-12	A_23_P15798	4.71E-03	-2.55	keratin associated protein 4-12	Homo sapiens keratin associated protein 4-12 (KRTAP4-12), mRNA [NM_031854]
WIPF1	A_23_P330616	4.75E-03	2.81	WAS/WASL interacting protein family, member 1	Homo sapiens WAS/WASL interacting protein family, member 1 (WIPF1), transcript variant 2, mRNA [NM_001077269]

ARHGAP25	A_23_P142974	4.75E-03	2.81	Rho GTPase activating protein 25	Homo sapiens Rho GTPase activating protein 25 (ARHGAP25), transcript variant 1, mRNA [NM_001007231]
PTDSS1	A_23_P168868	4.76E-03	-2.22	phosphatidylserine synthase 1	Homo sapiens phosphatidylserine synthase 1 (PTDSS1), mRNA [NM_014754]
CDKN2A	A_33_P3411628	4.77E-03	4.66	cyclin-dependent kinase inhibitor 2A (melanoma, p16, inhibits CDK4)	Homo sapiens cyclin-dependent kinase inhibitor 2A (melanoma, p16, inhibits CDK4) (CDKN2A), transcript variant 1, mRNA [NM_000077]
GRAMD1C	A_23_P253012	4.79E-03	4.17	GRAM domain containing 1C	Homo sapiens GRAM domain containing 1C (GRAMD1C), transcript variant 1, mRNA [NM_017577]
FAM133A	A_23_P345808	4.80E-03	4.78	family with sequence similarity 133, member A	Homo sapiens family with sequence similarity 133, member A (FAM133A), transcript variant 2, mRNA [NM_173698]
RIMKLA	A_23_P349406	4.80E-03	-2.22	ribosomal modification protein rimK-like family member A	Homo sapiens ribosomal modification protein rimK-like family member A (RIMKLA), mRNA [NM_173642]
C16orf47	A_33_P3417840	4.92E-03	2.11	chromosome 16 open reading frame 47	Homo sapiens cDNA FLJ26184 fis, clone ADG04431. [AK129695]
PENK	A_23_P417918	4.94E-03	10.01	proenkephalin	Homo sapiens proenkephalin (PENK), transcript variant 2, mRNA [NM_006211]
TMEM40	A_33_P3391418	4.94E-03	-2.26	transmembrane protein 40	Homo sapiens transmembrane protein 40 (TMEM40), mRNA [NM_018306]
RGS9BP	A_32_P227605	4.99E-03	-3.03	regulator of G protein signaling 9 binding protein	Homo sapiens regulator of G protein signaling 9 binding protein (RGS9BP), mRNA [NM_207391]
CA14	A_23_P63379	5.10E-03	-7.22	carbonic anhydrase XIV	Homo sapiens carbonic anhydrase XIV (CA14), mRNA [NM_012113]
SLC16A9	A_23_P115726	5.10E-03	5.33	solute carrier family 16, member 9 (monocarboxylic acid transporter 9)	Homo sapiens solute carrier family 16, member 9 (monocarboxylic acid transporter 9) (SLC16A9), mRNA

					[NM_194298]
GNLY	A_23_P209954	5.12E-03	6.94	granulysin	Homo sapiens granulysin (GNLY), transcript variant NKG5, mRNA [NM_006433]
TXNIP	A_23_P97700	5.12E-03	2.52	thioredoxin interacting protein	Homo sapiens thioredoxin interacting protein (TXNIP), mRNA [NM_006472]
ZGLP1	A_33_P3417452	5.18E-03	2.49	zinc finger, GATA-like protein 1	Homo sapiens zinc finger, GATA-like protein 1 (ZGLP1), mRNA [NM_001103167]
OTUD7A	A_33_P3262376	5.19E-03	-2.94	OTU domain containing 7A	Homo sapiens OTU domain containing 7A (OTUD7A), mRNA [NM_130901]
RTN4RL2	A_32_P528311	5.20E-03	-2.36	reticulon 4 receptor-like 2	Homo sapiens reticulon 4 receptor-like 2 (RTN4RL2), mRNA [NM_178570]
HLA-DPB1	A_24_P166443	5.24E-03	4.94	major histocompatibility complex, class II, DP beta 1	Homo sapiens major histocompatibility complex, class II, DP beta 1 (HLA-DPB1), mRNA [NM_002121]
GMIP	A_23_P56228	5.25E-03	4.05	GEM interacting protein	Homo sapiens GEM interacting protein (GMIP), mRNA [NM_016573]
CLDN14	A_33_P3284036	5.25E-03	3.98	claudin 14	claudin 14 [Source:HGNC Symbol;Acc:2035] [ENST00000478313]
ADCK3	A_23_P85598	5.27E-03	-2.03	aarF domain containing kinase 3	Homo sapiens aarF domain containing kinase 3 (ADCK3), nuclear gene encoding mitochondrial protein, mRNA [NM_020247]
HKDC1	A_23_P202427	5.28E-03	3.34	hexokinase domain containing 1	Homo sapiens hexokinase domain containing 1 (HKDC1), mRNA [NM_025130]
PLA2G4F	A_24_P147169	5.28E-03	-2.71	phospholipase A2, group IVF	Homo sapiens phospholipase A2, group IVF (PLA2G4F), transcript variant 1, mRNA [NM_213600]
ZFP36L2	A_23_P101960	5.30E-03	2.34	zinc finger protein 36, C3H type-like 2	Homo sapiens zinc finger protein 36, C3H type-like 2 (ZFP36L2), mRNA [NM_006887]

LPXN	A_23_P87150	5.32E-03	2.66	leupaxin	Homo sapiens leupaxin (LPXN), transcript variant 2, mRNA [NM_004811]
CPNE7	A_33_P3262191	5.34E-03	6.06	copine VII	Homo sapiens copine VII (CPNE7), transcript variant 2, mRNA [NM_014427]
LILRB1	A_33_P3231414	5.34E-03	6.06	leukocyte immunoglobulin-like receptor, subfamily B (with TM and ITIM domains), member 1	Homo sapiens leukocyte immunoglobulin-like receptor, subfamily B (with TM and ITIM domains), member 1 (LILRB1), transcript variant 1, mRNA [NM_006669]
TRIM11	A_24_P50908	5.36E-03	2.29	tripartite motif containing 11	tripartite motif containing 11 [Source:HGNC Symbol;Acc:16281] [ENST00000366699]
GPR174	A_23_P11070	5.37E-03	4.59	G protein-coupled receptor 174	Homo sapiens G protein-coupled receptor 174 (GPR174), mRNA [NM_032553]
ADAM28	A_33_P3380652	5.37E-03	4.07	ADAM metallopeptidase domain 28	Homo sapiens ADAM metallopeptidase domain 28 (ADAM28), transcript variant 3, mRNA [NM_021777]
MARVELD2	A_23_P401675	5.37E-03	-2.64	MARVEL domain containing 2	MARVEL domain containing 2 [Source:HGNC Symbol;Acc:26401] [ENST00000454295]
HLA-A	A_33_P3237567	5.37E-03	2.18	major histocompatibility complex, class I, A	major histocompatibility complex, class I, A [Source:HGNC Symbol;Acc:4931] [ENST00000464610]
CHAC2	A_32_P194264	5.37E-03	-2.01	ChaC, cation transport regulator homolog 2 (E. coli)	Homo sapiens ChaC, cation transport regulator homolog 2 (E. coli) (CHAC2), mRNA [NM_001008708]
C1orf186	A_23_P95640	5.38E-03	5.86	chromosome 1 open reading frame 186	Homo sapiens chromosome 1 open reading frame 186 (C1orf186), mRNA [NM_001007544]
AIM1L	A_33_P3327642	5.39E-03	3.71	absent in melanoma 1-like	Homo sapiens absent in melanoma 1-like (AIM1L), mRNA [NM_001039775]
HLA-DOA	A_32_P356316	5.44E-03	5.60	major histocompatibility	Homo sapiens major histocompatibility

				complex, class II, DO alpha	complex, class II, DO alpha (HLA-DOA), mRNA [NM_002119]
FLJ31104	A_33_P3695899	5.47E-03	3.27	uncharacterized LOC441072	PREDICTED: Homo sapiens hypothetical LOC441072 (FLJ31104), miscRNA [XR_108600]
MYO1G	A_24_P206343	5.49E-03	7.73	myosin IG	Homo sapiens myosin IG (MYO1G), mRNA [NM_033054]
MCTP2	A_23_P65789	5.50E-03	2.21	multiple C2 domains, transmembrane 2	Homo sapiens multiple C2 domains, transmembrane 2 (MCTP2), transcript variant 1, mRNA [NM_018349]
AIG1	A_33_P3416568	5.50E-03	-2.11	androgen-induced 1	androgen-induced 1 [Source:HGNC Symbol;Acc:21607] [ENST00000367596]
FAM49A	A_23_P21560	5.50E-03	2.11	family with sequence similarity 49, member A	Homo sapiens family with sequence similarity 49, member A (FAM49A), mRNA [NM_030797]
KLHDC7B	A_23_P6535	5.52E-03	8.19	kelch domain containing 7B	Homo sapiens kelch domain containing 7B (KLHDC7B), mRNA [NM_138433]
ZSWIM5	A_23_P383118	5.52E-03	2.67	zinc finger, SWIM-type containing 5	Homo sapiens zinc finger, SWIM-type containing 5 (ZSWIM5), mRNA [NM_020883]
KIR2DS2	A_23_P130815	5.53E-03	7.11	killer cell immunoglobulin-like receptor, two domains, short cytoplasmic tail, 2	Homo sapiens killer cell immunoglobulin-like receptor, two domains, short cytoplasmic tail, 2 (KIR2DS2), mRNA [NM_012312]
TNFSF13B	A_23_P14174	5.53E-03	5.40	tumor necrosis factor (ligand) superfamily, member 13b	Homo sapiens tumor necrosis factor (ligand) superfamily, member 13b (TNFSF13B), transcript variant 1, mRNA [NM_006573]
ANKRD36B	A_33_P3261648	5.53E-03	3.54	ankyrin repeat domain 36B	Homo sapiens ankyrin repeat domain 36B (ANKRD36B), mRNA [NM_025190]
SRGAP2P2	A_33_P3372705	5.54E-03	2.26	SLIT-ROBO Rho GTPase activating protein 2 pseudogene 2	Homo sapiens SLIT-ROBO Rho GTPase activating protein 2 pseudogene 2 (SRGAP2P2), non-coding RNA

					[NR_034178]
ASPN	A_33_P3216059	5.56E-03	5.01	asporin	Homo sapiens asporin (ASPN), transcript variant 2, mRNA [NM_001193335]
CLEC10A	A_23_P141505	5.57E-03	5.23	C-type lectin domain family 10, member A	Homo sapiens C-type lectin domain family 10, member A (CLEC10A), transcript variant 1, mRNA [NM_182906]
CPXM2	A_33_P3333158	5.57E-03	5.10	carboxypeptidase X (M14 family), member 2	Homo sapiens carboxypeptidase X (M14 family), member 2 (CPXM2), mRNA [NM_198148]
CD300LF	A_23_P55020	5.57E-03	4.95	CD300 molecule-like family member f	Homo sapiens CD300 molecule-like family member f (CD300LF), mRNA [NM_139018]
LOC728392	A_33_P3331491	5.62E-03	2.26	uncharacterized LOC728392	Homo sapiens uncharacterized protein LOC728392 (LOC728392), mRNA [NM_001162371]
MYO19	A_23_P100868	5.63E-03	-2.48	myosin XIX	Homo sapiens myosin XIX (MYO19), transcript variant 3, mRNA [NM_001033580]
PCNXL2	A_23_P200260	5.69E-03	4.77	pecanex-like 2 (Drosophila)	Homo sapiens pecanex-like 2 (Drosophila) (PCNXL2), transcript variant 1, mRNA [NM_014801]
SEMA6B	A_33_P3263651	5.74E-03	-2.23	sema domain, transmembrane domain (TM), and cytoplasmic domain, (semaphorin) 6B	Homo sapiens sema domain, transmembrane domain (TM), and cytoplasmic domain, (semaphorin) 6B (SEMA6B), mRNA [NM_032108]
RASGRP2	A_33_P3209962	5.79E-03	-2.38	RAS guanyl releasing protein 2 (calcium and DAG-regulated)	Homo sapiens RAS guanyl releasing protein 2 (calcium and DAG-regulated) (RASGRP2), transcript variant 2, mRNA [NM_153819]
SLC20A2	A_33_P3386835	5.83E-03	-2.02	solute carrier family 20 (phosphate transporter), member 2	solute carrier family 20 (phosphate transporter), member 2 [Source:HGNC Symbol;Acc:10947] [ENST00000523340]
BTN3A2	A_33_P3227880	5.87E-03	4.60	butyrophilin, subfamily 3,	Homo sapiens butyrophilin, subfamily 3,

				member A2	member A2 (BTN3A2), transcript variant 3, mRNA [NM_001197247]
HDAC10	A_23_P368740	5.88E-03	2.93	histone deacetylase 10	Homo sapiens histone deacetylase 10 (HDAC10), transcript variant 1, mRNA [NM_032019]
PTPRVP	A_33_P3309481	5.95E-03	4.47	protein tyrosine phosphatase, receptor type, V, pseudogene	Homo sapiens protein tyrosine phosphatase, receptor type, V, pseudogene (PTPRVP), non-coding RNA [NR_002930]
RNASET2	A_24_P360269	5.96E-03	2.72	ribonuclease T2	Homo sapiens ribonuclease T2 (RNASET2), mRNA [NM_003730]
TNFSF14	A_24_P237036	6.00E-03	8.94	tumor necrosis factor (ligand) superfamily, member 14	Homo sapiens tumor necrosis factor (ligand) superfamily, member 14 (TNFSF14), transcript variant 1, mRNA [NM_003807]
HELB	A_23_P2294	6.00E-03	3.76	helicase (DNA) B	Homo sapiens helicase (DNA) B (HELB), mRNA [NM_033647]
SFI1	A_23_P80278	6.04E-03	2.36	Sfi1 homolog, spindle assembly associated (yeast)	Homo sapiens Sfi1 homolog, spindle assembly associated (yeast) (SFI1), transcript variant 1, mRNA [NM_001007467]
LEF1	A_24_P20630	6.05E-03	9.16	lymphoid enhancer-binding factor 1	Homo sapiens lymphoid enhancer-binding factor 1 (LEF1), transcript variant 1, mRNA [NM_016269]
MREG	A_23_P108948	6.05E-03	5.06	melanoregulin	Homo sapiens melanoregulin (MREG), mRNA [NM_018000]
VAV3	A_23_P201551	6.05E-03	2.64	vav 3 guanine nucleotide exchange factor	Homo sapiens vav 3 guanine nucleotide exchange factor (VAV3), transcript variant 1, mRNA [NM_006113]
GTF2IRD1	A_23_P111621	6.05E-03	-2.16	GTF2I repeat domain containing 1	Homo sapiens GTF2I repeat domain containing 1 (GTF2IRD1), transcript variant 2, mRNA [NM_005685]
sept-11	A_33_P3310475	6.05E-03	2.12	septin 11	Homo sapiens septin 11 (SEPT11), mRNA

					[NM_018243]
AURKA	A_23_P131866	6.07E-03	-2.19	aurora kinase A	Homo sapiens aurora kinase A (AURKA), transcript variant 1, mRNA [NM_198433]
COL16A1	A_23_P160318	6.09E-03	3.28	collagen, type XVI, alpha 1	Homo sapiens collagen, type XVI, alpha 1 (COL16A1), mRNA [NM_001856]
GPR34	A_23_P11201	6.12E-03	5.36	G protein-coupled receptor 34	Homo sapiens G protein-coupled receptor 34 (GPR34), transcript variant 4, mRNA [NM_001097579]
LMF2	A_33_P3408632	6.14E-03	-2.23	lipase maturation factor 2	Homo sapiens lipase maturation factor 2 (LMF2), mRNA [NM_033200]
LOC100506380	A_19_P00809902	6.22E-03	-3.24		PREDICTED: Homo sapiens hypothetical LOC100506380, transcript variant 2 (LOC100506380), miscRNA [XR_108713]
ZMYND15	A_23_P89570	6.24E-03	2.55	zinc finger, MYND-type containing 15	Homo sapiens zinc finger, MYND-type containing 15 (ZMYND15), transcript variant 2, mRNA [NM_032265]
C21orf96	A_24_P65941	6.28E-03	8.55	chromosome 21 open reading frame 96	Homo sapiens chromosome 21 open reading frame 96 (C21orf96), non-coding RNA [NR_026812]
FOXN2	A_32_P140898	6.32E-03	2.44	forkhead box N2	Homo sapiens forkhead box N2 (FOXN2), mRNA [NM_002158]
PCDH20	A_23_P204885	6.34E-03	-12.28	protocadherin 20	Homo sapiens protocadherin 20 (PCDH20), mRNA [NM_022843]
HEMK1	A_24_P268729	6.34E-03	2.80	HemK methyltransferase family member 1	Homo sapiens HemK methyltransferase family member 1 (HEMK1), mRNA [NM_016173]
LOC100128851	A_33_P3411885	6.35E-03	-2.37	uncharacterized LOC100128851	Homo sapiens cDNA FLJ45515 fis, clone BRTHA2022914. [AK127423]
BMF	A_23_P379649	6.39E-03	3.14	Bcl2 modifying factor	Homo sapiens Bcl2 modifying factor (BMF), transcript variant 1, mRNA [NM_001003940]
LPHN1	A_23_P391926	6.42E-03	-2.01	latrophilin 1	Homo sapiens latrophilin 1 (LPHN1), transcript variant 1, mRNA

					[NM_001008701]
FIBIN	A_33_P3325497	6.42E-03	4.81	fin bud initiation factor homolog (zebrafish)	Homo sapiens fin bud initiation factor homolog (zebrafish) (FIBIN), mRNA [NM_203371]
C9orf93	A_32_P395879	6.42E-03	3.58	chromosome 9 open reading frame 93	Homo sapiens chromosome 9 open reading frame 93 (C9orf93), mRNA [NM_173550]
EDEM1	A_24_P285768	6.42E-03	2.15	ER degradation enhancer, mannosidase alpha-like 1	Homo sapiens ER degradation enhancer, mannosidase alpha-like 1 (EDEM1), mRNA [NM_014674]
LOC100129516	A_32_P214284	6.43E-03	-2.42	uncharacterized LOC100129516	PREDICTED: Homo sapiens hypothetical protein LOC100129516 (LOC100129516), mRNA [XM_003119920]
PABPC1L	A_24_P92183	6.44E-03	3.93	poly(A) binding protein, cytoplasmic 1-like	Homo sapiens poly(A) binding protein, cytoplasmic 1-like (PABPC1L), mRNA [NM_001124756]
RHOD	A_23_P1523	6.45E-03	-2.92	ras homolog gene family, member D	Homo sapiens ras homolog gene family, member D (RHOD), mRNA [NM_014578]
ARHGAP15	A_23_P84154	6.50E-03	2.65	Rho GTPase activating protein 15	Homo sapiens Rho GTPase activating protein 15 (ARHGAP15), mRNA [NM_018460]
SELL	A_33_P3400273	6.50E-03	8.13	selectin L	Homo sapiens selectin L (SELL), transcript variant 1, mRNA [NM_000655]
FER1L5	A_33_P3299055	6.56E-03	-2.01	fer-1-like 5 (C. elegans)	Homo sapiens fer-1-like 5 (C. elegans) (FER1L5), mRNA [NM_001113382]
ARRDC2	A_24_P22976	6.59E-03	2.80	arrestin domain containing 2	Homo sapiens arrestin domain containing 2 (ARRDC2), transcript variant 2, mRNA [NM_001025604]
DDIT4	A_23_P104318	6.61E-03	4.02	DNA-damage-inducible transcript 4	Homo sapiens DNA-damage-inducible transcript 4 (DDIT4), mRNA [NM_019058]
HIST1H2AM	A_32_P221799	6.61E-03	3.41	histone cluster 1, H2am	Homo sapiens histone cluster 1, H2am (HIST1H2AM), mRNA [NM_003514]

ICOS	A_23_P371215	6.66E-03	5.01	inducible T-cell co-stimulator	Homo sapiens inducible T-cell co-stimulator (ICOS), mRNA [NM_012092]
VANGL2	A_33_P3282005	6.68E-03	-2.56	vang-like 2 (van gogh, Drosophila)	Homo sapiens vang-like 2 (van gogh, Drosophila) (VANGL2), mRNA [NM_020335]
ABCC3	A_23_P207507	6.76E-03	5.58	ATP-binding cassette, sub-family C (CFTR/MRP), member 3	Homo sapiens ATP-binding cassette, sub-family C (CFTR/MRP), member 3 (ABCC3), transcript variant 1, mRNA [NM_003786]
TNFRSF9	A_23_P51936	6.78E-03	2.34	tumor necrosis factor receptor superfamily, member 9	Homo sapiens tumor necrosis factor receptor superfamily, member 9 (TNFRSF9), mRNA [NM_001561]
APBA2	A_33_P3344504	6.82E-03	11.73	amyloid beta (A4) precursor protein-binding, family A, member 2	amyloid beta (A4) precursor protein-binding, family A, member 2 [Source:HGNC Symbol;Acc:579] [ENST00000382938]
KCNIP4	A_33_P3274194	6.84E-03	2.11	Kv channel interacting protein 4	Homo sapiens Kv channel interacting protein 4 (KCNIP4), transcript variant 5, mRNA [NM_001035003]
IFNG	A_23_P151294	6.86E-03	5.86	interferon, gamma	Homo sapiens interferon, gamma (IFNG), mRNA [NM_000619]
TLR8	A_23_P73837	6.97E-03	7.09	toll-like receptor 8	Homo sapiens toll-like receptor 8 (TLR8), mRNA [NM_138636]
LDLRAD2	A_33_P3260125	6.97E-03	6.63	low density lipoprotein receptor class A domain containing 2	Homo sapiens low density lipoprotein receptor class A domain containing 2 (LDLRAD2), mRNA [NM_001013693]
FAM159A	A_33_P3295786	6.97E-03	2.68	family with sequence similarity 159, member A	Homo sapiens family with sequence similarity 159, member A (FAM159A), mRNA [NM_001042693]
PDE4A	A_24_P322474	7.02E-03	-2.14	phosphodiesterase 4A, cAMP-specific	Homo sapiens phosphodiesterase 4A, cAMP-specific (PDE4A), transcript variant 4, mRNA [NM_006202]
AIDA	A_33_P3250857	7.02E-03	-2.08	axin interactor, dorsalization	axin interactor, dorsalization associated

				associated	[Source:HGNC Symbol;Acc:25761] [ENST00000474863]
PDE6A	A_23_P81590	7.08E-03	4.42	phosphodiesterase 6A, cGMP-specific, rod, alpha	Homo sapiens phosphodiesterase 6A, cGMP-specific, rod, alpha (PDE6A), mRNA [NM_000440]
NPFFR2	A_23_P155900	7.08E-03	6.33	neuropeptide FF receptor 2	Homo sapiens neuropeptide FF receptor 2 (NPFFR2), transcript variant 2, mRNA [NM_053036]
TP53INP1	A_23_P168882	7.08E-03	2.51	tumor protein p53 inducible nuclear protein 1	Homo sapiens tumor protein p53 inducible nuclear protein 1 (TP53INP1), transcript variant 1, mRNA [NM_033285]
S100A7A	A_33_P3393504	7.14E-03	-2.65	S100 calcium binding protein A7A	S100 calcium binding protein A7A [Source:HGNC Symbol;Acc:21657] [ENST00000329256]
CD72	A_23_P250245	7.17E-03	7.27	CD72 molecule	Homo sapiens CD72 molecule (CD72), mRNA [NM_001782]
H1FX-AS1	A_33_P3368771	7.18E-03	3.17	H1FX antisense RNA 1 (non-protein coding)	Homo sapiens H1FX antisense RNA 1 (non-protein coding) (H1FX-AS1), non-coding RNA [NR_026991]
ATP2A3	A_23_P207632	7.18E-03	3.07	ATPase, Ca++ transporting, ubiquitous	Homo sapiens ATPase, Ca++ transporting, ubiquitous (ATP2A3), transcript variant 5, mRNA [NM_174953]
APOL6	A_23_P155052	7.19E-03	2.73	apolipoprotein L, 6	Homo sapiens apolipoprotein L, 6 (APOL6), mRNA [NM_030641]
CA5B	A_33_P3316068	7.23E-03	2.35	carbonic anhydrase VB, mitochondrial	carbonic anhydrase VB, mitochondrial [Source:HGNC Symbol;Acc:1378] [ENST00000380313]
MPEG1	A_24_P153568	7.23E-03	5.88	macrophage expressed 1	Homo sapiens macrophage expressed 1 (MPEG1), mRNA [NM_001039396]
JPH1	A_33_P3364348	7.23E-03	-3.02	junctophilin 1	Homo sapiens junctophilin 1 (JPH1), mRNA [NM_020647]
PYCARD	A_23_P26629	7.23E-03	2.93	PYD and CARD domain containing	Homo sapiens PYD and CARD domain containing (PYCARD), transcript variant

					1, mRNA [NM_013258]
SLFN13	A_33_P3275487	7.23E-03	4.95	schlafen family member 13	Homo sapiens schlafen family member 13 (SLFN13), mRNA [NM_144682]
SHC4	A_32_P234145	7.31E-03	3.69	SHC (Src homology 2 domain containing) family, member 4	Homo sapiens SHC (Src homology 2 domain containing) family, member 4 (SHC4), mRNA [NM_203349]
SPHK1	A_33_P3222069	7.31E-03	-2.06	sphingosine kinase 1	Homo sapiens sphingosine kinase 1 (SPHK1), transcript variant 2, mRNA [NM_182965]
LOC100129596	A_33_P3364964	7.31E-03	-3.34	uncharacterized LOC100129596	PREDICTED: Homo sapiens hypothetical LOC100129596 (LOC100129596), miscRNA [XR_110585]
ANPEP	A_23_P88626	7.34E-03	4.51	alanyl (membrane) aminopeptidase	Homo sapiens alanyl (membrane) aminopeptidase (ANPEP), mRNA [NM_001150]
MEG3	A_19_P00316675	7.35E-03	3.03		Homo sapiens maternally expressed 3 (non-protein coding) (MEG3), transcript variant 5, non-coding RNA [NR_033359]
VAV1	A_23_P38959	7.36E-03	4.98	vav 1 guanine nucleotide exchange factor	Homo sapiens vav 1 guanine nucleotide exchange factor (VAV1), mRNA [NM_005428]
GPR132	A_23_P128808	7.40E-03	3.34	G protein-coupled receptor 132	Homo sapiens G protein-coupled receptor 132 (GPR132), mRNA [NM_013345]
SIGLECP3	A_32_P128258	7.43E-03	9.06	sialic acid binding Ig-like lectin, pseudogene 3	Homo sapiens sialic acid binding Ig-like lectin, pseudogene 3 (SIGLECP3), non-coding RNA [NR_002804]
FMO3	A_23_P155596	7.51E-03	4.15	flavin containing monooxygenase 3	Homo sapiens flavin containing monooxygenase 3 (FMO3), transcript variant 2, mRNA [NM_001002294]
SPATA24	A_24_P220897	7.51E-03	-2.72	spermatogenesis associated 24	Homo sapiens spermatogenesis associated 24 (SPATA24), mRNA [NM_194296]

PTPN18	A_23_P210015	7.57E-03	2.41	protein tyrosine phosphatase, non-receptor type 18 (brain-derived)	Homo sapiens protein tyrosine phosphatase, non-receptor type 18 (brain-derived) (PTPN18), transcript variant 1, mRNA [NM_014369]
ZNF217	A_23_P210608	7.59E-03	2.06	zinc finger protein 217	Homo sapiens zinc finger protein 217 (ZNF217), mRNA [NM_006526]
OR5F1	A_23_P127684	7.60E-03	-5.02	olfactory receptor, family 5, subfamily F, member 1	Homo sapiens olfactory receptor, family 5, subfamily F, member 1 (OR5F1), mRNA [NM_003697]
PVR	A_23_P141894	7.67E-03	-2.61	poliovirus receptor	Homo sapiens poliovirus receptor (PVR), transcript variant 1, mRNA [NM_006505]
PRLHR	A_33_P3370364	7.67E-03	-2.00	prolactin releasing hormone receptor	Homo sapiens prolactin releasing hormone receptor (PRLHR), mRNA [NM_004248]
CP	A_33_P3296587	7.74E-03	3.54	ceruloplasmin (ferroxidase)	Homo sapiens ceruloplasmin (ferroxidase) (CP), mRNA [NM_000096]
PTPN6	A_23_P162486	7.74E-03	3.53	protein tyrosine phosphatase, non-receptor type 6	Homo sapiens protein tyrosine phosphatase, non-receptor type 6 (PTPN6), transcript variant 1, mRNA [NM_002831]
ANGPTL1	A_23_P126706	7.77E-03	3.96	angiopoietin-like 1	Homo sapiens angiopoietin-like 1 (ANGPTL1), mRNA [NM_004673]
KIAA1407	A_23_P419213	7.83E-03	4.45	KIAA1407	Homo sapiens KIAA1407 (KIAA1407), mRNA [NM_020817]
LRRC38	A_23_P103877	7.85E-03	-9.11	leucine rich repeat containing 38	Homo sapiens leucine rich repeat containing 38 (LRRC38), mRNA [NM_001010847]
C4orf11	A_33_P3304060	7.85E-03	-4.60	chromosome 4 open reading frame 11	Homo sapiens chromosome 4 open reading frame 11 (C4orf11), transcript variant 1, non-coding RNA [NR_024087]
RAP1GAP2	A_33_P3333146	7.85E-03	-3.45	RAP1 GTPase activating protein 2	Homo sapiens RAP1 GTPase activating protein 2 (RAP1GAP2), transcript variant 1, mRNA [NM_015085]

VAC14	A_33_P3363091	7.85E-03	2.68	Vac14 homolog (S. cerevisiae)	Human Tax1 binding protein mRNA, partial cds. [U25801]
SPC24	A_24_P314571	7.86E-03	-2.28	SPC24, NDC80 kinetochore complex component, homolog (S. cerevisiae)	Homo sapiens cDNA FLJ90806 fis, clone Y79AA1000750. [AK075287]
ATP1A4	A_23_P160177	7.91E-03	-2.46	ATPase, Na+/K+ transporting, alpha 4 polypeptide	Homo sapiens ATPase, Na+/K+ transporting, alpha 4 polypeptide (ATP1A4), transcript variant 1, mRNA [NM_144699]
DUSP18	A_23_P143650	7.94E-03	2.07	dual specificity phosphatase 18	Homo sapiens dual specificity phosphatase 18 (DUSP18), mRNA [NM_152511]
ZBED2	A_23_P113793	7.99E-03	3.20	zinc finger, BED-type containing 2	Homo sapiens zinc finger, BED-type containing 2 (ZBED2), mRNA [NM_024508]
CYTL1	A_23_P10647	7.99E-03	2.40	cytokine-like 1	Homo sapiens cytokine-like 1 (CYTL1), mRNA [NM_018659]
LOC283050	A_33_P3394140	8.01E-03	2.43	uncharacterized LOC283050	Homo sapiens uncharacterized LOC283050 (LOC283050), transcript variant 1, non-coding RNA [NR_024431]
HLA-DPB2	A_24_P288836	8.02E-03	5.22	major histocompatibility complex, class II, DP beta 2 (pseudogene)	Homo sapiens major histocompatibility complex, class II, DP beta 2 (pseudogene) (HLA-DPB2), non-coding RNA [NR_001435]
FNDC5	A_23_P420326	8.08E-03	-2.56	fibronectin type III domain containing 5	Homo sapiens fibronectin type III domain containing 5 (FNDC5), transcript variant 2, mRNA [NM_153756]
MEX3D	A_23_P39453	8.08E-03	-2.45	mex-3 homolog D (C. elegans)	Homo sapiens mex-3 homolog D (C. elegans) (MEX3D), transcript variant 1, mRNA [NM_203304]
BEND6	A_33_P3329769	8.10E-03	4.83	BEN domain containing 6	Homo sapiens BEN domain containing 6 (BEND6), mRNA [NM_152731]
C15orf59	A_33_P3398917	8.10E-03	-2.32	chromosome 15 open reading	Homo sapiens chromosome 15 open

				frame 59	reading frame 59 (C15orf59), mRNA [NM_001039614]
GCET2	A_24_P182947	8.10E-03	4.00	germinal center expressed transcript 2	Homo sapiens germinal center expressed transcript 2 (GCET2), transcript variant 3, mRNA [NM_001190259]
ANKRD36BP2	A_24_P341089	8.10E-03	3.64	ankyrin repeat domain 36B pseudogene 2	Homo sapiens ankyrin repeat domain 36B pseudogene 2 (ANKRD36BP2), non-coding RNA [NR_015424]
SPRY3	A_23_P311010	8.10E-03	-2.23	sprouty homolog 3 (Drosophila)	Homo sapiens sprouty homolog 3 (Drosophila) (SPRY3), mRNA [NM_005840]
MFAP4	A_23_P164057	8.12E-03	3.06	microfibrillar-associated protein 4	Homo sapiens microfibrillar-associated protein 4 (MFAP4), transcript variant 2, mRNA [NM_002404]
ARHGAP4	A_33_P3237927	8.12E-03	2.70	Rho GTPase activating protein 4	Homo sapiens Rho GTPase activating protein 4 (ARHGAP4), transcript variant 1, mRNA [NM_001164741]
INO80D	A_23_P209652	8.14E-03	-2.09	INO80 complex subunit D	Homo sapiens INO80 complex subunit D (INO80D), mRNA [NM_017759]
STARD5	A_23_P158880	8.16E-03	2.62	StAR-related lipid transfer (START) domain containing 5	Homo sapiens StAR-related lipid transfer (START) domain containing 5 (STARD5), mRNA [NM_181900]
PPM1N	A_33_P3279681	8.20E-03	2.24	protein phosphatase, Mg2+/Mn2+ dependent, 1N (putative)	Homo sapiens protein phosphatase, Mg2+/Mn2+ dependent, 1N (putative) (PPM1N), mRNA [NM_001080401]
PIF1	A_23_P416468	8.20E-03	2.97	PIF1 5'-to-3' DNA helicase homolog (S. cerevisiae)	Homo sapiens PIF1 5'-to-3' DNA helicase homolog (S. cerevisiae) (PIF1), mRNA [NM_025049]
AGAP3	A_33_P3240543	8.20E-03	-2.27	ArfGAP with GTPase domain, ankyrin repeat and PH domain 3	Homo sapiens ArfGAP with GTPase domain, ankyrin repeat and PH domain 3 (AGAP3), transcript variant 2, mRNA [NM_001042535]

B3GAT1	A_23_P1833	8.30E-03	6.52	beta-1,3-glucuronyltransferase 1 (glucuronosyltransferase P)	Homo sapiens beta-1,3-glucuronyltransferase 1 (glucuronosyltransferase P) (B3GAT1), transcript variant 2, mRNA [NM_054025]
WNT3	A_23_P130158	8.35E-03	-2.38	wingless-type MMTV integration site family, member 3	Homo sapiens wingless-type MMTV integration site family, member 3 (WNT3), mRNA [NM_030753]
RNASE6	A_23_P3014	8.39E-03	5.26	ribonuclease, RNase A family, k6	Homo sapiens ribonuclease, RNase A family, k6 (RNASE6), mRNA [NM_005615]
RASSF10	A_33_P3399634	8.41E-03	-3.73	Ras association (RalGDS/AF-6) domain family (N-terminal) member 10	Homo sapiens Ras association (RalGDS/AF-6) domain family (N-terminal) member 10 (RASSF10), mRNA [NM_001080521]
LOC284276	A_33_P3387766	8.43E-03	2.41	uncharacterized LOC284276	Homo sapiens uncharacterized LOC284276 (LOC284276), non-coding RNA [NR_015417]
KRTCAP3	A_23_P433132	8.44E-03	3.78	keratinocyte associated protein 3	Homo sapiens keratinocyte associated protein 3 (KRTCAP3), transcript variant 2, mRNA [NM_173853]
ENDOG	A_23_P83266	8.44E-03	-2.07	endonuclease G	Homo sapiens endonuclease G (ENDOG), nuclear gene encoding mitochondrial protein, mRNA [NM_004435]
SCGB1D2	A_23_P150555	8.50E-03	-8.50	secretoglobin, family 1D, member 2	Homo sapiens secretoglobin, family 1D, member 2 (SCGB1D2), mRNA [NM_006551]
NCKAP1L	A_23_P128201	8.53E-03	8.12	NCK-associated protein 1-like	Homo sapiens NCK-associated protein 1-like (NCKAP1L), transcript variant 1, mRNA [NM_005337]
LOC100129322	A_33_P3385121	8.54E-03	8.06	uncharacterized LOC100129322	Homo sapiens cDNA FLJ45776 fis, clone NETRP2004090. [AK127678]
IFIT2	A_23_P24004	8.54E-03	3.20	interferon-induced protein with tetratricopeptide repeats 2	Homo sapiens interferon-induced protein with tetratricopeptide repeats 2

					(IFIT2), mRNA [NM_001547]
PCBP1-AS1	A_19_P00317631	8.54E-03	2.42		Homo sapiens PCBP1 antisense RNA 1 (non-protein coding) (PCBP1-AS1), non-coding RNA [NR_033872]
MIS18BP1	A_23_P364107	8.54E-03	2.21	MIS18 binding protein 1	Homo sapiens MIS18 binding protein 1 (MIS18BP1), mRNA [NM_018353]
OMD	A_23_P94397	8.55E-03	4.83	osteomodulin	Homo sapiens osteomodulin (OMD), mRNA [NM_005014]
GRIK2	A_33_P3307144	8.58E-03	-4.91	glutamate receptor, ionotropic, kainate 2	Homo sapiens glutamate receptor, ionotropic, kainate 2 (GRIK2), transcript variant 1, mRNA [NM_021956]
MLLT3	A_24_P192627	8.59E-03	2.35	myeloid/lymphoid or mixed-lineage leukemia (trithorax homolog, Drosophila); translocated to, 3	Homo sapiens myeloid/lymphoid or mixed-lineage leukemia (trithorax homolog, Drosophila); translocated to, 3 (MLLT3), mRNA [NM_004529]
C20orf94	A_23_P364766	8.60E-03	2.40	chromosome 20 open reading frame 94	Homo sapiens chromosome 20 open reading frame 94 (C20orf94), mRNA [NM_001009608]
UBA7	A_23_P21207	8.60E-03	2.36	ubiquitin-like modifier activating enzyme 7	Homo sapiens ubiquitin-like modifier activating enzyme 7 (UBA7), mRNA [NM_003335]
FAM89A	A_24_P418408	8.63E-03	-2.43	family with sequence similarity 89, member A	Homo sapiens family with sequence similarity 89, member A (FAM89A), mRNA [NM_198552]
CMAHP	A_33_P3281567	8.69E-03	2.04	cytidine monophospho-N-acetylneuraminic acid hydroxylase, pseudogene	Homo sapiens cytidine monophospho-N-acetylneuraminic acid hydroxylase, pseudogene (CMAHP), transcript variant 1, non-coding RNA [NR_002174]
CD1C	A_33_P3273474	8.70E-03	3.41	CD1c molecule	Homo sapiens CD1c molecule (CD1C), mRNA [NM_001765]
PSTPIP2	A_24_P322353	8.71E-03	4.46	proline-serine-threonine phosphatase interacting protein 2	Homo sapiens proline-serine-threonine phosphatase interacting protein 2 (PSTPIP2), mRNA [NM_024430]

TNFAIP3	A_24_P157926	8.71E-03	4.49	tumor necrosis factor, alpha-induced protein 3	Homo sapiens tumor necrosis factor, alpha-induced protein 3 (TNFAIP3), mRNA [NM_006290]
P2RY2	A_23_P24903	8.71E-03	-3.56	purinergic receptor P2Y, G-protein coupled, 2	Homo sapiens purinergic receptor P2Y, G-protein coupled, 2 (P2RY2), transcript variant 1, mRNA [NM_176072]
HESX1	A_23_P121106	8.71E-03	3.26	HESX homeobox 1	Homo sapiens HESX homeobox 1 (HESX1), mRNA [NM_003865]
ERCC2	A_24_P401990	8.71E-03	-2.06	excision repair cross-complementing rodent repair deficiency, complementation group 2	Homo sapiens excision repair cross-complementing rodent repair deficiency, complementation group 2 (ERCC2), transcript variant 1, mRNA [NM_000400]
RAD1	A_23_P144697	8.71E-03	-2.02	RAD1 homolog (S. pombe)	Homo sapiens RAD1 homolog (S. pombe) (RAD1), transcript variant 1, mRNA [NM_002853]
JMJD6	A_23_P311616	8.76E-03	-2.36	jumonji domain containing 6	Homo sapiens jumonji domain containing 6 (JMJD6), transcript variant 2, mRNA [NM_015167]
PARP15	A_33_P3316544	8.77E-03	4.38	poly (ADP-ribose) polymerase family, member 15	Homo sapiens poly (ADP-ribose) polymerase family, member 15 (PARP15), transcript variant 1, mRNA [NM_001113523]
SLC40A1	A_23_P102391	8.79E-03	2.04	solute carrier family 40 (iron-regulated transporter), member 1	Homo sapiens solute carrier family 40 (iron-regulated transporter), member 1 (SLC40A1), mRNA [NM_014585]
RARRES3	A_23_P1962	8.92E-03	5.18	retinoic acid receptor responder (tazarotene induced) 3	Homo sapiens retinoic acid receptor responder (tazarotene induced) 3 (RARRES3), mRNA [NM_004585]
LINC00174	A_23_P397543	8.99E-03	4.74	long intergenic non-protein coding RNA 174	Homo sapiens long intergenic non-protein coding RNA 174 (LINC00174), non-coding RNA [NR_026873]
ATXN7L2	A_23_P317207	9.00E-03	-2.61	ataxin 7-like 2	Homo sapiens ataxin 7-like 2 (ATXN7L2), mRNA [NM_153340]

CFP	A_23_P22444	9.02E-03	3.52	complement factor properdin	Homo sapiens complement factor properdin (CFP), transcript variant 1, mRNA [NM_002621]
TLCD1	A_23_P420417	9.07E-03	-2.43	TLC domain containing 1	Homo sapiens TLC domain containing 1 (TLCD1), transcript variant 1, mRNA [NM_138463]
RHEBL1	A_33_P3264895	9.14E-03	2.37	Ras homolog enriched in brain like 1	Homo sapiens Ras homolog enriched in brain like 1 (RHEBL1), mRNA [NM_144593]
ADRB1	A_33_P3310189	9.15E-03	-2.21	adrenergic, beta-1-, receptor	Homo sapiens adrenergic, beta-1-, receptor (ADRB1), mRNA [NM_000684]
SYNRG	A_23_P396626	9.15E-03	2.15	synergin, gamma	Homo sapiens synergin, gamma (SYNRG), transcript variant 1, mRNA [NM_007247]
MTMR9LP	A_33_P3354975	9.19E-03	-2.23	myotubularin related protein 9-like, pseudogene	Homo sapiens myotubularin related protein 9-like, pseudogene (MTMR9LP), non-coding RNA [NR_026850]
UBE2QL1	A_24_P479551	9.26E-03	-2.35	ubiquitin-conjugating enzyme E2Q family-like 1	Homo sapiens ubiquitin-conjugating enzyme E2Q family-like 1 (UBE2QL1), mRNA [NM_001145161]
RTKN2	A_24_P13041	9.35E-03	3.81	rhotekin 2	Homo sapiens rhotekin 2 (RTKN2), mRNA [NM_145307]
COL3A1	A_24_P935491	9.35E-03	3.54	collagen, type III, alpha 1	Homo sapiens collagen, type III, alpha 1 (COL3A1), mRNA [NM_000090]
RASL11B	A_23_P69738	9.38E-03	5.97	RAS-like, family 11, member B	Homo sapiens RAS-like, family 11, member B (RASL11B), mRNA [NM_023940]
ZNRD1-AS1	A_33_P3219840	9.42E-03	3.08	ZNRD1 antisense RNA 1 (non-protein coding)	Homo sapiens ZNRD1 antisense RNA 1 (non-protein coding) (ZNRD1-AS1), non-coding RNA [NR_026751]
LARP6	A_23_P117782	9.44E-03	-2.14	La ribonucleoprotein domain family, member 6	Homo sapiens La ribonucleoprotein domain family, member 6 (LARP6), transcript variant 1, mRNA [NM_018357]

SLA2	A_23_P143173	9.49E-03	5.03	Src-like-adaptor 2	Homo sapiens Src-like-adaptor 2 (SLA2), transcript variant 1, mRNA [NM_032214]
XLOC_002049	A_19_P00320954	9.49E-03	-2.30		DB092735 TESTI4 Homo sapiens cDNA clone TESTI4042456 5', mRNA sequence [DB092735]
CEP164	A_23_P75609	9.49E-03	2.03	centrosomal protein 164kDa	Homo sapiens centrosomal protein 164kDa (CEP164), mRNA [NM_014956]
PAPLN	A_23_P333640	9.52E-03	4.58	papilin, proteoglycan-like sulfated glycoprotein	Homo sapiens papilin, proteoglycan-like sulfated glycoprotein (PAPLN), mRNA [NM_173462]
IKBKE	A_23_P887	9.52E-03	2.68	inhibitor of kappa light polypeptide gene enhancer in B-cells, kinase epsilon	Homo sapiens inhibitor of kappa light polypeptide gene enhancer in B-cells, kinase epsilon (IKBKE), transcript variant 1, mRNA [NM_014002]
C11orf65	A_23_P418485	9.52E-03	2.39	chromosome 11 open reading frame 65	Homo sapiens chromosome 11 open reading frame 65 (C11orf65), mRNA [NM_152587]
CXCR2P1	A_33_P3375859	9.56E-03	18.38	chemokine (C-X-C motif) receptor 2 pseudogene 1	Homo sapiens chemokine (C-X-C motif) receptor 2 pseudogene 1 (CXCR2P1), non-coding RNA [NR_002712]
KRTAP3-3	A_33_P3411315	9.58E-03	-3.73	keratin associated protein 3-3	Homo sapiens keratin associated protein 3-3 (KRTAP3-3), mRNA [NM_033185]
C1QTNF2	A_33_P3403153	9.61E-03	3.72	C1q and tumor necrosis factor related protein 2	Homo sapiens C1q and tumor necrosis factor related protein 2 (C1QTNF2), mRNA [NM_031908]
SAMSN1	A_23_P29005	9.63E-03	7.42	SAM domain, SH3 domain and nuclear localization signals 1	Homo sapiens SAM domain, SH3 domain and nuclear localization signals 1 (SAMSN1), mRNA [NM_022136]
IL15RA	A_33_P3399268	9.63E-03	-2.95	interleukin 15 receptor, alpha	interleukin 15 receptor, alpha [Source:HGNC Symbol;Acc:5978] [ENST00000379971]
CTSK	A_23_P34744	9.64E-03	2.97	cathepsin K	Homo sapiens cathepsin K (CTSK), mRNA [NM_000396]

MNDA	A_23_P137935	9.70E-03	5.02	myeloid cell nuclear differentiation antigen	Homo sapiens myeloid cell nuclear differentiation antigen (MNDA), mRNA [NM_002432]
KRT86	A_23_P363769	9.70E-03	3.31	keratin 86	Homo sapiens keratin 86 (KRT86), mRNA [NM_002284]
HRH2	A_23_P7535	9.70E-03	-2.08	histamine receptor H2	Homo sapiens histamine receptor H2 (HRH2), transcript variant 2, mRNA [NM_022304]
LOC100507199	A_33_P3407863	9.70E-03	-2.07	uncharacterized LOC100507199	PREDICTED: Homo sapiens hypothetical LOC100507199, transcript variant 1 (LOC100507199), miscRNA [XR_110934]
ERN1	A_23_P164042	9.75E-03	2.95	endoplasmic reticulum to nucleus signaling 1	Homo sapiens endoplasmic reticulum to nucleus signaling 1 (ERN1), mRNA [NM_001433]
RPL27A	A_23_P416305	9.79E-03	-3.47	ribosomal protein L27a	Homo sapiens ribosomal protein L27a (RPL27A), mRNA [NM_000990]
NACC2	A_23_P113825	9.83E-03	-2.61	NACC family member 2, BEN and BTB (POZ) domain containing	Homo sapiens NACC family member 2, BEN and BTB (POZ) domain containing (NACC2), mRNA [NM_144653]
MOG	A_33_P3391791	9.83E-03	-5.28	myelin oligodendrocyte glycoprotein	Homo sapiens myelin oligodendrocyte glycoprotein (MOG), transcript variant alpha1, mRNA [NM_206809]
C6orf192	A_23_P134113	9.87E-03	2.12	chromosome 6 open reading frame 192	Homo sapiens chromosome 6 open reading frame 192 (C6orf192), mRNA [NM_052831]
RRAD	A_24_P262127	9.87E-03	-7.91	Ras-related associated with diabetes	Homo sapiens Ras-related associated with diabetes (RRAD), transcript variant 2, mRNA [NM_004165]
RAB7B	A_23_P252541	9.87E-03	3.75	RAB7B, member RAS oncogene family	Homo sapiens RAB7B, member RAS oncogene family (RAB7B), transcript variant 1, mRNA [NM_177403]
ATP8B2	A_24_P366749	9.90E-03	2.77	ATPase, class I, type 8B, member 2	Homo sapiens ATPase, class I, type 8B, member 2 (ATP8B2), transcript variant 2,

					mRNA [NM_001005855]
LOC283861	A_33_P3577142	1.00E-02	2.81	uncharacterized LOC283861	Homo sapiens cDNA FLJ40824 fis, clone TRACH2011113. [AK098143]
WSCD1	A_24_P942493	1.00E-02	-3.32	WSC domain containing 1	Homo sapiens WSC domain containing 1 (WSCD1), mRNA [NM_015253]
C6orf174	A_33_P3284453	1.01E-02	4.47	chromosome 6 open reading frame 174	Homo sapiens chromosome 6 open reading frame 174 (C6orf174), mRNA [NM_001012279]
BCAR1	A_33_P3369034	1.01E-02	-2.28	breast cancer anti-estrogen resistance 1	Homo sapiens breast cancer anti-estrogen resistance 1 (BCAR1), transcript variant 1, mRNA [NM_001170714]
ADAM11	A_33_P3386775	1.01E-02	-3.33	ADAM metallopeptidase domain 11	Homo sapiens ADAM metallopeptidase domain 11 (ADAM11), mRNA [NM_002390]
ANKRD44	A_23_P209347	1.01E-02	3.17	ankyrin repeat domain 44	Homo sapiens ankyrin repeat domain 44 (ANKRD44), transcript variant B, mRNA [NM_153697]
USP2	A_24_P107317	1.01E-02	-2.01	ubiquitin specific peptidase 2	Homo sapiens ubiquitin specific peptidase 2 (USP2), transcript variant 1, mRNA [NM_004205]
CBLN3	A_33_P3412900	1.01E-02	2.79	cerebellin 3 precursor	Homo sapiens cerebellin 3 precursor (CBLN3), mRNA [NM_001039771]
YPEL1	A_32_P88120	1.01E-02	2.10	yippee-like 1 (Drosophila)	Homo sapiens yippee-like 1 (Drosophila) (YPEL1), mRNA [NM_013313]
DNASE1	A_23_P66311	1.01E-02	2.07	deoxyribonuclease I	Homo sapiens deoxyribonuclease I (DNASE1), mRNA [NM_005223]
STXBP2	A_33_P3388835	1.02E-02	3.47	syntaxin binding protein 2	Homo sapiens syntaxin binding protein 2 (STXBP2), transcript variant 1, mRNA [NM_006949]
LOC285556	A_33_P3411155	1.02E-02	-40.89	uncharacterized LOC285556	PREDICTED: Homo sapiens hypothetical protein LOC285556 (LOC285556), mRNA [XM_001717423]
FLT3	A_23_P99442	1.02E-02	5.91	fms-related tyrosine kinase 3	Homo sapiens fms-related tyrosine

					kinase 3 (FLT3), mRNA [NM_004119]
NLRC4	A_23_P119835	1.02E-02	5.29	NLR family, CARD domain containing 4	Homo sapiens NLR family, CARD domain containing 4 (NLRC4), transcript variant 1, mRNA [NM_021209]
ASB2	A_23_P205370	1.02E-02	-2.12	ankyrin repeat and SOCS box containing 2	Homo sapiens ankyrin repeat and SOCS box containing 2 (ASB2), transcript variant 2, mRNA [NM_016150]
GPR45	A_23_P131534	1.02E-02	-2.12	G protein-coupled receptor 45	Homo sapiens G protein-coupled receptor 45 (GPR45), mRNA [NM_007227]
MAGEB1	A_23_P217341	1.03E-02	-4.17	melanoma antigen family B, 1	Homo sapiens melanoma antigen family B, 1 (MAGEB1), transcript variant 1, mRNA [NM_002363]
CARD9	A_23_P500433	1.03E-02	4.85	caspase recruitment domain family, member 9	Homo sapiens caspase recruitment domain family, member 9 (CARD9), transcript variant 1, mRNA [NM_052813]
SNX22	A_33_P3391517	1.03E-02	-3.04	sorting nexin 22	sorting nexin 22 [Source:HGNC Symbol;Acc:16315] [ENST00000380278]
RP1-177G6.2	A_33_P3343820	1.03E-02	-2.65	uncharacterized LOC286411	Homo sapiens uncharacterized LOC286411 (RP1-177G6.2), transcript variant 1, non-coding RNA [NR_028344]
GMFG	A_23_P208866	1.03E-02	3.04	glia maturation factor, gamma	Homo sapiens glia maturation factor, gamma (GMFG), mRNA [NM_004877]
ARRDC3	A_24_P274615	1.05E-02	2.96	arrestin domain containing 3	Homo sapiens arrestin domain containing 3 (ARRDC3), mRNA [NM_020801]
LOC100130691	A_19_P00315584	1.05E-02	2.15	uncharacterized LOC100130691	Homo sapiens uncharacterized LOC100130691 (LOC100130691), non-coding RNA [NR_026966]
PTAFR	A_24_P102821	1.05E-02	4.43	platelet-activating factor receptor	Homo sapiens platelet-activating factor receptor (PTAFR), transcript variant 3, mRNA [NM_000952]
FREM1	A_23_P43337	1.05E-02	3.33	FRAS1 related extracellular	Homo sapiens FRAS1 related

				matrix 1	extracellular matrix 1 (FREM1), transcript variant 1, mRNA [NM_144966]
TNFRSF19	A_23_P140057	1.05E-02	-2.87	tumor necrosis factor receptor superfamily, member 19	Homo sapiens tumor necrosis factor receptor superfamily, member 19 (TNFRSF19), transcript variant 1, mRNA [NM_018647]
STAB1	A_23_P32500	1.07E-02	2.55	stabilin 1	Homo sapiens stabilin 1 (STAB1), mRNA [NM_015136]
COL5A2	A_23_P33196	1.07E-02	2.43	collagen, type V, alpha 2	Homo sapiens collagen, type V, alpha 2 (COL5A2), mRNA [NM_000393]
REEP4	A_23_P96209	1.07E-02	2.19	receptor accessory protein 4	Homo sapiens receptor accessory protein 4 (REEP4), mRNA [NM_025232]
MR1	A_23_P74928	1.07E-02	2.29	major histocompatibility complex, class I-related	Homo sapiens major histocompatibility complex, class I-related (MR1), transcript variant 1, mRNA [NM_001531]
SIDT1	A_23_P132515	1.07E-02	4.25	SID1 transmembrane family, member 1	Homo sapiens SID1 transmembrane family, member 1 (SIDT1), mRNA [NM_017699]
GLTPD1	A_33_P3266396	1.07E-02	-2.13	glycolipid transfer protein domain containing 1	Homo sapiens glycolipid transfer protein domain containing 1 (GLTPD1), mRNA [NM_001029885]
ZNF812	A_32_P33434	1.08E-02	3.35	zinc finger protein 812	Homo sapiens zinc finger protein 812 (ZNF812), mRNA [NM_001199814]
ATHL1	A_23_P98686	1.08E-02	2.57	ATH1, acid trehalase-like 1 (yeast)	Homo sapiens ATH1, acid trehalase-like 1 (yeast) (ATHL1), mRNA [NM_025092]
CASC2	A_33_P3213362	1.08E-02	4.76	cancer susceptibility candidate 2 (non-protein coding)	Homo sapiens cancer susceptibility candidate 2 (non-protein coding) (CASC2), transcript variant 1, non-coding RNA [NR_026939]
HAAO	A_33_P3244122	1.08E-02	3.94	3-hydroxyanthranilate 3,4-dioxygenase	Homo sapiens 3-hydroxyanthranilate 3,4-dioxygenase (HAAO), mRNA [NM_012205]
CD1E	A_23_P310410	1.09E-02	5.13	CD1e molecule	Homo sapiens CD1e molecule (CD1E),

					transcript variant 2, mRNA [NM_001042583]
MYCL1	A_33_P3306068	1.09E-02	6.23	v-myc myelocytomatosis viral oncogene homolog 1, lung carcinoma derived (avian)	Homo sapiens v-myc myelocytomatosis viral oncogene homolog 1, lung carcinoma derived (avian) (MYCL1), transcript variant 1, mRNA [NM_001033081]
IGF1	A_23_P13907	1.09E-02	2.72	insulin-like growth factor 1 (somatomedin C)	Homo sapiens insulin-like growth factor 1 (somatomedin C) (IGF1), transcript variant 4, mRNA [NM_000618]
CACNA1B	A_33_P3280114	1.09E-02	-3.42	calcium channel, voltage-dependent, N type, alpha 1B subunit	calcium channel, voltage-dependent, N type, alpha 1B subunit [Source:HGNC Symbol;Acc:1389] [ENST00000371365]
LINC00173	A_33_P3331511	1.10E-02	3.47	long intergenic non-protein coding RNA 173	Homo sapiens long intergenic non-protein coding RNA 173 (LINC00173), transcript variant 1, non-coding RNA [NR_027345]
MARVELD1	A_23_P138725	1.10E-02	2.18	MARVEL domain containing 1	Homo sapiens MARVEL domain containing 1 (MARVELD1), mRNA [NM_031484]
PRB1	A_33_P3258593	1.10E-02	-2.37	proline-rich protein BstNI subfamily 1	Homo sapiens proline-rich protein BstNI subfamily 1 (PRB1), transcript variant 1, mRNA [NM_005039]
F13A1	A_33_P3416097	1.10E-02	2.61	coagulation factor XIII, A1 polypeptide	Homo sapiens coagulation factor XIII, A1 polypeptide (F13A1), mRNA [NM_000129]
C5	A_23_P71855	1.11E-02	3.81	complement component 5	Homo sapiens complement component 5 (C5), mRNA [NM_001735]
HCP5	A_24_P17870	1.11E-02	2.87	HLA complex P5 (non-protein coding)	Homo sapiens HLA complex P5 (non-protein coding) (HCP5), non-coding RNA [NR_040662]
C1orf35	A_33_P3263523	1.11E-02	2.28	chromosome 1 open reading frame 35	Homo sapiens chromosome 1 open reading frame 35 (C1orf35), mRNA

					[NM_024319]
SUSD5	A_24_P238131	1.11E-02	2.63	sushi domain containing 5	Homo sapiens sushi domain containing 5 (SUSD5), mRNA [NM_015551]
MID1IP1	A_33_P3217465	1.11E-02	-3.59	MID1 interacting protein 1 (gastrulation specific G12 homolog (zebrafish))	Homo sapiens MID1 interacting protein 1 (gastrulation specific G12 homolog (zebrafish)) (MID1IP1), transcript variant 1, mRNA [NM_021242]
LAMP3	A_23_P29773	1.12E-02	14.05	lysosomal-associated membrane protein 3	Homo sapiens lysosomal-associated membrane protein 3 (LAMP3), mRNA [NM_014398]
NUP210	A_23_P212159	1.12E-02	4.33	nucleoporin 210kDa	Homo sapiens nucleoporin 210kDa (NUP210), mRNA [NM_024923]
RLN2	A_23_P216455	1.12E-02	3.77	relaxin 2	Homo sapiens relaxin 2 (RLN2), transcript variant 2, mRNA [NM_005059]
XLOC_003641	A_19_P00804922	1.12E-02	-2.64		BROAD Institute lincRNA (XLOC_003641), lincRNA [TCONS_00008193]
C1QTNF7	A_32_P190769	1.12E-02	7.08	C1q and tumor necrosis factor related protein 7	Homo sapiens C1q and tumor necrosis factor related protein 7 (C1QTNF7), transcript variant 3, mRNA [NM_031911]
C16orf89	A_33_P3296862	1.13E-02	5.38	chromosome 16 open reading frame 89	Homo sapiens chromosome 16 open reading frame 89 (C16orf89), transcript variant 1, mRNA [NM_152459]
LRRC4C	A_23_P24457	1.13E-02	3.19	leucine rich repeat containing 4C	Homo sapiens leucine rich repeat containing 4C (LRRC4C), mRNA [NM_020929]
SLC43A3	A_24_P406986	1.13E-02	-2.01	solute carrier family 43, member 3	Homo sapiens solute carrier family 43, member 3 (SLC43A3), mRNA [NM_199329]
TMEM132E	A_33_P3287108	1.14E-02	4.13	transmembrane protein 132E	Homo sapiens transmembrane protein 132E (TMEM132E), mRNA [NM_207313]
DEFB128	A_33_P3272962	1.14E-02	-3.19	defensin, beta 128	Homo sapiens defensin, beta 128 (DEFB128), mRNA [NM_001037732]

LOC100131733	A_32_P56249	1.15E-02	5.94	uncharacterized LOC100131733	Homo sapiens uncharacterized LOC100131733 (LOC100131733), non-coding RNA [NR_038996]
MXRA8	A_24_P353905	1.15E-02	2.33	matrix-remodelling associated 8	Homo sapiens matrix-remodelling associated 8 (MXRA8), mRNA [NM_032348]
CD19	A_23_P113572	1.15E-02	8.23	CD19 molecule	Homo sapiens CD19 molecule (CD19), transcript variant 2, mRNA [NM_001770]
FKBP11	A_33_P3267296	1.15E-02	4.93	FK506 binding protein 11, 19 kDa	Homo sapiens FK506 binding protein 11, 19 kDa (FKBP11), transcript variant 1, mRNA [NM_016594]
IL12RB1	A_23_P399156	1.15E-02	2.88	interleukin 12 receptor, beta 1	Homo sapiens interleukin 12 receptor, beta 1 (IL12RB1), transcript variant 2, mRNA [NM_153701]
C5orf46	A_33_P3392052	1.16E-02	5.85	chromosome 5 open reading frame 46	Homo sapiens chromosome 5 open reading frame 46 (C5orf46), mRNA [NM_206966]
LOC100128019	A_33_P3259960	1.16E-02	3.39	uncharacterized LOC100128019	Homo sapiens cDNA FLJ43591 fis, clone SMINT2002743. [AK125579]
LOC100129098	A_33_P3417432	1.17E-02	-2.72	uncharacterized LOC100129098	PREDICTED: Homo sapiens hypothetical protein LOC100129098 (LOC100129098), mRNA [XM_001714893]
MICAL1	A_24_P283341	1.17E-02	2.16	microtubule associated monoxygenase, calponin and LIM domain containing 1	Homo sapiens microtubule associated monoxygenase, calponin and LIM domain containing 1 (MICAL1), transcript variant 1, mRNA [NM_022765]
ICAM4	A_33_P3363355	1.17E-02	2.94	intercellular adhesion molecule 4 (Landsteiner-Wiener blood group)	Homo sapiens intercellular adhesion molecule 4 (Landsteiner-Wiener blood group) (ICAM4), transcript variant 2, mRNA [NM_022377]
SPATA18	A_23_P407112	1.17E-02	2.69	spermatogenesis associated 18 homolog (rat)	Homo sapiens spermatogenesis associated 18 homolog (rat) (SPATA18), mRNA [NM_145263]

NPAS3	A_23_P2942	1.17E-02	2.29	neuronal PAS domain protein 3	Homo sapiens neuronal PAS domain protein 3 (NPAS3), transcript variant 2, mRNA [NM_022123]
KIF26B	A_33_P3396746	1.18E-02	-2.85	kinesin family member 26B	kinesin family member 26B [Source:HGNC Symbol;Acc:25484] [ENST00000479506]
LOC386597	A_33_P3519424	1.18E-02	-3.06	uncharacterized LOC386597	Homo sapiens cDNA FLJ32573 fis, clone SPLEN2000210. [AK057135]
MS4A1	A_33_P3406567	1.19E-02	5.12	membrane-spanning 4-domains, subfamily A, member 1	Homo sapiens membrane-spanning 4-domains, subfamily A, member 1 (MS4A1), transcript variant 1, mRNA [NM_152866]
EBI3	A_23_P119478	1.19E-02	5.27	Epstein-Barr virus induced 3	Homo sapiens Epstein-Barr virus induced 3 (EBI3), mRNA [NM_005755]
KIF21B	A_23_P126888	1.20E-02	2.69	kinesin family member 21B	Homo sapiens kinesin family member 21B (KIF21B), transcript variant 2, mRNA [NM_017596]
IGSF22	A_32_P486620	1.20E-02	-3.58	immunoglobulin superfamily, member 22	Homo sapiens immunoglobulin superfamily, member 22 (IGSF22), mRNA [NM_173588]
NBPF6	A_33_P3229328	1.21E-02	2.06	neuroblastoma breakpoint family, member 6	Homo sapiens neuroblastoma breakpoint family, member 6 (NBPF6), transcript variant 2, mRNA [NM_001143988]
HMOX2	A_23_P100501	1.21E-02	-2.47	heme oxygenase (decycling) 2	Homo sapiens heme oxygenase (decycling) 2 (HMOX2), transcript variant 3, mRNA [NM_002134]
BOC	A_23_P257763	1.21E-02	2.25	Boc homolog (mouse)	Homo sapiens Boc homolog (mouse) (BOC), mRNA [NM_033254]
OGN	A_33_P3279590	1.23E-02	6.98	osteoglycin	Homo sapiens osteoglycin (OGN), transcript variant 1, mRNA [NM_033014]
ZNF214	A_23_P127840	1.23E-02	3.15	zinc finger protein 214	Homo sapiens zinc finger protein 214 (ZNF214), mRNA [NM_013249]

SERPINA1	A_23_P218111	1.23E-02	4.65	serpin peptidase inhibitor, clade A (alpha-1 antiproteinase, antitrypsin), member 1	Homo sapiens serpin peptidase inhibitor, clade A (alpha-1 antiproteinase, antitrypsin), member 1 (SERPINA1), transcript variant 2, mRNA [NM_001002236]
ZNF215	A_33_P3310981	1.23E-02	6.07	zinc finger protein 215	Homo sapiens zinc finger protein 215 (ZNF215), mRNA [NM_013250]
TRPC4	A_23_P105873	1.23E-02	-2.68	transient receptor potential cation channel, subfamily C, member 4	Homo sapiens transient receptor potential cation channel, subfamily C, member 4 (TRPC4), transcript variant alpha, mRNA [NM_016179]
SPINK5	A_33_P3391126	1.23E-02	4.84	serine peptidase inhibitor, Kazal type 5	Homo sapiens serine peptidase inhibitor, Kazal type 5 (SPINK5), transcript variant 1, mRNA [NM_001127698]
TRGV7	A_33_P3638471	1.23E-02	4.01	T cell receptor gamma variable 7 (pseudogene)	Homo sapiens T cell receptor gamma variable 7 pseudogene, mRNA (cDNA clone IMAGE:5210958). [BC027954]
FAM115C	A_24_P237912	1.24E-02	2.27	family with sequence similarity 115, member C	Homo sapiens family with sequence similarity 115, member C (FAM115C), transcript variant 3, mRNA [NM_001130026]
GPR65	A_23_P14564	1.24E-02	5.06	G protein-coupled receptor 65	Homo sapiens G protein-coupled receptor 65 (GPR65), mRNA [NM_003608]
PPIL1	A_23_P133995	1.25E-02	-2.03	peptidylprolyl isomerase (cyclophilin)-like 1	Homo sapiens peptidylprolyl isomerase (cyclophilin)-like 1 (PPIL1), mRNA [NM_016059]
GINS2	A_23_P118246	1.25E-02	2.70	GINS complex subunit 2 (Psf2 homolog)	Homo sapiens GINS complex subunit 2 (Psf2 homolog) (GINS2), mRNA [NM_016095]
ZNF169	A_33_P3421365	1.25E-02	2.02	zinc finger protein 169	Homo sapiens zinc finger protein 169 (ZNF169), mRNA [NM_194320]
KIAA0513	A_23_P206310	1.25E-02	2.03	KIAA0513	Homo sapiens KIAA0513 (KIAA0513),

					mRNA [NM_014732]
ACSM1	A_23_P106933	1.26E-02	3.47	acyl-CoA synthetase medium-chain family member 1	Homo sapiens acyl-CoA synthetase medium-chain family member 1 (ACSM1), mRNA [NM_052956]
CXorf57	A_23_P96369	1.26E-02	3.24	chromosome X open reading frame 57	Homo sapiens chromosome X open reading frame 57 (CXorf57), transcript variant 1, mRNA [NM_018015]
LOC645431	A_23_P327156	1.26E-02	2.23	uncharacterized LOC645431	Homo sapiens uncharacterized LOC645431 (LOC645431), non-coding RNA [NR_024334]
PLEKHH2	A_33_P3315719	1.26E-02	2.91	pleckstrin homology domain containing, family H (with MyTH4 domain) member 2	Homo sapiens pleckstrin homology domain containing, family H (with MyTH4 domain) member 2 (PLEKHH2), mRNA [NM_172069]
IL18	A_23_P104798	1.27E-02	4.81	interleukin 18 (interferon-gamma-inducing factor)	Homo sapiens interleukin 18 (interferon-gamma-inducing factor) (IL18), transcript variant 1, mRNA [NM_001562]
STARD10	A_23_P36345	1.27E-02	-2.70	StAR-related lipid transfer (START) domain containing 10	Homo sapiens StAR-related lipid transfer (START) domain containing 10 (STARD10), mRNA [NM_006645]
SERPINA6	A_23_P117363	1.28E-02	-2.85	serpin peptidase inhibitor, clade A (alpha-1 antiproteinase, antitrypsin), member 6	Homo sapiens serpin peptidase inhibitor, clade A (alpha-1 antiproteinase, antitrypsin), member 6 (SERPINA6), mRNA [NM_001756]
SCARNA5	A_33_P3363082	1.28E-02	3.03	small Cajal body-specific RNA 5	Homo sapiens small Cajal body-specific RNA 5 (SCARNA5), guide RNA [NR_003008]
Q8YNJ1	A_19_P00322166	1.28E-02	2.97		Q8YNJ1_ANASP (Q8YNJ1) Permease protein of phosphate ABC transporter, partial (6%) [THC2671932]
TUSC1	A_32_P133884	1.28E-02	-2.11	tumor suppressor candidate 1	Homo sapiens tumor suppressor candidate 1 (TUSC1), mRNA [NM_001004125]

ITGAX	A_23_P312132	1.28E-02	6.81	integrin, alpha X (complement component 3 receptor 4 subunit)	Homo sapiens integrin, alpha X (complement component 3 receptor 4 subunit) (ITGAX), mRNA [NM_000887]
ZMAT1	A_24_P11100	1.28E-02	3.30	zinc finger, matrin-type 1	Homo sapiens zinc finger, matrin-type 1 (ZMAT1), transcript variant 1, mRNA [NM_001011657]
TRAF5	A_23_P201731	1.28E-02	2.39	TNF receptor-associated factor 5	Homo sapiens TNF receptor-associated factor 5 (TRAF5), transcript variant 1, mRNA [NM_004619]
IL18RAP	A_23_P28334	1.28E-02	5.96	interleukin 18 receptor accessory protein	Homo sapiens interleukin 18 receptor accessory protein (IL18RAP), mRNA [NM_003853]
HLA-DMA	A_23_P42306	1.29E-02	3.21	major histocompatibility complex, class II, DM alpha	Homo sapiens major histocompatibility complex, class II, DM alpha (HLA-DMA), mRNA [NM_006120]
LTC4S	A_24_P397294	1.29E-02	3.19	leukotriene C4 synthase	Homo sapiens leukotriene C4 synthase (LTC4S), mRNA [NM_145867]
SLITRK4	A_32_P131640	1.29E-02	5.22	SLIT and NTRK-like family, member 4	Homo sapiens SLIT and NTRK-like family, member 4 (SLITRK4), transcript variant 2, mRNA [NM_173078]
HES6	A_33_P3265359	1.29E-02	-3.30	hairy and enhancer of split 6 (Drosophila)	Homo sapiens hairy and enhancer of split 6 (Drosophila) (HES6), transcript variant 1, mRNA [NM_018645]
CACNA2D3	A_33_P3373259	1.29E-02	-2.56	calcium channel, voltage-dependent, alpha 2/delta subunit 3	Homo sapiens calcium channel, voltage-dependent, alpha 2/delta subunit 3 (CACNA2D3), mRNA [NM_018398]
PLA2G7	A_23_P145096	1.29E-02	8.25	phospholipase A2, group VII (platelet-activating factor acetylhydrolase, plasma)	Homo sapiens phospholipase A2, group VII (platelet-activating factor acetylhydrolase, plasma) (PLA2G7), transcript variant 1, mRNA [NM_005084]
PGBD2	A_33_P3286608	1.29E-02	2.10	piggyBac transposable element derived 2	Homo sapiens piggyBac transposable element derived 2 (PGBD2), transcript variant 1, mRNA [NM_170725]

OSBPL3	A_23_P215525	1.30E-02	2.47	oxysterol binding protein-like 3	Homo sapiens oxysterol binding protein-like 3 (OSBPL3), transcript variant 1, mRNA [NM_015550]
TMSB4X	A_24_P374516	1.31E-02	2.04	thymosin beta 4, X-linked	Homo sapiens thymosin beta 4, X-linked (TMSB4X), mRNA [NM_021109]
FAM125A	A_33_P3291732	1.32E-02	2.97	family with sequence similarity 125, member A	family with sequence similarity 125, member A [Source:HGNC Symbol;Acc:25153] [ENST00000529490]
CYBA	A_33_P3372332	1.32E-02	2.69	cytochrome b-245, alpha polypeptide	Homo sapiens cytochrome b-245, alpha polypeptide (CYBA), mRNA [NM_000101]
CDRT3	A_33_P3629131	1.32E-02	-2.16	CMT1A duplicated region transcript 3	DKFZp686P15240_r1 686 (synonym: hlcc3) Homo sapiens cDNA clone DKFZp686P15240 5', mRNA sequence [BX484257]
SDC1	A_23_P16944	1.32E-02	3.66	syndecan 1	Homo sapiens syndecan 1 (SDC1), transcript variant 1, mRNA [NM_001006946]
KNG1	A_23_P212258	1.32E-02	-3.40	kininogen 1	Homo sapiens kininogen 1 (KNG1), transcript variant 2, mRNA [NM_000893]
C8orf71	A_33_P3238182	1.32E-02	-2.65	chromosome 8 open reading frame 71	Homo sapiens chromosome 8 open reading frame 71 (C8orf71), non-coding RNA [NR_026772]
TLR5	A_23_P85903	1.33E-02	2.99	toll-like receptor 5	Homo sapiens toll-like receptor 5 (TLR5), mRNA [NM_003268]
UNC93B1	A_33_P3404470	1.33E-02	2.32	unc-93 homolog B1 (C. elegans)	Homo sapiens unc-93 homolog B1 (C. elegans) (UNC93B1), mRNA [NM_030930]
PLXDC2	A_23_P161424	1.33E-02	2.17	plexin domain containing 2	Homo sapiens plexin domain containing 2 (PLXDC2), mRNA [NM_032812]
PTN	A_24_P870620	1.33E-02	2.69	pleiotrophin	Homo sapiens pleiotrophin (PTN), mRNA [NM_002825]
PTPRO	A_23_P204304	1.34E-02	3.76	protein tyrosine phosphatase,	Homo sapiens protein tyrosine

				receptor type, O	phosphatase, receptor type, O (PTPRO), transcript variant 1, mRNA [NM_030667]
COMTD1	A_33_P3257222	1.34E-02	-2.04	catechol-O-methyltransferase domain containing 1	Homo sapiens catechol-O-methyltransferase domain containing 1 (COMTD1), mRNA [NM_144589]
LOC100507006	A_19_P00317704	1.34E-02	3.91		PREDICTED: Homo sapiens hypothetical LOC100507006 (LOC100507006), miscRNA [XR_109941]
ZNF846	A_33_P3306823	1.35E-02	3.60	zinc finger protein 846	Homo sapiens zinc finger protein 846 (ZNF846), mRNA [NM_001077624]
CGNL1	A_23_P163306	1.35E-02	2.85	cingulin-like 1	Homo sapiens cingulin-like 1 (CGNL1), transcript variant 2, mRNA [NM_032866]
LTB	A_33_P3248265	1.35E-02	8.56	lymphotoxin beta (TNF superfamily, member 3)	Homo sapiens lymphotoxin beta (TNF superfamily, member 3) (LTB), transcript variant 1, mRNA [NM_002341]
CCL3	A_33_P3316273	1.36E-02	6.04	chemokine (C-C motif) ligand 3	Homo sapiens chemokine (C-C motif) ligand 3 (CCL3), mRNA [NM_002983]
TMEM236	A_33_P3382031	1.36E-02	4.00	transmembrane protein 236	Homo sapiens transmembrane protein 236 (TMEM236), mRNA [NM_001098844]
RNF213	A_19_P00317052	1.36E-02	3.05		Homo sapiens ring finger protein 213 (RNF213), transcript variant 1, mRNA [NM_020914]
LOC100652917	A_24_P694820	1.36E-02	2.26	uncharacterized LOC100652917	PREDICTED: Homo sapiens hypothetical LOC100652917 (LOC100652917), miscRNA [XR_132741]
IL12RB2	A_23_P72077	1.37E-02	3.67	interleukin 12 receptor, beta 2	Homo sapiens interleukin 12 receptor, beta 2 (IL12RB2), mRNA [NM_001559]
C1orf96	A_33_P3361202	1.37E-02	2.46	chromosome 1 open reading frame 96	Homo sapiens chromosome 1 open reading frame 96 (C1orf96), mRNA [NM_145257]
TMEM63B	A_23_P167856	1.38E-02	-2.87	transmembrane protein 63B	Homo sapiens transmembrane protein 63B (TMEM63B), mRNA [NM_018426]

LOC554207	A_24_P109887	1.38E-02	-2.57	uncharacterized LOC554207	Homo sapiens hypothetical LOC554207, mRNA (cDNA clone MGC:21504 IMAGE:3882600), complete cds. [BC031469]
ATP5G1	A_23_P164228	1.38E-02	-2.26	ATP synthase, H+ transporting, mitochondrial Fo complex, subunit C1 (subunit 9)	Homo sapiens ATP synthase, H+ transporting, mitochondrial Fo complex, subunit C1 (subunit 9) (ATP5G1), nuclear gene encoding mitochondrial protein, transcript variant 1, mRNA [NM_005175]
TACC3	A_23_P212844	1.38E-02	2.78	transforming, acidic coiled-coil containing protein 3	Homo sapiens transforming, acidic coiled-coil containing protein 3 (TACC3), mRNA [NM_006342]
ZBTB37	A_23_P137504	1.38E-02	2.42	zinc finger and BTB domain containing 37	Homo sapiens zinc finger and BTB domain containing 37 (ZBTB37), transcript variant 2, mRNA [NM_032522]
TRIP13	A_33_P3407256	1.38E-02	-2.29	thyroid hormone receptor interactor 13	Homo sapiens thyroid hormone receptor interactor 13 (TRIP13), transcript variant 2, mRNA [NM_001166260]
FOXP2	A_24_P921683	1.38E-02	3.36	forkhead box P2	Homo sapiens forkhead box P2 (FOXP2), transcript variant 1, mRNA [NM_014491]
FAM83F	A_23_P343963	1.38E-02	-2.83	family with sequence similarity 83, member F	Homo sapiens family with sequence similarity 83, member F (FAM83F), mRNA [NM_138435]
ANK2	A_23_P133068	1.38E-02	-3.86	ankyrin 2, neuronal	Homo sapiens ankyrin 2, neuronal (ANK2), transcript variant 1, mRNA [NM_001148]
SNHG5	A_19_P00316541	1.38E-02	-2.37	small nucleolar RNA host gene 5 (non-protein coding)	Homo sapiens small nucleolar RNA host gene 5 (non-protein coding) (SNHG5), non-coding RNA [NR_003038]
C5orf25	A_33_P3282384	1.38E-02	2.16	chromosome 5 open reading frame 25	chromosome 5 open reading frame 25 [Source:HGNC Symbol;Acc:24779] [ENST00000377277]
RAB8B	A_23_P317465	1.39E-02	2.13	RAB8B, member RAS oncogene	Homo sapiens RAB8B, member RAS

				family	oncogene family (RAB8B), mRNA [NM_016530]
KCNJ5	A_24_P309521	1.40E-02	-2.87	potassium inwardly-rectifying channel, subfamily J, member 5	Homo sapiens potassium inwardly-rectifying channel, subfamily J, member 5 (KCNJ5), mRNA [NM_000890]
TLR3	A_23_P29922	1.40E-02	2.12	toll-like receptor 3	Homo sapiens toll-like receptor 3 (TLR3), mRNA [NM_003265]
FAM72A	A_33_P3242952	1.40E-02	5.54	family with sequence similarity 72, member A	Homo sapiens family with sequence similarity 72, member A (FAM72A), mRNA [NM_001123168]
PILRA	A_23_P31224	1.40E-02	2.09	paired immunoglobin-like type 2 receptor alpha	Homo sapiens paired immunoglobin-like type 2 receptor alpha (PILRA), transcript variant 3, mRNA [NM_178273]
DDX60	A_23_P41470	1.40E-02	2.32	DEAD (Asp-Glu-Ala-Asp) box polypeptide 60	Homo sapiens DEAD (Asp-Glu-Ala-Asp) box polypeptide 60 (DDX60), mRNA [NM_017631]
DOK2	A_23_P134851	1.41E-02	5.42	docking protein 2, 56kDa	Homo sapiens docking protein 2, 56kDa (DOK2), mRNA [NM_003974]
PTGER3	A_33_P3265749	1.41E-02	-3.44	prostaglandin E receptor 3 (subtype EP3)	Homo sapiens prostaglandin E receptor 3 (subtype EP3) (PTGER3), transcript variant 7, mRNA [NM_198717]
TNFRSF18	A_24_P411121	1.41E-02	3.48	tumor necrosis factor receptor superfamily, member 18	Homo sapiens tumor necrosis factor receptor superfamily, member 18 (TNFRSF18), transcript variant 2, mRNA [NM_148901]
RPH3A	A_33_P3395384	1.41E-02	-3.04	rabphilin 3A homolog (mouse)	Homo sapiens rabphilin 3A homolog (mouse) (RPH3A), transcript variant 1, mRNA [NM_001143854]
MYOG	A_23_P160438	1.41E-02	-3.82	myogenin (myogenic factor 4)	Homo sapiens myogenin (myogenic factor 4) (MYOG), mRNA [NM_002479]
DRD4	A_33_P3373765	1.42E-02	2.33	dopamine receptor D4	Homo sapiens dopamine receptor D4 (DRD4), mRNA [NM_000797]
SNORA76	A_33_P3876591	1.42E-02	-2.50	small nucleolar RNA, H/ACA box	AGENCOURT_13972169 NIH_MGC_173

				76	Homo sapiens cDNA 5', mRNA sequence [CD050382]
KIAA0408	A_23_P215048	1.42E-02	2.74	KIAA0408	Homo sapiens KIAA0408 (KIAA0408), mRNA [NM_014702]
PPP4R1L	A_24_P65851	1.42E-02	2.01	protein phosphatase 4, regulatory subunit 1-like	Homo sapiens protein phosphatase 4, regulatory subunit 1-like (PPP4R1L), non-coding RNA [NR_003505]
HSPA6	A_23_P114903	1.43E-02	4.03	heat shock 70kDa protein 6 (HSP70B')	Homo sapiens heat shock 70kDa protein 6 (HSP70B') (HSPA6), mRNA [NM_002155]
DCHS2	A_23_P144490	1.43E-02	-3.37	dachsous 2 (Drosophila)	Homo sapiens dachsous 2 (Drosophila) (DCHS2), transcript variant 1, mRNA [NM_017639]
HSBP1L1	A_32_P46981	1.43E-02	-2.04	heat shock factor binding protein 1-like 1	Homo sapiens heat shock factor binding protein 1-like 1 (HSBP1L1), mRNA [NM_001136180]
NLRP3	A_23_P9883	1.43E-02	4.02	NLR family, pyrin domain containing 3	Homo sapiens NLR family, pyrin domain containing 3 (NLRP3), transcript variant 3, mRNA [NM_001079821]
CCDC69	A_24_P97825	1.43E-02	-2.40	coiled-coil domain containing 69	Homo sapiens coiled-coil domain containing 69 (CCDC69), mRNA [NM_015621]
C1orf127	A_32_P468341	1.44E-02	-2.61	chromosome 1 open reading frame 127	Homo sapiens chromosome 1 open reading frame 127 (C1orf127), mRNA [NM_001170754]
C6orf26	A_24_P3804	1.46E-02	2.62	chromosome 6 open reading frame 26	Homo sapiens chromosome 6 open reading frame 26 (C6orf26), mRNA [NM_001039651]
TMED8	A_33_P3346157	1.47E-02	2.30	transmembrane emp24 protein transport domain containing 8	Homo sapiens transmembrane emp24 protein transport domain containing 8 (TMED8), mRNA [NM_213601]
C1orf38	A_24_P85775	1.48E-02	4.01	chromosome 1 open reading frame 38	Homo sapiens chromosome 1 open reading frame 38 (C1orf38), transcript

					variant 2, mRNA [NM_001039477]
VCY	A_33_P3291510	1.48E-02	-2.58	variable charge, Y-linked	Homo sapiens variable charge, Y-linked (VCY), mRNA [NM_004679]
SLC7A7	A_23_P99642	1.48E-02	3.83	solute carrier family 7 (amino acid transporter light chain, y+L system), member 7	Homo sapiens solute carrier family 7 (amino acid transporter light chain, y+L system), member 7 (SLC7A7), transcript variant 3, mRNA [NM_001126106]
DKFZp779M0652	A_32_P189790	1.49E-02	-2.86	uncharacterized DKFZp779M0652	Homo sapiens uncharacterized DKFZp779M0652 (DKFZp779M0652), non-coding RNA [NR_027134]
VNN2	A_23_P122724	1.49E-02	2.58	vanin 2	Homo sapiens vanin 2 (VNN2), transcript variant 1, mRNA [NM_004665]
MTFP1	A_23_P29204	1.49E-02	-2.46	mitochondrial fission process 1	Homo sapiens mitochondrial fission process 1 (MTFP1), nuclear gene encoding mitochondrial protein, transcript variant 1, mRNA [NM_016498]
LAIR1	A_33_P3253394	1.50E-02	3.87	leukocyte-associated immunoglobulin-like receptor 1	Homo sapiens leukocyte-associated immunoglobulin-like receptor 1 (LAIR1), transcript variant a, mRNA [NM_002287]
ART4	A_23_P116902	1.50E-02	3.53	ADP-ribosyltransferase 4 (Dombrock blood group)	Homo sapiens ADP-ribosyltransferase 4 (Dombrock blood group) (ART4), mRNA [NM_021071]
DNAJB5	A_23_P112241	1.50E-02	-2.40	DnaJ (Hsp40) homolog, subfamily B, member 5	Homo sapiens DnaJ (Hsp40) homolog, subfamily B, member 5 (DNAJB5), transcript variant 3, mRNA [NM_012266]
CEL	A_33_P3219303	1.50E-02	-2.25	carboxyl ester lipase (bile salt-stimulated lipase)	Homo sapiens carboxyl ester lipase (bile salt-stimulated lipase) (CEL), mRNA [NM_001807]
LOC645261	A_33_P3499692	1.50E-02	-2.60	PP565	Homo sapiens PP565 mRNA, complete cds. [AF258587]
LOC651337	A_33_P3350892	1.50E-02	-2.38	uncharacterized LOC651337	PREDICTED: Homo sapiens hypothetical LOC651337 (LOC651337), miscRNA [XR_132727]

PDE4DIP	A_23_P200801	1.50E-02	-2.07	phosphodiesterase 4D interacting protein	Homo sapiens phosphodiesterase 4D interacting protein (PDE4DIP), transcript variant 5, mRNA [NM_001002811]
PTPRH	A_23_P101642	1.50E-02	-3.25	protein tyrosine phosphatase, receptor type, H	Homo sapiens protein tyrosine phosphatase, receptor type, H (PTPRH), transcript variant 1, mRNA [NM_002842]
KIAA1456	A_32_P159234	1.50E-02	-2.86	KIAA1456	Homo sapiens KIAA1456 (KIAA1456), transcript variant 1, mRNA [NM_020844]
C4orf46	A_33_P3377691	1.50E-02	2.19	chromosome 4 open reading frame 46	Homo sapiens chromosome 4 open reading frame 46 (C4orf46), mRNA [NM_001008393]
CDH16	A_23_P100240	1.51E-02	-6.46	cadherin 16, KSP-cadherin	Homo sapiens cadherin 16, KSP-cadherin (CDH16), transcript variant 1, mRNA [NM_004062]
LOC100506123	A_33_P3884179	1.51E-02	4.84	uncharacterized LOC100506123	Homo sapiens uncharacterized LOC100506123 (LOC100506123), non-coding RNA [NR_040097]
C1QC	A_23_P125977	1.51E-02	3.33	complement component 1, q subcomponent, C chain	Homo sapiens complement component 1, q subcomponent, C chain (C1QC), transcript variant 2, mRNA [NM_172369]
TMEM198	A_24_P822704	1.51E-02	-2.13	transmembrane protein 198	Homo sapiens transmembrane protein 198 (TMEM198), mRNA [NM_001005209]
LOC100127983	A_24_P683011	1.53E-02	-2.13	uncharacterized LOC100127983	Homo sapiens uncharacterized protein LOC100127983 (LOC100127983), mRNA [NM_001190972]
PLAC8	A_24_P183128	1.53E-02	4.91	placenta-specific 8	Homo sapiens placenta-specific 8 (PLAC8), transcript variant 2, mRNA [NM_016619]
PKLR	A_23_P201022	1.53E-02	-2.63	pyruvate kinase, liver and RBC	Homo sapiens pyruvate kinase, liver and RBC (PKLR), nuclear gene encoding mitochondrial protein, transcript variant 1, mRNA [NM_000298]

MSI2	A_23_P44195	1.53E-02	-2.26	musashi homolog 2 (Drosophila)	Homo sapiens musashi homolog 2 (Drosophila) (MSI2), transcript variant 1, mRNA [NM_138962]
PITPNM2	A_23_P401361	1.53E-02	-2.34	phosphatidylinositol transfer protein, membrane-associated 2	Homo sapiens phosphatidylinositol transfer protein, membrane-associated 2 (PITPNM2), mRNA [NM_020845]
GRAMD4	A_24_P23258	1.53E-02	-2.24	GRAM domain containing 4	Homo sapiens GRAM domain containing 4 (GRAMD4), mRNA [NM_015124]
C10orf105	A_33_P3322999	1.53E-02	4.24	chromosome 10 open reading frame 105	Homo sapiens chromosome 10 open reading frame 105 (C10orf105), transcript variant 1, mRNA [NM_001164375]
CNN1	A_23_P125233	1.54E-02	-4.71	calponin 1, basic, smooth muscle	Homo sapiens calponin 1, basic, smooth muscle (CNN1), mRNA [NM_001299]
C6orf204	A_33_P3407339	1.54E-02	-2.66	chromosome 6 open reading frame 204	Homo sapiens chromosome 6 open reading frame 204 (C6orf204), transcript variant 2, mRNA [NM_206921]
MOSPD1	A_33_P3330353	1.54E-02	-2.34	motile sperm domain containing 1	Homo sapiens motile sperm domain containing 1 (MOSPD1), mRNA [NM_019556]
FOXI3	A_24_P15391	1.54E-02	-2.45	forkhead box I3	Homo sapiens forkhead box I3 (FOXI3), mRNA [NM_001135649]
AQP10	A_23_P126613	1.55E-02	3.50	aquaporin 10	Homo sapiens aquaporin 10 (AQP10), mRNA [NM_080429]
XRCC3	A_33_P3301524	1.55E-02	2.02	X-ray repair complementing defective repair in Chinese hamster cells 3	Homo sapiens X-ray repair complementing defective repair in Chinese hamster cells 3 (XRCC3), transcript variant 1, mRNA [NM_001100119]
NPC1L1	A_24_P324712	1.55E-02	-2.61	NPC1 (Niemann-Pick disease, type C1, gene)-like 1	Homo sapiens NPC1 (Niemann-Pick disease, type C1, gene)-like 1 (NPC1L1), transcript variant 1, mRNA [NM_013389]
HOPX	A_23_P254507	1.55E-02	-5.49	HOP homeobox	Homo sapiens HOP homeobox (HOPX),

					transcript variant 2, mRNA [NM_139211]
KLHDC8B	A_24_P148836	1.55E-02	-2.11	kelch domain containing 8B	Homo sapiens kelch domain containing 8B (KLHDC8B), mRNA [NM_173546]
ABLIM2	A_33_P3381666	1.55E-02	-2.25	actin binding LIM protein family, member 2	Homo sapiens actin binding LIM protein family, member 2 (ABLIM2), transcript variant 7, mRNA [NM_001130088]
PAQR5	A_33_P3368750	1.55E-02	-4.01	progestin and adipoQ receptor family member V	Homo sapiens progestin and adipoQ receptor family member V (PAQR5), transcript variant 1, mRNA [NM_001104554]
WAS	A_23_P96331	1.55E-02	2.03	Wiskott-Aldrich syndrome (eczema-thrombocytopenia)	Homo sapiens Wiskott-Aldrich syndrome (eczema-thrombocytopenia) (WAS), mRNA [NM_000377]
LOC157381	A_33_P3793702	1.55E-02	-2.27	uncharacterized LOC157381	Homo sapiens uncharacterized LOC157381 (LOC157381), non-coding RNA [NR_027321]
METTL21A	A_23_P209337	1.55E-02	-2.05	methyltransferase like 21A	Homo sapiens methyltransferase like 21A (METTL21A), transcript variant 1, mRNA [NM_145280]
TMEM171	A_32_P110390	1.56E-02	-2.64	transmembrane protein 171	Homo sapiens transmembrane protein 171 (TMEM171), transcript variant 1, mRNA [NM_173490]
C5orf39	A_33_P3299279	1.56E-02	2.21	chromosome 5 open reading frame 39	Homo sapiens chromosome 5 open reading frame 39 (C5orf39), mRNA [NM_001014279]
FCN3	A_33_P3275741	1.56E-02	-2.17	ficolin (collagen/fibrinogen domain containing) 3 (Hakata antigen)	ficolin (collagen/fibrinogen domain containing) 3 (Hakata antigen) [Source:HGNC Symbol;Acc:3625] [ENST00000498393]
HLA-E	A_32_P460973	1.56E-02	2.12	major histocompatibility complex, class I, E	Homo sapiens major histocompatibility complex, class I, E (HLA-E), mRNA [NM_005516]
CGA	A_23_P42386	1.56E-02	-8.65	glycoprotein hormones, alpha	Homo sapiens glycoprotein hormones,

				polypeptide	alpha polypeptide (CGA), transcript variant 2, mRNA [NM_000735]
PDE4D	A_33_P3389653	1.57E-02	-2.94	phosphodiesterase 4D, cAMP-specific	Homo sapiens phosphodiesterase 4D, cAMP-specific (PDE4D), transcript variant 3, mRNA [NM_001165899]
MXRA5	A_23_P258136	1.57E-02	8.60	matrix-remodelling associated 5	Homo sapiens matrix-remodelling associated 5 (MXRA5), mRNA [NM_015419]
FMNL1	A_24_P390668	1.57E-02	3.34	formin-like 1	Homo sapiens formin-like 1 (FMNL1), mRNA [NM_005892]
HOGA1	A_33_P3249076	1.57E-02	2.39	4-hydroxy-2-oxoglutarate aldolase 1	4-hydroxy-2-oxoglutarate aldolase 1 [Source:HGNC Symbol;Acc:25155] [ENST00000465608]
LARGE	A_23_P91697	1.57E-02	-2.00	like-glycosyltransferase	Homo sapiens like-glycosyltransferase (LARGE), transcript variant 1, mRNA [NM_004737]
LOC100508196	A_19_P00322332	1.57E-02	3.36	uncharacterized LOC100508196	PREDICTED: Homo sapiens hypothetical LOC100508196 (LOC100508196), miscRNA [XR_111691]
SOX10	A_23_P143694	1.58E-02	-2.98	SRY (sex determining region Y)-box 10	Homo sapiens SRY (sex determining region Y)-box 10 (SOX10), mRNA [NM_006941]
CFH	A_33_P3318288	1.58E-02	3.25	complement factor H	Homo sapiens complement factor H (CFH), nuclear gene encoding mitochondrial protein, transcript variant 2, mRNA [NM_001014975]
ABCC5	A_33_P3298057	1.58E-02	-2.19	ATP-binding cassette, sub-family C (CFTR/MRP), member 5	Homo sapiens ATP-binding cassette, sub-family C (CFTR/MRP), member 5 (ABCC5), transcript variant 2, mRNA [NM_001023587]
ITGB2	A_23_P329573	1.59E-02	6.30	integrin, beta 2 (complement component 3 receptor 3 and 4 subunit)	Homo sapiens integrin, beta 2 (complement component 3 receptor 3 and 4 subunit) (ITGB2), transcript variant

					1, mRNA [NM_000211]
KCNA4	A_24_P150023	1.59E-02	3.75	potassium voltage-gated channel, shaker-related subfamily, member 4	Homo sapiens potassium voltage-gated channel, shaker-related subfamily, member 4 (KCNA4), mRNA [NM_002233]
TMEM156	A_23_P92334	1.59E-02	3.49	transmembrane protein 156	Homo sapiens transmembrane protein 156 (TMEM156), mRNA [NM_024943]
SGMS2	A_23_P326204	1.59E-02	-2.30	sphingomyelin synthase 2	Homo sapiens sphingomyelin synthase 2 (SGMS2), transcript variant 1, mRNA [NM_152621]
ANKK1	A_33_P3336053	1.62E-02	3.03	ankyrin repeat and kinase domain containing 1	Homo sapiens ankyrin repeat and kinase domain containing 1 (ANKK1), mRNA [NM_178510]
SH2D2A	A_23_P160618	1.62E-02	2.42	SH2 domain containing 2A	Homo sapiens SH2 domain containing 2A (SH2D2A), transcript variant 2, mRNA [NM_003975]
NFATC2	A_23_P91278	1.62E-02	3.04	nuclear factor of activated T-cells, cytoplasmic, calcineurin-dependent 2	Homo sapiens nuclear factor of activated T-cells, cytoplasmic, calcineurin-dependent 2 (NFATC2), transcript variant 2, mRNA [NM_173091]
LOX	A_23_P122216	1.62E-02	2.78	lysyl oxidase	Homo sapiens lysyl oxidase (LOX), transcript variant 1, mRNA [NM_002317]
LOC100507429	A_19_P00803039	1.63E-02	3.33	uncharacterized LOC100507429	PREDICTED: Homo sapiens hypothetical LOC100507429 (LOC100507429), miscRNA [XR_110179]
MRC1	A_23_P12746	1.63E-02	4.01	mannose receptor, C type 1	Homo sapiens mannose receptor, C type 1 (MRC1), mRNA [NM_002438]
CD1B	A_23_P351844	1.63E-02	4.51	CD1b molecule	Homo sapiens CD1b molecule (CD1B), mRNA [NM_001764]
F2R	A_23_P213562	1.64E-02	3.00	coagulation factor II (thrombin) receptor	Homo sapiens coagulation factor II (thrombin) receptor (F2R), mRNA [NM_001992]
FCAR	A_24_P348265	1.64E-02	2.96	Fc fragment of IgA, receptor for	Homo sapiens Fc fragment of IgA,

					receptor for (FCAR), transcript variant 3, mRNA [NM_133271]
AP1S3	A_33_P3289005	1.64E-02	2.22	adaptor-related protein complex 1, sigma 3 subunit	Homo sapiens adaptor-related protein complex 1, sigma 3 subunit (AP1S3), mRNA [NM_001039569]
KLRF1	A_33_P3274501	1.64E-02	2.10	killer cell lectin-like receptor subfamily F, member 1	Homo sapiens killer cell lectin-like receptor subfamily F, member 1 (KLRF1), mRNA [NM_016523]
SIGLEC1	A_23_P17481	1.65E-02	3.96	sialic acid binding Ig-like lectin 1, sialoadhesin	Homo sapiens sialic acid binding Ig-like lectin 1, sialoadhesin (SIGLEC1), mRNA [NM_023068]
HLA-DOB	A_23_P30736	1.65E-02	2.77	major histocompatibility complex, class II, DO beta	Homo sapiens major histocompatibility complex, class II, DO beta (HLA-DOB), mRNA [NM_002120]
SRGAP2	A_32_P50123	1.67E-02	2.05	SLIT-ROBO Rho GTPase activating protein 2	Homo sapiens SLIT-ROBO Rho GTPase activating protein 2 (SRGAP2), transcript variant 2, mRNA [NM_001042758]
PRDX6	A_23_P983	1.68E-02	-2.01	peroxiredoxin 6	Homo sapiens peroxiredoxin 6 (PRDX6), mRNA [NM_004905]
SFRP4	A_33_P3335177	1.69E-02	8.34	secreted frizzled-related protein 4	Homo sapiens secreted frizzled-related protein 4 (SFRP4), mRNA [NM_003014]
CLECL1	A_23_P321984	1.69E-02	3.45	C-type lectin-like 1	Homo sapiens C-type lectin-like 1 (CLECL1), mRNA [NM_172004]
INPP5D	A_23_P61149	1.69E-02	2.95	inositol polyphosphate-5-phosphatase, 145kDa	Homo sapiens inositol polyphosphate-5-phosphatase, 145kDa (INPP5D), transcript variant 1, mRNA [NM_001017915]
TCOF1	A_24_P243396	1.70E-02	-2.29	Treacher Collins-Franceschetti syndrome 1	Homo sapiens Treacher Collins-Franceschetti syndrome 1 (TCOF1), transcript variant 3, mRNA [NM_001008657]
OTUD6A	A_24_P42389	1.70E-02	-3.20	OTU domain containing 6A	Homo sapiens OTU domain containing 6A (OTUD6A), mRNA [NM_207320]

TCF7	A_33_P3250671	1.70E-02	2.99	transcription factor 7 (T-cell specific, HMG-box)	Homo sapiens transcription factor 7 (T-cell specific, HMG-box) (TCF7), transcript variant 1, mRNA [NM_003202]
DDX11L2	A_32_P227317	1.70E-02	-3.15	DEAD/H (Asp-Glu-Ala-Asp/His) box polypeptide 11 like 2	Homo sapiens DEAD/H (Asp-Glu-Ala-Asp/His) box polypeptide 11 like 2 (DDX11L2), transcript variant 2, non-coding RNA [NR_024005]
ASPHD2	A_24_P245815	1.70E-02	2.91	aspartate beta-hydroxylase domain containing 2	Homo sapiens aspartate beta-hydroxylase domain containing 2 (ASPHD2), mRNA [NM_020437]
HLA-F	A_33_P3379967	1.70E-02	2.66	major histocompatibility complex, class I, F	Homo sapiens major histocompatibility complex, class I, F (HLA-F), transcript variant 3, mRNA [NM_001098478]
IDH2	A_23_P129209	1.70E-02	-2.02	isocitrate dehydrogenase 2 (NADP+), mitochondrial	Homo sapiens isocitrate dehydrogenase 2 (NADP+), mitochondrial (IDH2), nuclear gene encoding mitochondrial protein, mRNA [NM_002168]
CXCL10	A_33_P3343175	1.71E-02	12.85	chemokine (C-X-C motif) ligand 10	Homo sapiens chemokine (C-X-C motif) ligand 10 (CXCL10), mRNA [NM_001565]
WFDC2	A_23_P218675	1.71E-02	-3.28	WAP four-disulfide core domain 2	Homo sapiens WAP four-disulfide core domain 2 (WFDC2), mRNA [NM_006103]
TRMT5	A_33_P3238976	1.72E-02	2.27	TRM5 tRNA methyltransferase 5 homolog (S. cerevisiae)	Homo sapiens TRM5 tRNA methyltransferase 5 homolog (S. cerevisiae) (TRMT5), mRNA [NM_020810]
GABRR3	A_23_P69020	1.74E-02	-2.26	gamma-aminobutyric acid (GABA) receptor, rho 3	Homo sapiens gamma-aminobutyric acid (GABA) receptor, rho 3 (GABRR3), mRNA [NM_001105580]
FLJ42351	A_33_P3387971	1.74E-02	3.38	uncharacterized LOC400999	Homo sapiens cDNA FLJ42351 fis, clone UTERU2005664. [AK124342]
RMRP	A_33_P3401284	1.75E-02	2.06	RNA component of mitochondrial RNA processing endoribonuclease	Homo sapiens RNA component of mitochondrial RNA processing endoribonuclease (RMRP), RNase MRP

					RNA [NR_003051]
PLCB2	A_33_P3260614	1.75E-02	3.45	phospholipase C, beta 2	Homo sapiens phospholipase C, beta 2 (PLCB2), mRNA [NM_004573]
PPM1M	A_23_P258393	1.75E-02	2.12	protein phosphatase, Mg2+/Mn2+ dependent, 1M	Homo sapiens protein phosphatase, Mg2+/Mn2+ dependent, 1M (PPM1M), transcript variant 1, mRNA [NM_144641]
LOC100653245	A_24_P627503	1.75E-02	4.21	ig heavy chain V-III region VH26-like	immunoglobulin heavy variable 3-30 [Source:HGNC Symbol;Acc:5591] [ENST00000390613]
LOC282997	A_33_P3645465	1.76E-02	3.06	uncharacterized LOC282997	Homo sapiens uncharacterized LOC282997 (LOC282997), non-coding RNA [NR_026932]
GHRL	A_23_P40956	1.76E-02	2.60	ghrelin/obestatin prepropeptide	Homo sapiens ghrelin/obestatin prepropeptide (GHRL), transcript variant 1, mRNA [NM_016362]
TTC18	A_23_P326931	1.76E-02	2.01	tetratricopeptide repeat domain 18	Homo sapiens tetratricopeptide repeat domain 18 (TTC18), mRNA [NM_145170]
CCL19	A_23_P123853	1.76E-02	16.14	chemokine (C-C motif) ligand 19	Homo sapiens chemokine (C-C motif) ligand 19 (CCL19), mRNA [NM_006274]
BACH2	A_23_P30634	1.76E-02	2.01	BTB and CNC homology 1, basic leucine zipper transcription factor 2	Homo sapiens BTB and CNC homology 1, basic leucine zipper transcription factor 2 (BACH2), transcript variant 1, mRNA [NM_021813]
TGM1	A_23_P65618	1.77E-02	2.79	transglutaminase 1 (K polypeptide epidermal type I, protein-glutamine-gamma-glutamyltransferase)	Homo sapiens transglutaminase 1 (K polypeptide epidermal type I, protein-glutamine-gamma-glutamyltransferase) (TGM1), mRNA [NM_000359]
LOC100272228	A_19_P00320226	1.77E-02	2.64	uncharacterized LOC100272228	Homo sapiens uncharacterized LOC100272228 (LOC100272228), non-coding RNA [NR_027456]
MATL2963	A_33_P3365408	1.77E-02	4.35	uncharacterized LOC283314	Homo sapiens uncharacterized LOC283314 (MATL2963), non-coding RNA [NR_026947]

CYTH4	A_24_P74559	1.77E-02	3.33	cytohesin 4	Homo sapiens cytohesin 4 (CYTH4), mRNA [NM_013385]
GOLM1	A_33_P3231653	1.77E-02	2.10	golgi membrane protein 1	Homo sapiens golgi membrane protein 1 (GOLM1), transcript variant 1, mRNA [NM_016548]
LOC100130430	A_33_P3227934	1.77E-02	-2.08	uncharacterized LOC100130430	Homo sapiens cDNA FLJ45052 fis, clone BRAWH3022542. [AK126997]
SSC5D	A_33_P3358898	1.77E-02	5.08	scavenger receptor cysteine rich domain containing (5 domains)	Homo sapiens scavenger receptor cysteine rich domain containing (5 domains) (SSC5D), transcript variant 1, mRNA [NM_001144950]
C17orf66	A_23_P430792	1.77E-02	2.85	chromosome 17 open reading frame 66	Homo sapiens chromosome 17 open reading frame 66 (C17orf66), mRNA [NM_152781]
LOC100129034	A_32_P91042	1.78E-02	2.03	uncharacterized LOC100129034	Homo sapiens uncharacterized LOC100129034 (LOC100129034), non-coding RNA [NR_027406]
AMD1	A_24_P371962	1.79E-02	-2.24	adenosylmethionine decarboxylase 1	Homo sapiens adenosylmethionine decarboxylase 1 (AMD1), transcript variant 1, mRNA [NM_001634]
TMEM189	A_33_P3259801	1.79E-02	-2.20	transmembrane protein 189	Homo sapiens transmembrane protein 189 (TMEM189), transcript variant 1, mRNA [NM_199129]
NOTCH2NL	A_33_P3212640	1.80E-02	2.70	notch 2 N-terminal like	Homo sapiens notch 2 N-terminal like (NOTCH2NL), mRNA [NM_203458]
ACR	A_32_P191262	1.80E-02	-2.10	acrosin	Homo sapiens acrosin (ACR), mRNA [NM_001097]
CABLES2	A_23_P307761	1.82E-02	-2.36	Cdk5 and Abl enzyme substrate 2	Homo sapiens Cdk5 and Abl enzyme substrate 2 (CABLES2), mRNA [NM_031215]
ANKRD30BP2	A_24_P917819	1.82E-02	5.11	ankyrin repeat domain 30B pseudogene 2	Homo sapiens ankyrin repeat domain 30B pseudogene 2 (ANKRD30BP2), non-coding RNA [NR_026916]

PDE9A	A_33_P3419594	1.82E-02	-3.64	phosphodiesterase 9A	Homo sapiens cDNA FLJ90181 fis, clone MAMMA1000706. [AK074662]
LOC284108	A_33_P3634554	1.83E-02	2.50	uncharacterized LOC284108	full-length cDNA clone CS0DB003YF07 of Neuroblastoma Cot 10-normalized of Homo sapiens (human) [CR616125]
RGS10	A_23_P138717	1.83E-02	3.43	regulator of G-protein signaling 10	Homo sapiens regulator of G-protein signaling 10 (RGS10), transcript variant 1, mRNA [NM_001005339]
COTL1	A_24_P416131	1.84E-02	3.90	coactosin-like 1 (Dictyostelium)	Homo sapiens coactosin-like 1 (Dictyostelium) (COTL1), mRNA [NM_021149]
LOC388780	A_33_P3241661	1.85E-02	-2.20	uncharacterized LOC388780	PREDICTED: Homo sapiens hypothetical LOC388780 (LOC388780), miscRNA [XR_109642]
CTRC	A_23_P34852	1.85E-02	-2.12	chymotrypsin C (caldecrin)	Homo sapiens chymotrypsin C (caldecrin) (CTRC), mRNA [NM_007272]
COL4A3	A_23_P170679	1.85E-02	2.47	collagen, type IV, alpha 3 (Goodpasture antigen)	Homo sapiens collagen, type IV, alpha 3 (Goodpasture antigen) (COL4A3), mRNA [NM_000091]
TONSL	A_24_P1054	1.85E-02	-2.26	tonsoku-like, DNA repair protein	Homo sapiens tonsoku-like, DNA repair protein (TONSL), mRNA [NM_013432]
HMGN2	A_32_P41487	1.85E-02	2.14	high mobility group nucleosomal binding domain 2	Homo sapiens high mobility group nucleosomal binding domain 2 (HMGN2), mRNA [NM_005517]
LMO3	A_24_P381441	1.86E-02	5.29	LIM domain only 3 (rhombotin-like 2)	Homo sapiens LIM domain only 3 (rhombotin-like 2) (LMO3), transcript variant 1, mRNA [NM_018640]
COL6A6	A_32_P224525	1.86E-02	4.55	collagen, type VI, alpha 6	Homo sapiens collagen, type VI, alpha 6 (COL6A6), mRNA [NM_001102608]
ANKRD22	A_33_P3407880	1.87E-02	9.33	ankyrin repeat domain 22	Homo sapiens ankyrin repeat domain 22 (ANKRD22), mRNA [NM_144590]
ARHGAP9	A_23_P64661	1.87E-02	4.34	Rho GTPase activating protein 9	Homo sapiens Rho GTPase activating protein 9 (ARHGAP9), transcript variant

					1, mRNA [NM_032496]
NRADDP	A_33_P3386150	1.88E-02	2.61	neurotrophin receptor associated death domain, pseudogene	Homo sapiens neurotrophin receptor associated death domain, pseudogene (NRADDP), non-coding RNA [NR_024046]
ZC3HAV1	A_33_P3397418	1.90E-02	2.13	zinc finger CCCH-type, antiviral 1	Homo sapiens zinc finger CCCH-type, antiviral 1 (ZC3HAV1), transcript variant 1, mRNA [NM_020119]
RASL10B	A_24_P322229	1.90E-02	-2.19	RAS-like, family 10, member B	Homo sapiens RAS-like, family 10, member B (RASL10B), mRNA [NM_033315]
FANCD2	A_33_P3257808	1.90E-02	2.86	Fanconi anemia, complementation group D2	Homo sapiens Fanconi anemia, complementation group D2 (FANCD2), transcript variant 2, mRNA [NM_001018115]
DHX58	A_23_P38346	1.91E-02	2.17	DEXH (Asp-Glu-X-His) box polypeptide 58	Homo sapiens DEXH (Asp-Glu-X-His) box polypeptide 58 (DHX58), mRNA [NM_024119]
S100A16	A_23_P147918	1.92E-02	-2.23	S100 calcium binding protein A16	Homo sapiens S100 calcium binding protein A16 (S100A16), mRNA [NM_080388]
RANBP6	A_23_P146379	1.93E-02	-2.06	RAN binding protein 6	Homo sapiens RAN binding protein 6 (RANBP6), transcript variant 1, mRNA [NM_012416]
TJP2	A_33_P3215529	1.93E-02	-2.02	tight junction protein 2 (zona occludens 2)	Homo sapiens tight junction protein ZO-2 isoform C mRNA, partial cds. [AF083893]
STAT1	A_24_P274270	1.93E-02	3.87	signal transducer and activator of transcription 1, 91kDa	Homo sapiens signal transducer and activator of transcription 1, 91kDa (STAT1), transcript variant beta, mRNA [NM_139266]
SLC25A34	A_33_P3227904	1.93E-02	3.80	solute carrier family 25, member 34	Homo sapiens solute carrier family 25, member 34 (SLC25A34), mRNA

					[NM_207348]
CLEC4E	A_33_P3318509	1.93E-02	3.58	C-type lectin domain family 4, member E	Homo sapiens C-type lectin domain family 4, member E (CLEC4E), mRNA [NM_014358]
SYK	A_23_P9255	1.93E-02	3.99	spleen tyrosine kinase	Homo sapiens spleen tyrosine kinase (SYK), transcript variant 1, mRNA [NM_003177]
USP51	A_33_P3301915	1.93E-02	3.02	ubiquitin specific peptidase 51	Homo sapiens ubiquitin specific peptidase 51 (USP51), mRNA [NM_201286]
CLEC4A	A_23_P48029	1.94E-02	3.23	C-type lectin domain family 4, member A	Homo sapiens C-type lectin domain family 4, member A (CLEC4A), transcript variant 1, mRNA [NM_016184]
TTC24	A_33_P3297345	1.94E-02	3.16	tetratricopeptide repeat domain 24	Homo sapiens tetratricopeptide repeat domain 24 (TTC24), mRNA [NM_001105669]
LOC100131564	A_24_P481375	1.95E-02	3.08	uncharacterized LOC100131564	Homo sapiens uncharacterized LOC100131564 (LOC100131564), non-coding RNA [NR_034089]
LOC144571	A_33_P3280950	1.95E-02	2.59	uncharacterized LOC144571	Homo sapiens uncharacterized LOC144571 (LOC144571), non-coding RNA [NR_026971]
AIF1	A_23_P214627	1.96E-02	4.25	allograft inflammatory factor 1	Homo sapiens allograft inflammatory factor 1 (AIF1), transcript variant 2, mRNA [NM_004847]
CA13	A_23_P381714	1.96E-02	-2.34	carbonic anhydrase XIII	Homo sapiens carbonic anhydrase XIII (CA13), mRNA [NM_198584]
PPPDE2	A_23_P356330	1.96E-02	-2.08	PPPDE peptidase domain containing 2	Homo sapiens PPPDE peptidase domain containing 2 (PPPDE2), mRNA [NM_015704]
RASGRP1	A_23_P124642	1.96E-02	5.04	RAS guanyl releasing protein 1 (calcium and DAG-regulated)	Homo sapiens RAS guanyl releasing protein 1 (calcium and DAG-regulated) (RASGRP1), transcript variant 1, mRNA

					[NM_005739]
HHAT	A_23_P136355	1.97E-02	2.10	hedgehog acyltransferase	Homo sapiens hedgehog acyltransferase (HHAT), transcript variant 1, mRNA [NM_018194]
SLC36A4	A_23_P86838	1.97E-02	-2.37	solute carrier family 36 (proton/amino acid symporter), member 4	Homo sapiens solute carrier family 36 (proton/amino acid symporter), member 4 (SLC36A4), mRNA [NM_152313]
UMOD	A_23_P158775	1.97E-02	-2.18	uromodulin	Homo sapiens uromodulin (UMOD), transcript variant 1, mRNA [NM_003361]
FLJ46906	A_33_P3340490	1.98E-02	3.66	uncharacterized LOC441172	Homo sapiens uncharacterized LOC441172 (FLJ46906), non-coding RNA [NR_033896]
LPCAT3	A_33_P3295283	1.98E-02	-2.18	lysophosphatidylcholine acyltransferase 3	Homo sapiens lysophosphatidylcholine acyltransferase 3 (LPCAT3), mRNA [NM_005768]
C19orf23	A_33_P3851788	1.98E-02	-5.12	chromosome 19 open reading frame 23	Homo sapiens chromosome 19 open reading frame 23 (C19orf23), non-coding RNA [NR_027271]
OASL	A_23_P139786	1.99E-02	4.18	2'-5'-oligoadenylate synthetase-like	Homo sapiens 2'-5'-oligoadenylate synthetase-like (OASL), transcript variant 1, mRNA [NM_003733]
SPN	A_33_P3404706	2.00E-02	2.39	sialophorin	Homo sapiens sialophorin (SPN), transcript variant 1, mRNA [NM_001030288]
KCNA7	A_23_P164897	2.02E-02	-7.82	potassium voltage-gated channel, shaker-related subfamily, member 7	Homo sapiens potassium voltage-gated channel, shaker-related subfamily, member 7 (KCNA7), mRNA [NM_031886]
PIP4K2A	A_33_P3394065	2.02E-02	2.77	phosphatidylinositol-5-phosphate 4-kinase, type II, alpha	phosphatidylinositol-5-phosphate 4-kinase, type II, alpha [Source:HGNC Symbol;Acc:8997] [ENST00000376565]
HOOK1	A_24_P921897	2.02E-02	-4.28	hook homolog 1 (Drosophila)	Homo sapiens hook homolog 1 (Drosophila) (HOOK1), mRNA

					[NM_015888]
C4orf50	A_33_P3237507	2.02E-02	2.60	chromosome 4 open reading frame 50	chromosome 4 open reading frame 50 [Source:HGNC Symbol;Acc:33766] [ENST00000531445]
KLHL6	A_23_P212655	2.02E-02	4.50	kelch-like 6 (Drosophila)	Homo sapiens kelch-like 6 (Drosophila) (KLHL6), mRNA [NM_130446]
UCMA	A_24_P18270	2.03E-02	4.17	upper zone of growth plate and cartilage matrix associated	Homo sapiens upper zone of growth plate and cartilage matrix associated (UCMA), mRNA [NM_145314]
BMP6	A_23_P19624	2.03E-02	2.92	bone morphogenetic protein 6	Homo sapiens bone morphogenetic protein 6 (BMP6), mRNA [NM_001718]
CCDC168	A_33_P3307008	2.04E-02	3.94	coiled-coil domain containing 168	Homo sapiens coiled-coil domain containing 168 (CCDC168), mRNA [NM_001146197]
SLC6A18	A_24_P212234	2.04E-02	-2.21	solute carrier family 6, member 18	Homo sapiens solute carrier family 6, member 18 (SLC6A18), mRNA [NM_182632]
CD200R1	A_23_P325155	2.04E-02	2.55	CD200 receptor 1	Homo sapiens CD200 receptor 1 (CD200R1), transcript variant 2, mRNA [NM_138939]
PYGM	A_23_P405815	2.04E-02	-2.33	phosphorylase, glycogen, muscle	Homo sapiens phosphorylase, glycogen, muscle (PYGM), transcript variant 1, mRNA [NM_005609]
TFE3	A_23_P84952	2.04E-02	-2.63	transcription factor binding to IGHM enhancer 3	Homo sapiens transcription factor binding to IGHM enhancer 3 (TFE3), mRNA [NM_006521]
LOC729451	A_33_P3279515	2.05E-02	-2.39	uncharacterized LOC729451	Homo sapiens cDNA FLJ39579 fis, clone SKMUS2003168. [AK096898]
GLT8D2	A_23_P48198	2.05E-02	2.87	glycosyltransferase 8 domain containing 2	Homo sapiens glycosyltransferase 8 domain containing 2 (GLT8D2), mRNA [NM_031302]
HLA-DRB1	A_24_P343233	2.06E-02	3.14	major histocompatibility complex, class II, DR beta 1	Homo sapiens major histocompatibility complex, class II, DR beta 1 (HLA-DRB1),

					transcript variant 1, mRNA [NM_002124]
AMIGO2	A_23_P14083	2.06E-02	2.39	adhesion molecule with Ig-like domain 2	Homo sapiens adhesion molecule with Ig-like domain 2 (AMIGO2), transcript variant 2, mRNA [NM_181847]
FERMT3	A_23_P64044	2.06E-02	3.54	fermitin family member 3	Homo sapiens fermitin family member 3 (FERMT3), transcript variant URP2LF, mRNA [NM_178443]
GATA4	A_23_P384761	2.06E-02	-2.11	GATA binding protein 4	Homo sapiens GATA binding protein 4 (GATA4), mRNA [NM_002052]
OR10G2	A_33_P3225507	2.06E-02	-4.18	olfactory receptor, family 10, subfamily G, member 2	Homo sapiens olfactory receptor, family 10, subfamily G, member 2 (OR10G2), mRNA [NM_001005466]
FLJ10661	A_33_P3421200	2.06E-02	2.23	family with sequence similarity 86, member A pseudogene	Homo sapiens family with sequence similarity 86, member A pseudogene (FLJ10661), transcript variant 2, non-coding RNA [NR_024361]
WWC1	A_23_P81392	2.06E-02	-3.39	WW and C2 domain containing 1	Homo sapiens WW and C2 domain containing 1 (WWC1), transcript variant 3, mRNA [NM_015238]
SLC27A2	A_23_P140450	2.06E-02	3.23	solute carrier family 27 (fatty acid transporter), member 2	Homo sapiens solute carrier family 27 (fatty acid transporter), member 2 (SLC27A2), transcript variant 1, mRNA [NM_003645]
C1S	A_23_P2492	2.06E-02	2.42	complement component 1, s subcomponent	Homo sapiens complement component 1, s subcomponent (C1S), transcript variant 2, mRNA [NM_001734]
ARHGEF16	A_23_P114670	2.07E-02	-2.75	Rho guanine nucleotide exchange factor (GEF) 16	Homo sapiens Rho guanine nucleotide exchange factor (GEF) 16 (ARHGEF16), mRNA [NM_014448]
CXCL11	A_23_P125278	2.07E-02	14.71	chemokine (C-X-C motif) ligand 11	Homo sapiens chemokine (C-X-C motif) ligand 11 (CXCL11), mRNA [NM_005409]
CNTNAP3B	A_33_P3321382	2.10E-02	-3.17	contactin associated protein-like 3B	contactin associated protein-like 3B [Source:HGNC Symbol;Acc:32035]

					[ENST00000341990]
DNAH1	A_23_P18213	2.11E-02	2.26	dynein, axonemal, heavy chain 1	Homo sapiens dynein, axonemal, heavy chain 1 (DNAH1), mRNA [NM_015512]
C18orf54	A_32_P162374	2.11E-02	2.69	chromosome 18 open reading frame 54	Homo sapiens chromosome 18 open reading frame 54 (C18orf54), mRNA [NM_173529]
TTC39B	A_23_P390097	2.12E-02	2.28	tetratricopeptide repeat domain 39B	Homo sapiens tetratricopeptide repeat domain 39B (TTC39B), transcript variant 1, mRNA [NM_152574]
COL5A1	A_33_P3629678	2.13E-02	2.23	collagen, type V, alpha 1	Homo sapiens collagen, type V, alpha 1 (COL5A1), mRNA [NM_000093]
TCP11L2	A_23_P419107	2.13E-02	-2.03	t-complex 11 (mouse)-like 2	Homo sapiens t-complex 11 (mouse)-like 2 (TCP11L2), mRNA [NM_152772]
PARP4	A_33_P3303259	2.14E-02	2.00	poly (ADP-ribose) polymerase family, member 4	Homo sapiens poly (ADP-ribose) polymerase family, member 4 (PARP4), mRNA [NM_006437]
SREBF1	A_23_P129786	2.14E-02	-2.08	sterol regulatory element binding transcription factor 1	Homo sapiens sterol regulatory element binding transcription factor 1 (SREBF1), transcript variant 1, mRNA [NM_001005291]
SCUBE2	A_23_P105144	2.14E-02	4.59	signal peptide, CUB domain, EGF-like 2	Homo sapiens signal peptide, CUB domain, EGF-like 2 (SCUBE2), transcript variant 1, mRNA [NM_020974]
IGFN1	A_33_P3382100	2.14E-02	3.79	immunoglobulin-like and fibronectin type III domain containing 1	Homo sapiens immunoglobulin-like and fibronectin type III domain containing 1 (IGFN1), mRNA [NM_001164586]
C1orf220	A_33_P3259865	2.14E-02	3.07	chromosome 1 open reading frame 220	Homo sapiens chromosome 1 open reading frame 220 (C1orf220), non-coding RNA [NR_033186]
ZNF501	A_33_P3257856	2.14E-02	2.69	zinc finger protein 501	Homo sapiens zinc finger protein 501 (ZNF501), mRNA [NM_145044]
CDC42	A_23_P300056	2.15E-02	-2.99	cell division cycle 42 (GTP binding protein, 25kDa)	Homo sapiens cell division cycle 42 (GTP binding protein, 25kDa) (CDC42),

					transcript variant 2, mRNA [NM_044472]
FNDC9	A_33_P3385656	2.16E-02	-2.08	fibronectin type III domain containing 9	Homo sapiens fibronectin type III domain containing 9 (FNDC9), mRNA [NM_001001343]
SCN9A	A_24_P3005	2.16E-02	2.73	sodium channel, voltage-gated, type IX, alpha subunit	Homo sapiens sodium channel, voltage-gated, type IX, alpha subunit (SCN9A), mRNA [NM_002977]
RBP5	A_24_P386746	2.16E-02	4.99	retinol binding protein 5, cellular	Homo sapiens retinol binding protein 5, cellular (RBP5), mRNA [NM_031491]
NCF2	A_23_P138194	2.16E-02	3.55	neutrophil cytosolic factor 2	Homo sapiens neutrophil cytosolic factor 2 (NCF2), transcript variant 1, mRNA [NM_000433]
FANK1	A_23_P115785	2.16E-02	2.01	fibronectin type III and ankyrin repeat domains 1	Homo sapiens fibronectin type III and ankyrin repeat domains 1 (FANK1), mRNA [NM_145235]
PRSS30P	A_23_P350719	2.16E-02	4.38	protease, serine, 30 homolog (mouse), pseudogene	Homo sapiens protease, serine, 30 homolog (mouse), pseudogene (PRSS30P), non-coding RNA [NR_026864]
GPT	A_33_P3301871	2.16E-02	-2.16	glutamic-pyruvate transaminase (alanine aminotransferase)	Homo sapiens glutamic-pyruvate transaminase (alanine aminotransferase) (GPT), mRNA [NM_005309]
CCDC85C	A_23_P37391	2.16E-02	-2.19	coiled-coil domain containing 85C	Homo sapiens coiled-coil domain containing 85C (CCDC85C), mRNA [NM_001144995]
DEFA7P	A_33_P3298173	2.17E-02	-2.05	defensin, alpha 7 pseudogene	Homo sapiens defensin alpha 7 (DEFA7P) pseudogene mRNA, complete sequence. [AY746432]
FST	A_23_P110531	2.17E-02	3.24	follistatin	Homo sapiens follistatin (FST), transcript variant FST344, mRNA [NM_013409]
ART5	A_23_P427122	2.17E-02	-2.89	ADP-ribosyltransferase 5	Homo sapiens ADP-ribosyltransferase 5 (ART5), transcript variant 1, mRNA [NM_053017]

TMEM63C	A_33_P3406661	2.17E-02	-3.06	transmembrane protein 63C	Homo sapiens transmembrane protein 63C (TMEM63C), mRNA [NM_020431]
LSP1	A_23_P13382	2.17E-02	3.06	lymphocyte-specific protein 1	Homo sapiens lymphocyte-specific protein 1 (LSP1), transcript variant 3, mRNA [NM_001013254]
HLA-DRB5	A_23_P45099	2.18E-02	4.16	major histocompatibility complex, class II, DR beta 5	Homo sapiens major histocompatibility complex, class II, DR beta 5 (HLA-DRB5), mRNA [NM_002125]
P2RX3	A_23_P127721	2.18E-02	-2.78	purinergic receptor P2X, ligand-gated ion channel, 3	Homo sapiens purinergic receptor P2X, ligand-gated ion channel, 3 (P2RX3), mRNA [NM_002559]
NPHP3	A_33_P3228102	2.18E-02	2.09	nephronophthisis 3 (adolescent)	Homo sapiens nephronophthisis 3 (adolescent) (NPHP3), mRNA [NM_153240]
LOC100129826	A_33_P3369245	2.19E-02	-2.03	uncharacterized LOC100129826	Homo sapiens cDNA FLJ44967 fis, clone BRAWH2016514. [AK126915]
PLEKHO1	A_33_P3330039	2.19E-02	-2.32	pleckstrin homology domain containing, family O member 1	Homo sapiens pleckstrin homology domain containing, family O member 1 (PLEKHO1), mRNA [NM_016274]
MSX1	A_23_P110430	2.19E-02	-2.74	msh homeobox 1	Homo sapiens msh homeobox 1 (MSX1), mRNA [NM_002448]
GPX3	A_33_P3369371	2.21E-02	-2.58	glutathione peroxidase 3 (plasma)	Homo sapiens glutathione peroxidase 3 (plasma) (GPX3), mRNA [NM_002084]
LONRF3	A_23_P114414	2.21E-02	-2.30	LON peptidase N-terminal domain and ring finger 3	Homo sapiens LON peptidase N-terminal domain and ring finger 3 (LONRF3), transcript variant 1, mRNA [NM_001031855]
NIPSNAP3B	A_33_P3271001	2.21E-02	-2.04	nipsnap homolog 3B (C. elegans)	Homo sapiens nipsnap homolog 3B (C. elegans) (NIPSNAP3B), mRNA [NM_018376]
IRF8	A_33_P3343120	2.21E-02	4.61	interferon regulatory factor 8	Homo sapiens interferon regulatory factor 8 (IRF8), mRNA [NM_002163]
CYP1B1-AS1	A_19_P00807643	2.22E-02	2.51		Homo sapiens CYP1B1 antisense RNA 1

					(non-protein coding) (CYP1B1-AS1), non-coding RNA [NR_027252]
ZNF594	A_23_P321160	2.22E-02	2.01	zinc finger protein 594	Homo sapiens zinc finger protein 594 (ZNF594), mRNA [NM_032530]
RASGRP3	A_24_P54390	2.22E-02	-2.18	RAS guanyl releasing protein 3 (calcium and DAG-regulated)	Homo sapiens RAS guanyl releasing protein 3 (calcium and DAG-regulated) (RASGRP3), transcript variant 2, mRNA [NM_170672]
C1QA	A_24_P222655	2.22E-02	4.35	complement component 1, q subcomponent, A chain	Homo sapiens complement component 1, q subcomponent, A chain (C1QA), mRNA [NM_015991]
TPD52	A_33_P3418838	2.24E-02	-2.42	tumor protein D52	tumor protein D52 [Source:HGNC Symbol;Acc:12005] [ENST00000523564]
EIF4EBP2	A_24_P115621	2.24E-02	-2.08	eukaryotic translation initiation factor 4E binding protein 2	Homo sapiens eukaryotic translation initiation factor 4E binding protein 2 (EIF4EBP2), mRNA [NM_004096]
FOSL1	A_23_P161624	2.24E-02	-3.06	FOS-like antigen 1	Homo sapiens FOS-like antigen 1 (FOSL1), mRNA [NM_005438]
ATXN2L	A_33_P3369956	2.24E-02	-2.30	ataxin 2-like	Homo sapiens ataxin 2-like (ATXN2L), transcript variant E, mRNA [NM_148416]
CPO	A_23_P102172	2.24E-02	-2.13	carboxypeptidase O	Homo sapiens carboxypeptidase O (CPO), mRNA [NM_173077]
HLA-J	A_24_P418044	2.25E-02	2.36	major histocompatibility complex, class I, J (pseudogene)	Homo sapiens major histocompatibility complex, class I, J (pseudogene) (HLA-J), non-coding RNA [NR_024240]
LMO1	A_23_P87310	2.25E-02	-3.18	LIM domain only 1 (rhombotin 1)	Homo sapiens LIM domain only 1 (rhombotin 1) (LMO1), mRNA [NM_002315]
TGM6	A_33_P3249259	2.26E-02	-3.33	transglutaminase 6	Homo sapiens transglutaminase 6 (TGM6), mRNA [NM_198994]
FNBP1	A_33_P3367565	2.27E-02	2.03	formin binding protein 1	formin binding protein 1 [Source:HGNC Symbol;Acc:17069] [ENST00000355681]
CENPE	A_23_P253524	2.27E-02	2.88	centromere protein E, 312kDa	Homo sapiens centromere protein E,

					312kDa (CENPE), mRNA [NM_001813]
PITPNM1	A_23_P1519	2.27E-02	-2.09	phosphatidylinositol transfer protein, membrane-associated 1	Homo sapiens phosphatidylinositol transfer protein, membrane-associated 1 (PITPNM1), transcript variant 1, mRNA [NM_004910]
ADCY7	A_24_P416177	2.27E-02	3.35	adenylate cyclase 7	Homo sapiens adenylate cyclase 7 (ADCY7), mRNA [NM_001114]
ANGPTL5	A_23_P64161	2.27E-02	5.09	angiopoietin-like 5	Homo sapiens angiopoietin-like 5 (ANGPTL5), mRNA [NM_178127]
RMI2	A_23_P353717	2.27E-02	3.57	RMI2, RecQ mediated genome instability 2, homolog (S. cerevisiae)	Homo sapiens RMI2, RecQ mediated genome instability 2, homolog (S. cerevisiae) (RMI2), mRNA [NM_152308]
CYCS	A_32_P174083	2.27E-02	-2.02	cytochrome c, somatic	Homo sapiens cytochrome c, somatic (CYCS), nuclear gene encoding mitochondrial protein, mRNA [NM_018947]
KRBA1	A_32_P38637	2.28E-02	-2.13	KRAB-A domain containing 1	Homo sapiens KRAB-A domain containing 1 (KRBA1), mRNA [NM_032534]
KRTAP3-1	A_33_P3382399	2.28E-02	-3.81	keratin associated protein 3-1	Homo sapiens keratin associated protein 3-1 (KRTAP3-1), mRNA [NM_031958]
C8orf39	A_23_P134729	2.28E-02	-2.36	chromosome 8 open reading frame 39	Homo sapiens chromosome 8 open reading frame 39 (C8orf39), non-coding RNA [NR_027259]
MNS1	A_23_P3302	2.28E-02	3.88	meiosis-specific nuclear structural 1	Homo sapiens meiosis-specific nuclear structural 1 (MNS1), mRNA [NM_018365]
METTL7A	A_23_P415021	2.29E-02	2.92	methyltransferase like 7A	Homo sapiens methyltransferase like 7A (METTL7A), mRNA [NM_014033]
ISLR	A_23_P3312	2.29E-02	3.47	immunoglobulin superfamily containing leucine-rich repeat	Homo sapiens immunoglobulin superfamily containing leucine-rich repeat (ISLR), transcript variant 1, mRNA [NM_005545]

C12orf34	A_23_P128375	2.30E-02	-3.60	chromosome 12 open reading frame 34	Homo sapiens chromosome 12 open reading frame 34 (C12orf34), mRNA [NM_032829]
TMOD4	A_23_P126605	2.30E-02	3.10	tropomodulin 4 (muscle)	Homo sapiens tropomodulin 4 (muscle) (TMOD4), mRNA [NM_013353]
KMO	A_24_P77082	2.30E-02	3.04	kynurenine 3-monooxygenase (kynurenine 3-hydroxylase)	Homo sapiens kynurenine 3-monooxygenase (kynurenine 3-hydroxylase) (KMO), mRNA [NM_003679]
COL1A2	A_24_P277934	2.30E-02	2.88	collagen, type I, alpha 2	Homo sapiens collagen, type I, alpha 2 (COL1A2), mRNA [NM_000089]
WNT3A	A_33_P3417502	2.30E-02	-2.56	wingless-type MMTV integration site family, member 3A	Homo sapiens wingless-type MMTV integration site family, member 3A (WNT3A), mRNA [NM_033131]
CACNA2D4	A_23_P353014	2.30E-02	2.13	calcium channel, voltage-dependent, alpha 2/delta subunit 4	Homo sapiens calcium channel, voltage-dependent, alpha 2/delta subunit 4 (CACNA2D4), mRNA [NM_172364]
STK10	A_33_P3389153	2.30E-02	2.08	serine/threonine kinase 10	Homo sapiens serine/threonine kinase 10 (STK10), mRNA [NM_005990]
KCNN2	A_23_P500353	2.30E-02	-3.23	potassium intermediate/small conductance calcium-activated channel, subfamily N, member 2	Homo sapiens potassium intermediate/small conductance calcium-activated channel, subfamily N, member 2 (KCNN2), transcript variant 1, mRNA [NM_021614]
MLF1IP	A_23_P254733	2.31E-02	3.23	MLF1 interacting protein	Homo sapiens MLF1 interacting protein (MLF1IP), mRNA [NM_024629]
HELLS	A_23_P12816	2.32E-02	3.33	helicase, lymphoid-specific	Homo sapiens helicase, lymphoid-specific (HELLS), mRNA [NM_018063]
GALNT12	A_23_P415652	2.33E-02	2.87	UDP-N-acetyl-alpha-D-galactosamine:polypeptide N-acetylgalactosaminyltransferase 12 (GalNAc-T12)	Homo sapiens UDP-N-acetyl-alpha-D-galactosamine:polypeptide N-acetylgalactosaminyltransferase 12 (GalNAc-T12) (GALNT12), mRNA [NM_024642]

TPSD1	A_23_P140716	2.33E-02	2.08	tryptase delta 1	Homo sapiens tryptase delta 1 (TPSD1), mRNA [NM_012217]
LOC100128946	A_33_P3384502	2.33E-02	-2.41	uncharacterized LOC100128946	Homo sapiens uncharacterized LOC100128946 (LOC100128946), non-coding RNA [NR_038944]
TMEM38A	A_23_P101392	2.34E-02	-2.30	transmembrane protein 38A	Homo sapiens transmembrane protein 38A (TMEM38A), mRNA [NM_024074]
CD33	A_23_P331748	2.35E-02	3.81	CD33 molecule	Homo sapiens CD33 molecule (CD33), transcript variant 1, mRNA [NM_001772]
CAPN12	A_24_P79040	2.35E-02	3.39	calpain 12	Homo sapiens calpain 12 (CAPN12), mRNA [NM_144691]
KRTAP12-1	A_24_P66312	2.35E-02	-2.32	keratin associated protein 12-1	Homo sapiens keratin associated protein 12-1 (KRTAP12-1), mRNA [NM_181686]
IL5RA	A_33_P3328254	2.36E-02	4.92	interleukin 5 receptor, alpha	Homo sapiens interleukin 5 receptor, alpha (IL5RA), transcript variant 3, mRNA [NM_175725]
ZNF599	A_24_P353103	2.36E-02	2.32	zinc finger protein 599	Homo sapiens zinc finger protein 599 (ZNF599), mRNA [NM_001007248]
SVEP1	A_23_P216596	2.37E-02	2.73	sushi, von Willebrand factor type A, EGF and pentraxin domain containing 1	Homo sapiens sushi, von Willebrand factor type A, EGF and pentraxin domain containing 1 (SVEP1), mRNA [NM_153366]
VIPR2	A_23_P145978	2.38E-02	-2.02	vasoactive intestinal peptide receptor 2	Homo sapiens vasoactive intestinal peptide receptor 2 (VIPR2), mRNA [NM_003382]
GZMM	A_23_P130836	2.38E-02	2.42	granzyme M (lymphocyte met-ase 1)	Homo sapiens granzyme M (lymphocyte met-ase 1) (GZMM), mRNA [NM_005317]
CD74	A_23_P70095	2.38E-02	3.16	CD74 molecule, major histocompatibility complex, class II invariant chain	Homo sapiens CD74 molecule, major histocompatibility complex, class II invariant chain (CD74), transcript variant 3, mRNA [NM_001025158]
KIF11	A_24_P227091	2.38E-02	2.56	kinesin family member 11	Homo sapiens kinesin family member 11

					(KIF11), mRNA [NM_004523]
B4GALT6	A_24_P228228	2.38E-02	-2.11	UDP-Gal:betaGlcNAc beta 1,4-galactosyltransferase, polypeptide 6	Homo sapiens UDP-Gal:betaGlcNAc beta 1,4- galactosyltransferase, polypeptide 6 (B4GALT6), mRNA [NM_004775]
ZCCHC5	A_23_P411353	2.38E-02	3.75	zinc finger, CCHC domain containing 5	Homo sapiens zinc finger, CCHC domain containing 5 (ZCCHC5), mRNA [NM_152694]
SLC25A45	A_23_P422511	2.38E-02	2.18	solute carrier family 25, member 45	Homo sapiens solute carrier family 25, member 45 (SLC25A45), transcript variant 1, mRNA [NM_182556]
RINL	A_33_P3407895	2.39E-02	2.07	Ras and Rab interactor-like	Homo sapiens Ras and Rab interactor-like (RINL), transcript variant 1, mRNA [NM_001195833]
GLB1L	A_23_P218579	2.39E-02	2.14	galactosidase, beta 1-like	Homo sapiens galactosidase, beta 1-like (GLB1L), mRNA [NM_024506]
NOTCH2	A_33_P3258824	2.41E-02	2.30	notch 2	Homo sapiens notch 2 (NOTCH2), transcript variant 2, mRNA [NM_001200001]
KIAA1257	A_32_P163739	2.41E-02	2.08	KIAA1257	Homo sapiens KIAA1257 (KIAA1257), mRNA [NM_020741]
F2RL2	A_32_P60065	2.42E-02	5.19	coagulation factor II (thrombin) receptor-like 2	Homo sapiens coagulation factor II (thrombin) receptor-like 2 (F2RL2), mRNA [NM_004101]
PARD6B	A_33_P3227457	2.42E-02	-2.45	par-6 partitioning defective 6 homolog beta (C. elegans)	par-6 partitioning defective 6 homolog beta (C. elegans) [Source:HGNC Symbol;Acc:16245] [ENST00000396039]
MELK	A_23_P94422	2.42E-02	2.26	maternal embryonic leucine zipper kinase	Homo sapiens maternal embryonic leucine zipper kinase (MELK), mRNA [NM_014791]
ATL1	A_23_P88351	2.42E-02	2.05	atlastin GTPase 1	Homo sapiens atlastin GTPase 1 (ATL1), transcript variant 2, mRNA [NM_181598]
RANBP17	A_23_P58819	2.43E-02	3.35	RAN binding protein 17	Homo sapiens RAN binding protein 17 (RANBP17), mRNA [NM_022897]

HCG26	A_32_P220770	2.44E-02	3.66	HLA complex group 26 (non-protein coding)	Homo sapiens HLA complex group 26 (non-protein coding) (HCG26), non-coding RNA [NR_002812]
SLFN12L	A_33_P3261468	2.44E-02	2.33	schlafen family member 12-like	Homo sapiens schlafen family member 12-like (SLFN12L), mRNA [NM_001195790]
CCL14	A_23_P218369	2.45E-02	4.11	chemokine (C-C motif) ligand 14	Homo sapiens chemokine (C-C motif) ligand 14 (CCL14), transcript variant 3, mRNA [NM_032963]
XAF1	A_33_P3258346	2.45E-02	2.49	XIAP associated factor 1	Homo sapiens XIAP associated factor 1 (XAF1), transcript variant 1, mRNA [NM_017523]
PROSER1	A_33_P3398533	2.45E-02	-2.18	proline and serine rich 1	Homo sapiens proline and serine rich 1 (PROSER1), transcript variant 1, mRNA [NM_025138]
GRID2IP	A_33_P3411097	2.45E-02	-4.44	glutamate receptor, ionotropic, delta 2 (Grid2) interacting protein	Homo sapiens glutamate receptor, ionotropic, delta 2 (Grid2) interacting protein (GRID2IP), mRNA [NM_001145118]
JSRP1	A_23_P397320	2.45E-02	10.87	junctional sarcoplasmic reticulum protein 1	Homo sapiens junctional sarcoplasmic reticulum protein 1 (JSRP1), mRNA [NM_144616]
CATSPER1	A_23_P52676	2.45E-02	3.02	cation channel, sperm associated 1	Homo sapiens cation channel, sperm associated 1 (CATSPER1), mRNA [NM_053054]
MGC34800	A_33_P3373243	2.45E-02	2.02	uncharacterized protein MGC34800	PREDICTED: Homo sapiens hypothetical protein MGC34800 (MGC34800), miscRNA [XR_110135]
DYRK3	A_24_P345209	2.45E-02	-2.14	dual-specificity tyrosine-(Y)-phosphorylation regulated kinase 3	Homo sapiens dual-specificity tyrosine-(Y)-phosphorylation regulated kinase 3 (DYRK3), transcript variant 2, mRNA [NM_001004023]
C7orf63	A_33_P3615922	2.46E-02	2.61	chromosome 7 open reading	Homo sapiens chromosome 7 open

				frame 63	reading frame 63 (C7orf63), transcript variant 1, mRNA [NM_001039706]
CD160	A_23_P354341	2.47E-02	3.42	CD160 molecule	Homo sapiens CD160 molecule (CD160), mRNA [NM_007053]
SEL1L3	A_23_P426021	2.47E-02	3.67	sel-1 suppressor of lin-12-like 3 (C. elegans)	Homo sapiens sel-1 suppressor of lin-12-like 3 (C. elegans) (SEL1L3), mRNA [NM_015187]
RBM43	A_32_P116660	2.47E-02	2.46	RNA binding motif protein 43	Homo sapiens RNA binding motif protein 43 (RBM43), mRNA [NM_198557]
IRAK3	A_23_P162300	2.47E-02	2.12	interleukin-1 receptor-associated kinase 3	Homo sapiens interleukin-1 receptor-associated kinase 3 (IRAK3), transcript variant 1, mRNA [NM_007199]
SLCO5A1	A_23_P135669	2.49E-02	-3.45	solute carrier organic anion transporter family, member 5A1	Homo sapiens solute carrier organic anion transporter family, member 5A1 (SLCO5A1), transcript variant 1, mRNA [NM_030958]
CFB	A_23_P156687	2.49E-02	2.85	complement factor B	Homo sapiens complement factor B (CFB), mRNA [NM_001710]
APOA1	A_23_P203191	2.50E-02	4.56	apolipoprotein A-I	Homo sapiens apolipoprotein A-I (APOA1), mRNA [NM_000039]
APBB3	A_23_P110445	2.50E-02	2.01	amyloid beta (A4) precursor protein-binding, family B, member 3	Homo sapiens amyloid beta (A4) precursor protein-binding, family B, member 3 (APBB3), transcript variant 4, mRNA [NM_006051]
OBFC2A	A_24_P229531	2.51E-02	3.60	oligonucleotide/oligosaccharide-binding fold containing 2A	Homo sapiens oligonucleotide/oligosaccharide-binding fold containing 2A (OBFC2A), transcript variant 1, mRNA [NM_001031716]
CILP	A_23_P151895	2.52E-02	3.69	cartilage intermediate layer protein, nucleotide pyrophosphohydrolase	Homo sapiens cartilage intermediate layer protein, nucleotide pyrophosphohydrolase (CILP), mRNA [NM_003613]
LINC00346	A_23_P368909	2.53E-02	-2.24	long intergenic non-protein	Homo sapiens long intergenic non-

				coding RNA 346	protein coding RNA 346 (LINC00346), non-coding RNA [NR_027701]
HLA-DQB1	A_23_P8108	2.54E-02	3.78	major histocompatibility complex, class II, DQ beta 1	Homo sapiens major histocompatibility complex, class II, DQ beta 1 (HLA-DQB1), transcript variant 3, mRNA [NM_001243962]
RAB38	A_23_P161563	2.54E-02	3.10	RAB38, member RAS oncogene family	Homo sapiens RAB38, member RAS oncogene family (RAB38), mRNA [NM_022337]
LOC440149	A_33_P3573141	2.55E-02	3.72	uncharacterized LOC440149	Homo sapiens cDNA FLJ32523 fis, clone SMINT2000032. [AK057085]
TNFAIP8L2	A_23_P46356	2.55E-02	4.12	tumor necrosis factor, alpha-induced protein 8-like 2	Homo sapiens tumor necrosis factor, alpha-induced protein 8-like 2 (TNFAIP8L2), mRNA [NM_024575]
LOC728503	A_33_P3345931	2.58E-02	-3.01	uncharacterized LOC728503	PREDICTED: Homo sapiens hypothetical protein LOC728503 (LOC728503), mRNA [XM_001127575]
NFAM1	A_33_P3292769	2.58E-02	4.30	NFAT activating protein with ITAM motif 1	Homo sapiens NFAT activating protein with ITAM motif 1 (NFAM1), mRNA [NM_145912]
PTH1R	A_33_P3365368	2.61E-02	-2.45	parathyroid hormone 1 receptor	parathyroid hormone 1 receptor [Source:HGNC Symbol;Acc:9608] [ENST00000313063]
XLOC_009868	A_19_P00322036	2.63E-02	-3.56		BROAD Institute lincRNA (XLOC_009868), lincRNA [TCONS_00020555]
LOC642980	A_33_P3256880	2.63E-02	-2.59	uncharacterized LOC642980	Homo sapiens cDNA FLJ16523 fis, clone NT2RP8000521. [AK131413]
NAPSA	A_23_P90130	2.65E-02	2.79	napsin A aspartic peptidase	Homo sapiens napsin A aspartic peptidase (NAPSA), mRNA [NM_004851]
HAUS5	A_23_P349771	2.65E-02	2.81	HAUS augmin-like complex, subunit 5	Homo sapiens HAUS augmin-like complex, subunit 5 (HAUS5), mRNA [NM_015302]

RHEB	A_33_P3413083	2.65E-02	-2.09	Ras homolog enriched in brain	Homo sapiens Ras homolog enriched in brain (RHEB), mRNA [NM_005614]
LOC100129399	A_33_P3403937	2.66E-02	3.09	uncharacterized LOC100129399	Homo sapiens cDNA FLJ42017 fis, clone SPLEN2033153. [AK124011]
PLK2	A_23_P30254	2.66E-02	-2.32	polo-like kinase 2	Homo sapiens polo-like kinase 2 (PLK2), transcript variant 1, mRNA [NM_006622]
APOBEC3G	A_23_P143713	2.66E-02	2.95	apolipoprotein B mRNA editing enzyme, catalytic polypeptide-like 3G	Homo sapiens apolipoprotein B mRNA editing enzyme, catalytic polypeptide-like 3G (APOBEC3G), mRNA [NM_021822]
MAFB	A_23_P17345	2.66E-02	3.07	v-maf musculoaponeurotic fibrosarcoma oncogene homolog B (avian)	Homo sapiens v-maf musculoaponeurotic fibrosarcoma oncogene homolog B (avian) (MAFB), mRNA [NM_005461]
FCRL3	A_33_P3368334	2.67E-02	8.90	Fc receptor-like 3	Fc receptor-like 3 [Source:HGNC Symbol;Acc:18506] [ENST00000368186]
VAMP8	A_23_P28434	2.67E-02	3.00	vesicle-associated membrane protein 8 (endobrevin)	Homo sapiens vesicle-associated membrane protein 8 (endobrevin) (VAMP8), mRNA [NM_003761]
GLT25D2	A_24_P62505	2.69E-02	3.04	glycosyltransferase 25 domain containing 2	Homo sapiens glycosyltransferase 25 domain containing 2 (GLT25D2), mRNA [NM_015101]
C5orf56	A_33_P3388983	2.71E-02	2.02	chromosome 5 open reading frame 56	Homo sapiens cDNA: FLJ21568 fis, clone COL06492. [AK025221]
PCYOX1L	A_23_P30275	2.72E-02	2.49	prenylcysteine oxidase 1 like	Homo sapiens prenylcysteine oxidase 1 like (PCYOX1L), mRNA [NM_024028]
E2F2	A_23_P408955	2.72E-02	4.28	E2F transcription factor 2	Homo sapiens E2F transcription factor 2 (E2F2), mRNA [NM_004091]
ARSF	A_23_P304304	2.72E-02	-3.04	arylsulfatase F	Homo sapiens arylsulfatase F (ARSF), transcript variant 1, mRNA [NM_004042]
AKT1S1	A_33_P3222451	2.74E-02	2.48	AKT1 substrate 1 (proline-rich)	AKT1 substrate 1 (proline-rich) [Source:HGNC Symbol;Acc:28426] [ENST00000391830]

CHEK2	A_23_P109452	2.74E-02	-2.28	checkpoint kinase 2	Homo sapiens CHK2 checkpoint homolog (S. pombe) (CHEK2), transcript variant 3, mRNA [NM_001005735]
MPRIP	A_33_P3230528	2.74E-02	-2.01	myosin phosphatase Rho interacting protein	myosin phosphatase Rho interacting protein [Source:HGNC Symbol;Acc:30321] [ENST00000395807]
FAM21C	A_33_P3423240	2.77E-02	3.12	family with sequence similarity 21, member C	family with sequence similarity 21, member C [Source:HGNC Symbol;Acc:23414] [ENST00000374359]
FLJ40606	A_33_P3303031	2.78E-02	-2.03	uncharacterized LOC643549	Homo sapiens hypothetical protein LOC643549, mRNA (cDNA clone IMAGE:40147028). [BC133006]
SPAG4	A_23_P132027	2.78E-02	2.70	sperm associated antigen 4	Homo sapiens sperm associated antigen 4 (SPAG4), mRNA [NM_003116]
LOC145837	A_32_P46594	2.79E-02	2.80	uncharacterized LOC145837	Homo sapiens uncharacterized LOC145837 (LOC145837), non-coding RNA [NR_026979]
POSTN	A_33_P3511265	2.79E-02	6.29	periostin, osteoblast specific factor	Homo sapiens periostin, osteoblast specific factor (POSTN), transcript variant 1, mRNA [NM_006475]
AKAP10	A_23_P317105	2.80E-02	2.06	A kinase (PRKA) anchor protein 10	Homo sapiens A kinase (PRKA) anchor protein 10 (AKAP10), nuclear gene encoding mitochondrial protein, mRNA [NM_007202]
ACOXL	A_33_P3395008	2.81E-02	5.02	acyl-CoA oxidase-like	Homo sapiens acyl-CoA oxidase-like (ACOXL), mRNA [NM_001142807]
CMA1	A_23_P151778	2.81E-02	2.45	chymase 1, mast cell	Homo sapiens chymase 1, mast cell (CMA1), mRNA [NM_001836]
THY1	A_33_P3280845	2.81E-02	6.81	Thy-1 cell surface antigen	Homo sapiens Thy-1 cell surface antigen (THY1), mRNA [NM_006288]
C20orf103	A_23_P40295	2.81E-02	6.34	chromosome 20 open reading frame 103	Homo sapiens chromosome 20 open reading frame 103 (C20orf103), transcript variant 1, mRNA [NM_012261]

RAB44	A_33_P3349912	2.82E-02	2.79	RAB44, member RAS oncogene family	Homo sapiens cDNA FLJ43093 fis, clone CORDB1000140. [AK125083]
XLOC_l2_013314	A_19_P00318390	2.82E-02	-2.14		BROAD Institute lincRNA (XLOC_l2_013314), lincRNA [TCONS_l2_00027075]
LOC100131831	A_33_P3243652	2.84E-02	-2.42	uncharacterized LOC100131831	Homo sapiens cDNA FLJ26174 fis, clone ADG03920. [AK129685]
CDX1	A_33_P3289848	2.84E-02	-2.14	caudal type homeobox 1	Homo sapiens caudal type homeobox 1 (CDX1), mRNA [NM_001804]
SLAMF1	A_33_P3352827	2.85E-02	4.95	signaling lymphocytic activation molecule family member 1	Homo sapiens signaling lymphocytic activation molecule family member 1 (SLAMF1), mRNA [NM_003037]
C1QB	A_23_P137366	2.85E-02	4.05	complement component 1, q subcomponent, B chain	Homo sapiens complement component 1, q subcomponent, B chain (C1QB), mRNA [NM_000491]
FLJ23867	A_33_P3220475	2.85E-02	-2.93	uncharacterized protein FLJ23867	Homo sapiens uncharacterized protein FLJ23867 (FLJ23867), non-coding RNA [NR_026900]
KIAA1217	A_33_P3233580	2.85E-02	-2.07	KIAA1217	Homo sapiens cDNA FLJ43687 fis, clone TBAES2002197. [AK125675]
ZNF441	A_33_P3397323	2.85E-02	2.05	zinc finger protein 441	Homo sapiens zinc finger protein 441 (ZNF441), mRNA [NM_152355]
GRM1	A_23_P30976	2.85E-02	8.52	glutamate receptor, metabotropic 1	Homo sapiens glutamate receptor, metabotropic 1 (GRM1), transcript variant 1, mRNA [NM_000838]
ZNF154	A_33_P3233754	2.85E-02	-2.04	zinc finger protein 154	Homo sapiens zinc finger protein 154 (ZNF154), mRNA [NM_001085384]
TYROBP	A_33_P3409062	2.85E-02	3.21	TYRO protein tyrosine kinase binding protein	Homo sapiens TYRO protein tyrosine kinase binding protein (TYROBP), transcript variant 1, mRNA [NM_003332]
PDIA2	A_23_P325642	2.86E-02	2.64	protein disulfide isomerase family A, member 2	Homo sapiens protein disulfide isomerase family A, member 2 (PDIA2), mRNA [NM_006849]

ADARB2	A_23_P86751	2.86E-02	-3.02	adenosine deaminase, RNA-specific, B2	Homo sapiens adenosine deaminase, RNA-specific, B2 (ADARB2), mRNA [NM_018702]
C11orf80	A_23_P96165	2.87E-02	3.00	chromosome 11 open reading frame 80	Homo sapiens chromosome 11 open reading frame 80 (C11orf80), mRNA [NM_024650]
LOC113230	A_32_P52330	2.87E-02	-2.70	uncharacterized LOC113230	Homo sapiens uncharacterized LOC113230 (LOC113230), non-coding RNA [NR_024282]
C1orf110	A_24_P6370	2.87E-02	2.66	chromosome 1 open reading frame 110	Homo sapiens chromosome 1 open reading frame 110 (C1orf110), mRNA [NM_178550]
PTCRA	A_33_P3229918	2.87E-02	3.13	pre T-cell antigen receptor alpha	Homo sapiens pre T-cell antigen receptor alpha (PTCRA), transcript variant 1, mRNA [NM_001243168]
OR5K3	A_33_P3419557	2.88E-02	-2.46	olfactory receptor, family 5, subfamily K, member 3	Homo sapiens olfactory receptor, family 5, subfamily K, member 3 (OR5K3), mRNA [NM_001005516]
NAP1L3	A_23_P125717	2.88E-02	8.66	nucleosome assembly protein 1-like 3	Homo sapiens nucleosome assembly protein 1-like 3 (NAP1L3), mRNA [NM_004538]
SAMD9L	A_33_P3264846	2.88E-02	2.98	sterile alpha motif domain containing 9-like	Homo sapiens sterile alpha motif domain containing 9-like (SAMD9L), mRNA [NM_152703]
IL4	A_23_P213706	2.90E-02	2.34	interleukin 4	Homo sapiens interleukin 4 (IL4), transcript variant 1, mRNA [NM_000589]
KIAA0895	A_23_P305205	2.90E-02	-2.28	KIAA0895	Homo sapiens KIAA0895 (KIAA0895), transcript variant 2, mRNA [NM_015314]
RRP12	A_23_P24192	2.91E-02	-2.00	ribosomal RNA processing 12 homolog (S. cerevisiae)	Homo sapiens ribosomal RNA processing 12 homolog (S. cerevisiae) (RRP12), transcript variant 1, mRNA [NM_015179]
A2LD1	A_32_P110872	2.92E-02	2.17	AIG2-like domain 1	Homo sapiens AIG2-like domain 1 (A2LD1), transcript variant 1, mRNA

					[NM_033110]
TNFSF13	A_23_P152620	2.93E-02	2.19	tumor necrosis factor (ligand) superfamily, member 13	Homo sapiens tumor necrosis factor (ligand) superfamily, member 13 (TNFSF13), transcript variant gamma, mRNA [NM_172088]
EML4	A_24_P273413	2.93E-02	2.29	echinoderm microtubule associated protein like 4	Homo sapiens echinoderm microtubule associated protein like 4 (EML4), transcript variant 1, mRNA [NM_019063]
ESPNL	A_24_P170983	2.93E-02	5.78	espin-like	Homo sapiens espin-like (ESPNL), mRNA [NM_194312]
FBP1	A_23_P257111	2.93E-02	4.41	fructose-1,6-bisphosphatase 1	Homo sapiens fructose-1,6-bisphosphatase 1 (FBP1), transcript variant 1, mRNA [NM_000507]
FAM19A2	A_24_P297551	2.93E-02	2.22	family with sequence similarity 19 (chemokine (C-C motif)-like), member A2	Homo sapiens family with sequence similarity 19 (chemokine (C-C motif)-like), member A2 (FAM19A2), mRNA [NM_178539]
RNU2-2	A_33_P3279708	2.93E-02	2.27	RNA, U2 small nuclear 2	Homo sapiens RNA, U2 small nuclear 2 (RNU2-2), small nuclear RNA [NR_002761]
GGN	A_33_P3329984	2.94E-02	-2.01	gametogenetin	Homo sapiens gametogenetin (GGN), mRNA [NM_152657]
SH3TC1	A_23_P41390	2.94E-02	2.65	SH3 domain and tetratricopeptide repeats 1	Homo sapiens SH3 domain and tetratricopeptide repeats 1 (SH3TC1), mRNA [NM_018986]
ZACN	A_33_P3413394	2.95E-02	-2.06	zinc activated ligand-gated ion channel	zinc activated ligand-gated ion channel [Source:HGNC Symbol;Acc:29504] [ENST00000392503]
UAP1	A_23_P160460	2.95E-02	-2.11	UDP-N-acteylglucosamine pyrophosphorylase 1	Homo sapiens UDP-N-acteylglucosamine pyrophosphorylase 1 (UAP1), mRNA [NM_003115]
UTS2D	A_32_P225659	2.95E-02	2.53	urotensin 2 domain containing	Homo sapiens urotensin 2 domain containing (UTS2D), mRNA

					[NM_198152]
OCM2	A_32_P145867	2.95E-02	-3.04	oncomodulin 2	Homo sapiens oncomodulin 2 (OCM2), mRNA [NM_006188]
TNFSF8	A_23_P169257	2.96E-02	2.66	tumor necrosis factor (ligand) superfamily, member 8	Homo sapiens tumor necrosis factor (ligand) superfamily, member 8 (TNFSF8), transcript variant 1, mRNA [NM_001244]
LOC389634	A_33_P3243502	2.96E-02	2.37	uncharacterized LOC389634	Homo sapiens uncharacterized LOC389634 (LOC389634), non-coding RNA [NR_024420]
ALOX5AP	A_24_P347378	2.96E-02	4.65	arachidonate 5-lipoxygenase-activating protein	Homo sapiens arachidonate 5-lipoxygenase-activating protein (ALOX5AP), transcript variant 1, mRNA [NM_001629]
RNF126	A_33_P3270102	2.96E-02	-2.20	ring finger protein 126	Homo sapiens ring finger protein 126 (RNF126), mRNA [NM_194460]
STK17B	A_33_P3342957	2.96E-02	3.00	serine/threonine kinase 17b	Homo sapiens serine/threonine kinase 17b (STK17B), mRNA [NM_004226]
MGC16025	A_23_P79572	2.97E-02	5.37	uncharacterized LOC85009	Homo sapiens uncharacterized LOC85009 (MGC16025), non-coding RNA [NR_026664]
OSBPL6	A_33_P3255194	2.98E-02	-2.61	oxysterol binding protein-like 6	Homo sapiens oxysterol binding protein-like 6 (OSBPL6), transcript variant 5, mRNA [NM_001201482]
LSAMP	A_23_P301855	3.00E-02	-5.31	limbic system-associated membrane protein	Homo sapiens limbic system-associated membrane protein (LSAMP), mRNA [NM_002338]
TOX	A_24_P226755	3.00E-02	3.26	thymocyte selection-associated high mobility group box	Homo sapiens thymocyte selection-associated high mobility group box (TOX), mRNA [NM_014729]
CIRBP	A_23_P377616	3.00E-02	2.20	cold inducible RNA binding protein	Homo sapiens cold inducible RNA binding protein (CIRBP), transcript variant 2, non-coding RNA [NR_023312]

LOC100288069	A_33_P3232624	3.00E-02	2.10	general transcription factor IIi pseudogene	Homo sapiens general transcription factor IIi pseudogene (LOC100288069), non-coding RNA [NR_033908]
PLEK	A_23_P209678	3.01E-02	4.19	pleckstrin	Homo sapiens pleckstrin (PLEK), mRNA [NM_002664]
ALOX15B	A_23_P60627	3.01E-02	4.06	arachidonate 15-lipoxygenase, type B	Homo sapiens arachidonate 15-lipoxygenase, type B (ALOX15B), transcript variant d, mRNA [NM_001141]
LOC284630	A_33_P3655775	3.02E-02	2.59	uncharacterized LOC284630	Homo sapiens cDNA FLJ39065 fis, clone NT2RP7014721. [AK096384]
IGSF21	A_32_P78101	3.05E-02	3.84	immunoglobin superfamily, member 21	Homo sapiens immunoglobin superfamily, member 21 (IGSF21), mRNA [NM_032880]
CXCR2	A_33_P3214550	3.05E-02	2.40	chemokine (C-X-C motif) receptor 2	Homo sapiens chemokine (C-X-C motif) receptor 2 (CXCR2), transcript variant 1, mRNA [NM_001557]
LUM	A_23_P99063	3.06E-02	4.47	lumican	Homo sapiens lumican (LUM), mRNA [NM_002345]
NAV1	A_24_P102880	3.06E-02	2.27	neuron navigator 1	Homo sapiens neuron navigator 1 (NAV1), transcript variant 1, mRNA [NM_020443]
FAM83G	A_33_P3335386	3.06E-02	-3.14	family with sequence similarity 83, member G	family with sequence similarity 83, member G [Source:HGNC Symbol;Acc:32554] [ENST00000388995]
FOXD1	A_32_P34920	3.06E-02	5.25	forkhead box D1	Homo sapiens forkhead box D1 (FOXD1), mRNA [NM_004472]
PARP10	A_33_P3398448	3.06E-02	2.17	poly (ADP-ribose) polymerase family, member 10	Homo sapiens poly (ADP-ribose) polymerase family, member 10 (PARP10), mRNA [NM_032789]
RAD21-AS1	A_33_P3276323	3.06E-02	2.17	RAD21 antisense RNA 1 (non-protein coding)	Homo sapiens RAD21 antisense RNA 1 (non-protein coding) (RAD21-AS1), non-coding RNA [NR_033886]
LOC392196	A_33_P3338491	3.08E-02	-2.37	ubiquitin carboxyl-terminal	Homo sapiens ubiquitin carboxyl-

				hydrolase 17-like pseudogene	terminal hydrolase 17-like pseudogene (LOC392196), non-coding RNA [NR_003275]
ENO3	A_23_P130149	3.09E-02	-2.15	enolase 3 (beta, muscle)	Homo sapiens enolase 3 (beta, muscle) (ENO3), transcript variant 1, mRNA [NM_001976]
SFMBT2	A_33_P3387455	3.10E-02	2.01	Scm-like with four mbt domains 2	Homo sapiens Scm-like with four mbt domains 2 (SFMBT2), transcript variant 1, mRNA [NM_001029880]
BRCA2	A_23_P99452	3.10E-02	2.20	breast cancer 2, early onset	Homo sapiens breast cancer 2, early onset (BRCA2), mRNA [NM_000059]
HLA-DPA1	A_33_P3234277	3.10E-02	3.21	major histocompatibility complex, class II, DP alpha 1	Homo sapiens major histocompatibility complex, class II, DP alpha 1 (HLA-DPA1), transcript variant 2, mRNA [NM_001242524]
SHBG	A_33_P3353116	3.10E-02	-4.11	sex hormone-binding globulin	Homo sapiens sex hormone-binding globulin (SHBG), transcript variant 5, non-coding RNA [NR_027462]
MN1	A_23_P6381	3.10E-02	2.19	meningioma (disrupted in balanced translocation) 1	Homo sapiens meningioma (disrupted in balanced translocation) 1 (MN1), mRNA [NM_002430]
TMEM154	A_23_P361584	3.10E-02	2.19	transmembrane protein 154	Homo sapiens transmembrane protein 154 (TMEM154), mRNA [NM_152680]
GFI1B	A_23_P216845	3.11E-02	-2.30	growth factor independent 1B transcription repressor	Homo sapiens growth factor independent 1B transcription repressor (GFI1B), transcript variant 1, mRNA [NM_004188]
KIAA1958	A_33_P3314341	3.11E-02	-2.56	KIAA1958	KIAA1958 [Source:HGNC Symbol;Acc:23427] [ENST00000374244]
DMXL2	A_23_P327361	3.11E-02	2.37	Dmx-like 2	Homo sapiens Dmx-like 2 (DMXL2), transcript variant 2, mRNA [NM_015263]
DOK3	A_33_P3253144	3.12E-02	2.96	docking protein 3	Homo sapiens docking protein 3 (DOK3), transcript variant 1, mRNA [NM_024872]

HIST1H2AI	A_33_P3360216	3.12E-02	2.88	histone cluster 1, H2ai	Homo sapiens histone cluster 1, H2ai (HIST1H2AI), mRNA [NM_003509]
LOC151657	A_33_P3436646	3.13E-02	3.20	uncharacterized LOC151657	Homo sapiens cDNA FLJ33795 fis, clone CTONG1000097. [AK091114]
MS4A7	A_33_P3352098	3.15E-02	2.80	membrane-spanning 4-domains, subfamily A, member 7	Homo sapiens membrane-spanning 4-domains, subfamily A, member 7 (MS4A7), transcript variant 1, mRNA [NM_021201]
GIMAP2	A_23_P368681	3.17E-02	2.76	GTPase, IMAP family member 2	Homo sapiens GTPase, IMAP family member 2 (GIMAP2), mRNA [NM_015660]
FCHO1	A_33_P3255509	3.17E-02	2.46	FCH domain only 1	Homo sapiens FCH domain only 1 (FCHO1), transcript variant 1, mRNA [NM_001161357]
RASEF	A_32_P231568	3.17E-02	2.34	RAS and EF-hand domain containing	Homo sapiens RAS and EF-hand domain containing (RASEF), mRNA [NM_152573]
XLOC_l2_003666	A_19_P00802587	3.17E-02	-3.97		BROAD Institute lincRNA (XLOC_l2_003666), lincRNA [TCONS_l2_00007378]
FLT3LG	A_23_P78742	3.19E-02	2.00	fms-related tyrosine kinase 3 ligand	Homo sapiens fms-related tyrosine kinase 3 ligand (FLT3LG), transcript variant 3, mRNA [NM_001459]
XLOC_005365	A_19_P00805367	3.19E-02	-2.18		BROAD Institute lincRNA (XLOC_005365), lincRNA [TCONS_00011871]
PLB1	A_23_P56356	3.20E-02	2.66	phospholipase B1	Homo sapiens phospholipase B1 (PLB1), transcript variant 1, mRNA [NM_153021]
LOC100130348	A_33_P3211327	3.20E-02	-2.24	uncharacterized LOC100130348	Uncharacterized protein [Source:UniProtKB/TrEMBL;Acc:E9PME8] [ENST00000334821]
LOC100128402	A_33_P3290409	3.20E-02	2.30	uncharacterized LOC100128402	Homo sapiens cDNA FLJ42583 fis, clone BRACE3009090. [AK124574]
FAM78B	A_33_P3244104	3.20E-02	-2.66	family with sequence similarity	family with sequence similarity 78,

				78, member B	member B [Source:HGNC Symbol;Acc:13495] [ENST00000435676]
HAMP	A_33_P3336720	3.21E-02	-7.02	hepcidin antimicrobial peptide	Homo sapiens hepcidin antimicrobial peptide (HAMP), mRNA [NM_021175]
TEX21P	A_33_P3220822	3.21E-02	2.32	testis expressed 21, pseudogene	Homo sapiens testis expressed 21, pseudogene (TEX21P), non-coding RNA [NR_033777]
C9orf174	A_32_P180315	3.22E-02	2.05	chromosome 9 open reading frame 174	Homo sapiens chromosome 9 open reading frame 174 (C9orf174), mRNA [NM_020893]
NT5DC2	A_23_P44836	3.22E-02	-2.80	5'-nucleotidase domain containing 2	Homo sapiens 5'-nucleotidase domain containing 2 (NT5DC2), transcript variant 2, mRNA [NM_022908]
BDNF-AS1	A_33_P3323842	3.22E-02	2.39	BDNF antisense RNA 1 (non-protein coding)	Homo sapiens BDNF antisense RNA 1 (non-protein coding) (BDNF-AS1), transcript variant BT2B, non-coding RNA [NR_002832]
FLJ44054	A_33_P3265314	3.26E-02	-2.45	uncharacterized LOC643365	Homo sapiens uncharacterized LOC643365 (FLJ44054), non-coding RNA [NR_024609]
CORO7	A_33_P3343295	3.26E-02	2.04	coronin 7	Homo sapiens coronin 7 (CORO7), transcript variant 1, mRNA [NM_024535]
TFCP2L1	A_23_P5301	3.27E-02	2.99	transcription factor CP2-like 1	Homo sapiens transcription factor CP2-like 1 (TFCP2L1), mRNA [NM_014553]
HENMT1	A_23_P309361	3.27E-02	2.78	HEN1 methyltransferase homolog 1 (Arabidopsis)	Homo sapiens HEN1 methyltransferase homolog 1 (Arabidopsis) (HENMT1), transcript variant 1, mRNA [NM_144584]
DGKD	A_23_P210253	3.27E-02	-2.25	diacylglycerol kinase, delta 130kDa	Homo sapiens diacylglycerol kinase, delta 130kDa (DGKD), transcript variant 2, mRNA [NM_152879]
COX5A	A_32_P98502	3.27E-02	-2.10	cytochrome c oxidase subunit Va	Homo sapiens cytochrome c oxidase subunit Va (COX5A), nuclear gene encoding mitochondrial protein, mRNA

					[NM_004255]
CHST1	A_23_P98205	3.27E-02	-2.22	carbohydrate (keratan sulfate Gal-6) sulfotransferase 1	Homo sapiens carbohydrate (keratan sulfate Gal-6) sulfotransferase 1 (CHST1), mRNA [NM_003654]
MTMR11	A_33_P3393927	3.27E-02	2.08	myotubularin related protein 11	Homo sapiens myotubularin related protein 11 (MTMR11), transcript variant 2, mRNA [NM_181873]
FGR	A_23_P103932	3.28E-02	2.85	Gardner-Rasheed feline sarcoma viral (v-fgr) oncogene homolog	Homo sapiens Gardner-Rasheed feline sarcoma viral (v-fgr) oncogene homolog (FGR), transcript variant 2, mRNA [NM_001042747]
WDR62	A_33_P3254606	3.29E-02	-2.96	WD repeat domain 62	Homo sapiens WD repeat domain 62 (WDR62), transcript variant 1, mRNA [NM_001083961]
BCL2L11	A_24_P122921	3.29E-02	2.36	BCL2-like 11 (apoptosis facilitator)	Homo sapiens BCL2-like 11 (apoptosis facilitator) (BCL2L11), transcript variant 1, mRNA [NM_138621]
PDCD1LG2	A_33_P3213707	3.30E-02	2.71	programmed cell death 1 ligand 2	Homo sapiens programmed cell death 1 ligand 2 (PDCD1LG2), mRNA [NM_025239]
BFSP2	A_23_P159316	3.32E-02	2.83	beaded filament structural protein 2, phakinin	Homo sapiens beaded filament structural protein 2, phakinin (BFSP2), mRNA [NM_003571]
SPOCK2	A_33_P3214948	3.34E-02	2.47	sparc/osteonectin, cwcv and kazal-like domains proteoglycan (testican) 2	Homo sapiens sparc/osteonectin, cwcv and kazal-like domains proteoglycan (testican) 2 (SPOCK2), transcript variant 2, mRNA [NM_014767]
TUBB2A	A_33_P3365002	3.34E-02	-2.06	tubulin, beta 2A class IIa	Homo sapiens tubulin, beta 2A class IIa (TUBB2A), mRNA [NM_001069]
XLOC_002275	A_19_P00802420	3.34E-02	2.47		BROAD Institute lincRNA (XLOC_002275), lincRNA [TCONS_00004391]
EREG	A_23_P41344	3.36E-02	-5.66	epiregulin	Homo sapiens epiregulin (EREG), mRNA

					[NM_001432]
P2RY13	A_24_P63136	3.36E-02	3.60	purinergic receptor P2Y, G-protein coupled, 13	Homo sapiens purinergic receptor P2Y, G-protein coupled, 13 (P2RY13), mRNA [NM_176894]
TMEM119	A_33_P3395605	3.38E-02	3.89	transmembrane protein 119	Homo sapiens transmembrane protein 119 (TMEM119), mRNA [NM_181724]
UTY	A_23_P329835	3.38E-02	-4.64	ubiquitously transcribed tetratricopeptide repeat gene, Y-linked	Homo sapiens ubiquitously transcribed tetratricopeptide repeat gene, Y-linked (UTY), transcript variant 3, mRNA [NM_007125]
TRANK1	A_24_P68079	3.39E-02	3.12	tetratricopeptide repeat and ankyrin repeat containing 1	Homo sapiens tetratricopeptide repeat and ankyrin repeat containing 1 (TRANK1), mRNA [NM_014831]
PPP6R2	A_33_P3397768	3.40E-02	2.41	protein phosphatase 6, regulatory subunit 2	Homo sapiens cDNA FLJ14086 fis, clone MAMMA1000143. [AK024148]
APOBR	A_23_P54770	3.40E-02	4.23	apolipoprotein B receptor	Homo sapiens apolipoprotein B receptor (APOBR), mRNA [NM_018690]
KCNN4	A_23_P67529	3.41E-02	3.79	potassium intermediate/small conductance calcium-activated channel, subfamily N, member 4	Homo sapiens potassium intermediate/small conductance calcium-activated channel, subfamily N, member 4 (KCNN4), mRNA [NM_002250]
ANKAR	A_23_P370544	3.42E-02	2.40	ankyrin and armadillo repeat containing	Homo sapiens ankyrin and armadillo repeat containing (ANKAR), mRNA [NM_144708]
MERTK	A_33_P3402086	3.42E-02	3.59	c-mer proto-oncogene tyrosine kinase	c-mer proto-oncogene tyrosine kinase [Source:HGNC Symbol;Acc:7027] [ENST00000393237]
PRSS57	A_33_P3298775	3.42E-02	2.76	protease, serine, 57	Homo sapiens protease, serine, 57 (PRSS57), mRNA [NM_214710]
PYDC1	A_23_P407614	3.42E-02	2.38	PYD (pyrin domain) containing 1	Homo sapiens PYD (pyrin domain) containing 1 (PYDC1), mRNA [NM_152901]

DBNDD2	A_23_P28772	3.43E-02	-2.30	dysbindin (dystrobrevin binding protein 1) domain containing 2	Homo sapiens dysbindin (dystrobrevin binding protein 1) domain containing 2 (DBNDD2), transcript variant 6, mRNA [NM_001048226]
GPD1	A_23_P204736	3.45E-02	-3.03	glycerol-3-phosphate dehydrogenase 1 (soluble)	Homo sapiens glycerol-3-phosphate dehydrogenase 1 (soluble) (GPD1), mRNA [NM_005276]
CYP4F30P	A_24_P28811	3.45E-02	-2.63	cytochrome P450, family 4, subfamily F, polypeptide 30, pseudogene	Homo sapiens cytochrome P450, family 4, subfamily F, polypeptide 30, pseudogene (CYP4F30P), non-coding RNA [NR_023391]
SYT17	A_23_P163697	3.45E-02	2.42	synaptotagmin XVII	Homo sapiens synaptotagmin XVII (SYT17), mRNA [NM_016524]
ZNF287	A_23_P4416	3.46E-02	3.32	zinc finger protein 287	Homo sapiens zinc finger protein 287 (ZNF287), mRNA [NM_020653]
TRIM66	A_33_P3390773	3.48E-02	3.11	tripartite motif containing 66	Homo sapiens tripartite motif containing 66 (TRIM66), mRNA [NM_014818]
C7orf54	A_24_P389285	3.49E-02	-2.37	chromosome 7 open reading frame 54	Homo sapiens chromosome 7 open reading frame 54 (C7orf54), non-coding RNA [NR_027330]
SGK110	A_33_P3237744	3.49E-02	-3.04	putative uncharacterized serine/threonine-protein kinase SgK110-like	Homo sapiens putative uncharacterized serine/threonine-protein kinase SgK110-like (SGK110), mRNA [NM_001199824]
C8G	A_33_P3319006	3.52E-02	-2.81	complement component 8, gamma polypeptide	Homo sapiens complement component 8, gamma polypeptide (C8G), mRNA [NM_000606]
NR4A3	A_23_P398566	3.52E-02	-8.61	nuclear receptor subfamily 4, group A, member 3	Homo sapiens nuclear receptor subfamily 4, group A, member 3 (NR4A3), transcript variant 3, mRNA [NM_173200]
XLOC_005342	A_19_P00317974	3.52E-02	-3.27		BROAD Institute lincRNA (XLOC_005342), lincRNA [TCONS_00011323]

C21orf7	A_33_P3238305	3.53E-02	4.82	chromosome 21 open reading frame 7	chromosome 21 open reading frame 7 [Source:HGNC Symbol;Acc:16457] [ENST00000399926]
APOBEC2	A_23_P70387	3.53E-02	-2.01	apolipoprotein B mRNA editing enzyme, catalytic polypeptide-like 2	Homo sapiens apolipoprotein B mRNA editing enzyme, catalytic polypeptide-like 2 (APOBEC2), mRNA [NM_006789]
Q6IHG2	A_19_P00317023	3.55E-02	4.63		Q6IHG2_DROME (Q6IHG2) HDC02633, partial (8%) [THC2534303]
ABRA	A_33_P3368208	3.55E-02	-4.34	actin-binding Rho activating protein	Homo sapiens actin-binding Rho activating protein (ABRA), mRNA [NM_139166]
SCG5	A_23_P62081	3.55E-02	2.85	secretogranin V (7B2 protein)	Homo sapiens secretogranin V (7B2 protein) (SCG5), transcript variant 2, mRNA [NM_003020]
MFSD4	A_23_P365412	3.55E-02	2.39	major facilitator superfamily domain containing 4	Homo sapiens major facilitator superfamily domain containing 4 (MFSD4), mRNA [NM_181644]
XLOC_005730	A_19_P00321670	3.56E-02	-2.65		zt85h04.r1 Soares_testis_NHT Homo sapiens cDNA clone IMAGE:729175 5', mRNA sequence [AA397666]
FAM176A	A_23_P108673	3.56E-02	-3.49	family with sequence similarity 176, member A	Homo sapiens family with sequence similarity 176, member A (FAM176A), transcript variant 2, mRNA [NM_032181]
EDA2R	A_33_P3413468	3.57E-02	3.42	ectodysplasin A2 receptor	Homo sapiens ectodysplasin A2 receptor (EDA2R), transcript variant 3, mRNA [NM_001242310]
ERI2	A_23_P129717	3.57E-02	2.07	ERI1 exoribonuclease family member 2	Homo sapiens ERI1 exoribonuclease family member 2 (ERI2), transcript variant 2, mRNA [NM_080663]
VNN1	A_33_P3399571	3.60E-02	4.33	vanin 1	Homo sapiens vanin 1 (VNN1), mRNA [NM_004666]
TFF1	A_24_P322771	3.60E-02	-2.13	trefoil factor 1	Homo sapiens trefoil factor 1 (TFF1), mRNA [NM_003225]

CXCL16	A_33_P3351249	3.62E-02	3.05	chemokine (C-X-C motif) ligand 16	Homo sapiens chemokine (C-X-C motif) ligand 16 (CXCL16), transcript variant 2, mRNA [NM_001100812]
DNALI1	A_33_P3289227	3.62E-02	2.12	dynein, axonemal, light intermediate chain 1	Homo sapiens dynein, axonemal, light intermediate chain 1 (DNALI1), mRNA [NM_003462]
RPGRIP1	A_23_P88278	3.62E-02	2.98	retinitis pigmentosa GTPase regulator interacting protein 1	Homo sapiens retinitis pigmentosa GTPase regulator interacting protein 1 (RPGRIP1), mRNA [NM_020366]
MMP9	A_23_P40174	3.63E-02	7.26	matrix metallopeptidase 9 (gelatinase B, 92kDa gelatinase, 92kDa type IV collagenase)	Homo sapiens matrix metallopeptidase 9 (gelatinase B, 92kDa gelatinase, 92kDa type IV collagenase) (MMP9), mRNA [NM_004994]
MIR155HG	A_32_P108156	3.63E-02	3.40	MIR155 host gene (non-protein coding)	Homo sapiens MIR155 host gene (non-protein coding) (MIR155HG), non-coding RNA [NR_001458]
Q3T5P7	A_19_P00318803	3.64E-02	2.16		Q3T5P7_HORVD (Q3T5P7) HvCBF5, partial (10%) [THC2521292]
VSIG4	A_23_P217269	3.64E-02	2.67	V-set and immunoglobulin domain containing 4	Homo sapiens V-set and immunoglobulin domain containing 4 (VSIG4), transcript variant 1, mRNA [NM_007268]
OLFML2B	A_33_P3401243	3.64E-02	2.09	olfactomedin-like 2B	Homo sapiens olfactomedin-like 2B (OLFML2B), mRNA [NM_015441]
SIRPD	A_33_P3244610	3.64E-02	2.48	signal-regulatory protein delta	Homo sapiens signal-regulatory protein delta (SIRPD), mRNA [NM_178460]
STIM2	A_24_P180242	3.64E-02	2.03	stromal interaction molecule 2	Homo sapiens stromal interaction molecule 2 (STIM2), transcript variant 2, mRNA [NM_020860]
MB21D1	A_32_P1173	3.65E-02	2.18	Mab-21 domain containing 1	Homo sapiens Mab-21 domain containing 1 (MB21D1), mRNA [NM_138441]
NANP	A_33_P3406458	3.66E-02	-2.41	N-acetylneuraminic acid	Homo sapiens N-acetylneuraminic acid

				phosphatase	phosphatase (NANP), mRNA [NM_152667]
C1QTNF1	A_23_P3956	3.66E-02	-2.90	C1q and tumor necrosis factor related protein 1	Homo sapiens C1q and tumor necrosis factor related protein 1 (C1QTNF1), mRNA [NM_198594]
HLA-DMB	A_32_P351968	3.67E-02	3.38	major histocompatibility complex, class II, DM beta	Homo sapiens major histocompatibility complex, class II, DM beta (HLA-DMB), mRNA [NM_002118]
GGTLC1	A_23_P57199	3.68E-02	2.32	gamma-glutamyltransferase light chain 1	Homo sapiens gamma-glutamyltransferase light chain 1 (GGTLC1), transcript variant A, mRNA [NM_178311]
LAPTM5	A_23_P86283	3.69E-02	4.08	lysosomal protein transmembrane 5	Homo sapiens lysosomal protein transmembrane 5 (LAPTM5), mRNA [NM_006762]
MT1F	A_23_P15174	3.70E-02	2.84	metallothionein 1F	Homo sapiens metallothionein 1F (MT1F), mRNA [NM_005949]
MLXIPL	A_23_P145786	3.71E-02	-5.15	MLX interacting protein-like	Homo sapiens MLX interacting protein-like (MLXIPL), transcript variant 1, mRNA [NM_032951]
SUSD4	A_23_P201066	3.72E-02	-4.58	sushi domain containing 4	Homo sapiens sushi domain containing 4 (SUSD4), transcript variant 2, mRNA [NM_001037175]
XLOC_l2_009639	A_19_P00321549	3.73E-02	2.93		BROAD Institute lincRNA (XLOC_l2_009639), lincRNA [TCONS_l2_00019763]
ZNF624	A_23_P153037	3.73E-02	2.19	zinc finger protein 624	Homo sapiens zinc finger protein 624 (ZNF624), mRNA [NM_020787]
N4BP2L1	A_33_P3245415	3.73E-02	2.06	NEDD4 binding protein 2-like 1	Homo sapiens NEDD4 binding protein 2-like 1 (N4BP2L1), transcript variant 1, mRNA [NM_052818]
BOD1P	A_33_P3503279	3.75E-02	-3.11	biorientation of chromosomes in cell division 1 pseudogene	AGENCOURT_6565391 NIH_MGC_119 Homo sapiens cDNA clone

					IMAGE:5744262 5', mRNA sequence [BM559531]
ACCS	A_23_P24586	3.75E-02	2.03	1-aminocyclopropane-1-carboxylate synthase homolog (Arabidopsis)(non-functional)	Homo sapiens 1-aminocyclopropane-1-carboxylate synthase homolog (Arabidopsis)(non-functional) (ACCS), transcript variant 1, mRNA [NM_032592]
EFHC1	A_32_P86245	3.79E-02	2.57	EF-hand domain (C-terminal) containing 1	Homo sapiens EF-hand domain (C-terminal) containing 1 (EFHC1), transcript variant A, mRNA [NM_018100]
LOC100132339	A_33_P3242075	3.79E-02	-2.10	uncharacterized LOC100132339	PREDICTED: Homo sapiens hypothetical protein LOC100132339 (LOC100132339), mRNA [XM_001716934]
CCNG2	A_24_P80532	3.80E-02	2.91	cyclin G2	Homo sapiens cyclin G2 (CCNG2), mRNA [NM_004354]
LOC100240735	A_32_P170481	3.81E-02	2.71	uncharacterized LOC100240735	Homo sapiens uncharacterized LOC100240735 (LOC100240735), non-coding RNA [NR_026658]
O3FAR1	A_24_P129588	3.81E-02	-4.02	omega-3 fatty acid receptor 1	Homo sapiens omega-3 fatty acid receptor 1 (O3FAR1), transcript variant 1, mRNA [NM_181745]
LRRC25	A_23_P165136	3.81E-02	3.33	leucine rich repeat containing 25	Homo sapiens leucine rich repeat containing 25 (LRRC25), mRNA [NM_145256]
TCEANC	A_33_P3400057	3.81E-02	3.29	transcription elongation factor A (SII) N-terminal and central domain containing	Homo sapiens transcription elongation factor A (SII) N-terminal and central domain containing (TCEANC), mRNA [NM_152634]
SH3BP5	A_24_P148750	3.81E-02	2.16	SH3-domain binding protein 5 (BTK-associated)	Homo sapiens SH3-domain binding protein 5 (BTK-associated) (SH3BP5), transcript variant 1, mRNA [NM_004844]
GSDMB	A_33_P3221999	3.82E-02	2.54	gasdermin B	Homo sapiens gasdermin B (GSDMB), transcript variant 3, mRNA

					[NM_001165958]
CNOT6L	A_33_P3277943	3.85E-02	2.13	CCR4-NOT transcription complex, subunit 6-like	Homo sapiens CCR4-NOT transcription complex, subunit 6-like (CNOT6L), mRNA [NM_144571]
MYRIP	A_23_P326760	3.85E-02	3.10	myosin VIIA and Rab interacting protein	Homo sapiens myosin VIIA and Rab interacting protein (MYRIP), mRNA [NM_015460]
GLIS2	A_33_P3333224	3.85E-02	-2.16	GLIS family zinc finger 2	Homo sapiens GLIS family zinc finger 2 (GLIS2), mRNA [NM_032575]
CHST15	A_23_P383986	3.86E-02	2.32	carbohydrate (N-acetylgalactosamine 4-sulfate 6-O) sulfotransferase 15	Homo sapiens carbohydrate (N-acetylgalactosamine 4-sulfate 6-O) sulfotransferase 15 (CHST15), transcript variant 1, mRNA [NM_015892]
LINC00204B	A_33_P3371402	3.86E-02	2.14	long intergenic non-protein coding RNA 204B	Homo sapiens long intergenic non-protein coding RNA 204B (LINC00204B), non-coding RNA [NR_027402]
HLA-DQB2	A_23_P19510	3.87E-02	3.56	major histocompatibility complex, class II, DQ beta 2	Homo sapiens major histocompatibility complex, class II, DQ beta 2 (HLA-DQB2), mRNA [NM_001198858]
TESC	A_23_P76538	3.90E-02	-2.28	tescalcin	Homo sapiens tescalcin (TESC), transcript variant 1, mRNA [NM_017899]
IDO2	A_24_P12690	3.90E-02	4.82	indoleamine 2,3-dioxygenase 2	Homo sapiens indoleamine 2,3-dioxygenase 2 (IDO2), mRNA [NM_194294]
LILRA2	A_23_P142205	3.91E-02	3.12	leukocyte immunoglobulin-like receptor, subfamily A (with TM domain), member 2	Homo sapiens leukocyte immunoglobulin-like receptor, subfamily A (with TM domain), member 2 (LILRA2), transcript variant 2, mRNA [NM_006866]
DNASE1L3	A_33_P3234202	3.91E-02	4.13	deoxyribonuclease I-like 3	Homo sapiens deoxyribonuclease I-like 3 (DNASE1L3), mRNA [NM_004944]
SYNGAP1	A_24_P9346	3.91E-02	2.56	synaptic Ras GTPase activating protein 1	Homo sapiens synaptic Ras GTPase activating protein 1 (SYNGAP1), mRNA [NM_006772]

COL4A4	A_33_P3227400	3.91E-02	2.39	collagen, type IV, alpha 4	Homo sapiens collagen, type IV, alpha 4 (COL4A4), mRNA [NM_000092]
GUSBP11	A_19_P00812151	3.91E-02	2.01		Homo sapiens glucuronidase, beta pseudogene 11 (GUSBP11), non-coding RNA [NR_024448]
STAG3	A_33_P3317058	3.91E-02	2.86	stromal antigen 3	Homo sapiens stromal antigen 3 (STAG3), mRNA [NM_012447]
EMX1	A_33_P3343715	3.92E-02	-2.33	empty spiracles homeobox 1	Homo sapiens empty spiracles homeobox 1, mRNA (cDNA clone IMAGE:5198260), complete cds. [BC037242]
SLC10A2	A_23_P140009	3.92E-02	2.08	solute carrier family 10 (sodium/bile acid cotransporter family), member 2	Homo sapiens solute carrier family 10 (sodium/bile acid cotransporter family), member 2 (SLC10A2), mRNA [NM_000452]
PIM1	A_23_P345118	3.92E-02	-2.72	pim-1 oncogene	Homo sapiens pim-1 oncogene (PIM1), transcript variant 1, mRNA [NM_002648]
FKBP4	A_23_P128372	3.93E-02	-2.13	FK506 binding protein 4, 59kDa	Homo sapiens FK506 binding protein 4, 59kDa (FKBP4), mRNA [NM_002014]
LILRB2	A_23_P208493	3.93E-02	3.70	leukocyte immunoglobulin-like receptor, subfamily B (with TM and ITIM domains), member 2	Homo sapiens leukocyte immunoglobulin-like receptor, subfamily B (with TM and ITIM domains), member 2 (LILRB2), transcript variant 1, mRNA [NM_005874]
H1FNT	A_32_P202859	3.94E-02	-2.56	H1 histone family, member N, testis-specific	Homo sapiens H1 histone family, member N, testis-specific (H1FNT), mRNA [NM_181788]
C10orf108	A_33_P3322949	3.94E-02	-4.45	chromosome 10 open reading frame 108	Homo sapiens chromosome 10 open reading frame 108 (C10orf108), transcript variant 2, non-coding RNA [NR_027151]
KCNG1	A_23_P210581	3.94E-02	-4.03	potassium voltage-gated channel, subfamily G, member 1	Homo sapiens potassium voltage-gated channel, subfamily G, member 1

					(KCNG1), mRNA [NM_002237]
KIAA1598	A_33_P3419139	3.98E-02	2.31	KIAA1598	Homo sapiens KIAA1598 (KIAA1598), transcript variant 1, mRNA [NM_001127211]
PYY2	A_33_P3257866	3.98E-02	-2.27	peptide YY, 2 (seminalplasmin)	Homo sapiens peptide YY, 2 (seminalplasmin) (PYY2), non-coding RNA [NR_003064]
DPRXP4	A_33_P3306048	3.99E-02	2.05	divergent-paired related homeobox pseudogene 4	Homo sapiens divergent-paired related homeobox pseudogene 4 (DPRXP4), non-coding RNA [NR_002221]
CATSPER2	A_24_P181422	4.00E-02	2.07	cation channel, sperm associated 2	Homo sapiens cation channel, sperm associated 2 (CATSPER2), transcript variant 4, mRNA [NM_172097]
LOC100129461	A_33_P3349591	4.00E-02	-2.47	uncharacterized LOC100129461	Homo sapiens cDNA clone IMAGE:40108878. [BC133670]
PTK6	A_23_P56978	4.00E-02	2.18	PTK6 protein tyrosine kinase 6	Homo sapiens PTK6 protein tyrosine kinase 6 (PTK6), mRNA [NM_005975]
NFE2L3	A_23_P42718	4.00E-02	2.44	nuclear factor (erythroid-derived 2)-like 3	Homo sapiens nuclear factor (erythroid-derived 2)-like 3 (NFE2L3), mRNA [NM_004289]
PLVAP	A_23_P56328	4.00E-02	3.59	plasmalemma vesicle associated protein	Homo sapiens plasmalemma vesicle associated protein (PLVAP), mRNA [NM_031310]
ZNF596	A_23_P82762	4.01E-02	2.33	zinc finger protein 596	Homo sapiens zinc finger protein 596 (ZNF596), transcript variant 1, mRNA [NM_001042416]
XLOC_006829	A_19_P00809915	4.01E-02	-6.30		BROAD Institute lincRNA (XLOC_006829), lincRNA [TCONS_00014745]
RNF6	A_24_P144881	4.01E-02	-2.33	ring finger protein (C3H2C3 type) 6	Homo sapiens ring finger protein (C3H2C3 type) 6 (RNF6), transcript variant 1, mRNA [NM_005977]
MUC12	A_24_P658413	4.02E-02	2.17	mucin 12, cell surface associated	Homo sapiens mucin 12, cell surface

					associated (MUC12), mRNA [NM_001164462]
MGC4294	A_23_P102681	4.02E-02	3.15	uncharacterized MGC4294	PREDICTED: Homo sapiens hypothetical MGC4294 (MGC4294), miscRNA [XR_109628]
EPB41	A_33_P3227842	4.02E-02	2.39	erythrocyte membrane protein band 4.1 (elliptocytosis 1, RH-linked)	Homo sapiens erythrocyte membrane protein band 4.1 (elliptocytosis 1, RH-linked) (EPB41), transcript variant 3, mRNA [NM_001166006]
CDYL2	A_23_P371865	4.02E-02	2.56	chromodomain protein, Y-like 2	Homo sapiens chromodomain protein, Y-like 2 (CDYL2), mRNA [NM_152342]
CFHR3	A_23_P103256	4.03E-02	2.98	complement factor H-related 3	Homo sapiens complement factor H-related 3 (CFHR3), transcript variant 1, mRNA [NM_021023]
PPP1R32	A_23_P98571	4.03E-02	2.29	protein phosphatase 1, regulatory subunit 32	Homo sapiens protein phosphatase 1, regulatory subunit 32 (PPP1R32), nuclear gene encoding mitochondrial protein, transcript variant 1, mRNA [NM_145017]
C2orf43	A_33_P3210516	4.05E-02	2.42	chromosome 2 open reading frame 43	chromosome 2 open reading frame 43 [Source:HGNC Symbol;Acc:26145] [ENST00000419825]
SNORD17	A_33_P3263379	4.07E-02	2.15	small nucleolar RNA, C/D box 17	Homo sapiens small nucleolar RNA, C/D box 17 (SNORD17), small nucleolar RNA [NR_003045]
PIK3R1	A_24_P29401	4.08E-02	2.67	phosphoinositide-3-kinase, regulatory subunit 1 (alpha)	Homo sapiens phosphoinositide-3-kinase, regulatory subunit 1 (alpha) (PIK3R1), transcript variant 1, mRNA [NM_181523]
C9orf68	A_23_P157766	4.09E-02	2.56	chromosome 9 open reading frame 68	Homo sapiens chromosome 9 open reading frame 68 (C9orf68), mRNA [NM_001039395]
CRY1	A_24_P407235	4.10E-02	-2.09	cryptochrome 1 (photolyase-like)	Homo sapiens cryptochrome 1 (photolyase-like) (CRY1), mRNA

					[NM_004075]
ECM2	A_23_P303671	4.10E-02	3.00	extracellular matrix protein 2, female organ and adipocyte specific	Homo sapiens extracellular matrix protein 2, female organ and adipocyte specific (ECM2), transcript variant 1, mRNA [NM_001393]
MDK	A_23_P116235	4.11E-02	2.35	midkine (neurite growth-promoting factor 2)	Homo sapiens midkine (neurite growth-promoting factor 2) (MDK), transcript variant 1, mRNA [NM_001012334]
LOC729175	A_33_P3214072	4.11E-02	-3.35	uncharacterized LOC729175	PREDICTED: Homo sapiens hypothetical protein LOC729175 (LOC729175), mRNA [XM_001129558]
C1QTNF6	A_33_P3269428	4.12E-02	-2.65	C1q and tumor necrosis factor related protein 6	C1q and tumor necrosis factor related protein 6 [Source:HGNC Symbol;Acc:14343] [ENST00000255836]
CD300C	A_23_P26771	4.12E-02	2.62	CD300c molecule	Homo sapiens CD300c molecule (CD300C), mRNA [NM_006678]
HMX1	A_33_P3328450	4.12E-02	-2.75	H6 family homeobox 1	Homo sapiens H6 family homeobox 1 (HMX1), mRNA [NM_018942]
GDF10	A_23_P52227	4.13E-02	5.26	growth differentiation factor 10	Homo sapiens growth differentiation factor 10 (GDF10), mRNA [NM_004962]
TFEC	A_32_P184394	4.13E-02	4.40	transcription factor EC	Homo sapiens transcription factor EC (TFEC), transcript variant 1, mRNA [NM_012252]
JAK3	A_24_P59667	4.13E-02	4.05	Janus kinase 3	Homo sapiens Janus kinase 3 (JAK3), mRNA [NM_000215]
CEACAM1	A_33_P3268507	4.13E-02	-2.43	carcinoembryonic antigen-related cell adhesion molecule 1 (biliary glycoprotein)	Homo sapiens carcinoembryonic antigen-related cell adhesion molecule 1 (biliary glycoprotein) (CEACAM1), transcript variant 5, mRNA [NM_001184816]
FLJ45513	A_33_P3237580	4.13E-02	2.28	uncharacterized LOC729220	Homo sapiens uncharacterized LOC729220 (FLJ45513), mRNA [NM_001242791]

CLDN3	A_33_P3285565	4.16E-02	2.14	claudin 3	Homo sapiens claudin 3 (CLDN3), mRNA [NM_001306]
MRGPRF	A_23_P432573	4.20E-02	-2.67	MAS-related GPR, member F	Homo sapiens MAS-related GPR, member F (MRGPRF), transcript variant 2, mRNA [NM_145015]
ZFHX4	A_23_P43095	4.22E-02	3.00	zinc finger homeobox 4	Homo sapiens zinc finger homeobox 4 (ZFHX4), mRNA [NM_024721]
ZC3H12B	A_33_P3224735	4.22E-02	2.90	zinc finger CCCH-type containing 12B	Homo sapiens zinc finger CCCH-type containing 12B (ZC3H12B), mRNA [NM_001010888]
GDPD3	A_23_P26511	4.23E-02	3.11	glycerophosphodiester phosphodiesterase domain containing 3	Homo sapiens glycerophosphodiester phosphodiesterase domain containing 3 (GDPD3), mRNA [NM_024307]
ATP8A2	A_23_P258612	4.23E-02	-5.39	ATPase, aminophospholipid transporter, class I, type 8A, member 2	Homo sapiens ATPase, aminophospholipid transporter, class I, type 8A, member 2 (ATP8A2), mRNA [NM_016529]
IFIH1	A_23_P68155	4.24E-02	2.10	interferon induced with helicase C domain 1	Homo sapiens interferon induced with helicase C domain 1 (IFIH1), mRNA [NM_022168]
ABCG1	A_23_P166297	4.25E-02	-2.04	ATP-binding cassette, sub-family G (WHITE), member 1	Homo sapiens ATP-binding cassette, sub-family G (WHITE), member 1 (ABCG1), transcript variant 5, mRNA [NM_207627]
APCDD1	A_23_P337262	4.26E-02	2.03	adenomatosis polyposis coli down-regulated 1	Homo sapiens adenomatosis polyposis coli down-regulated 1 (APCDD1), mRNA [NM_153000]
NUDT4	A_24_P67946	4.27E-02	-2.53	nudix (nucleoside diphosphate linked moiety X)-type motif 4	Homo sapiens nudix (nucleoside diphosphate linked moiety X)-type motif 4 (NUDT4), transcript variant 2, mRNA [NM_199040]
LOC100131176	A_32_P138396	4.30E-02	3.06	uncharacterized LOC100131176	Homo sapiens cDNA FLJ38667 fis, clone HLUNG2006843. [AK095986]
CD300A	A_23_P207037	4.30E-02	2.62	CD300a molecule	Homo sapiens CD300a molecule

					(CD300A), mRNA [NM_007261]
GPSM2	A_23_P63402	4.30E-02	-3.11	G-protein signaling modulator 2	Homo sapiens G-protein signaling modulator 2 (GPSM2), mRNA [NM_013296]
PVT1	A_33_P3357748	4.32E-02	2.49	Pvt1 oncogene (non-protein coding)	Homo sapiens Pvt1 oncogene (non-protein coding) (PVT1), non-coding RNA [NR_003367]
SRRM3	A_23_P331700	4.34E-02	-3.17	serine/arginine repetitive matrix 3	Homo sapiens serine/arginine repetitive matrix 3 (SRRM3), mRNA [NM_001110199]
LOC100129463	A_33_P3373437	4.34E-02	-2.14	uncharacterized LOC100129463	PREDICTED: Homo sapiens hypothetical LOC100129463 (LOC100129463), miscRNA [XR_108760]
GEMIN8P4	A_32_P197621	4.34E-02	-3.25	gem (nuclear organelle) associated protein 8 pseudogene 4	Homo sapiens gem (nuclear organelle) associated protein 8 pseudogene 4 (GEMIN8P4), non-coding RNA [NR_002830]
LOC100129363	A_33_P3307049	4.36E-02	2.14	uncharacterized LOC100129363	Homo sapiens cDNA FLJ46623 fis, clone TLUNG2001810. [AK128478]
TUBA3C	A_23_P128598	4.37E-02	-2.00	tubulin, alpha 3c	Homo sapiens tubulin, alpha 3c (TUBA3C), mRNA [NM_006001]
TSPAN32	A_23_P501722	4.37E-02	2.63	tetraspanin 32	Homo sapiens tetraspanin 32 (TSPAN32), mRNA [NM_139022]
DDX31	A_24_P112377	4.38E-02	2.22	DEAD (Asp-Glu-Ala-Asp) box polypeptide 31	Homo sapiens DEAD (Asp-Glu-Ala-Asp) box polypeptide 31 (DDX31), transcript variant 2, mRNA [NM_138620]
LGI4	A_24_P310256	4.39E-02	2.18	leucine-rich repeat LGI family, member 4	Homo sapiens leucine-rich repeat LGI family, member 4 (LGI4), mRNA [NM_139284]
KCNMA1	A_24_P156490	4.39E-02	3.32	potassium large conductance calcium-activated channel, subfamily M, alpha member 1	Homo sapiens potassium large conductance calcium-activated channel, subfamily M, alpha member 1 (KCNMA1), transcript variant 2, mRNA

					[NM_002247]
FCRL2	A_23_P160751	4.39E-02	3.45	Fc receptor-like 2	Homo sapiens Fc receptor-like 2 (FCRL2), mRNA [NM_030764]
NQO2	A_23_P58953	4.40E-02	-2.37	NAD(P)H dehydrogenase, quinone 2	Homo sapiens NAD(P)H dehydrogenase, quinone 2 (NQO2), mRNA [NM_000904]
SNORD15A	A_33_P3578325	4.40E-02	2.04	small nucleolar RNA, C/D box 15A	Homo sapiens small nucleolar RNA, C/D box 15A (SNORD15A), small nucleolar RNA [NR_000005]
BEST3	A_33_P3249877	4.41E-02	-2.68	bestrophin 3	bestrophin 3 [Source:HGNC Symbol;Acc:17105] [ENST00000331471]
LOC100506650	A_33_P3339187	4.41E-02	-2.39	uncharacterized LOC100506650	Homo sapiens uncharacterized LOC100506650 (LOC100506650), mRNA [NM_001243552]
KLRG1	A_23_P64898	4.41E-02	2.47	killer cell lectin-like receptor subfamily G, member 1	Homo sapiens killer cell lectin-like receptor subfamily G, member 1 (KLRG1), mRNA [NM_005810]
TTC9B	A_33_P3293593	4.43E-02	-2.41	tetratricopeptide repeat domain 9B	Homo sapiens tetratricopeptide repeat domain 9B (TTC9B), mRNA [NM_152479]
PCP4L1	A_32_P214665	4.44E-02	-2.21	Purkinje cell protein 4 like 1	Homo sapiens Purkinje cell protein 4 like 1 (PCP4L1), mRNA [NM_001102566]
FRAT1	A_33_P3261173	4.44E-02	2.16	frequently rearranged in advanced T-cell lymphomas	Homo sapiens frequently rearranged in advanced T-cell lymphomas (FRAT1), mRNA [NM_005479]
NKAPP1	A_24_P365753	4.44E-02	2.19	NFKB activating protein pseudogene 1	Homo sapiens NFKB activating protein pseudogene 1 (NKAPP1), non-coding RNA [NR_027131]
COQ5	A_33_P3213512	4.44E-02	-6.28	coenzyme Q5 homolog, methyltransferase (S. cerevisiae)	coenzyme Q5 homolog, methyltransferase (S. cerevisiae) [Source:HGNC Symbol;Acc:28722] [ENST00000551769]
LOC150622	A_33_P3361292	4.45E-02	3.98	uncharacterized LOC150622	Homo sapiens uncharacterized LOC150622 (LOC150622), non-coding RNA [NR_026832]

APCDD1L	A_32_P300427	4.45E-02	6.19	adenomatosis polyposis coli down-regulated 1-like	Homo sapiens adenomatosis polyposis coli down-regulated 1-like (APCDD1L), mRNA [NM_153360]
MYPN	A_24_P376120	4.45E-02	-2.19	myopalladin	Homo sapiens myopalladin (MYPN), mRNA [NM_032578]
MS4A4A	A_23_P75769	4.45E-02	3.19	membrane-spanning 4-domains, subfamily A, member 4	Homo sapiens membrane-spanning 4-domains, subfamily A, member 4 (MS4A4A), transcript variant 2, mRNA [NM_024021]
VSIG7	A_19_P00801412	4.47E-02	2.75	V-set and immunoglobulin domain containing 7	PREDICTED: Homo sapiens V-set and immunoglobulin domain containing 7 (VSIG7), mRNA [XM_003403542]
NCF4	A_33_P3365432	4.49E-02	2.63	neutrophil cytosolic factor 4, 40kDa	Homo sapiens neutrophil cytosolic factor 4, 40kDa (NCF4), transcript variant 1, mRNA [NM_000631]
FOLR2	A_23_P47709	4.50E-02	3.03	folate receptor 2 (fetal)	Homo sapiens folate receptor 2 (fetal) (FOLR2), transcript variant 1, mRNA [NM_000803]
VDR	A_23_P162589	4.52E-02	3.32	vitamin D (1,25-dihydroxyvitamin D3) receptor	Homo sapiens vitamin D (1,25-dihydroxyvitamin D3) receptor (VDR), transcript variant 2, mRNA [NM_001017535]
SCRG1	A_23_P167159	4.54E-02	3.31	stimulator of chondrogenesis 1	Homo sapiens stimulator of chondrogenesis 1 (SCRG1), mRNA [NM_007281]
ZNF774	A_33_P3230414	4.54E-02	-2.17	zinc finger protein 774	Homo sapiens zinc finger protein 774 (ZNF774), mRNA [NM_001004309]
TLR6	A_23_P256561	4.55E-02	2.14	toll-like receptor 6	Homo sapiens toll-like receptor 6 (TLR6), mRNA [NM_006068]
KY	A_33_P3412493	4.55E-02	3.27	kyphoscoliosis peptidase	kyphoscoliosis peptidase [Source:HGNC Symbol;Acc:26576] [ENST00000506319]
WBSCR27	A_23_P381017	4.56E-02	5.68	Williams Beuren syndrome chromosome region 27	Homo sapiens Williams Beuren syndrome chromosome region 27

					(WBSCR27), mRNA [NM_152559]
HIST1H3H	A_33_P3404989	4.56E-02	2.02	histone cluster 1, H3h	Homo sapiens histone cluster 1, H3h (HIST1H3H), mRNA [NM_003536]
LOC100130930	A_33_P3361584	4.56E-02	2.38	uncharacterized LOC100130930	Homo sapiens cDNA FLJ44616 fis, clone BRACE2012936. [AK126579]
KISS1	A_23_P124892	4.58E-02	-3.29	KiSS-1 metastasis-suppressor	Homo sapiens KiSS-1 metastasis-suppressor (KISS1), mRNA [NM_002256]
SYPL2	A_33_P3385376	4.59E-02	-2.06	synaptophysin-like 2	Homo sapiens synaptophysin-like 2 (SYPL2), mRNA [NM_001040709]
FAAH	A_33_P3261803	4.59E-02	-2.15	fatty acid amide hydrolase	Homo sapiens fatty acid amide hydrolase (FAAH), mRNA [NM_001441]
XLOC_007052	A_19_P00320885	4.60E-02	-2.79		DKFZp779O0656_r1 779 (synonym: hncc1) Homo sapiens cDNA clone DKFZp779O0656 5', mRNA sequence [BX500531]
ZNF575	A_23_P401084	4.60E-02	2.10	zinc finger protein 575	Homo sapiens zinc finger protein 575 (ZNF575), mRNA [NM_174945]
LOC100128843	A_32_P834166	4.61E-02	-2.52	uncharacterized LOC100128843	Homo sapiens DNA polymerase epsilon catalytic subunit isoform a (POLE1) mRNA, partial cds. [AF128541]
NPL	A_33_P3414912	4.61E-02	2.75	N-acetylneuraminate pyruvate lyase (dihydrodipicolinate synthase)	Homo sapiens N-acetylneuraminate pyruvate lyase (dihydrodipicolinate synthase) (NPL), transcript variant 3, mRNA [NM_001200056]
LOC100132111	A_33_P3369441	4.62E-02	2.46	uncharacterized LOC100132111	Homo sapiens uncharacterized LOC100132111 (LOC100132111), non-coding RNA [NR_024237]
PYGO1	A_32_P530933	4.62E-02	-2.41	pygopus homolog 1 (Drosophila)	Homo sapiens pygopus homolog 1 (Drosophila) (PYGO1), mRNA [NM_015617]
PVRL1	A_23_P76034	4.64E-02	-2.44	poliovirus receptor-related 1 (herpesvirus entry mediator C)	Homo sapiens poliovirus receptor-related 1 (herpesvirus entry mediator C) (PVRL1), transcript variant 3, mRNA

					[NM_203286]
FCGR3A	A_23_P200728	4.65E-02	4.37	Fc fragment of IgG, low affinity IIIa, receptor (CD16a)	Homo sapiens Fc fragment of IgG, low affinity IIIa, receptor (CD16a) (FCGR3A), transcript variant 1, mRNA [NM_000569]
USP31	A_23_P206612	4.65E-02	-2.20	ubiquitin specific peptidase 31	Homo sapiens ubiquitin specific peptidase 31 (USP31), mRNA [NM_020718]
AVIL	A_24_P419087	4.65E-02	2.45	advillin	Homo sapiens advillin (AVIL), mRNA [NM_006576]
PPM1J	A_23_P201939	4.67E-02	-2.03	protein phosphatase, Mg2+/Mn2+ dependent, 1J	Homo sapiens protein phosphatase, Mg2+/Mn2+ dependent, 1J (PPM1J), mRNA [NM_005167]
XLOC_005647	A_19_P00807468	4.68E-02	-5.01		BROAD Institute lincRNA (XLOC_005647), lincRNA [TCONS_00012114]
C11orf35	A_33_P3377005	4.71E-02	2.08	chromosome 11 open reading frame 35	Homo sapiens chromosome 11 open reading frame 35 (C11orf35), mRNA [NM_173573]
PITX1	A_33_P3240328	4.71E-02	3.53	paired-like homeodomain 1	Homo sapiens paired-like homeodomain 1 (PITX1), mRNA [NM_002653]
LOC100506639	A_19_P00317159	4.71E-02	2.52		PREDICTED: Homo sapiens hypothetical LOC100506639 (LOC100506639), miscRNA [XR_108576]
XLOC_013282	A_19_P00320440	4.71E-02	2.06		HUMIGTB1A integrin beta-1 subunit {Homo sapiens} (exp=-1; wgp=0; cg=0), partial (25%) [THC2549799]
MEGF6	A_24_P926960	4.71E-02	2.85	multiple EGF-like-domains 6	Homo sapiens multiple EGF-like-domains 6 (MEGF6), mRNA [NM_001409]
MS4A14	A_23_P98565	4.72E-02	2.85	membrane-spanning 4-domains, subfamily A, member 14	Homo sapiens membrane-spanning 4-domains, subfamily A, member 14 (MS4A14), transcript variant 1, mRNA [NM_032597]
ISLR2	A_32_P379467	4.72E-02	2.63	immunoglobulin superfamily	Homo sapiens immunoglobulin

				containing leucine-rich repeat 2	superfamily containing leucine-rich repeat 2 (ISLR2), transcript variant 2, mRNA [NM_020851]
PCBP3	A_23_P147255	4.72E-02	-2.56	poly(rC) binding protein 3	Homo sapiens poly(rC) binding protein 3 (PCBP3), transcript variant 1, mRNA [NM_020528]
LINC00256A	A_24_P687326	4.72E-02	2.18	long intergenic non-protein coding RNA 256A	Homo sapiens long intergenic non-protein coding RNA 256A (LINC00256A), non-coding RNA [NR_024366]
GAB3	A_33_P3251369	4.74E-02	2.39	GRB2-associated binding protein 3	Homo sapiens GRB2-associated binding protein 3 (GAB3), transcript variant 1, mRNA [NM_001081573]
XLOC_006883	A_19_P00322611	4.75E-02	2.89		BROAD Institute lincRNA (XLOC_006883), lincRNA [TCONS_00014809]
LINC00246A	A_23_P387585	4.76E-02	2.94	long intergenic non-protein coding RNA 246A	Homo sapiens long intergenic non-protein coding RNA 246A (LINC00246A), non-coding RNA [NR_026595]
THBS3	A_23_P201047	4.76E-02	2.23	thrombospondin 3	Homo sapiens thrombospondin 3 (THBS3), mRNA [NM_007112]
TGFBR2	A_33_P3313825	4.78E-02	3.28	transforming growth factor, beta receptor II (70/80kDa)	transforming growth factor, beta receptor II (70/80kDa) [Source:HGNC Symbol;Acc:11773] [ENST00000383765]
ATP1A1OS	A_33_P3229215	4.78E-02	3.21	ATP1A1 opposite strand	Homo sapiens ATP1A1 opposite strand (ATP1A1OS), transcript variant 3, non-coding RNA [NR_024126]
RYR1	A_23_P78867	4.79E-02	2.28	ryanodine receptor 1 (skeletal)	Homo sapiens ryanodine receptor 1 (skeletal) (RYR1), transcript variant 1, mRNA [NM_000540]
SLC24A1	A_23_P205913	4.80E-02	2.46	solute carrier family 24 (sodium/potassium/calcium exchanger), member 1	Homo sapiens solute carrier family 24 (sodium/potassium/calcium exchanger), member 1 (SLC24A1), mRNA [NM_004727]

TACSTD2	A_23_P149529	4.80E-02	4.50	tumor-associated calcium signal transducer 2	Homo sapiens tumor-associated calcium signal transducer 2 (TACSTD2), mRNA [NM_002353]
NUAK2	A_33_P3338166	4.80E-02	2.06	NUAK family, SNF1-like kinase, 2	Homo sapiens NUAK family, SNF1-like kinase, 2 (NUAK2), mRNA [NM_030952]
XLOC_007290	A_19_P00322813	4.81E-02	-2.13		BC064849 C9orf93 protein {Homo sapiens} (exp=0; wgp=1; cg=0), partial (11%) [THC2626540]
C10orf57	A_23_P97853	4.83E-02	2.31	chromosome 10 open reading frame 57	Homo sapiens chromosome 10 open reading frame 57 (C10orf57), mRNA [NM_025125]
RAB11FIP4	A_23_P38427	4.83E-02	3.16	RAB11 family interacting protein 4 (class II)	Homo sapiens RAB11 family interacting protein 4 (class II) (RAB11FIP4), mRNA [NM_032932]
OR6K2	A_23_P51761	4.85E-02	-2.00	olfactory receptor, family 6, subfamily K, member 2	Homo sapiens olfactory receptor, family 6, subfamily K, member 2 (OR6K2), mRNA [NM_001005279]
ADAMDEC1	A_33_P3390172	4.85E-02	2.69	ADAM-like, decysin 1	Homo sapiens ADAM-like, decysin 1 (ADAMDEC1), transcript variant 2, mRNA [NM_001145271]
AQP7	A_23_P158041	4.87E-02	-2.02	aquaporin 7	Homo sapiens aquaporin 7 (AQP7), mRNA [NM_001170]
STAT4	A_23_P305198	4.87E-02	3.35	signal transducer and activator of transcription 4	Homo sapiens signal transducer and activator of transcription 4 (STAT4), transcript variant 1, mRNA [NM_003151]
LOC729995	A_33_P3324298	4.87E-02	-2.46	hCG1817208	PREDICTED: Homo sapiens hCG1817208 (LOC729995), miscRNA [XR_110198]
ENKUR	A_33_P3323136	4.88E-02	2.58	enkurin, TRPC channel interacting protein	Homo sapiens enkurin, TRPC channel interacting protein (ENKUR), mRNA [NM_145010]
IL17RB	A_24_P157370	4.88E-02	-3.70	interleukin 17 receptor B	Homo sapiens interleukin 17 receptor B (IL17RB), mRNA [NM_018725]
SMOC2	A_23_P70307	4.89E-02	2.34	SPARC related modular calcium	Homo sapiens SPARC related modular

				binding 2	calcium binding 2 (SMOC2), transcript variant 1, mRNA [NM_022138]
FMOD	A_23_P114883	4.89E-02	3.78	fibromodulin	Homo sapiens fibromodulin (FMOD), mRNA [NM_002023]
LOC440104	A_24_P93703	4.90E-02	2.15	1110012D08Rik pseudogene	Homo sapiens 1110012D08Rik pseudogene (LOC440104), transcript variant 1, non-coding RNA [NR_036476]
SCARNA10	A_33_P3363120	4.91E-02	2.39	small Cajal body-specific RNA 10	Homo sapiens small Cajal body-specific RNA 10 (SCARNA10), guide RNA [NR_004387]
SORCS1	A_33_P3414192	4.92E-02	-2.14	sortilin-related VPS10 domain containing receptor 1	Homo sapiens sortilin-related VPS10 domain containing receptor 1 (SORCS1), transcript variant 6, mRNA [NM_001206572]
LOC100129196	A_24_P401830	4.93E-02	2.62	uncharacterized LOC100129196	Homo sapiens uncharacterized LOC100129196 (LOC100129196), non-coding RNA [NR_034182]
UBE2L6	A_23_P75741	4.94E-02	2.12	ubiquitin-conjugating enzyme E2L 6	Homo sapiens ubiquitin-conjugating enzyme E2L 6 (UBE2L6), transcript variant 2, mRNA [NM_198183]
FAM83B	A_24_P66780	4.97E-02	-3.77	family with sequence similarity 83, member B	Homo sapiens family with sequence similarity 83, member B (FAM83B), mRNA [NM_001010872]
