# Supplementary material for: miRNAs may play a major role in the control of gene expression in key pathobiological processes in Chagas disease cardiomyopathy
Source: PLoS Negl Trop Dis. 2020 Dec 22;14(12):e0008889. doi: 10.1371/journal.pntd.0008889 (PMC7787679; doi:10.1371/journal.pntd.0008889)
Supplement: S2 Table — (PDF) [file pntd.0008889.s002.pdf]

S2 table. Description of the canonical pathways containing the DEGs in CCC myocardium.

Canonical Pathways	p-value	Ratio	z-score	Molecules
Th1 and Th2 Activation Pathway	2,5E-20	2,69E-01		CCR5, CD247, CD3D, CD3E, CD3G, CD40LG, CD8A, CXCR3, CXCR4, CXCR6, GFI1, HLA-A, HLA-DMA, HLA-DMB, HLA-DOA, HLA-DOB, HLA-DPA1, HLA-DPB1, HLA-DQB1, HLA-DQB2, HLA-DRB1, HLA-DRB5, ICOS, IFNG, IKZF1, IL10RA, IL12RB1, IL12RB2, IL17RB, IL18, IL2RB, IL2RG, IL4, ITGB2, JAK3, KLRC1, KLRD1, NFATC2, NOTCH2, PIK3CG, PIK3R1, PIK3R5, PRKCQ, RUNX3, SOCS1, STAT1, STAT4, TBX21, TGFBR2, VAV1
Th1 Pathway	1,0E-19	3,09E-01	5,209	CCR5, CD247, CD3D, CD3E, CD3G, CD40LG, CD8A, CXCR3, HLA-A, HLA-DMA, HLA-DMB, HLA-DOA, HLA-DOB, HLA-DPA1, HLA-DPB1, HLA-DQB1, HLA-DQB2, HLA-DRB1, HLA-DRB5, ICOS, IFNG, IL10RA, IL12RB1, IL12RB2, IL18, IL4, ITGB2, JAK3, KLRC1, KLRD1, NFATC2, NOTCH2, PIK3CG, PIK3R1, PIK3R5, PRKCQ, RUNX3, SOCS1, STAT1, STAT4, TBX21, VAV1
Th2 Pathway	4,0E-17	2,73E-01	1,800	CCR5, CD247, CD3D, CD3E, CD3G, CXCR4, CXCR6, GFI1, HLA-A, HLA-DMA, HLA-DMB, HLA-DOA, HLA-DOB, HLA-DPA1, HLA-DPB1, HLA-DQB1, HLA-DQB2, HLA-DRB1, HLA-DRB5, ICOS, IFNG, IKZF1, IL12RB1, IL12RB2, IL17RB, IL2RB, IL2RG, IL4, ITGB2, JAK3, NFATC2, NOTCH2, PIK3CG, PIK3R1, PIK3R5, PRKCQ, RUNX3, STAT4, TBX21, TGFBR2, VAV1
iCOS-iCOSL Signaling in T Helper Cells	2,0E-13	2,6E-01	5,014	CAMK4, CD247, CD3D, CD3E, CD3G, CD40LG, GRAP2, HLA-A, HLA-DMA, HLA-DMB, HLA-DOA, HLA-DOB, HLA-DQB1, HLA-DRB1, HLA-DRB5, ICOS, IKBKE, IL2RB, IL2RG, INPP5D, ITK, LAT, LCK, LCP2, NFATC2, PIK3CG, PIK3R1, PIK3R5, PRKCQ, PTPRC, TRAT1, VAV1, ZAP70
CD28 Signaling in T Helper Cells	1,6E-12	2,43E-01	3,273	CAMK4, CARD11, CD247, CD3D, CD3E, CD3G, CDC42, CTLA4, GRAP2, HLA-A, HLA-DMA, HLA-DMB, HLA-DOA, HLA-DOB, HLA-DQB1, HLA-DRB1, HLA-DRB5, IKBKE, ITK, LAT, LCK, LCP2, NFATC2, PIK3CG, PIK3R1, PIK3R5, PRKCQ, PTPN6, PTPRC, SYK, VAV1, WAS, ZAP70
Natural Killer Cell Signaling	6,3E-12	2,44E-01		CD244, CD247, CD300A, FCGR3A/FCGR3B, HCST, INPP5D, KIR2DS2 (includes others), KLRB1, KLRC1, KLRC3, KLRC4-KLRK1/KLRK1, KLRD1, LAIR1, LAT, LCK, LCP2, LILRB1, NCR3,

				PIK3CG, PIK3R1, PIK3R5, PRKCB, PRKCQ, PTPN6, RAC2, SH2D1A, SYK, TYROBP, VAV1, VAV3, ZAP70
T Cell Receptor Signaling	6,3E-11	2,46E-01		BTK, CAMK4, CARD11, CD247, CD3D, CD3E, CD3G, CD8A, CD8B, CTLA4, GRAP2, IKBKE, ITK, LAT, LCK, LCP2, NFATC2, PIK3CG, PIK3R1, PIK3R5, PRKCQ, PTPN7, PTPRC, PTPRH, RASGRP1, VAV1, VAV3, ZAP70
Antigen Presentation Pathway	1,1E-10	4,21E-01		CD74, HLA-A, HLA-DMA, HLA-DMB, HLA-DOA, HLA-DOB, HLA-DPA1, HLA-DPB1, HLA-DQB1, HLA-DQB2, HLA-DRB1, HLA-DRB5, HLA-E, HLA-F, IFNG, MR1
T Helper Cell Differentiation	1,2E-10	2,97E-01		CD40LG, HLA-A, HLA-DMA, HLA-DMB, HLA-DOA, HLA-DOB, HLA-DQB1, HLA-DRB1, HLA-DRB5, ICOS, IFNG, IL10RA, IL12RB1, IL12RB2, IL18, IL21R, IL2RG, IL4, STAT1, STAT4, TBX21, TGFBR2
Communication between Innate and Adaptive Immune Cells	2,1E-10	2,67E-01		CCL3, CCL3L3, CCL4, CCL5, CCR7, CD40LG, CD8A, CD8B, CXCL10, HLA-A, HLA-DRB1, HLA-DRB5, HLA-E, HLA-F, IFNG, IL18, IL4, TLR3, TLR5, TLR6, TLR8, TNFRSF17, TNFSF13, TNFSF13B
Crosstalk between Dendritic Cells and Natural Killer Cells	2,1E-10	2,67E-01		CCR7, CD226, CD40LG, CD69, FASLG, HLA-A, HLA-DRB1, HLA-DRB5, HLA-E, HLA-F, IFNG, IL15RA, IL18, IL2RB, IL2RG, IL4, ITGAL, KLRC4-KLRK1/KLRK1, KLRD1, LTB, NCR3, PRF1, TLR3, TYROBP
Altered T Cell and B Cell Signaling in Rheumatoid Arthritis	2,1E-10	2,67E-01		CD40LG, CD79A, FASLG, HLA-A, HLA-DMA, HLA-DMB, HLA-DOA, HLA-DOB, HLA-DQB1, HLA-DRB1, HLA-DRB5, IFNG, IL18, IL23A, IL4, LTB, SLAMF1, TLR3, TLR5, TLR6, TLR8, TNFRSF17, TNFSF13, TNFSF13B
Pathogenesis of Multiple Sclerosis	1,1E-09	8,89E-01		CCL3, CCL4, CCL5, CCR5, CXCL10, CXCL11, CXCL9, CXCR3
Calcium-induced T Lymphocyte Apoptosis	1,8E-09	2,86E-01	4,025	ATP2A2, ATP2A3, CAMK4, CD247, CD3D, CD3E, CD3G, HLA-A, HLA-DMA, HLA-DMB, HLA-DOA, HLA-DOB, HLA-DQB1, HLA-DRB1, HLA-DRB5, LCK, NFATC2, PRKCB, PRKCQ, ZAP70
B Cell Development	3,6E-09	0,4		CD19, CD79A, HLA-A, HLA-DMA, HLA-DMB, HLA-DOA, HLA-DOB, HLA-DQB1, HLA-DRB1, HLA-DRB5, IL7, IL7R, PTPRC, SPN
Primary Immunodeficiency Signaling	6,5E-09	3,33E-01		BLNK, BTK, CD19, CD3D, CD3E, CD40LG, CD79A, CD8A, ICOS, IGLL1/IGLL5, IL2RG, IL7R, JAK3, LCK, PTPRC, ZAP70
Autoimmune Thyroid Disease Signaling	8,9E-09	3,27E-01		CD40LG, CGA, FASLG, GZMB, HLA-A, HLA-DMA, HLA-DMB, HLA-DOA, HLA-DOB, HLA-DQB1, HLA-DRB1, HLA-DRB5, HLA-E, HLA-F, IL4, PRF1
CTLA4 Signaling in Cytotoxic T Lymphocytes	9,3E-09	2,32E-01		AP1S3, CD247, CD3D, CD3E, CD3G, CD8A, CD8B, CTLA4, GRAP2, HLA-A, LAT, LCK, LCP2, PIK3CG, PIK3R1, PIK3R5, PPM1J, PPP2R2B, PTPN22, PTPN6, SYK, TRAT1, ZAP70

PKCθ Signaling in T Lymphocytes	1,9E-08	1,99E-01	4,796	CARD11, CD247, CD3D, CD3E, CD3G, GRAP2, HLA-A, HLA-DMA, HLA-DMB, HLA-DOA, HLA-DOB, HLA-DQB1, HLA-DRB1, HLA-DRB5, IKBKE, LAT, LCK, LCP2, NFATC2, PIK3CG, PIK3R1, PIK3R5, PRKCQ, RAC2, VAV1, VAV3, ZAP70
Leukocyte Extravasation Signaling	4,0E-08	1,62E-01	3,651	ARHGAP4, ARHGAP9, BCAR1, BTK, CDC42, CLDN14, CLDN3, CXCR4, CYBA, ITGA4, ITGAL, ITGB2, ITK, MMP25, MMP9, NCF1, NCF2, NCF4, PIK3CG, PIK3R1, PIK3R5, PRKCB, PRKCQ, PTK2, PTK2B, RAC2, RASGRP1, RHOH, SELPLG, SPN, THY1, VAV1, VAV3, WAS, WIPF1
Graft-versus-Host Disease Signaling	6,9E-08	3,06E-01		FASLG, GZMB, HLA-A, HLA-DMA, HLA-DMB, HLA-DOA, HLA-DOB, HLA-DQB1, HLA-DRB1, HLA-DRB5, HLA-E, HLA-F, IFNG, IL18, PRF1
Type I Diabetes Mellitus Signaling	9,1E-08	2,07E-01	0,000	CASP8, CD247, CD3D, CD3E, CD3G, CYCS, FASLG, GZMB, HLA-A, HLA-DMA, HLA-DMB, HLA-DOA, HLA-DOB, HLA-DQB1, HLA-DRB1, HLA-DRB5, HLA-E, HLA-F, IFNG, IKBKE, PRF1, SOCS1, STAT1
Granulocyte Adhesion and Diapedesis	1,7E-07	1,68E-01		C5, CCL14, CCL17, CCL19, CCL28, CCL3, CCL3L3, CCL4, CCL5, CLDN14, CLDN3, CXCL10, CXCL11, CXCL16, CXCL9, CXCR2, CXCR4, HRH2, IL18, IL18RAP, ITGA4, ITGAL, ITGB2, MMP25, MMP9, SDC1, SELL, SELPLG, THY1, XCL1
Role of NFAT in Regulation of the Immune Response	2,2E-07	1,62E-01	5,112	BLNK, BTK, CAMK4, CD247, CD3D, CD3E, CD3G, CD79A, FCGR3A/FCGR3B, GATA4, HLA-A, HLA-DMA, HLA-DMB, HLA-DOA, HLA-DOB, HLA-DQB1, HLA-DRB1, HLA-DRB5, IKBKE, ITK, LAT, LCK, LCP2, NFATC2, PIK3CG, PIK3R1, PIK3R5, PLCB2, PRKCQ, SYK, ZAP70
Allograft Rejection Signaling	3,4E-07	2,24E-01		CD40LG, FASLG, GZMB, HLA-A, HLA-DMA, HLA-DMB, HLA-DOA, HLA-DOB, HLA-DPA1, HLA-DPB1, HLA-DQB1, HLA-DQB2, HLA-DRB1, HLA-DRB5, HLA-E, HLA-F, IFNG, IL4, PRF1
Fcγ Receptor-mediated Phagocytosis in Macrophages and Monocytes	9,5E-07	2,02E-01	4,025	CDC42, FCGR3A/FCGR3B, FGR, FYB1, INPP5D, LCP2, MYO5A, NCF1, NCK2, PIK3CG, PIK3R1, PLD4, PRKCB, PRKCQ, PTK2B, RAC2, SYK, VAV1, VAV3, WAS
Complement System	9,8E-07	3,16E-01	1,414	C1QA, C1QB, C1QC, C1S, C2, C5, C8G, CD59, CFB, CFH, ITGAX, ITGB2
IL-4 Signaling	1,0E-06	2,09E-01		HLA-A, HLA-DMA, HLA-DMB, HLA-DOA, HLA-DOB, HLA-DQB1, HLA-DRB1, HLA-DRB5, IL2RG, IL4, INPP5D, IRF4, JAK3, NFATC2, PIK3CG, PIK3R1, PIK3R5, PTPN6, SOCS1
Dendritic Cell Maturation	2,2E-06	1,52E-01	5,196	CCR7, CD1B, CD1C, CD40LG, COL1A2, COL3A1, FCGR3A/FCGR3B, HLA-A, HLA-DMA, HLA-DMB, HLA-DOA, HLA-DOB, HLA-DQB1, HLA-DRB1, HLA-DRB5, IKBKE, IL18, IL23A,

				IRF8, LTB, LY75, PIK3CG, PIK3R1, PIK3R5, PLCB2, STAT1, STAT4, TLR3, TYROBP
Role of JAK1 and JAK3 in γc Cytokine Signaling	2,5E-06	2,25E-01		BLNK, IL15RA, IL21R, IL2RB, IL2RG, IL4, IL7, IL7R, JAK3, PIK3CG, PIK3R1, PIK3R5, PTK2B, SOCS1, STAT1, SYK
OX40 Signaling Pathway	3,2E-06	2,02E-01	-1,342	CD247, CD3D, CD3E, CD3G, HLA-A, HLA-DMA, HLA-DMB, HLA-DOA, HLA-DOB, HLA-DPA1, HLA-DPB1, HLA-DQB1, HLA-DQB2, HLA-DRB1, HLA-DRB5, HLA-E, HLA-F, TRAF5
Nur77 Signaling in T Lymphocytes	6,9E-06	2,33E-01		CAMK4, CD247, CD3D, CD3E, CD3G, CYCS, HLA-A, HLA-DMA, HLA-DMB, HLA-DOA, HLA-DOB, HLA-DQB1, HLA-DRB1, HLA-DRB5
Cytotoxic T Lymphocyte-mediated Apoptosis of Target Cells	8,9E-06	3,12E-01	-0,333	CASP8, CD247, CD3D, CD3E, CD3G, CYCS, FASLG, GZMB, HLA-A, PRF1
Agranulocyte Adhesion and Diapedesis	1,7E-05	1,42E-01		C5, CCL14, CCL17, CCL19, CCL28, CCL3, CCL3L3, CCL4, CCL5, CLDN14, CLDN3, CXCL10, CXCL11, CXCL16, CXCL9, CXCR2, CXCR4, IL18, ITGA4, ITGB2, ITGB7, MMP25, MMP9, MYH6, SELL, SELPLG, XCL1
Cdc42 Signaling	1,7E-05	1,48E-01	0,000	CD247, CD3D, CD3E, CD3G, CDC42, FGD3, HLA-A, HLA-DMA, HLA-DMB, HLA-DOA, HLA-DOB, HLA-DPA1, HLA-DPB1, HLA-DQB1, HLA-DQB2, HLA-DRB1, HLA-DRB5, HLA-E, HLA-F, ITGA4, ITK, MPRIP, VAV1, WAS, WIPF1
Role of Pattern Recognition Receptors in Recognition of Bacteria and Viruses	4,7E-05	1,53E-01	4,000	C1QA, C1QB, C1QC, C5, CCL5, IFIH1, IFNG, IL18, IL4, NLRC4, NLRP3, PIK3CG, PIK3R1, PIK3R5, PRKCB, PRKCQ, SYK, TLR3, TLR5, TLR6, TLR8
Atherosclerosis Signaling	5,2E-05	1,56E-01		ALOX15B, APOA1, CCR2, CD40LG, CMA1, COL1A2, COL3A1, CXCR4, IFNG, IL18, ITGA4, ITGB2, MMP9, PLA2G4F, PLA2G7, PLAAT4, PLB1, SELPLG, SERPINA1, TNFSF14
Phagosome Formation	8,5E-05	1,56E-01		FCAR, FCGR3A/FCGR3B, FNBP1, INPP5D, ITGA4, MRC1, PIK3CG, PIK3R1, PIK3R5, PLCB2, PRKCB, PRKCQ, RHOD, RHOH, SYK, TLR3, TLR5, TLR6, TLR8
Hepatic Fibrosis / Hepatic Stellate Cell Activation	9,5E-05	1,34E-01		CCL5, CCR5, CCR7, CD40LG, COL16A1, COL1A2, COL3A1, COL4A3, COL4A4, COL5A1, COL5A2, COL6A6, CXCL9, CXCR3, FASLG, IFNG, IGF1, IL10RA, IL18RAP, IL4, MET, MMP9, MYH6, STAT1, TGFBR2
Systemic Lupus Erythematosus Signaling	1,8E-04	1,22E-01		C5, C8G, CAMK4, CD247, CD3D, CD3E, CD3G, CD40LG, CD72, CD79A, FCGR3A/FCGR3B, HLA-A, HLA-E, HLA-F, IL18, INPP5D, KNG1, LAT, LCK, NFATC2, PIK3CG, PIK3R1, PIK3R5, PIM2, PTPN6, PTPRC, RNU2-2P, TNFSF13B

Tec Kinase Signaling	1,9E-04	1,33E-01	3,838	BLK, BTK, FASLG, FGR, FNBP1, ITGA4, ITK, JAK3, LCK, PIK3CG, PIK3R1, PIK3R5, PRKCB, PRKCQ, PTK2, PTK2B, RHOD, RHOH, STAT1, STAT4, VAV1, VAV3, WAS
PI3K Signaling in B Lymphocytes	2,7E-04	1,43E-01	3,441	BLK, BLNK, BTK, CAMK4, CD19, CD79A, DAPP1, IKBKE, IL4, INPP5D, NFATC2, PIK3CG, PIK3R1, PLCB2, PRKCB, PTPRC, SYK, VAV1, VAV3
Phospholipase C Signaling	3,0E-04	1,17E-01	3,128	ADCY7, ARHGEF16, BLNK, BTK, CAMK4, CD247, CD3D, CD3E, CD3G, CD79A, FNBP1, GRAP2, HDAC10, ITGA4, ITK, LAT, LCK, LCP2, MPRIP, NFATC2, PLA2G4F, PLCB2, PLD4, PRKCB, PRKCQ, RHOD, RHOH, SYK, ZAP70
B Cell Receptor Signaling	3,1E-04	1,26E-01	2,711	APBB1IP, BLNK, BTK, CAMK4, CD19, CD79A, CDC42, DAPP1, IKBKE, INPP5D, NFATC2, PIK3CG, PIK3R1, PIK3R5, PRKCB, PRKCQ, PTK2, PTK2B, PTPN6, PTPRC, RAC2, SYK, VAV1, VAV3
CCR5 Signaling in Macrophages	3,2E-04	1,76E-01		CAMK4, CCL3, CCL4, CCL5, CCR5, CD247, CD3D, CD3E, CD3G, FASLG, PRKCB, PRKCQ, PTK2B
Tumoricidal Function of Hepatic Natural Killer Cells	3,3E-04	2,92E-01	1,342	CASP8, CYCS, ENDOG, FASLG, GZMB, ITGAL, PRF1
Production of Nitric Oxide and Reactive Oxygen Species in Macrophages	6,2E-04	1,21E-01	3,838	APOA1, CYBA, FNBP1, IFNG, IKBKE, IL4, IRF8, JAK3, NCF1, NCF2, NCF4, PIK3CG, PIK3R1, PIK3R5, PPM1J, PPP1CC, PPP2R2B, PRKCB, PRKCQ, PTPN6, RHOD, RHOH, SERPINA1, STAT1
Regulation of IL-2 Expression in Activated and Anergic T Lymphocytes	7,9E-04	1,6E-01		CAMK4, CARD11, CD247, CD3D, CD3E, CD3G, IKBKE, LAT, NFATC2, TGFBR2, VAV1, VAV3, ZAP70
Role of Macrophages, Fibroblasts and Endothelial Cells in Rheumatoid Arthritis	8,7E-04	1,05E-01		C5, CAMK4, CCL5, CEBPE, FCGR3A/FCGR3B, FRZB, IKBKE, IL16, IL18, IL18RAP, IL7, IRAK3, LEF1, LTB, NFATC2, PIK3CG, PIK3R1, PIK3R5, PLCB2, PRKCB, PRKCQ, SFRP4, SOCS1, TLR3, TLR5, TLR6, TLR8, TNFSF13B, TRAF1, TRAF5, WNT10A, WNT3, WNT3A
G-Protein Coupled Receptor Signaling	1,0E-03	1,07E-01		ADCY7, ADORA3, ADRB1, CAMK4, CXCR2, DRD4, GRM1, HRH2, IKBKE, P2RY12, P2RY13, P2RY14, PDE4A, PDE4D, PDE6A, PDE9A, PIK3CG, PIK3R1, PIK3R5, PLCB2, PRKCB, PTGDR, PTGER3, PTH1R, PTK2B, RASGRP1, RGS10, RGS18, SYNGAP1, VIPR2
IL-12 Signaling and Production in Macrophages	1,2E-03	1,27E-01		APOA1, CD40LG, IFNG, IKBKE, IL12RB1, IL12RB2, IL18, IL23A, IL4, IRF8, JMJD6, PIK3CG, PIK3R1, PIK3R5, PRKCB, PRKCQ, SERPINA1, STAT1, STAT4
Role of Osteoblasts, Osteoclasts and Chondrocytes in Rheumatoid Arthritis	1,6E-03	1,09E-01		ACP5, BMP6, BMP7, CAMK4, CSF1R, CTSK, FRZB, IFNG, IGF1, IKBKE, IL18, IL18RAP, IL4, IL7, LEF1, NFATC2, PIK3CG, PIK3R1,

				PIK3R5, PTK2B, RUNX2, SFRP4, TRAF5, WNT10A, WNT3, WNT3A
IL-15 Signaling	1,6E-03	1,56E-01		FASLG, IL15RA, IL2RB, IL2RG, IL4, JAK3, LCK, PIK3CG, PIK3R1, PIK3R5, PTK2, SYK
Inflammasome pathway	1,7E-03	2,61E-01	2,449	AIM2, CASP8, IL18, NLRC4, NLRP3, PYCARD
Granzyme B Signaling	1,7E-03	3,12E-01	0,000	CASP8, CYCS, ENDOG, GZMB, PRF1
NF-κB Signaling	1,9E-03	1,17E-01	3,710	CARD11, CASP8, CD40LG, IL18, IRAK3, LCK, PIK3CG, PIK3R1, PIK3R5, PRKCB, PRKCQ, TGFBR2, TLR3, TLR5, TLR6, TLR8, TNFAIP3, TNFRSF17, TNFSF13B, TRAF5, ZAP70
Fc Epsilon RI Signaling	2,6E-03	1,28E-01	2,673	BTK, GRAP2, IL4, INPP5D, LAT, LCP2, PIK3CG, PIK3R1, PIK3R5, PLA2G4F, PRKCB, PRKCQ, RAC2, SYK, VAV1, VAV3
Sperm Motility	3,4E-03	1,21E-01	1,069	ATP1A4, CAMK4, CATSPER1, CATSPER2, NPPA, PDE4A, PDE4D, PLA2G4F, PLA2G7, PLAAT4, PLB1, PLCB2, PRKCB, PRKCQ, PTK2, PTK2B, PTK6
Axonal Guidance Signaling	4,1E-03	9,01E-02		ABLIM2, ADAM11, ADAM28, ADAM8, AOPEP, BCAR1, BMP6, BMP7, CDC42, CXCR4, GLIS1, GLIS2, IGF1, ITGA4, LRRC4C, MET, MICAL1, MMP9, NCK2, NFATC2, PIK3CG, PIK3R1, PIK3R5, PLCB2, PLXNC1, PRKCB, PRKCQ, PTK2, RAC2, RHOD, SEMA4D, SEMA6B, SLIT2, SRGAP2, TUBA3C/TUBA3D, TUBB2A, WAS, WIPF1, WNT10A, WNT3, WNT3A
Chemokine Signaling	4,1E-03	1,47E-01	2,714	CAMK4, CCL4, CCL5, CCR5, CXCR4, MPRIP, PIK3CG, PLCB2, PRKCB, PTK2, PTK2B
TREM1 Signaling	4,5E-03	1,45E-01	3,317	CCL3, IL18, ITGAX, NLRC3, NLRC4, NLRP3, TLR3, TLR5, TLR6, TLR8, TYROBP
IL-2 Signaling	4,8E-03	1,52E-01	2,530	IL2RB, IL2RG, JAK3, LCK, PIK3CG, PIK3R1, PIK3R5, PTK2B, SOCS1, SYK
Hematopoiesis from Pluripotent Stem Cells	5,5E-03	1,7E-01		CD247, CD3D, CD3E, CD3G, CD8A, CD8B, IL4, IL7
Molecular Mechanisms of Cancer	5,8E-03	9,16E-02		ADCY7, ARHGEF16, AURKA, BCL2L11, BMP6, BMP7, CASP8, CDC42, CDKN2A, CHEK2, CYCS, E2F2, FANCD2, FASLG, FNBP1, HHAT, ITGA4, JAK3, LEF1, PIK3CG, PIK3R1, PIK3R5, PLCB2, PRKCB, PRKCQ, PTK2, RAC2, RASGRP1, RHOD, RHOH, SYNGAP1, TGFBR2, WNT10A, WNT3, WNT3A
Paxillin Signaling	6,2E-03	1,24E-01	1,000	BCAR1, CDC42, ITGA4, ITGAD, ITGAL, ITGAX, ITGB2, ITGB7, NCK2, PIK3CG, PIK3R1, PIK3R5, PTK2, PTK2B
p70S6K Signaling	6,3E-03	1,17E-01	3,000	BTK, CD19, CD79A, F2R, F2RL2, IL2RG, IL4, PIK3CG, PIK3R1, PIK3R5, PLCB2, PPM1J, PPP2R2B, PRKCB, PRKCQ, SYK
3-phosphoinositide Biosynthesis	6,5E-03	1,04E-01		ACP5, CD19, CILP, DUSP13, ICOS, LCK, MET, NUDT4, PIK3CG, PIK3R1, PIK3R5, PIP4K2A, PPP1CC, PPP1R1A, PTPN22, PTPN6, PTPN7, PTPRC, PTPRH, PTPRO, TRAT1, VAV1

IL-8 Signaling	7,2E-03	1,04E-01	3,710	ANGPT1, CXCR2, FNBP1, IKBKE, IRAK3, ITGAX, ITGB2, MMP9, NCF2, PIK3CG, PIK3R1, PIK3R5, PLCB2, PLD4, PRKCB, PRKCQ, PTK2, PTK2B, RAC2, RHOD, RHOH
Sphingosine-1-phosphate Signaling	7,2E-03	1,18E-01	1,069	ADCY7, CASP8, FNBP1, NAAA, PIK3CG, PIK3R1, PIK3R5, PLCB2, PTK2, PTK2B, RHOD, RHOH, S1PR4, SMPD3, SPHK1
Macropinocytosis Signaling	7,2E-03	1,36E-01	1,414	CDC42, CSF1R, ITGB2, ITGB7, MET, MRC1, PIK3CG, PIK3R1, PIK3R5, PRKCB, PRKCQ
Role of PKR in Interferon Induction and Antiviral Response	7,8E-03	1,75E-01		CASP8, CYCS, IFNG, IKBKE, STAT1, TLR3, TRAF5
Superpathway of Inositol Phosphate Compounds	8,7E-03	9,73E-02		ACP5, CD19, CILP, DUSP13, ICOS, INPP5D, LCK, MET, NUDT4, PIK3CG, PIK3R1, PIK3R5, PIP4K2A, PLCB2, PLD4, PPP1CC, PPP1R1A, PTPN22, PTPN6, PTPN7, PTPRC, PTPRH, PTPRO, TRAT1, VAV1
Gα12/13 Signaling	9,5E-03	1,15E-01	2,840	BTK, CDC42, CDH16, CDH9, F2R, F2RL2, IKBKE, LPAR2, PIK3CG, PIK3R1, PIK3R5, PTK2, PTK2B, VAV1, VAV3
Eicosanoid Signaling	1,0E-02	1,29E-01	1,633	ALOX15B, ALOX5AP, CYSLTR1, DPEP2, LTC4S, PLA2G4F, PLA2G7, PLAAT4, PLB1, PTGDR, PTGER3
Role of JAK1, JAK2 and TYK2 in Interferon Signaling	1,1E-02	2,08E-01	0,000	CGA, IFNG, PTPN6, SOCS1, STAT1
Role of Hypercytokinemia/hyperchemokinemia in the Pathogenesis of Influenza	1,1E-02	1,63E-01		CCL3, CCL4, CCL5, CCR5, CXCL10, IFNG, IL18
NF-κB Activation by Viruses	1,2E-02	1,26E-01	3,162	CCR5, IKBKE, ITGA4, ITGAL, ITGB2, LCK, PIK3CG, PIK3R1, PIK3R5, PRKCB, PRKCQ
Actin Nucleation by ARP-WASP Complex	1,5E-02	1,43E-01	0,816	CDC42, FNBP1, ITGA4, NCK2, RHOD, RHOH, WAS, WIPF1
Neuroprotective Role of THOP1 in Alzheimer's Disease	1,7E-02	1,52E-01		HLA-A, HLA-E, HLA-F, IFNG, KNG1, MMP9, PNOC
UVA-Induced MAPK Signaling	1,7E-02	1,17E-01	2,714	CYCS, PARP10, PARP15, PARP4, PARP8, PIK3CG, PIK3R1, PIK3R5, PLCB2, SMPD3, STAT1, ZC3HAV1
FcγRIIB Signaling in B Lymphocytes	1,7E-02	1,4E-01	1,414	BLNK, BTK, CD79A, INPP5D, PIK3CG, PIK3R1, PIK3R5, SYK
Reelin Signaling in Neurons	1,8E-02	1,2E-01		ARHGEF16, BLK, FGR, ITGA4, ITGAL, ITGB2, LCK, MAP4K1, PIK3CG, PIK3R1, PIK3R5
Lymphotoxin β Receptor Signaling	1,9E-02	1,3E-01	2,333	CYCS, IKBKE, LTB, PIK3CG, PIK3R1, PIK3R5, TNFSF14, TRAF1, TRAF5
IL-15 Production	1,9E-02	1,85E-01		JAK3, PTK2, PTK2B, PTK6, STAT1
Integrin Signaling	1,9E-02	9,55E-02	2,524	BCAR1, CDC42, FNBP1, ITGA4, ITGAD, ITGAL, ITGAX, ITGB2, ITGB7, MPRIP, NCK2, PIK3CG, PIK3R1, PIK3R5, PTK2, RAC2, RHOD, RHOH, TSPAN5, WAS, WIPF1
Gαq Signaling	1,9E-02	1,01E-01	3,153	BTK, CAMK4, FNBP1, GRM1, IKBKE, NFATC2, PIK3CG, PIK3R1, PIK3R5, PLCB2, PLD4, PRKCB, PRKCQ, PTK2B, RGS18, RHOD, RHOH

Acute Myeloid Leukemia Signaling	2,1E-02	1,17E-01	1,414	CSF1R, CSF2RA, FLT3, FLT3LG, IDH2, LEF1, PIK3CG, PIK3R1, PIK3R5, PIM1, PIM2
April Mediated Signaling	2,2E-02	1,58E-01	2,449	IKBKE, NFATC2, TNFRSF17, TNFSF13, TRAF1, TRAF5
Glioma Invasiveness Signaling	2,2E-02	1,27E-01	1,667	F2R, FNBP1, MMP9, PIK3CG, PIK3R1, PIK3R5, PTK2, RHOD, RHOH
ILK Signaling	2,3E-02	9,6E-02	1,213	CDC42, FBLIM1, FERMT2, FNBP1, ITGB2, ITGB7, LEF1, MMP9, MYH6, NCK2, PIK3CG, PIK3R1, PIK3R5, PPM1J, PPP2R2B, PTK2, RHOD, RHOH, TMSB10/TMSB4X
Virus Entry via Endocytic Pathways	2,4E-02	1,11E-01		CDC42, HLA-A, ITGA4, ITGAL, ITGB2, ITGB7, PIK3CG, PIK3R1, PIK3R5, PRKCB, PRKCQ, RAC2
PTEN Signaling	2,5E-02	1,07E-01	0,000	BCAR1, BCL2L11, CDC42, FASLG, IKBKE, INPP5D, ITGA4, PIK3CG, PIK3R1, PIK3R5, PTK2, RAC2, TGFBR2
cAMP-mediated signaling	2,5E-02	9,25E-02	0,000	ADCY7, ADORA3, ADRB1, AKAP10, CAMK4, CXCR2, DRD4, HRH2, P2RY12, P2RY13, P2RY14, PDE4A, PDE4D, PDE6A, PDE9A, PTGDR, PTGER3, PTH1R, RGS10, RGS18, VIPR2
Caveolar-mediated Endocytosis Signaling	2,6E-02	1,23E-01		CD48, DYRK3, HLA-A, ITGA4, ITGAD, ITGAL, ITGAX, ITGB2, ITGB7
Ephrin A Signaling	2,8E-02	1,29E-01		BCAR1, CDC42, PIK3CG, PIK3R1, PIK3R5, PTK2, VAV1, VAV3
B Cell Activating Factor Signaling	2,8E-02	1,5E-01	2,449	IKBKE, NFATC2, TNFRSF17, TNFSF13B, TRAF1, TRAF5
Toll-like Receptor Signaling	2,8E-02	1,22E-01	1,342	IL18, IRAK3, TLR3, TLR5, TLR6, TLR8, TNFAIP3, TRAF1, UBD
Retinoic acid Mediated Apoptosis Signaling	3,2E-02	1,25E-01	2,121	CASP8, CYCS, IFNG, PARP10, PARP15, PARP4, PARP8, ZC3HAV1
CD40 Signaling	3,8E-02	1,15E-01	2,333	CD40LG, IKBKE, JAK3, PIK3CG, PIK3R1, PIK3R5, TNFAIP3, TRAF1, TRAF5
Colorectal Cancer Metastasis Signaling	4,0E-02	8,73E-02	2,558	ADCY7, FNBP1, IFNG, JAK3, LEF1, MMP25, MMP9, PIK3CG, PIK3R1, PIK3R5, PTGER3, RHOD, RHOH, STAT1, TGFBR2, TLR3, TLR5, TLR6, TLR8, WNT10A, WNT3, WNT3A
Death Receptor Signaling	4,2E-02	1,09E-01	2,530	ARHGDIB, CASP8, CYCS, FASLG, IKBKE, PARP10, PARP15, PARP4, PARP8, ZC3HAV1
Differential Regulation of Cytokine Production in Intestinal Epithelial Cells by IL-17A and IL-17F	4,3E-02	1,74E-01		CCL3, CCL4, CCL5, IFNG
Regulation of Actin-based Motility by Rho	4,5E-02	1,08E-01	1,897	CDC42, FNBP1, ITGA4, MPRIP, PIP4K2A, RAC2, RHOD, RHOH, WAS, WIPF1
IL-9 Signaling	4,6E-02	1,33E-01	2,449	IL2RG, JAK3, PIK3CG, PIK3R1, PIK3R5, STAT1
Pancreatic Adenocarcinoma Signaling	4,8E-02	0,1	3,000	BRCA2, CDC42, CDKN2A, E2F2, JAK3, MMP9, PIK3CG, PIK3R1, PIK3R5, PLD4, STAT1, TGFBR2
Myc Mediated Apoptosis Signaling	5,1E-02	1,14E-01		CASP8, CDKN2A, CYCS, FASLG, IGF1, PIK3CG, PIK3R1, PIK3R5

Actin Cytoskeleton Signaling	5,4E-02	8,62E-02	2,357	BCAR1, CDC42, F2R, FGD3, ITGA4, KNG1, MATK, MPRIP, MYH6, NCKAP1L, PIK3CG, PIK3R1, PIK3R5, PIP4K2A, PTK2, RAC2, TMSB10/TMSB4X, VAV1, VAV3, WAS
iNOS Signaling	5,5E-02	1,28E-01	2,236	CAMK4, IFNG, IKBKE, IRAK3, JAK3, STAT1
Glioblastoma Multiforme Signaling	5,8E-02	9,15E-02	1,069	CDC42, CDKN2A, E2F2, FNBP1, IGF1, LEF1, PIK3CG, PIK3R1, PIK3R5, PLCB2, RHOD, RHOH, WNT10A, WNT3, WNT3A
Growth Hormone Signaling	6,0E-02	1,06E-01	1,414	IGF1, PIK3CG, PIK3R1, PIK3R5, PRKCB, PRKCQ, PTPN6, SOCS1, STAT1
Basal Cell Carcinoma Signaling	6,2E-02	1,1E-01	0,000	BMP6, BMP7, GLIS1, GLIS2, LEF1, WNT10A, WNT3, WNT3A
D-myo-inositol-5-phosphate Metabolism	6,8E-02	8,93E-02		ACP5, CILP, DUSP13, NUDT4, PIP4K2A, PLCB2, PLD4, PPP1CC, PPP1R1A, PTPN22, PTPN6, PTPN7, PTPRC, PTPRH, PTPRO
fMLP Signaling in Neutrophils	7,1E-02	9,38E-02	2,887	CAMK4, CDC42, NCF1, NCF2, NFATC2, PIK3CG, PIK3R1, PIK3R5, PLCB2, PRKCB, PRKCQ, WAS
HER-2 Signaling in Breast Cancer	7,1E-02	1,02E-01		CDC42, ITGB2, ITGB7, PARD6B, PIK3CG, PIK3R1, PIK3R5, PRKCB, PRKCQ
Protein Kinase A Signaling	7,4E-02	7,67E-02	0,000	ADCY7, AKAP10, CAMK4, CDKN3, DUSP18, EBI3, HHAT, LEF1, NFATC2, PDE4A, PDE4D, PDE6A, PDE9A, PLCB2, PPP1CC, PRKCB, PRKCQ, PTK2, PTK2B, PTPN18, PTPN22, PTPN6, PTPN7, PTPRC, PTPRH, PTPRO, PTPRU, PYGM, RYR1, TGFBR2, TNNI2
Acute Phase Response Signaling	7,6E-02	8,77E-02	1,000	APOA1, C1S, C2, C5, CFB, CP, HAMP, HMOX2, IKBKE, IL18, PIK3CG, PIK3R1, RBP5, SERPINA1, SOCS1
Cardiac β-adrenergic Signaling	8,1E-02	8,97E-02	-0,577	ADCY7, ADRB1, AKAP10, ATP2A2, ATP2A3, PDE4A, PDE4D, PDE6A, PDE9A, PPM1J, PPP1CC, PPP1R1A, PPP2R2B
Differential Regulation of Cytokine Production in Macrophages and T Helper Cells by IL-17A and IL-17F	8,5E-02	1,67E-01		CCL3, CCL4, CCL5
Tryptophan Degradation to 2-amino-3-carboxymuconate Semialdehyde	8,5E-02	1,67E-01		HAAO, IDO2, KMO
γ-glutamyl Cycle	8,7E-02	1,38E-01		ANPEP, CHAC2, GGACT, GGTLC1
Semaphorin Signaling in Neurons	8,7E-02	1,13E-01		FNBP1, MET, PTK2, RHOD, RHOH, SEMA4D
Regulation of the Epithelial-Mesenchymal Transition Pathway	8,7E-02	8,47E-02		CLDN3, JAK3, LEF1, LOX, MET, MMP9, NOTCH2, PARD6B, PIK3CG, PIK3R1, PIK3R5, PYGO1, TGFBR2, WNT10A, WNT3, WNT3A
Factors Promoting Cardiogenesis in Vertebrates	8,9E-02	9,78E-02		BMP6, BMP7, GATA4, LEF1, NPPA, PRKCB, PRKCQ, TGFBR2, WNT3
Angiopoietin Signaling	8,9E-02	1,01E-01	-0,447	ANGPT1, ANGPTL1, DOK2, IKBKE, PIK3CG, PIK3R1, PIK3R5, PTK2

Cholecystokinin/Gastrin-mediated Signaling	9,1E-02	9,43E-02	1,265	BCAR1, FNBP1, IL18, PLCB2, PRKCB, PRKCQ, PTK2, PTK2B, RHOD, RHOH
Unfolded protein response	9,3E-02	1,11E-01		CEBPE, EDEM1, ERN1, HSPA6, PDIA2, SREBF1
Intrinsic Prothrombin Activation Pathway	9,5E-02	1,33E-01		COL1A2, COL3A1, F13A1, KNG1
Thrombin Signaling	1,1E-01	8,13E-02	1,698	ADCY7, ARHGEF16, CAMK4, F2R, F2RL2, FNBP1, GATA4, MPRIP, PIK3CG, PIK3R1, PIK3R5, PLCB2, PRKCB, PRKCQ, PTK2, RHOD, RHOH
Cardiomyocyte Differentiation via BMP Receptors	1,1E-01	1,5E-01		BMP7, GATA4, NPPA
IL-3 Signaling	1,1E-01	9,64E-02	1,414	INPP5D, PIK3CG, PIK3R1, PIK3R5, PRKCB, PRKCQ, PTPN6, STAT1
JAK/Stat Signaling	1,1E-01	9,64E-02	1,414	JAK3, PIK3CG, PIK3R1, PIK3R5, PTPN6, SOCS1, STAT1, STAT4
Netrin Signaling	1,1E-01	1,14E-01		ABLIM2, NCK2, NFATC2, RAC2, RYR1
Ceramide Signaling	1,1E-01	9,28E-02	-1,414	CYCS, PIK3CG, PIK3R1, PIK3R5, PPM1J, PPP2R2B, S1PR4, SMPD3, SPHK1
Agrin Interactions at Neuromuscular Junction	1,1E-01	0,1	0,816	CDC42, ITGA4, ITGAL, ITGB2, PKLR, PTK2, RAC2
FLT3 Signaling in Hematopoietic Progenitor Cells	1,2E-01	9,41E-02	2,121	FLT3, FLT3LG, INPP5D, PIK3CG, PIK3R1, PIK3R5, STAT1, STAT4
Wnt/β-catenin Signaling	1,2E-01	8,24E-02	-0,632	CDKN2A, FRAT1, FRZB, LEF1, MAP4K1, PPM1J, PPP2R2B, SFRP4, SOX10, TGFBR2, UBD, WNT10A, WNT3, WNT3A
Prolactin Signaling	1,3E-01	9,3E-02	2,121	PIK3CG, PIK3R1, PIK3R5, PRKCB, PRKCQ, SOCS1, STAT1, TCF7
Renin-Angiotensin Signaling	1,3E-01	8,53E-02	2,530	ADCY7, CCL5, PIK3CG, PIK3R1, PIK3R5, PRKCB, PRKCQ, PTK2, PTK2B, PTPN6, STAT1
Small Cell Lung Cancer Signaling	1,3E-01	9,2E-02		CYCS, IKBKE, PIK3CG, PIK3R1, PIK3R5, PTK2, TRAF1, TRAF5
TWEAK Signaling	1,3E-01	1,18E-01	0,000	CASP8, CYCS, IKBKE, TRAF1
TGF-β Signaling	1,3E-01	9,2E-02	1,342	BMP7, CDC42, MAP4K1, RUNX2, RUNX3, TFE3, TGFBR2, VDR
Glucocorticoid Receptor Signaling	1,4E-01	7,51E-02		BAG1, CCL3, CCL5, CD247, CD3D, CD3E, CD3G, ERCC2, FKBP4, HSPA6, IFNG, IKBKE, IL4, JAK3, NFATC2, NPPA, PIK3CG, PIK3R1, PIK3R5, STAT1, TGFBR2, TSC22D3
Induction of Apoptosis by HIV1	1,4E-01	0,1	1,633	CASP8, CXCR4, CYCS, FASLG, IKBKE, TRAF1
MSP-RON Signaling Pathway	1,4E-01	0,1		CCR2, IFNG, ITGB2, PIK3CG, PIK3R1, PIK3R5
GM-CSF Signaling	1,4E-01	9,46E-02	1,633	CSF2RA, PIK3CG, PIK3R1, PIK3R5, PIM1, PRKCB, STAT1
Coagulation System	1,4E-01	1,14E-01	1,000	F13A1, F2R, KNG1, SERPINA1
Role of MAPK Signaling in the Pathogenesis of Influenza	1,5E-01	9,33E-02		CCL5, CXCL10, IFNG, PLA2G4F, PLA2G7, PLAAT4, PLB1
Ephrin B Signaling	1,5E-01	9,33E-02	0,816	CDC42, CXCR4, NCK2, PTK2, RAC2, VAV1, VAV3
Germ Cell-Sertoli Cell Junction Signaling	1,5E-01	7,95E-02		BCAR1, CDC42, FNBP1, PIK3CG, PIK3R1, PIK3R5, PTK2, RAB8B, RAC2, RHOD, RHOH, TGFBR2, TUBA3C/TUBA3D, TUBB2A

Gustation Pathway	1,5E-01	8,33E-02		ADCY7, P2RX3, P2RY12, P2RY13, P2RY14, P2RY2, PDE4A, PDE4D, PDE6A, PDE9A, PLCB2
Neuropathic Pain Signaling In Dorsal Horn Neurons	1,5E-01	8,47E-02	2,530	CAMK4, GRM1, KCNN2, KCNN4, PIK3CG, PIK3R1, PIK3R5, PLCB2, PRKCB, PRKCQ
HGF Signaling	1,5E-01	8,47E-02	0,632	CDC42, CDKN2A, ITGA4, MET, PIK3CG, PIK3R1, PIK3R5, PRKCB, PRKCQ, PTK2
Glioma Signaling	1,5E-01	8,47E-02	2,333	CAMK4, CDKN2A, E2F2, IDH2, IGF1, PIK3CG, PIK3R1, PIK3R5, PRKCB, PRKCQ
Activation of IRF by Cytosolic Pattern Recognition Receptors	1,5E-01	9,68E-02	1,633	DHX58, IFIH1, IFIT2, IKBKE, STAT1, ZBP1
Hematopoiesis from Multipotent Stem Cells	1,5E-01	1,67E-01		IL4, IL7
TNFR1 Signaling	1,6E-01	1,02E-01	0,000	CASP8, CDC42, CYCS, IKBKE, TNFAIP3
Human Embryonic Stem Cell Pluripotency	1,6E-01	8,11E-02		BMP6, BMP7, LEF1, PIK3CG, PIK3R1, PIK3R5, S1PR4, SPHK1, TGFBR2, WNT10A, WNT3, WNT3A
D-myo-inositol (1,4,5,6)-Tetrakisphosphate Biosynthesis	1,6E-01	8,05E-02		ACP5, CILP, DUSP13, NUDT4, PPP1CC, PPP1R1A, PTPN22, PTPN6, PTPN7, PTPRC, PTPRH, PTPRO
D-myo-inositol (3,4,5,6)-tetrakisphosphate Biosynthesis	1,6E-01	8,05E-02		ACP5, CILP, DUSP13, NUDT4, PPP1CC, PPP1R1A, PTPN22, PTPN6, PTPN7, PTPRC, PTPRH, PTPRO
3-phosphoinositide Degradation	1,6E-01	7,93E-02		ACP5, CILP, DUSP13, INPP5D, NUDT4, PPP1CC, PPP1R1A, PTPN22, PTPN6, PTPN7, PTPRC, PTPRH, PTPRO
Breast Cancer Regulation by Stathmin1	1,6E-01	7,66E-02		ADCY7, ARHGEF16, CAMK4, CDC42, E2F2, PIK3CG, PIK3R1, PIK3R5, PLCB2, PPM1J, PPP1CC, PPP2R2B, PRKCB, PRKCQ, TUBA3C/TUBA3D, TUBB2A
Role of BRCA1 in DNA Damage Response	1,7E-01	8,97E-02	0,000	BRCA2, CHEK2, E2F2, FANCA, FANCD2, IFNG, STAT1
Role of PI3K/AKT Signaling in the Pathogenesis of Influenza	1,7E-01	8,97E-02	2,449	CCL5, CCR5, IFNG, PIK3CG, PIK3R1, PIK3R5, PLAC8
Role of NANOG in Mammalian Embryonic Stem Cell Pluripotency	1,8E-01	8,2E-02	2,000	BMP6, BMP7, GATA4, JAK3, PIK3CG, PIK3R1, PIK3R5, WNT10A, WNT3, WNT3A
IL-17A Signaling in Gastric Cells	1,8E-01	1,2E-01		CCL5, CXCL10, CXCL11
Leukotriene Biosynthesis	1,8E-01	1,2E-01		DPEP2, GGTLC1, LTC4S
VDR/RXR Activation	1,8E-01	8,86E-02	0,378	CCL5, CXCL10, IFNG, PRKCB, PRKCQ, RUNX2, VDR
GDNF Family Ligand-Receptor Interactions	1,8E-01	8,86E-02	1,890	CDC42, DOK2, DOK3, GFRA2, PIK3CG, PIK3R1, PIK3R5
CD27 Signaling in Lymphocytes	1,9E-01	9,62E-02	1,342	CASP8, CD27, CYCS, IKBKE, TRAF5
Lipid Antigen Presentation by CD1	1,9E-01	1,15E-01		CD1B, CD1C, CD3E
Synaptic Long Term Depression	2,0E-01	7,74E-02	2,309	GRM1, IGF1, PLA2G4F, PLA2G7, PLAAT4, PLB1, PLCB2, PPM1J, PPP2R2B, PRKCB, PRKCQ, RYR1
DNA Double-Strand Break Repair by Homologous Recombination	2,0E-01	1,43E-01		BRCA2, LIG1
Calcium Transport I	2,0E-01	1,43E-01		ATP2A2, ATP2A3

Sphingomyelin Metabolism	2,0E-01	1,43E-01		SGMS2, SMPD3
PDGF Signaling	2,0E-01	8,33E-02	2,121	INPP5D, JAK3, PIK3CG, PIK3R1, PIK3R5, PRKCB, SPHK1, STAT1
Erythropoietin Signaling	2,0E-01	8,54E-02		PIK3CG, PIK3R1, PIK3R5, PRKCB, PRKCQ, PTPN6, SOCS1
Nitric Oxide Signaling in the Cardiovascular System	2,0E-01	7,94E-02	1,265	ADRB1, ATP2A2, ATP2A3, CAMK4, KNG1, PIK3CG, PIK3R1, PIK3R5, PRKCB, PRKCQ
CXCR4 Signaling	2,1E-01	7,51E-02	1,387	ADCY7, BCAR1, CXCR4, FNBP1, PIK3CG, PIK3R1, PIK3R5, PLCB2, PRKCB, PRKCQ, PTK2, RHOD, RHOH
ErbB Signaling	2,1E-01	8,16E-02		CDC42, EREG, NCK2, PIK3CG, PIK3R1, PIK3R5, PRKCB, PRKCQ
LXR/RXR Activation	2,1E-01	7,81E-02	-0,707	ABCG1, APOA1, IL18, IL18RAP, KNG1, MLXIPL, MMP9, SERPINA1, SREBF1, TLR3
Thrombopoietin Signaling	2,1E-01	8,7E-02	2,449	PIK3CG, PIK3R1, PIK3R5, PRKCB, PRKCQ, STAT1
Role of CHK Proteins in Cell Cycle Checkpoint Control	2,2E-01	9,09E-02	-1,342	CHEK2, E2F2, PPM1J, PPP2R2B, RAD1
Hepatic Cholestasis	2,2E-01	7,47E-02		ABCC3, ADCY7, IFNG, IKBKE, IL18, IL18RAP, IL4, IRAK3, PRKCB, PRKCQ, SLC10A2, SREBF1, TJP2
PEDF Signaling	2,2E-01	8,33E-02	2,646	CASP8, FASLG, IKBKE, PIK3CG, PIK3R1, PIK3R5, TCF7
Dopamine-DARPP32 Feedback in cAMP Signaling	2,3E-01	7,39E-02	2,530	ADCY7, ATP2A2, ATP2A3, CAMK4, DRD4, KCNJ15, KCNJ5, PLCB2, PPM1J, PPP1CC, PPP2R2B, PRKCB, PRKCQ
Ovarian Cancer Signaling	2,3E-01	7,53E-02		BRCA2, CDKN2A, CGA, LEF1, MMP9, PIK3CG, PIK3R1, PIK3R5, WNT10A, WNT3, WNT3A
LPS-stimulated MAPK Signaling	2,4E-01	8,14E-02	1,890	CDC42, IKBKE, PIK3CG, PIK3R1, PIK3R5, PRKCB, PRKCQ
TNFR2 Signaling	2,4E-01	1,03E-01		IKBKE, TNFAIP3, TRAF1
Role of RIG1-like Receptors in Antiviral Innate Immunity	2,4E-01	9,3E-02	1,000	CASP8, DHX58, IFIH1, IKBKE
Role of p14/p19ARF in Tumor Suppression	2,4E-01	9,3E-02	-1,000	CDKN2A, PIK3CG, PIK3R1, PIK3R5
Phospholipases	2,4E-01	8,33E-02		PLA2G4F, PLA2G7, PLAAT4, PLB1, PLCB2, PLD4
RANK Signaling in Osteoclasts	2,5E-01	7,84E-02	2,646	CAMK4, IKBKE, NFATC2, PIK3CG, PIK3R1, PIK3R5, PTK2B, TRAF5
HMGB1 Signaling	2,5E-01	7,52E-02	1,897	CDC42, FNBP1, IFNG, IL18, IL4, PIK3CG, PIK3R1, PIK3R5, RHOD, RHOH
PAK Signaling	2,5E-01	7,77E-02	1,414	CDC42, ITGA4, NCK2, PIK3CG, PIK3R1, PIK3R5, PTK2, PTK2B
Airway Inflammation in Asthma	2,6E-01	0,2		IL4
Anandamide Degradation	2,6E-01	0,2		FAAH
Chronic Myeloid Leukemia Signaling	2,6E-01	7,69E-02		CDKN2A, E2F2, HDAC10, IKBKE, PIK3CG, PIK3R1, PIK3R5, TGFBR2
UDP-N-acetyl-D-glucosamine Biosynthesis II	2,6E-01	1,18E-01		FMO3, UAP1
Apoptosis Signaling	2,6E-01	7,87E-02	-0,378	BCL2L11, CASP8, CYCS, ENDOG, FASLG, IKBKE, PRKCQ

Rac Signaling	2,7E-01	7,5E-02	1,667	CDC42, ITGA4, NCF2, PIK3CG, PIK3R1, PIK3R5, PIP4K2A, PTK2, PTK2B
4-1BB Signaling in T Lymphocytes	2,7E-01	9,68E-02		IKBKE, TNFRSF9, TRAF1
SAPK/JNK Signaling	2,7E-01	7,62E-02	2,121	CDC42, LCK, MAP4K1, PIK3CG, PIK3R1, PIK3R5, RAC2, SH2D2A
eNOS Signaling	2,8E-01	7,14E-02	2,111	ADCY7, AQP7, CAMK4, CASP8, HSPA6, KNG1, LPAR2, PIK3CG, PIK3R1, PIK3R5, PRKCB, PRKCQ
FXR/RXR Activation	2,8E-01	7,3E-02		APOA1, FBP1, IL18, KNG1, MLXIPL, PKLR, SDC1, SERPINA1, SLC10A2, SREBF1
tRNA Splicing	2,8E-01	8,7E-02		PDE4A, PDE4D, PDE6A, PDE9A
Signaling by Rho Family GTPases	2,9E-01	6,83E-02	0,728	ARHGEF16, CDC42, CDH16, CDH9, FNBP1, ITGA4, NCF2, PIK3CG, PIK3R1, PIK3R5, PIP4K2A, PTK2, PTK2B, RHOD, RHOH, WAS, WIPF1
Gαi Signaling	2,9E-01	7,32E-02	1,000	ADCY7, ADORA3, CXCR2, DRD4, P2RY12, P2RY13, P2RY14, PTGER3, RGS10
Role of Tissue Factor in Cancer	3,0E-01	7,26E-02		BLK, CDC42, FGR, LCK, PDIA2, PIK3CG, PIK3R1, PIK3R5, PTK2B
ERK/MAPK Signaling	3,0E-01	6,9E-02	1,604	BCAR1, ITGA4, PIK3CG, PIK3R1, PIK3R5, PLA2G4F, PPM1J, PPP1CC, PPP2R2B, PRKCB, PTK2, PTK2B, RAC2, STAT1
Alanine Degradation III	3,0E-01	1,67E-01		GPT
Alanine Biosynthesis II	3,0E-01	1,67E-01		GPT
Antioxidant Action of Vitamin C	3,0E-01	7,34E-02	-2,121	CSF2RA, IKBKE, PLA2G4F, PLA2G7, PLAAT4, PLB1, PLCB2, PLD4
VEGF Signaling	3,0E-01	7,34E-02	0,707	PIK3CG, PIK3R1, PIK3R5, PRKCB, PTK2, PTK2B, PTPN6, SH2D2A
mTOR Signaling	3,1E-01	6,83E-02	2,138	AKT1S1, DDIT4, FNBP1, PIK3CG, PIK3R1, PIK3R5, PLD4, PPM1J, PPP2R2B, PRKCB, PRKCQ, RHEB, RHOD, RHOH
Role of JAK2 in Hormone-like Cytokine Signaling	3,2E-01	8,82E-02		PTPN6, SOCS1, STAT1
NAD biosynthesis II (from tryptophan)	3,2E-01	8,82E-02		HAAO, IDO2, KMO
Telomerase Signaling	3,2E-01	7,21E-02	1,890	HDAC10, IL2RB, IL2RG, PIK3CG, PIK3R1, PIK3R5, PPM1J, PPP2R2B
Cellular Effects of Sildenafil (Viagra)	3,3E-01	6,99E-02		ADCY7, CAMK4, KCNN2, KCNN4, MPRIP, MYH6, NPPA, PDE4A, PDE4D, PLCB2
Granzyme A Signaling	3,3E-01	0,1		GZMA, PRF1
nNOS Signaling in Skeletal Muscle Cells	3,3E-01	0,1		CAMK4, RYR1
Cell Cycle Regulation by BTG Family Proteins	3,4E-01	8,57E-02		E2F2, PPM1J, PPP2R2B
PI3K/AKT Signaling	3,4E-01	6,98E-02	0,333	IKBKE, INPP5D, ITGA4, JAK3, PIK3CG, PIK3R1, PPM1J, PPP2R2B, RHEB
Mitotic Roles of Polo-Like Kinase	3,4E-01	7,58E-02		CHEK2, KIF11, PLK2, PPM1J, PPP2R2B

UVB-Induced MAPK Signaling	3,4E-01	7,58E-02		PIK3CG, PIK3R1, PIK3R5, PRKCB, PRKCQ
Spermine Biosynthesis	3,4E-01	1,43E-01		AMD1
Choline Degradation I	3,4E-01	1,43E-01		CHDH
Spermidine Biosynthesis I	3,4E-01	1,43E-01		AMD1
Interferon Signaling	3,5E-01	8,33E-02		IFNG, SOCS1, STAT1
RhoGDI Signaling	3,5E-01	6,7E-02	0,000	ARHGAP4, ARHGAP9, ARHGDIB, ARHGEF16, CDC42, CDH16, CDH9, FNBP1, ITGA4, PIP4K2A, RHOD, RHOH
Renal Cell Carcinoma Signaling	3,6E-01	7,23E-02	0,447	CDC42, MET, PIK3CG, PIK3R1, PIK3R5, UBD
Type II Diabetes Mellitus Signaling	3,6E-01	6,76E-02	2,449	IKBKE, PIK3CG, PIK3R1, PIK3R5, PKLR, PRKCB, PRKCQ, SLC27A2, SMPD3, SOCS1
Epithelial Adherens Junction Signaling	3,6E-01	6,76E-02		CDC42, LEF1, MET, MYH6, NECTIN1, NOTCH2, TGFBR2, TUBA3C/TUBA3D, TUBB2A, WAS
14-3-3-mediated Signaling	3,7E-01	6,82E-02	1,890	AKT1S1, PIK3CG, PIK3R1, PIK3R5, PLCB2, PRKCB, PRKCQ, TUBA3C/TUBA3D, TUBB2A
D-myo-inositol (1,4,5)-Trisphosphate Biosynthesis	3,7E-01	8,11E-02		PIP4K2A, PLCB2, PLD4
Huntington's Disease Signaling	3,7E-01	6,48E-02	2,309	CACNA1B, CASP8, CYCS, GRM1, HDAC10, HSPA6, IGF1, PENK, PIK3CG, PIK3R1, PIK3R5, PLCB2, PRKCB, PRKCQ, RPH3A, UBD
p38 MAPK Signaling	3,8E-01	6,84E-02	2,121	FASLG, IL18, IL18RAP, IRAK3, MAP4K1, PLA2G4F, STAT1, TGFBR2
Airway Pathology in Chronic Obstructive Pulmonary Disease	3,8E-01	1,25E-01		MMP9
Retinoate Biosynthesis II	3,8E-01	1,25E-01		RBP5
Glycerol-3-phosphate Shuttle	3,8E-01	1,25E-01		GPD1
Tight Junction Signaling	3,9E-01	6,59E-02		CDC42, CLDN14, CLDN3, EPB41, F2RL2, MYH6, NECTIN1, PPM1J, PPP2R2B, TGFBR2, TJP2
FAK Signaling	3,9E-01	6,86E-02		BCAR1, ITGA4, PIK3CG, PIK3R1, PIK3R5, PTK2, WAS
Sumoylation Pathway	3,9E-01	6,86E-02	0,000	ARHGDIB, FASLG, FNBP1, MYB, RHOD, RHOH, ZNF217
Role of Cytokines in Mediating Communication between Immune Cells	3,9E-01	7,41E-02		IFNG, IL18, IL23A, IL4
Phagosome Maturation	3,9E-01	6,62E-02		CTSK, CTSW, HLA-A, HLA-DRB1, HLA-DRB5, NCF2, PRDX6, RAB7B, TUBA3C/TUBA3D, TUBB2A
CCR3 Signaling in Eosinophils	3,9E-01	6,67E-02		CAMK4, MPRIP, PIK3CG, PIK3R1, PIK3R5, PLA2G4F, PLCB2, PRKCB, PRKCQ
Endothelin-1 Signaling	4,0E-01	6,44E-02	2,496	ADCY7, CASP8, PIK3CG, PIK3R1, PIK3R5, PLA2G4F, PLA2G7, PLAAT4, PLB1, PLCB2, PLD4, PRKCB, PRKCQ
Inhibition of Angiogenesis by TSP1	4,0E-01	7,69E-02		MMP9, SDC1, TGFBR2

Sertoli Cell-Sertoli Cell Junction Signaling	4,0E-01	6,45E-02		BCAR1, CDC42, CLDN14, CLDN3, EPB41, ITGA4, NECTIN1, RAB8B, TJP2, TUBA3C/TUBA3D, TUBB2A, WAS
HIPPO signaling	4,1E-01	6,82E-02	0,000	PPM1J, PPP1CC, PPP2R2B, STK4, TJP2, WWC1
IL-10 Signaling	4,1E-01	6,94E-02		CCR5, IKBKE, IL10RA, IL18, IL18RAP
Transcriptional Regulatory Network in Embryonic Stem Cells	4,2E-01	7,5E-02		EOMES, GATA4, HESX1
Inhibition of Matrix Metalloproteases	4,2E-01	7,5E-02		MMP25, MMP9, SDC1
Leptin Signaling in Obesity	4,2E-01	6,74E-02		ADCY7, GHRL, PIK3CG, PIK3R1, PIK3R5, PLCB2
GABA Receptor Signaling	4,2E-01	6,85E-02		ADCY7, GABRR3, KCNN2, KCNN4, UBD
Melanoma Signaling	4,3E-01	7,02E-02		CDKN2A, PIK3CG, PIK3R1, PIK3R5
Role of JAK family kinases in IL-6-type Cytokine Signaling	4,3E-01	0,08		SOCS1, STAT1
ErbB4 Signaling	4,4E-01	6,76E-02	2,236	PIK3CG, PIK3R1, PIK3R5, PRKCB, PRKCQ
RhoA Signaling	4,4E-01	6,45E-02	0,707	ARHGAP4, ARHGAP9, IGF1, LPAR2, MPRIP, PIP4K2A, PTK2, PTK2B
Docosahexaenoic Acid (DHA) Signaling	4,4E-01	6,9E-02		CYCS, PIK3CG, PIK3R1, PIK3R5
Glycolysis I	4,5E-01	7,14E-02		ENO3, FBP1, PKLR
UVC-Induced MAPK Signaling	4,5E-01	7,14E-02		PRKCB, PRKCQ, SMPD3
P2Y Purigenic Receptor Signaling Pathway	4,5E-01	6,34E-02	1,667	ADCY7, P2RY12, P2RY2, PIK3CG, PIK3R1, PIK3R5, PLCB2, PRKCB, PRKCQ
Embryonic Stem Cell Differentiation into Cardiac Lineages	4,5E-01	0,1		GATA4
Role of Wnt/GSK-3β Signaling in the Pathogenesis of Influenza	4,6E-01	6,58E-02		IFNG, LEF1, WNT10A, WNT3, WNT3A
Ephrin Receptor Signaling	4,6E-01	6,21E-02	1,265	ANGPT1, BCAR1, CDC42, CXCR4, ITGA4, NCK2, PIK3CG, PTK2, RAC2, WAS, WIPF1
Neuregulin Signaling	4,6E-01	6,45E-02	0,447	EREG, ITGA4, MATK, PIK3R1, PRKCB, PRKCQ
Hereditary Breast Cancer Signaling	4,7E-01	6,25E-02		BRCA2, CHEK2, FANCA, FANCD2, HDAC10, PIK3CG, PIK3R1, PIK3R5, UBD
BMP signaling pathway	4,7E-01	6,49E-02	0,447	BMP6, BMP7, CAMK4, FST, RUNX2
VEGF Family Ligand-Receptor Interactions	4,7E-01	6,38E-02	1,633	PIK3CG, PIK3R1, PIK3R5, PLA2G4F, PRKCB, PRKCQ
p53 Signaling	4,7E-01	6,31E-02	-1,134	CDKN2A, CHEK2, COQ8A, PIK3CG, PIK3R1, PIK3R5, TP53INP1
Relaxin Signaling	4,8E-01	6,17E-02	2,449	ADCY7, MMP9, PDE4A, PDE4D, PDE6A, PDE9A, PIK3CG, PIK3R1, PIK3R5, RLN2
Role of IL-17F in Allergic Inflammatory Airway Diseases	4,8E-01	6,82E-02		CCL4, CXCL10, IGF1
Cyclins and Cell Cycle Regulation	4,8E-01	6,41E-02	0,000	CDKN2A, E2F2, HDAC10, PPM1J, PPP2R2B
Heme Degradation	4,8E-01	9,09E-02		HMOX2

4-hydroxyproline Degradation I	4,8E-01	9,09E-02		HOGA1
IL-17A Signaling in Airway Cells	4,9E-01	6,33E-02	2,236	IKBKE, JAK3, PIK3CG, PIK3R1, PIK3R5
Gαs Signaling	4,9E-01	6,19E-02	-0,378	ADCY7, ADRB1, HRH2, PTGDR, PTH1R, RYR1, VIPR2
Role of NFAT in Cardiac Hypertrophy	5,0E-01	6,03E-02		ADCY7, CAMK4, GATA4, HDAC10, IGF1, PIK3CG, PIK3R1, PIK3R5, PLCB2, PRKCB, PRKCQ, TGFBR2
Melanocyte Development and Pigmentation Signaling	5,0E-01	6,19E-02	1,342	ADCY7, PIK3CG, PIK3R1, PIK3R5, PTPN6, SOX10
Clathrin-mediated Endocytosis Signaling	5,0E-01	0,06		APOA1, CDC42, F2R, IGF1, ITGB2, ITGB7, MET, PIK3CG, PIK3R1, PIK3R5, SERPINA1, UBD
CNTF Signaling	5,1E-01	6,35E-02	2,000	PIK3CG, PIK3R1, PIK3R5, STAT1
Gap Junction Signaling	5,1E-01	6,01E-02		ADCY7, ADRB1, PIK3CG, PIK3R1, PIK3R5, PLCB2, PRKCB, PRKCQ, TJP2, TUBA3C/TUBA3D, TUBB2A
PCP pathway	5,1E-01	6,35E-02		SDC1, WNT10A, WNT3, WNT3A
G Beta Gamma Signaling	5,1E-01	6,12E-02	0,816	BTK, CDC42, KCNJ5, PIK3CG, PRKCB, PRKCQ
Neurotrophin/TRK Signaling	5,2E-01	6,17E-02	1,000	CDC42, PIK3CG, PIK3R1, PIK3R5, SORCS1
Glutamate Receptor Signaling	5,2E-01	6,25E-02		CAMK4, GRIK2, GRIK3, GRM1
Adipogenesis pathway	5,3E-01	5,93E-02		BMP7, CDKN2A, DGKD, ERCC2, HDAC10, SREBF1, TCF7, TXNIP
Non-Small Cell Lung Cancer Signaling	5,4E-01	6,02E-02	1,000	CDKN2A, PIK3CG, PIK3R1, PIK3R5, STK4
Endometrial Cancer Signaling	5,4E-01	6,06E-02		LEF1, PIK3CG, PIK3R1, PIK3R5
Assembly of RNA Polymerase III Complex	5,4E-01	7,69E-02		GTF3A
Glycerol Degradation I	5,4E-01	7,69E-02		GPD1
BER pathway	5,4E-01	7,69E-02		LIG1
Glutathione Redox Reactions I	5,5E-01	6,45E-02		GPX3, PRDX6
Sonic Hedgehog Signaling	5,5E-01	6,45E-02		GLIS1, GLIS2
Tryptophan Degradation III (Eukaryotic)	5,5E-01	6,12E-02		HAAO, IDO2, KMO
Cell Cycle: G2/M DNA Damage Checkpoint Regulation	5,5E-01	6,12E-02		AURKA, CDKN2A, CHEK2
IL-17 Signaling	5,6E-01	5,88E-02		CXCL10, CXCL11, PIK3CG, PIK3R1, PIK3R5
Ceramide Degradation	5,7E-01	7,14E-02		NAAA
Glycoaminoglycan-protein Linkage Region Biosynthesis	5,7E-01	7,14E-02		B3GAT1
Oncostatin M Signaling	6,0E-01	5,88E-02		JAK3, STAT1
Parkinson's Signaling	6,2E-01	6,25E-02		CYCS
CMP-N-acetylneuraminate Biosynthesis I (Eukaryotes)	6,4E-01	5,88E-02		NANP
Adenine and Adenosine Salvage III	6,4E-01	5,88E-02		ADAT3

Pregnenolone Biosynthesis	6,4E-01	5,88E-02		MICAL1
Circadian Rhythm Signaling	1,0E+00	5,71E-02		CRY1, VIPR2
Synaptic Long Term Potentiation	1,0E+00	5,47E-02	1,633	CAMK4, GRM1, PLCB2, PPP1CC, PPP1R1A, PRKCB, PRKCQ
Amyotrophic Lateral Sclerosis Signaling	1,0E+00	5,83E-02		CYCS, GRIK2, GRIK3, IGF1, PIK3CG, PIK3R1, PIK3R5
NRF2-mediated Oxidative Stress Response	1,0E+00	4,15E-02		DNAJB5, FOSL1, NQO2, PIK3CG, PIK3R1, PIK3R5, PRKCB, PRKCQ
PPARα/RXRα Activation	1,0E+00	4,37E-02	0,000	ADCY7, APOA1, GPD1, IKBKE, IL18RAP, PLCB2, PRKCB, TGFBR2
Aryl Hydrocarbon Receptor Signaling	1,0E+00	3,42E-02		CDKN2A, CHEK2, FASLG, NQO2, TFF1
LPS/IL-1 Mediated Inhibition of RXR Function	1,0E+00	3,96E-02		ABCC3, ABCG1, CHST1, CHST15, FMO3, IL18, IL18RAP, SLC27A2, SREBF1
Mitochondrial Dysfunction	1,0E+00	3,19E-02		ATP5MC1, CASP8, COX5A, CYCS, FURIN, LRRK2
PXR/RXR Activation	1,0E+00	1,43E-02		ABCC3
TR/RXR Activation	1,0E+00	5,71E-02		ADRB1, CAMK4, PIK3CG, PIK3R1, PIK3R5, SREBF1
RAR Activation	1,0E+00	3,55E-02		ADCY7, ERCC2, PIK3CG, PIK3R1, PRKCB, PRKCQ, RBP5
α-Adrenergic Signaling	1,0E+00	5,15E-02	1,342	ADCY7, CAMK4, PRKCB, PRKCQ, PYGM
MIF Regulation of Innate Immunity	1,0E+00	4,55E-02		CD74, PLA2G4F
IL-22 Signaling	1,0E+00	4,17E-02		STAT1
Mechanisms of Viral Exit from Host Cells	1,0E+00	4,88E-02		PRKCB, PRKCQ
HIF1α Signaling	1,0E+00	5,13E-02		EGLN1, MMP25, MMP9, PIK3CG, PIK3R1, PIK3R5
Melatonin Signaling	1,0E+00	5,19E-02	2,000	CAMK4, PLCB2, PRKCB, PRKCQ
Cardiac Hypertrophy Signaling	1,0E+00	5,33E-02	1,941	ADCY7, ADRB1, CAMK4, FNBP1, GATA4, IGF1, PIK3CG, PIK3R1, PIK3R5, PLCB2, RHOD, RHOH, TGFBR2
CDK5 Signaling	1,0E+00	3,88E-02	1,000	ADCY7, PPM1J, PPP1CC, PPP2R2B
Corticotropin Releasing Hormone Signaling	1,0E+00	4,13E-02	2,000	ADCY7, CAMK4, FASLG, PRKCB, PRKCQ
Maturity Onset Diabetes of Young (MODY) Signaling	1,0E+00	0,04		PKLR
GNRH Signaling	1,0E+00	4,44E-02	0,816	ADCY7, CDC42, PLCB2, PRKCB, PRKCQ, PTK2
ATM Signaling	1,0E+00	3,75E-02		CHEK2, FANCD2, PPP2R2B
Antiproliferative Role of Somatostatin Receptor 2	1,0E+00	5,06E-02	2,000	PIK3CG, PIK3R1, PIK3R5, PTPN6
Androgen Signaling	1,0E+00	4,39E-02	1,000	CAMK4, ERCC2, PRKCB, PRKCQ, SHBG
Aldosterone Signaling in Epithelial Cells	1,0E+00	5,11E-02	2,646	DNAJB5, HSPA6, PIK3CG, PIK3R1, PIK3R5, PIP4K2A, PLCB2, PRKCB, PRKCQ
CREB Signaling in Neurons	1,0E+00	5,73E-02	2,111	ADCY7, CAMK4, GRIK2, GRIK3, GRM1, PIK3CG, PIK3R1, PIK3R5, PLCB2, PRKCB, PRKCQ
Prostate Cancer Signaling	1,0E+00	4,08E-02		LEF1, PIK3CG, PIK3R1, PIK3R5

Thyroid Cancer Signaling	1,0E+00	2,44E-02		LEF1
Bladder Cancer Signaling	1,0E+00	3,45E-02		CDKN2A, MMP25, MMP9
ERK5 Signaling	1,0E+00	3,12E-02		FOSL1, SH2D2A
G Protein Signaling Mediated by Tubby	1,0E+00	5,71E-02		LCK, PLCB2
EIF2 Signaling	1,0E+00	2,68E-02	0,816	PIK3CG, PIK3R1, PIK3R5, PPP1CC, RPL27A, SREBF1
AMPK Signaling	1,0E+00	3,4E-02	0,378	ADRB1, AKT1S1, PIK3CG, PIK3R1, PIK3R5, PPM1J, PPP2R2B
Regulation of eIF4 and p70S6K Signaling	1,0E+00	4,35E-02	1,633	EIF4EBP2, ITGA4, PIK3CG, PIK3R1, PIK3R5, PPM1J, PPP2R2B
Estrogen-Dependent Breast Cancer Signaling	1,0E+00	4,94E-02	2,000	IGF1, PIK3CG, PIK3R1, PIK3R5
IL-1 Signaling	1,0E+00	3,19E-02		ADCY7, IKBKE, IRAK3
Role of Lipids/Lipid Rafts in the Pathogenesis of Influenza	1,0E+00	4,35E-02		IFNG
Antiproliferative Role of TOB in T Cell Signaling	1,0E+00	3,85E-02		TGFBR2
Extrinsic Prothrombin Activation Pathway	1,0E+00	5,56E-02		F13A1
MIF-mediated Glucocorticoid Regulation	1,0E+00	5,71E-02		CD74, PLA2G4F
Cell Cycle Control of Chromosomal Replication	1,0E+00	5,26E-02		CHEK2, LIG1
Assembly of RNA Polymerase II Complex	1,0E+00	0,02		ERCC2
Role of IL-17A in Arthritis	1,0E+00	5,8E-02		CCL5, PIK3CG, PIK3R1, PIK3R5
IL-17A Signaling in Fibroblasts	1,0E+00	2,86E-02		IKBKE
NGF Signaling	1,0E+00	4,88E-02	1,633	CDC42, IKBKE, PIK3CG, PIK3R1, PIK3R5, SMPD3
Mouse Embryonic Stem Cell Pluripotency	1,0E+00	5,66E-02	1,633	JAK3, LEF1, PIK3CG, PIK3R1, PIK3R5, WNT3A
nNOS Signaling in Neurons	1,0E+00	5,77E-02		CAMK4, PRKCB, PRKCQ
ErbB2-ErbB3 Signaling	1,0E+00	5,8E-02	2,000	JAK3, PIK3CG, PIK3R1, PIK3R5
Estrogen-mediated S-phase Entry	1,0E+00	4,17E-02		E2F2
Heparan Sulfate Biosynthesis	1,0E+00	4,17E-02		B3GAT1, CHST1, CHST15, PRDX6
Fatty Acid Activation	1,0E+00	5,26E-02		SLC27A2
Sphingosine and Sphingosine-1-phosphate Metabolism	1,0E+00	5,26E-02		NAAA
Retinoate Biosynthesis I	1,0E+00	2,63E-02		RBP5
Thyroid Hormone Metabolism II (via Conjugation and/or Degradation)	1,0E+00	3,28E-02		LARGE1, LARGE2
Nicotine Degradation III	1,0E+00	3,39E-02		LARGE1, LARGE2
Heparan Sulfate Biosynthesis (Late Stages)	1,0E+00	3,53E-02		CHST1, CHST15, PRDX6
Ubiquinol-10 Biosynthesis (Eukaryotic)	1,0E+00	3,12E-02		MICAL1
CDP-diacylglycerol Biosynthesis I	1,0E+00	2,86E-02		LPCAT3
The Visual Cycle	1,0E+00	3,12E-02		RBP5

Melatonin Degradation I	1,0E+00	2,74E-02	LARGE1, LARGE2
γ-linolenate Biosynthesis II (Animals)	1,0E+00	4,17E-02	SLC27A2
NAD Salvage Pathway II	1,0E+00	2,86E-02	ACP5
Pyridoxal 5'-phosphate Salvage Pathway	1,0E+00	2,67E-02	PIM1, PRKCQ
Pyrimidine Deoxyribonucleotides De Novo Biosynthesis I	1,0E+00	2,44E-02	APOBEC3G
Triacylglycerol Degradation	1,0E+00	5,08E-02	CEL, FAAH, PRDX6
Choline Biosynthesis III	1,0E+00	4,55E-02	PLD4
Chondroitin Sulfate Biosynthesis	1,0E+00	4,05E-02	B3GAT1, CHST1, CHST15
Dermatan Sulfate Biosynthesis	1,0E+00	0,04	B3GAT1, CHST1, CHST15
Glycogen Degradation III	1,0E+00	0,05	PYGM
Glutathione-mediated Detoxification	1,0E+00	2,44E-02	ANPEP
1D-myo-inositol Hexakisphosphate Biosynthesis II (Mammalian)	1,0E+00	3,7E-02	INPP5D
NAD Phosphorylation and Dephosphorylation	1,0E+00	0,05	ACP5
Dermatan Sulfate Biosynthesis (Late Stages)	1,0E+00	3,85E-02	CHST1, CHST15
Phosphatidylglycerol Biosynthesis II (Non-plastidic)	1,0E+00	2,44E-02	LPCAT3
Glycogen Degradation II	1,0E+00	5,56E-02	PYGM
Nicotine Degradation II	1,0E+00	4,11E-02	FMO3, LARGE1, LARGE2
Superpathway of D-myo-inositol (1,4,5)-trisphosphate Metabolism	1,0E+00	3,12E-02	INPP5D
Purine Nucleotides Degradation II (Aerobic)	1,0E+00	2,7E-02	ADAT3
Mitochondrial L-carnitine Shuttle Pathway	1,0E+00	4,76E-02	SLC27A2
D-myo-inositol (1,3,4)-trisphosphate Biosynthesis	1,0E+00	0,04	INPP5D
Serotonin Degradation	1,0E+00	2,2E-02	LARGE1, LARGE2
Phenylalanine Degradation IV (Mammalian, via Side Chain)	1,0E+00	3,12E-02	SLC27A2
D-myo-inositol (1,4,5)-trisphosphate Degradation	1,0E+00	4,55E-02	INPP5D
Retinol Biosynthesis	1,0E+00	4,08E-02	CEL, RBP5
Purine Ribonucleosides Degradation to Ribose-1-phosphate	1,0E+00	0,05	ADAT3
Triacylglycerol Biosynthesis	1,0E+00	1,72E-02	LPCAT3
Salvage Pathways of Pyrimidine Ribonucleotides	1,0E+00	4,46E-02	APOBEC2, APOBEC3G, APOBEC3H, PIM1, PRKCQ
Gluconeogenesis I	1,0E+00	4,26E-02	ENO3, FBP1

Histamine Degradation	1,0E+00	3,03E-02		HNMT
Chondroitin Sulfate Biosynthesis (Late Stages)	1,0E+00	3,39E-02		CHST1, CHST15
Salvage Pathways of Pyrimidine Deoxyribonucleotides	1,0E+00	0,05		APOBEC3G
Adenosine Nucleotides Degradation II	1,0E+00	3,57E-02		ADAT3
Superpathway of Melatonin Degradation	1,0E+00	2,3E-02		LARGE1, LARGE2
Histidine Degradation VI	1,0E+00	4,55E-02		MICAL1
Stearate Biosynthesis I (Animals)	1,0E+00	1,82E-02		SLC27A2
UDP-N-acetyl-D-galactosamine Biosynthesis II	1,0E+00	3,85E-02		UAP1
Fatty Acid β-oxidation I	1,0E+00	2,22E-02		SLC27A2
DNA damage-induced 14-3-3σ Signaling	1,0E+00	5,26E-02		RAD1
Regulation of Cellular Mechanics by Calpain Protease	1,0E+00	3,45E-02		ITGA4, PTK2
Remodeling of Epithelial Adherens Junctions	1,0E+00	4,35E-02		MET, TUBA3C/TUBA3D, TUBB2A
STAT3 Pathway	1,0E+00	5,41E-02	-1,000	PIM1, PTPN6, SOCS1, TGFBR2
Oxidative Phosphorylation	1,0E+00	2,52E-02		ATP5MC1, COX5A, CYCS
Wnt/Ca+ pathway	1,0E+00	3,33E-02		NFATC2, PLCB2
Estrogen Receptor Signaling	1,0E+00	1,56E-02		ERCC2, RUNX2
EGF Signaling	1,0E+00	5,56E-02	2,000	PIK3CG, PIK3R1, PIK3R5, STAT1
Cell Cycle: G1/S Checkpoint Regulation	1,0E+00	4,69E-02		CDKN2A, E2F2, HDAC10
Nucleotide Excision Repair Pathway	1,0E+00	2,86E-02		ERCC2
Protein Ubiquitination Pathway	1,0E+00	3,86E-02		BAG1, DNAJB5, HLA-A, HSPA6, IFNG, UBD, UBE2L6, USP2, USP31, USP51
FGF Signaling	1,0E+00	5,32E-02	0,447	MET, PIK3CG, PIK3R1, PIK3R5, PTPN6
Endoplasmic Reticulum Stress Pathway	1,0E+00	4,76E-02		ERN1
Xenobiotic Metabolism Signaling	1,0E+00	4,48E-02		ABCC3, CAMK4, CHST1, CHST15, FMO3, NQO2, PIK3CG, PIK3R1, PIK3R5, PPM1J, PPP2R2B, PRKCB, PRKCQ
Serotonin Receptor Signaling	1,0E+00	1,89E-02		ADCY7
Insulin Receptor Signaling	1,0E+00	3,4E-02	0,447	INPP5D, PIK3CG, PIK3R1, PIK3R5, PPP1CC
Phototransduction Pathway	1,0E+00	4,92E-02		PDE6A, RGS9, RGS9BP
PPAR Signaling	1,0E+00	3,23E-02		IKBKE, IL18, IL18RAP
IGF-1 Signaling	1,0E+00	5,56E-02	0,816	IGF1, PIK3CG, PIK3R1, PIK3R5, PTK2, SOCS1
Dopamine Receptor Signaling	1,0E+00	5,49E-02		ADCY7, DRD4, PPM1J, PPP1CC, PPP2R2B
Notch Signaling	1,0E+00	5,26E-02		FURIN, NOTCH2
Hypoxia Signaling in the Cardiovascular System	1,0E+00	1,54E-02		UBE2L6

IL-6 Signaling	1,0E+00	5,51E-02	1,890	IKBKE, IL18, IL18RAP, PIK3CG, PIK3R1, PIK3R5, SOCS1
GPCR-Mediated Integration of Enteroendocrine Signaling Exemplified by an L Cell	1,0E+00	3,75E-02		ADCY7, ADRB1, PLCB2
GPCR-Mediated Nutrient Sensing in Enteroendocrine Cells	1,0E+00	5,43E-02		ADCY7, FFAR4, PLCB2, PRKCB, PRKCQ
autophagy	1,0E+00	3,12E-02		CTSK, CTSW
Cancer Drug Resistance By Drug Efflux	1,0E+00	3,57E-02		PIK3CG, PIK3R1
Calcium Signaling	1,0E+00	4,84E-02		ATP2A2, ATP2A3, CAMK4, HDAC10, MYH6, NFATC2, RYR1, TNNI2, TRPC4

P-value of the overlap between DEGs and canonical pathway gene lists was calculated with Fisher's exact test. Ratio is the percentage of DEGs in each canonical pathway
