# Supplementary material for: miRNAs may play a major role in the control of gene expression in key pathobiological processes in Chagas disease cardiomyopathy
Source: PLoS Negl Trop Dis. 2020 Dec 22;14(12):e0008889. doi: 10.1371/journal.pntd.0008889 (PMC7787679; doi:10.1371/journal.pntd.0008889)
Supplement: S3 Table — (PDF) [file pntd.0008889.s003.pdf]

S3 table. DEGs associated to biological functions and processes in CCC myocardium.

DEG classification
**Inflammation (n=361); ratio=13.2% (361/2745); p=3.4E-41**

Up-regulated genes (n = 305 | 84.5%)

ADAM8, ADORA3, AIF1, AIM2, ALOX5AP, AMPD1, ANGPT1, AOAH, APOA1, ARAP2, ARHGDIB, ASPN, BACH2, BATF, BCL11B, BCL2L11, BLK, BLNK, BMF, BTLA, C1QA, C2, C5, CA5B, CACNA2D4, CARD11, CARD9, CASP8, CCDC88B, CCL14, CCL17, CCL19, CCL28, CCL3, CCL3L3, CCL4, CCL5, CCR2, CCR5, CCR7, CD19, CD2, CD200R1, CD244, CD247, CD27, CD300C, CD300LF, CD37, CD3D, CD40LG, CD48, CD5, CD6, CD69, CD7, CD72, CD74, CD84, CD8A, CDKN2A, CEBPE, CFB, CFH, CFP, CILP, CIRBP, CLEC12A, CLEC4A, CLEC4D, CLEC4E, CLEC9A, COL1A2, COL3A1, COL4A3, COL4A4, CORO1A, COTL1, CPNE7, CSF1R, CSF2RA, CTLA4, CXCL10, CXCL11, CXCL16, CXCL9, CXCR2, CXCR3, CXCR4, CYBA, CYSLTR1, CYTIP, CYTL1, DDIT4, DEF6, DNASE1, DOCK2, DRD4, E2F2, EAF2, EBI3, EOMES, ERN1, F13A1, F2R, FANCA, FANCD2, FASLG, FCAR, FCN1, FERMT3, FGR, FLT3, FLT3LG, FMOD, FOXD1, FRZB, GCNT1, GFI1, GHRL, GINS2, GNLY, GPR132, GPR174, GPR18, GPR65, GZMA, HCLS1, HCST, HELB, HLA-A, HLA-DMA, HLA-DMB, HLA-DOA, HLA-DOB, HLA-DQB1, HLA-DRB5, HLA-E, HNMT, ICOS, IFIH1, IFNG, IGF1, IKBKE, IKZF1, IKZF3, IL10RA, IL12RB1, IL12RB2, IL16, IL18, IL18RAP, IL21R, IL23A, IL2RB, IL2RG, IL4, IL5RA, IL7, IL7R, INPP5D, IRAK3, IRF4, ITGA4, ITGAL, ITGAX, ITGB2, ITGB7, ITK, JAK3, KCNN4, KLRB1, KLRC1, KLRG1, KMO, LAT, LCK, LCP1, LCP2, LILRA2, LPAR2, LRRK2, LSP1, LTB, LTC4S, LUM, LY75, MAFB, MAP4K1, MB21D1, MCTP2, MDK, MERTK, MMP25, MMP9, MR1, MRC1, MS4A1, MS4A6A, MS4A7, MYO1F, NAAA, NCF1, NCF4, NCKAP1L, NFATC2, NLRC3, NLRC4, NLRP3, NOTCH2, P2RY12, P2RY13, PARP4, PDCD1LG2, PENK, PIK3CG, PIK3R1, PIK3R5, PILRA, PIM2, PLA2G7, PNOC, POSTN, PPP2R2B, PRDM1, PRF1, PRKCB, PRKCQ, PSTPIP1, PSTPIP2, PTAFR, PTGDR, PTK2B, PTN, PTPN22, PTPN6, PTPRC, PTPRO, PYCARD, RAC2, RGS1, RUNX3, S1PR4, SAMD9L, SASH3, SCG5, SCN9A, SDC1, SELL, SELPLG, SEMA4D, SERPINA1, SH2D1A, SH2D2A, SIGLEC10, SIT1, SLAMF1, SLAMF8, SLFN12L, SLIT2, SOCS1, SPINK5, SPN, SRCIN1, STAB1, STAT1, STAT4, STIM2, STK17B, SYK, TAGAP, TBX21, TCF7, TGFBR2, TIGIT, TLR3, TLR5, TLR6, TLR8, TNFAIP3, TNFAIP8L2, TNFRSF18, TNFRSF9, TNFSF13, TNFSF13B, TNFSF14, TNFSF8, TP53INP1, TRAF1, TRPM2, TYROBP, UBASH3A, UBE2L6, UNC93B1, VAV1, VAV3, VDR, VNN1, VSIG4, WAS, WIPF1, WNT10A, XCL1, ZAP70, ZBP1

Down-regulated genes (n = 56 | 15.5%)

ADRB1, ASB2, BMP7, C1QTNF6, CA13, CA14, CACNA1B, CACNA2D3, CD59, CD79A, CDC42, CEACAM1, CHEK2, CRY1, CTRC, EDN2, EGLN1, EREG, FAAH, FKBP4, FURIN, GABRR3, GLIS2, GNMT, GPD1, GPT, HMOX2, HRH2, IL15RA, IL17RB, JMJD6, KCNJ15, KNG1, MOG, MYH6, NPC1L1, NQO2, NR4A3, P2RX3, P2RY2, PDE4A, PIM1, PRDX6, PTGER3, PTH1R, RBM38, S100A7A, SPHK1, SYT7, TFF1, TGM6, TUBB2A, UMOD, VIPR2, WNT3, WNT3A

IFNG induced genes and Th1 response (n=148); ratio=12.6% (148/1177); p=1.0E-14

Up-regulated genes (n = 125 | 84.5%)

ACP5, ADAM8, AIM1, AOAH, APOA1, BTLA, C1QA, C2, CARD9, CCL3, CCL3L3, CCL4, CCL5, CCNG2, CCR2, CCR5, CD226, CD244, CD247, CD40LG, CD74, CDKN2A, CFB, CLEC4D, CNOT6L, COL5A1, CORO1A, CPXM2, CRTAM, CTLA4, CXCL10, CXCL11, CXCL9, CXCR2, CXCR3, CXCR4, DPT, EBI3, FAM105A, FAM65B, FASLG, FCHO1, FGR, FLT3LG, FNBP1, FUT4, GCNT1, GOLM1, GPR65, HELB, HLA-A, HLA-DMA, HLA-DMB, HLA-DOA, HLA-DQB1, HLA-DRB1, HLA-DRB5, HLA-E, ICOS, IFIH1, IFIT2, IFNG, IL10RA, IL12RB1, IL12RB2, IL16, IL18, IL21R, IL23A, IL2RG, IL4, IRF4, IRF8, ITGA4, ITGAL, ITGB2, ITGB7, ITK, JAK3, KCNMA1, KLRC1, KLRD1, LCP2, LEF1, MAFB, MERTK, MPEG1, MRC1, NAV1, NCF1, NFATC2, NOTCH2, PARP8, PIK3CG, PIK3R1, PLA2G7, PLAC8, PLD4, POSTN, POU2AF1, PRKCQ, PTPN22, PTPN6, PYCARD, RGS1, RNASET2, S1PR4, SAMSN1, SELL, SELPLG, SH2D1A, SIT1, SOCS1, STAB1, STAT1, STAT4, STYK1, TBX21, TLR3, TNFSF14, TRAF1, TRAF5, UBA7, UBE2L6, WNT10A

Down-regulated genes (n = 23 | 15.5%)

ATP2A2, ATP5G1, B4GALT6, CEACAM1, CYCS, FKBP4, FURIN, GPX3, HRH2, IDH2, IL15RA, KIAA1217, MOG, MPRIP, NT5DC2, PIM1, PRDX6, PTH1R, PTK2, RBM38, RPL27A, SREBF1, TUBB2A

Fibrosis (n=82); ratio=12.31% (82/666); p=3.9E-8

Up-regulated genes (n = 63 | 76.8%)

ADORA3, APOA1, BMP6, C5, CCL17, CCL5, CCR2, CCR5, CD19, CD40LG, CD74, CDKN2A, COL1A2, COL3A1, COL4A3, CSF1R, CTSK, CXCL9, CXCR3, EBI3, F2R, FASLG, FLT3, GOLM1, IFNG, IGF1, IKBKE, IL16, IL23A, IL2RB, IL2RG, IL4, IL5RA, IL7, KCNN4, LCK, LOX, LPAR2, MMP9, NCF1, NLRP3, P2RY12, PLAC8, POSTN, PRF1, PRKCB, RUNX2, SELL, SELPLG, SFRP4, SLIT2, SOCS1, STAB1, STAT1, STAT4, STK4, TGFBR2, THY1, TNFAIP3, TNFSF14, UBD, VAV3, VDR

Down-regulated genes (n = 19 | 23.2%)

ADRB1, ATP2A2, BMP7, CEL, GLIS2, HOPX, IDH2, IL17RB, KNG1, LAMA4, MYH6, PTK2, SPHK1, SREBF1, SYT7, TUBB2A, UMOD, WFDC2, WNT3A

Extracellular matrix (n=47); ratio=11.8% (47/398); p=1.2E-4

Up-regulated genes (n = 39 | 83%)

ADAM8, ANGPT1, ANPEP, APOA1, BCL2L11, CASP8, CCNG2, CD96, CDKN2A, CMA1, COL3A1, CSF1R, CTSK, ECM2, FMOD, FREM1, GHRL, GZMB, IFNG, IGF1, IL4, IL7, ITGA4, ITGAL, ITGB2, ITGB7, LCP1, MMP9, NPHP3, OLFML2B, PARVG, PIK3R1, POSTN, SMOC2, SMPD3, SPINK5, SPOCK2, THY1, VDR

Down-regulated genes (n = 8 | 17%)

BMP7, EIF4EBP2, ERCC2, FERMT2, MET, NPPA, PTK2, WNT3A

Hypertrophy (n=53); ratio=8.77% (53/604); p=0.066

Up-regulated genes (n = 30 | 56.6%)

ADORA3, AKT1S1, ANGPT1, CAMK4, CCR2, CYBA, E2F2, F2R, FASLG, FST, IGF1, IKBKE, IL18, IL4, IL7, MMP9, NCF1, NFATC2, PIK3CG, PIK3R1, POSTN, PRKCB, RUNX2, SLIT2, STAB1, TGFBR2, TLR3, TNFAIP3, VAV3, VDR

Down-regulated genes (n = 23 | 43.4%)

ADRB1, ATP2A2, BMP7, CGA, EGLN1, ENDOG, GATA4, GPX3, H2AFZ, HOPX, HRH2, LAMA4, MYH6, NPPA, NR4A3, PDE9A, PIM1, PTH1R, PTK2, RHEB, RRAD, SREBF1, TRPC4

Contraction of heart and of cardiac muscle and contractility of heart (n=32); ratio=10.6% (32/301); p=0.011

Up-regulated genes (n = 18 | 56.3%)

ADORA3, AKAP10, CXCR4, DNASE1, FASLG, GHRL, IFNG, IL18, MMP9, NCF1, PIK3CG, PRKCB, PTAFR, TLR5, TRPM2, TXNIP, VAV3, VDR

Down-regulated genes (n = 14) | 43.7%)

ADRB1, ANK2, ATP2A2, BCAR1, CACNA1B, HOPX, LAMA4, MET, MYH6, NPPA, PDE4D, PPP1R1A, PTH1R, RRAD

Nrf2 induced genes and oxidative stress (n=35); ratio=6.6% (35/530); p=0.785

Up-regulated genes (n = 23 | 65.7%)

C1orf186, C1S, CA5B, CCL17, CLEC12A, DOCK10, ECM2, F2RL2, HNMT, IPCEF1, LRRK2, MMP9, NCF2, PARP10, PIK3R1, STK4, TLR6, TNIP3, TP53INP1, TRPM2, TXNIP, VAV1, VNN1

Down-regulated genes (n = 12 | 34.3%)

ATP2A2, DUSP13, ERCC2, HMOX2, MET, NQO2, NR4A3, OR6K2, PRDX6, RNF212, TLCD1, USP31

mitochondria (n=42); ratio=2.77% (42/1515); p=2.9E-10

Up-regulated genes (n = 27 | 64.3%)

ACSM1, AKAP10, BCL2L11, CA5B, CASP8, CDKN2A, CYBA, DDIT4, ERN1, FAM65B, FAM72A, HCLS1, HEMK1, HOGA1, HSH2D, LIG1, LRRK2, PIF1, PPP2R2B, PYCARD, RAB8B, SH3BP5, SLC25A45, SLC27A2, VAMP8, XAF1, XRCC3

Down-regulated genes (n = 15 | 35.7%)

ABCG1, ADCK3, ATP5G1, COMTD1, COQ5, CRY1, CYCS, ENDOG, FKBP4, GLYAT, GPD1, GRAMD4, IDH2, PPP1CC, TRMU

Arrhythmia (n=25); ratio=9.51% (25/263); p=0.095

Up-regulated genes (n = 13 | 52%)

ADORA3, AKAP10, CAMK4, COL3A1, GHRL, KCNA4, KCNMA1, KIF21B, LCP2, MMP9, P2RY12, PIK3CG, SCN9A

Down-regulated genes (n = 12 | 48%)

ADRB1, ANK2, ASB2, ATP1A4, ATP2A2, KCNA7, KCNJ5, LAMA4, MYH6, NPC1L1, NPPA, TUBB2A
