# Supplementary material for: miRNAs may play a major role in the control of gene expression in key pathobiological processes in Chagas disease cardiomyopathy
Source: PLoS Negl Trop Dis. 2020 Dec 22;14(12):e0008889. doi: 10.1371/journal.pntd.0008889 (PMC7787679; doi:10.1371/journal.pntd.0008889)
Supplement: S4 Table — (PDF) [file pntd.0008889.s004.pdf]

S4 table. Number of DEGs shared between several pathobiological functions or processes in CCC myocardium.

	IFNG/ Th1 response	Fibrosis	Extra cellular matrix	Hypertrophy	Contraction/ contractibility	Nrf2 and oxidative stress	Mitochondria	Arrhythmia
**Inflammation**	104	64	26	34	20	16	14	13
**IFNG/Th1 response**		32	13	18	8	3	7	5
**Fibrosis**			16	27	13	4	2	9
**Extracellular matrix**				11	6	5	3	4
**Hypertrophy**					17	4	2	9
**Contraction/contractibility**						5	1	11
**Nrf2 and oxidative stress**							2	2
**Mitochondria**								1
