# Supplementary material for: miRNAs may play a major role in the control of gene expression in key pathobiological processes in Chagas disease cardiomyopathy
Source: PLoS Negl Trop Dis. 2020 Dec 22;14(12):e0008889. doi: 10.1371/journal.pntd.0008889 (PMC7787679; doi:10.1371/journal.pntd.0008889)
Supplement: S5 Table — (PDF) [file pntd.0008889.s005.pdf]

S5 table. DEGs shared between several pathobiological functions or processes in CCC myocardium.

DEGs associated to inflammation genes and IFNG/Th1 genes (n=104)

Up-regulated genes (n = 93 | 89.4%)

ADAM8, AOAH, APOA1, BTLA, C1QA, C2, CARD9, CCL3, CCL3L3, CCL4, CCL5, CCR2, CCR5, CD244, CD247, CD40LG, CD74, CDKN2A, CFB, CLEC4D, CORO1A, CTLA4, CXCL10, CXCL11, CXCL9, CXCR2, CXCR3, CXCR4, EBI3, FASLG, FGR, FLT3LG, GCNT1, GPR65, HELB, HLA-A, HLA-DMA, HLA-DMB, HLA-DOA, HLA-DQB1, HLA-DRB5, HLA-E, ICOS, IFIH1, IFNG, IL10RA, IL12RB1, IL12RB2, IL16, IL18, IL21R, IL23A, IL2RG, IL4, IRF4, ITGA4, ITGAL, ITGB2, ITGB7, ITK, JAK3, KLRC1, LCP2, MAFB, MERTK, MRC1, NCF1, NFATC2, NOTCH2, PIK3CG, PIK3R1, PLA2G7, POSTN, PRKCQ, PTPN22, PTPN6, PYCARD, RGS1, S1PR4, SELL, SELPLG, SH2D1A, SIT1, SOCS1, STAB1, STAT1, STAT4, TBX21, TLR3, TNFSF14, TRAF1, UBE2L6, WNT10A

Down-regulated genes (n = 11 | 10.6%)

CEACAM1, F-KBP4, FURIN, HRH2, IL15RA, MOG, PIM1, PRDX6, PTH1R, RBM38, TUBB2A

DEGs associated to inflammation genes and fibrosis genes (n=64)

Up-regulated genes (n = 52 | 81.3%)

ADORA3, APOA1, C5, CCL17, CCL5, CCR2, CCR5, CD19, CD40LG, CD74, CDKN2A, COL1A2, COL3A1, COL4A3, CSF1R, CXCL9, CXCR3, EBI3, F2R, FASLG, FLT3, IFNG, IGF1, IL16, IL23A, IL2RB, IL2RG, IL4, IL5RA, IL7, KCNN4, LCK, LPAR2, MMP9, NCF1, NLRP3, P2RY12, POSTN, PRF1, PRKCB, SELL, SELPLG, SLIT2, SOCS1, STAB1, STAT1, STAT4, TGFBR2, TNFAIP3, TNFSF14, VAV3, VDR

Down-regulated genes (n = 12 | 18.7%)

ADRB1, BMP7, GLIS2, I-KBKE, IL17RB, KNG1, MYH6, SPHK1, SYT7, TUBB2A, UMOD, WNT3A

DEGs associated to inflammation genes and extracellular matrix genes (n=26)

Up-regulated genes (n = 24 | 92.3%)

ADAM8, ANGPT1, APOA1, BCL2L11, CASP8, CDKN2A, COL3A1, CSF1R, FMOD, GHRL, IFNG, IGF1, IL4, IL7, ITGA4, ITGAL, ITGB2, ITGB7, LCP1, MMP9, PIK3R1, POSTN, SPINK5, VDR

Down-regulated genes (n = 2 | 7.7%)

BMP7, WNT3A

DEGs associated to inflammation genes and hypertrophy genes (n=34)

Up-regulated genes (n = 25 | 73.6%)

ADORA3, ANGPT1, CCR2, CYBA, E2F2, F2R, FASLG, IGF1, IL18, IL4, IL7, MMP9, NCF1, NFATC2, PIK3CG, PIK3R1, POSTN, PRKCB, SLIT2, STAB1, TGFBR2, TLR3, TNFAIP3, VAV3, VDR

Down-regulated genes (n = 9 | 26.4%)

ADRB1, BMP7, EGLN1, HRH2, I-KBKE, MYH6, NR4A3, PIM1, PTH1R

DEGs associated to inflammation genes and contraction/contractility genes (n=20)

Up-regulated genes (n = 16 | 80%)

ADORA3, CXCR4, DNASE1, FASLG, GHRL, IFNG, IL18, MMP9, NCF1, PIK3CG, PRKCB, PTAFR, TLR5, TRPM2, VAV3, VDR

Down-regulated genes (n = 4 | 20%)

ADRB1, CACNA1B, MYH6, PTH1R

DEGs associated to inflammation genes and Nrf2 or oxidative stress genes (n=16)

Up-regulated genes (n = 12 | 75%)

CA5B, CCL17, CLEC12A, HNMT, LRRK2, MMP9, PIK3R1, TLR6, TP53INP1, TRPM2, VAV1, VNN1

Down-regulated genes (n = 4 | 25%)

HMOX2, NQO2, NR4A3, PRDX6

DEGs associated to inflammation genes and mitochondria genes (n=14)

Up-regulated genes (n = 11 | 78.6%)

BCL2L11, CA5B, CASP8, CDKN2A, CYBA, DDIT4, ERN1, HCLS1, LRRK2, PPP2R2B, PYCARD

Down-regulated genes (n = 3 | 21.4%)

CRY1, F-KBP4, GPD1

DEGs associated to inflammation genes and arrhythmia genes (n=13)

Up-regulated genes (n = 8 | 61.5%)

ADORA3, COL3A1, GHRL, LCP2, MMP9, P2RY12, PIK3CG, SCN9A

Down-regulated genes (n = 5 | 38.5%)

ADRB1, ASB2, MYH6, NPC1L1, TUBB2A

DEGs associated to IFNG/Th1 genes and fibrosis genes (n=32)

Up-regulated genes (n = 26 | 81.3%)
ADORA3, APOA1, CCL5, CCR2, CCR5, CD40LG, CD74, CDKN2A, CXCL9, CXCR3, EBI3, FASLG, GOLM1, IFNG, IL16, IL23A, IL2RG, IL4, NCF1, PLAC8, POSTN, SELL, SOCS1, SREBF1, STAB1, STAT1, STAT4

Down-regulated genes (n = 6 | 18.7%)
ADRB1, ATP2A2, TUBB2A, IDH2, PTK2, SELPLG

DEGs associated to IFNG/Th1 genes and extracellular matrix genes (n=13)

Up-regulated genes (n = 12 | 92.3%)
ADAM8, APOA1, CCNG2, CDKN2A, IFNG, IL4, ITGA4, ITGAL, ITGB2, ITGB7, PIK3R1, POSTN

Down-regulated genes (n = 1 | 7.7%)
PTK2

DEGs associated to IFNG/Th1 genes and hypertrophy genes (n=18)

Up-regulated genes (n = 17 | 94.4%)
ATP2A2, PIK3CG, CCR2, FASLG, GPX3, HRH2, IL18, IL4, NCF1, NFATC2, PIK3R1, PIM1, POSTN, PTH1R, SREBF1, STAB1, TLR3

Down-regulated genes (n = 1 | 5.6%)
PTK2

DEGs associated to IFNG/Th1 genes and contraction/contractility genes (n=8)

Up-regulated genes (n = 6 | 75%)
CXCR4, FASLG, IFNG, IL18, NCF1, PIK3CG

Down-regulated genes (n = 2 | 25%)
ATP2A2, PTH1R

DEGs associated to IFNG/Th1 genes and Nrf2 or oxidative stress genes (n=3)

Up-regulated genes (n = 1 | 33.3%)
PIK3R1

Down-regulated genes (n = 2 | 66.7%)
ATP2A2, PRDX6

DEGs associated to IFNG/Th1 genes and mitochondria genes (n=7)

Up-regulated genes (n = 3 | 42.9%)
CDKN2A, FAM65B, PYCARD

Down-regulated genes (n = 4 | 57.1%)
ATP5G1, CYCS, F-KBP4, IDH2

DEGs associated to IFNG/Th1 genes and arrhythmia genes (n=5)

Up-regulated genes (n = 3 | 60%)
KCNMA1, LCP2, PIK3CG

Down-regulated genes (n = 2 | 40%)
ATP2A2, TUBB2A

DEGs associated to fibrosis genes and extracellular matrix genes (n=16)

Up-regulated genes (n = 13 | 81.3%)
COL3A1, MMP9, APOA1, CDKN2A, CSF1R, CTSK, IFNG, IGF1, IL4, IL7, POSTN, THY1, VDR

Down-regulated genes (n = 3 | 18.7%)
BMP7, PTK2, WNT3A

DEGs associated to fibrosis genes and hypertrophy genes (n=27)

Up-regulated genes (n = 18 | 66.7%)
ADORA3, MMP9, CCR2, F2R, FASLG, IGF1, IL4, IL7, NCF1, POSTN, PRKCB, RUNX2, SLIT2, STAB1, TGFBR2, TNFAIP3, VAV3, VDR

Down-regulated genes (n = 9 | 33.3%)
ADRB1, ATP2A2, LAMA4, MYH6, BMP7, HOPX, I-KBKE, PTK2, SREBF1

DEGs associated to fibrosis genes and contraction/contractility genes (n=13)

Up-regulated genes (n = 8 | 61.5%)
ADORA3, MMP9, FASLG, IFNG, NCF1, PRKCB, VAV3, VDR

Down-regulated genes (n = 5 | 38.5%)
ADRB1, ATP2A2, LAMA4, MYH6, HOPX

DEGs associated to fibrosis genes and Nrf2 or oxidative stress genes (n=4)

Up-regulated genes (n = 3 | 75%)
MMP9, CCL17, STK4

Down-regulated genes (n = 1 | 25%)
ATP2A2

DEGs associated to mitochondria fibrosis genes and mitochondria genes (n=2)

Up-regulated genes (n = 1 | 50%)
CDKN2A

Down-regulated genes (n = 1 | 50%)
IDH2

DEGs associated to arrhythmia fibrosis genes and arrhythmia genes (n=9)

Up-regulated genes (n = 4 | 44.4%)
ADORA3, COL3A1, MMP9, P2RY12,

Down-regulated genes (n = 5 | 55.6%)
ADRB1, ATP2A2, LAMA4, MYH6, TUBB2A

DEGs associated to extracellular matrix genes and hypertrophy genes (n=11)

Up-regulated genes (n = 8 | 72.7%)
MMP9, ANGPT1, IGF1, IL4, IL7, PIK3R1, POSTN, VDR

Down-regulated genes (n = 3 | 27.3%)
NPPA, BMP7, PTK2

DEGs associated to extracellular matrix genes and contraction/contractility genes (n=6)

Up-regulated genes (n = 4 | 66.7%)
GHRL, MMP9, IFNG, VDR

Down-regulated genes (n = 2 | 33.3%)
NPPA, MET

DEGs associated to extracellular matrix genes and Nrf2 or oxidative stress genes (n=5)

Up-regulated genes (n = 3 | 60%)
MMP9, ECM2, PIK3R1

Down-regulated genes (n = 2 | 40%)
ERCC2, MET

DEGs associated to extracellular matrix genes and mitochondria genes (n=3)

Up-regulated genes (n = 3 | 100%)
BCL2L11, CASP8, CDKN2A

DEGs associated to arrhythmia extracellular matrix genes and arrhythmia genes (n=4)

Up-regulated genes (n = 3 | 75%)
COL3A1, GHRL, MMP9

Down-regulated genes (n = 1 | 25%)
NPPA

DEGs associated to hypertrophy genes and contraction/contractility (n=17)

Up-regulated genes (n = 9 | 52.9%)
ADORA3, MMP9, PIK3CG, FASLG, IL18, NCF1, PRKCB, VAV3, VDR

Down-regulated genes (n = 8 | 47.1%)
ADRB1, ATP2A2, LAMA4, MYH6, NPPA, HOPX, PTH1R, RRAD

DEGs associated to hypertrophy genes and Nrf2 or oxidative stress genes (n=4)

Up-regulated genes (n = 2 | 50%)
MMP9, PIK3R1

Down-regulated genes (n = 2 | 50%)
ATP2A2, NR4A3

DEGs associated to hypertrophy genes and mitochondria genes (n=2)

Up-regulated genes (n = 1 | 50%)
CYBA

Down-regulated genes (n = 1 | 50%)
ENDOG

DEGs associated to hypertrophy genes and arrhythmia genes (n=9)

Up-regulated genes (n = 4 | 44.4%)
ADORA3, CAMK4, MMP9, PIK3CG

Down-regulated genes (n = 5 | 55.6%)
ADRB1, ATP2A2, LAMA4, MYH6, NPPA

DEGs associated to contraction and contractibility genes and Nrf2 or oxidative stress genes (n=5)

Up-regulated genes (n = 3 | 60%)
MMP9, TRPM2, TXNIP

Down-regulated genes (n = 2 | 40%)
ATP2A2, MET

DEGs associated to contraction/contractility genes and mitochondria genes (n=1)

Up-regulated genes (n = 1 | 100%)
AKAP10

DEGs associated to arrhythmia contraction/contractility genes and arrhythmia genes (n=11)

Up-regulated genes (n = 5 | 54.5%)
ADORA3, AKAP10, GHRL, MMP9, PIK3CG

Down-regulated genes (n = 6 | 45.5%)
ADRB1, ANK2, ATP2A2, LAMA4, MYH6, NPPA

DEGs associated to mitochondria Nrf2 or oxidative stress genes and mitochondria genes (n=2)

Up-regulated genes (n = 2 | 100%)
CA5B, LRRK2

DEGs associated to Nrf2 or oxidative stress genes and arrhythmia genes (n=2)

Up-regulated genes (n = 1 | 50%)
MMP9

Down-regulated genes (n = 1 | 50%)
ATP2A2

DEGs associated to mitochondria genes and arrhythmia genes (n=1)

Up-regulated genes (n = 1 | 100%)
AKAP10
