# Supplementary material for: miRNAs may play a major role in the control of gene expression in key pathobiological processes in Chagas disease cardiomyopathy
Source: PLoS Negl Trop Dis. 2020 Dec 22;14(12):e0008889. doi: 10.1371/journal.pntd.0008889 (PMC7787679; doi:10.1371/journal.pntd.0008889)
Supplement: S6 Table — (PDF) [file pntd.0008889.s006.pdf]

S6 table. Cytokines and chemokines differentially expressed in CCC heart tissue.

Gene	Gene name	Expr Fold Change	Corrected p-value
**CCL5**	C-C motif chemokine ligand 5/RANTES	77.89	3.17E-05
**CXCL9**	C-X-C motif chemokine ligand 9/MIG	64.05	3.94E-04
**CCL4**	C-C motif chemokine ligand 4/MIP-1β	20.66	6.28E-06
**XCL1**	X-C motif chemokine ligand 1/Lymphotaxin	19.99	4.38E-04
**CCL17**	C-C motif chemokine ligand 17/TARC	18.55	1.40E-04
**CCL19**	C-C motif chemokine ligand 19/ MIP-3β	16.14	1.76E-02
**CXCL11**	C-X-C motif chemokine ligand 11/I-TAC	14.71	2.07E-02
**CXCL10**	C-X-C motif chemokine ligand 10/IP10	12.85	1.71E-02
**TNFSF14**	HVEML/LIGHT- TNF superfamily member 14	8.94	6.00E-03
**LTB**	lymphotoxin beta	8.56	1.35E-02
**IL7**	interleukin 7	6.23	2.24E-03
**CCL3**	C-C motif chemokine ligand 3/ MIP-1α	6.04	1.36E-02
**IFNG**	Interferon gamma	5.86	6.86E-03
**IL16**	interleukin 16	5.72	1.53E-04
**CCL3L3**	C-C motif chemokine ligand 3 like 3	5.43	1.68E-03
**TNFSF13B**	BAFF	5.40	5.53E-03
**EBI3**	IL-27B /Epstein-Barr virus induced 3	5.27	1.19E-02
**IL18**	interleukin 18	4.81	1.27E-02
**CCL28**	C-C motif chemokine ligand 28	4.51	3.46E-03
**CCL14**	C-C motif chemokine ligand 14	4.11	2.45E-02
**C5**	complement C5	3.81	1.11E-02
**IL23A**	interleukin 23 subunit alpha	3.23	7.08E-04
**CXCL16**	C-X-C motif chemokine ligand 16	3.05	3.62E-02
**TNFSF8**	TNF superfamily member 8	2.66	2.96E-02
**CYTL1**	cytokine like 1	2.41	7.99E-03
**IL4**	interleukin 4	2.34	2.90E-02
**TNFSF13**	APRIL/TNF superfamily member 13	2.19	2.93E-02
**FLT3LG**	Flt Ligand/fms related tyrosine kinase 3 ligand	2.00	3.19E-02
