# Supplementary material for: miRNAs may play a major role in the control of gene expression in key pathobiological processes in Chagas disease cardiomyopathy
Source: PLoS Negl Trop Dis. 2020 Dec 22;14(12):e0008889. doi: 10.1371/journal.pntd.0008889 (PMC7787679; doi:10.1371/journal.pntd.0008889)
Supplement: S7 Table — (PDF) [file pntd.0008889.s007.pdf]

S7 table. DEMs in CCC myocardium.

Significant miRNAs			
**genes**	**P value**	**Adjusted p value**	**Fold Change**
hsa-miR-146a-5p	3.3E-05	6.9E-03	3.9
hsa-miR-155-5p	6.5E-05	6.9E-03	3.8
hsa-miR-193a-5p	4.7E-02	1.2E-01	-1.7
hsa-miR-409-3p	4.2E-02	1.2E-01	-1.8
hsa-miR-24-3p	3.9E-02	1.2E-01	-1.8
hsa-miR-93-5p	4.3E-02	1.2E-01	-1.8
hsa-miR-92a-3p	2.7E-02	1.0E-01	-1.9
hsa-miR-192-5p	3.0E-02	1.0E-01	-1.9
hsa-miR-127-3p	4.0E-02	1.2E-01	-1.9
hsa-miR-30c-5p	4.3E-02	1.2E-01	-1.9
hsa-miR-26a-5p	3.0E-02	1.0E-01	-1.9
hsa-miR-532-5p	3.6E-02	1.1E-01	-1.9
hsa-miR-376c-3p	1.9E-02	9.7E-02	-2.0
hsa-miR-486-5p	4.6E-02	1.2E-01	-2.0
hsa-miR-25-3p	4.5E-02	1.2E-01	-2.0
hsa-miR-95-3p	4.0E-02	1.2E-01	-2.1
hsa-miR-152-3p	4.0E-02	1.2E-01	-2.1
hsa-miR-24-2-5p	2.3E-02	9.9E-02	-2.1
hsa-miR-15b-5p	9.0E-03	8.8E-02	-2.1
hsa-miR-378	3.1E-02	1.0E-01	-2.1
hsa-miR-20a-3p	1.5E-02	9.0E-02	-2.1
hsa-miR-744-5p	3.0E-02	1.0E-01	-2.1
hsa-miR-99a-3p	2.4E-02	1.0E-01	-2.1
hsa-miR-345-5p	1.4E-02	9.0E-02	-2.1
hsa-miR-328-3p	3.0E-02	1.0E-01	-2.1
hsa-miR-103a-3p	2.6E-02	1.0E-01	-2.1
hsa-miR-33a-3p	1.9E-02	9.7E-02	-2.2
hsa-miR-15a-5p	1.9E-02	9.7E-02	-2.2
hsa-miR-423-5p	2.2E-02	9.8E-02	-2.2
hsa-miR-411-5p	1.5E-02	9.0E-02	-2.2
hsa-miR-28-5p	2.9E-02	1.0E-01	-2.2
hsa-miR-296-5p	4.1E-02	1.2E-01	-2.2
hsa-miR-335-5p	2.1E-02	9.8E-02	-2.2
hsa-miR-29a-3p	2.3E-02	9.9E-02	-2.2
hsa-miR-125b-5p	4.6E-02	1.2E-01	-2.3
hsa-miR-340-5p	2.4E-02	1.0E-01	-2.3
hsa-miR-20b-5p	1.3E-02	9.0E-02	-2.3
hsa-miR-302b-3p	3.4E-02	1.1E-01	-2.3
hsa-miR-151a-5p	7.9E-03	8.6E-02	-2.3
hsa-miR-133b	1.3E-02	9.0E-02	-2.3
hsa-miR-106b-5p	3.0E-02	1.0E-01	-2.3
hsa-miR-379-5p	1.3E-02	9.0E-02	-2.4
hsa-miR-133a-3p	1.4E-02	9.0E-02	-2.4
hsa-miR-302d-3p	2.7E-02	1.0E-01	-2.4
hsa-miR-19b-1-5p	1.6E-02	9.3E-02	-2.5
hsa-miR-642a-5p	7.8E-03	8.6E-02	-2.5

hsa-miR-9-3p	3.9E-03	8.0E-02	-2.5
hsa-miR-145-3p	3.9E-03	8.0E-02	-2.5
hsa-miR-1233-3p	7.6E-03	8.6E-02	-2.5
hsa-miR-487b-3p	2.1E-02	9.8E-02	-2.5
hsa-miR-185-5p	2.7E-02	1.0E-01	-2.6
hsa-miR-212-3p	1.9E-02	9.7E-02	-2.6
hsa-miR-22-5p	3.8E-03	8.0E-02	-2.6
hsa-miR-422a	9.6E-03	8.8E-02	-2.6
hsa-miR-376a-3p	2.6E-03	8.0E-02	-2.6
hsa-miR-148a-3p	4.4E-02	1.2E-01	-2.6
hsa-miR-365a-3p	2.1E-03	8.0E-02	-2.7
hsa-miR-1-3p	5.0E-03	8.6E-02	-2.7
hsa-miR-324-5p	2.0E-02	9.8E-02	-2.9
hsa-miR-9-5p	3.5E-03	8.0E-02	-2.9
hsa-miR-99a-5p	1.4E-02	9.0E-02	-2.9
hsa-miR-101-3p	4.2E-02	1.2E-01	-2.9
hsa-miR-361-5p	3.1E-02	1.0E-01	-2.9
hsa-miR-27a-3p	2.8E-02	1.0E-01	-2.9
hsa-miR-455-5p	1.2E-02	9.0E-02	-3.0
hsa-miR-145-5p	2.0E-03	8.0E-02	-3.0
hsa-miR-29c-3p	1.1E-02	9.0E-02	-3.1
hsa-miR-378a-5p	2.8E-03	8.0E-02	-3.1
hsa-miR-23b-3p	1.0E-02	8.8E-02	-3.2
hsa-miR-224-5p	1.8E-02	9.7E-02	-3.2
hsa-miR-455-3p	1.5E-02	9.0E-02	-3.3
hsa-miR-203a-3p	4.2E-03	8.0E-02	-3.4
hsa-miR-494-3p	8.2E-03	8.6E-02	-3.5
hsa-miR-143-3p	9.5E-03	8.8E-02	-3.6
hsa-miR-451a	1.7E-02	9.6E-02	-3.7
hsa-miR-499a-5p	6.2E-03	8.6E-02	-3.8
hsa-miR-27b-3p	6.1E-03	8.6E-02	-4.1
hsa-miR-221-3p	6.5E-03	8.6E-02	-4.3
hsa-miR-22-3p	7.3E-03	8.6E-02	-4.7
hsa-miR-151a-3p	2.5E-02	1.0E-01	-4.9

Non significant miRNAs			
**genes**	**genes**	**genes**	**genes**
hsa-miR-598-3p	1.7E-01	2.6E-01	7.0
hsa-miR-142-3p	5.8E-02	1.4E-01	2.2
hsa-miR-146b-5p	5.4E-02	1.3E-01	2.0
hsa-miR-34a-3p	1.6E-01	2.5E-01	1.7
hsa-miR-339-5p	2.0E-01	3.1E-01	1.5
hsa-miR-1275	2.8E-01	3.8E-01	1.5
hsa-miR-489-3p	3.2E-01	4.1E-01	1.5
hsa-let-7b-5p	2.7E-01	3.7E-01	1.5
hsa-miR-150-5p	1.2E-01	2.2E-01	1.5
hsa-miR-539-5p	5.6E-01	6.4E-01	1.4
hsa-miR-140-3p	3.0E-01	3.9E-01	1.4
hsa-miR-628-3p	3.5E-01	4.5E-01	1.4
hsa-miR-766-3p	3.5E-01	4.5E-01	1.4
hsa-miR-320a	5.2E-01	6.0E-01	1.3

hsa-miR-10a-5p	6.1E-01	6.8E-01	1.3
hsa-miR-625-3p	3.7E-01	4.6E-01	1.3
hsa-miR-1260a	6.9E-01	7.5E-01	1.2
hsa-miR-454-3p	7.5E-01	8.0E-01	1.1
hsa-miR-28-3p	8.0E-01	8.6E-01	1.1
hsa-miR-664a-3p	8.5E-01	8.9E-01	1.1
hsa-miR-339-3p	8.9E-01	9.3E-01	1.0
hsa-miR-886-5p	9.4E-01	9.5E-01	1.0
hsa-let-7a-5p	9.2E-01	9.5E-01	1.0
hsa-miR-210-3p	9.6E-01	9.7E-01	1.0
hsa-miR-708-5p	9.8E-01	9.8E-01	1.0
hsa-miR-886-3p	9.9E-01	9.9E-01	-1.0
hsa-miR-16-5p	9.3E-01	9.5E-01	-1.0
hsa-let-7c-5p	9.2E-01	9.5E-01	-1.0
hsa-miR-197-3p	9.4E-01	9.5E-01	-1.0
hsa-miR-720	8.8E-01	9.2E-01	-1.0
hsa-miR-186-5p	8.4E-01	8.8E-01	-1.1
hsa-miR-7-1-3p	8.1E-01	8.6E-01	-1.1
hsa-miR-425-5p	7.1E-01	7.6E-01	-1.1
hsa-let-7e-5p	6.6E-01	7.3E-01	-1.1
hsa-miR-362-5p	7.0E-01	7.6E-01	-1.1
hsa-miR-574-3p	6.9E-01	7.6E-01	-1.1
hsa-miR-374a-5p	6.9E-01	7.6E-01	-1.1
hsa-miR-93-3p	6.4E-01	7.2E-01	-1.1
hsa-miR-130b-3p	6.5E-01	7.3E-01	-1.2
hsa-miR-769-5p	5.7E-01	6.5E-01	-1.2
hsa-miR-628-5p	5.7E-01	6.4E-01	-1.2
hsa-miR-30d-5p	4.9E-01	5.8E-01	-1.2
hsa-miR-1271-5p	4.1E-01	5.0E-01	-1.3
hsa-miR-511-5p	5.6E-01	6.4E-01	-1.3
hsa-miR-222-5p	4.9E-01	5.8E-01	-1.3
hsa-miR-483-5p	5.4E-01	6.3E-01	-1.3
hsa-miR-191-5p	3.8E-01	4.7E-01	-1.3
hsa-miR-135b-5p	4.9E-01	5.8E-01	-1.3
hsa-miR-1180-3p	4.8E-01	5.8E-01	-1.3
hsa-miR-222-3p	3.9E-01	4.8E-01	-1.3
hsa-miR-21-5p	5.2E-01	6.1E-01	-1.3
hsa-miR-30a-5p	3.6E-01	4.6E-01	-1.3
hsa-miR-30d-3p	4.4E-01	5.3E-01	-1.3
hsa-miR-1274b	2.9E-01	3.9E-01	-1.4
hsa-miR-331-3p	2.7E-01	3.7E-01	-1.4
hsa-miR-98-5p	3.6E-01	4.6E-01	-1.4
hsa-miR-181a-2-3p	3.2E-01	4.1E-01	-1.4
hsa-miR-140-5p	2.4E-01	3.5E-01	-1.4
hsa-miR-425-3p	4.1E-01	4.9E-01	-1.4
hsa-miR-484	2.8E-01	3.8E-01	-1.4
hsa-miR-301a-3p	2.8E-01	3.8E-01	-1.4
hsa-miR-193a-3p	2.4E-01	3.5E-01	-1.4
hsa-miR-590-5p	2.2E-01	3.3E-01	-1.4
hsa-miR-340-3p	2.1E-01	3.1E-01	-1.4
hsa-miR-335-3p	3.2E-01	4.1E-01	-1.4
hsa-miR-218-5p	2.5E-01	3.6E-01	-1.4

hsa-miR-30a-3p	2.6E-01	3.7E-01	-1.4
hsa-miR-497-5p	3.1E-01	4.1E-01	-1.5
hsa-miR-126-3p	2.0E-01	3.1E-01	-1.5
hsa-miR-550a-3p	2.7E-01	3.8E-01	-1.5
hsa-miR-148b-5p	3.0E-01	3.9E-01	-1.5
hsa-miR-320b	1.4E-01	2.4E-01	-1.5
hsa-miR-125a-5p	2.2E-01	3.3E-01	-1.5
hsa-miR-181a-3p	2.4E-01	3.5E-01	-1.5
hsa-miR-374b-5p	1.4E-01	2.4E-01	-1.5
hsa-miR-214-3p	2.2E-01	3.3E-01	-1.5
hsa-miR-195-5p	2.3E-01	3.4E-01	-1.5
hsa-miR-532-3p	8.9E-02	1.7E-01	-1.5
hsa-miR-452-5p	3.0E-01	3.9E-01	-1.5
hsa-miR-126-5p	1.5E-01	2.4E-01	-1.5
hsa-miR-204-5p	3.3E-01	4.2E-01	-1.6
hsa-miR-30e-3p	1.6E-01	2.5E-01	-1.6
hsa-miR-19a-3p	8.9E-02	1.7E-01	-1.6
hsa-miR-324-3p	9.8E-02	1.8E-01	-1.6
hsa-miR-1290	1.4E-01	2.4E-01	-1.6
hsa-miR-486-3p	1.6E-01	2.6E-01	-1.6
hsa-miR-149-5p	1.4E-01	2.3E-01	-1.6
hsa-miR-1274a	2.7E-01	3.8E-01	-1.6
hsa-miR-19b-3p	7.8E-02	1.6E-01	-1.6
hsa-miR-223-3p	1.6E-01	2.5E-01	-1.7
hsa-miR-27b-5p	1.0E-01	1.9E-01	-1.7
hsa-miR-132-3p	9.3E-02	1.8E-01	-1.7
hsa-miR-17-5p	5.9E-02	1.4E-01	-1.7
hsa-let-7d-5p	1.3E-01	2.3E-01	-1.7
hsa-miR-20a-5p	6.0E-02	1.4E-01	-1.7
hsa-miR-590-3p	6.4E-02	1.4E-01	-1.7
hsa-miR-106a-5p	5.1E-02	1.3E-01	-1.7
hsa-miR-99b-5p	8.2E-02	1.6E-01	-1.7
hsa-miR-500a-5p	1.3E-01	2.3E-01	-1.7
hsa-miR-193b-3p	5.9E-02	1.4E-01	-1.7
hsa-miR-181a-5p	8.6E-02	1.7E-01	-1.7
hsa-miR-491-5p	1.0E-01	1.9E-01	-1.7
hsa-miR-144-5p	1.1E-01	2.0E-01	-1.8
hsa-let-7g-5p	7.8E-02	1.6E-01	-1.8
hsa-miR-490-3p	1.5E-01	2.4E-01	-1.8
hsa-miR-199a-3p	1.2E-01	2.1E-01	-1.8
hsa-miR-660-5p	6.4E-02	1.4E-01	-1.8
hsa-miR-495-3p	1.0E-01	1.9E-01	-1.8
hsa-miR-136-3p	7.0E-02	1.5E-01	-1.8
hsa-miR-30b-5p	6.9E-02	1.5E-01	-1.8
hsa-miR-26b-5p	5.2E-02	1.3E-01	-1.9
hsa-miR-139-5p	6.4E-02	1.4E-01	-1.9
hsa-miR-34a-5p	2.7E-01	3.8E-01	-1.9
hsa-miR-29a-5p	1.1E-01	2.0E-01	-1.9
hsa-miR-100-5p	1.1E-01	2.0E-01	-1.9
hsa-miR-194-5p	5.8E-02	1.4E-01	-2.0
hsa-miR-148b-3p	5.9E-02	1.4E-01	-2.1
hsa-miR-130a-3p	8.0E-02	1.6E-01	-2.1

hsa-miR-128-3p	6.8E-02	1.4E-01	-2.1
hsa-miR-29c-5p	5.4E-02	1.3E-01	-2.2
hsa-miR-29b-3p	6.6E-02	1.4E-01	-2.3
hsa-miR-208b-3p	5.5E-02	1.4E-01	-2.3
hsa-miR-135a-5p	1.3E-01	2.2E-01	-2.5
hsa-miR-652-3p	5.1E-02	1.3E-01	-2.5
hsa-miR-939-5p	4.0E-01	4.8E-01	-2.5
hsa-miR-367-3p	1.3E-01	2.2E-01	-2.6
hsa-miR-214-5p	7.5E-02	1.6E-01	-2.6
hsa-miR-302c-3p	9.5E-02	1.8E-01	-2.6
hsa-miR-618	6.4E-02	1.4E-01	-2.9
hsa-miR-483-3p	7.9E-02	1.6E-01	-4.5
