# Supplementary material for: miRNAs may play a major role in the control of gene expression in key pathobiological processes in Chagas disease cardiomyopathy
Source: PLoS Negl Trop Dis. 2020 Dec 22;14(12):e0008889. doi: 10.1371/journal.pntd.0008889 (PMC7787679; doi:10.1371/journal.pntd.0008889)
Supplement: S8 Table — (PDF) [file pntd.0008889.s008.pdf]

S8 table. Correlation between DEG/DEM and the age of the patients.

	**No correlation** (pvalue > 0.05)		**Week correlation** (|corr| < 0.6 & pvalue <= 0.05)		**Moderate correlation** (0.6 <= |corr| < 0.8 & pvalue <= 0.05)		**Strong correlation** (0.8 <= |corr| & pvalue <= 0.05)	
	Number	*Percent*	*Number*	*Percent*	*Number*	*Percent*	*Number*	*Percent*
**DEG**	577	37.62%	358	23.34%	590	38.46%	9	0.59%
**DEM**	72	100%	0	100%	0	100%	0	100%

The age correlated genes are : JAKMIP1, TRIM34, RAC2, CLEC4D, GFRA2, LAIR2, CYSLTR1, GTSF1 and CD200R1.
