# Supplementary material for: miRNAs may play a major role in the control of gene expression in key pathobiological processes in Chagas disease cardiomyopathy
Source: PLoS Negl Trop Dis. 2020 Dec 22;14(12):e0008889. doi: 10.1371/journal.pntd.0008889 (PMC7787679; doi:10.1371/journal.pntd.0008889)
Supplement: S9 Table — (PDF) [file pntd.0008889.s009.pdf]

S9 table. DEM-DEG interactions in CCC myocardium.

ID	p-value	Fold Change	Source	Confidence	Symbol	p-value	Fold Change	Entrez Gene Name
hsa-miR-1-3p	5.0E-03	-2.7	TarBase	Experimentally Observed	ANPEP	7.3E-03	4.5	alanyl aminopeptidase, membrane
hsa-miR-1-3p	5.0E-03	-2.7	TarBase,TargetScan Human,miRecords	Experimentally Observed,High (predicted)	CCSAP	1.4E-02	2.5	centriole, cilia and spindle associated protein
hsa-miR-1-3p	5.0E-03	-2.7	TarBase,miRecords	Experimentally Observed	EML4	2.9E-02	2.3	echinoderm microtubule associated protein like 4
hsa-miR-1-3p	5.0E-03	-2.7	TargetScan Human,miRecords	Experimentally Observed,High (predicted)	IGF1	1.1E-02	2.7	insulin like growth factor 1
hsa-miR-1-3p	5.0E-03	-2.7	TargetScan Human	High (predicted)	MT1F	3.7E-02	2.8	metallothionein 1F
hsa-miR-1-3p	5.0E-03	-2.7	TargetScan Human	High (predicted)	NFATC2	1.6E-02	3.0	nuclear factor of activated T-cells 2
hsa-miR-1-3p	5.0E-03	-2.7	TarBase,TargetScan Human	Experimentally Observed	NOTCH2	2.4E-02	2.3	notch 2
hsa-miR-1-3p	5.0E-03	-2.7	TarBase,TargetScan Human,miRecords	Experimentally Observed	OSBPL7	3.5E-03	2.3	oxysterol binding protein like 7
hsa-miR-1-3p	5.0E-03	-2.7	TargetScan Human	High (predicted)	SAMSN1	9.6E-03	7.4	SAM domain, SH3 domain and nuclear localization signals 1
hsa-miR-1-3p	5.0E-03	-2.7	TarBase,miRecords	Experimentally Observed	SHTN1	4.0E-02	2.3	shootin 1
hsa-miR-1-3p	5.0E-03	-2.7	TarBase,miRecords	Experimentally Observed	SLC16A9	5.1E-03	5.3	solute carrier family 16 member 9
hsa-miR-1-3p	5.0E-03	-2.7	TarBase,TargetScan Human	Experimentally Observed,High (predicted)	TMSB10/TMSB4X	1.3E-02	2.0	thymosin beta 4, X-linked
hsa-miR-1-3p	5.0E-03	-2.7	TargetScan Human	High (predicted)	TNFAIP8L2	2.6E-02	4.1	TNF alpha induced protein 8 like 2
hsa-miR-1-3p	5.0E-03	-2.7	TarBase	Experimentally Observed	UNC93B1	1.3E-02	2.3	unc-93 homolog B1 (C. elegans)
hsa-miR-99a-5p	1.4E-02	-2.9	TargetScan Human	High (predicted)	NFE2L3	4.0E-02	2.4	nuclear factor, erythroid 2 like 3

hsa-miR-99a-5p	1.4E-02	-2.9	TargetScan Human	High (predicted)	RHEBL1	9.1E-03	2.4	Ras homolog enriched in brain like 1
hsa-miR-99a-5p	1.4E-02	-2.9	TargetScan Human	High (predicted)	SPN	2.0E-02	2.4	sialophorin
hsa-miR-99a-5p	1.4E-02	-2.9	TargetScan Human	High (predicted)	VNN1	3.6E-02	4.3	vanin 1
hsa-miR-101-3p	4.2E-02	-2.9	TargetScan Human	High (predicted)	AP1S3	1.6E-02	2.2	adaptor related protein complex 1 sigma 3 subunit
hsa-miR-101-3p	4.2E-02	-2.9	TargetScan Human	High (predicted)	ASPN	5.6E-03	5.0	asporin
hsa-miR-101-3p	4.2E-02	-2.9	TargetScan Human	High (predicted)	COTL1	1.8E-02	3.9	coactosin like F-actin binding protein 1
hsa-miR-101-3p	4.2E-02	-2.9	TargetScan Human	High (predicted)	DDIT4	6.6E-03	4.0	DNA damage inducible transcript 4
hsa-miR-101-3p	4.2E-02	-2.9	miRecords	Experimentally Observed	ICOS	6.7E-03	5.0	inducible T-cell costimulator
hsa-miR-101-3p	4.2E-02	-2.9	TargetScan Human	High (predicted)	MYRIP	3.9E-02	3.1	myosin VIIA and Rab interacting protein
hsa-miR-101-3p	4.2E-02	-2.9	TargetScan Human	High (predicted)	UTS2B	3.0E-02	2.5	urotensin 2B
hsa-miR-103a-3p	2.6E-02	-2.1	TargetScan Human	High (predicted)	BTLA	2.4E-03	8.6	B and T lymphocyte associated
hsa-miR-103a-3p	2.6E-02	-2.1	TargetScan Human	High (predicted)	CAPN12	2.4E-02	3.4	calpain 12
hsa-miR-103a-3p	2.6E-02	-2.1	TargetScan Human	High (predicted)	CRYBG1	2.5E-04	5.8	crystallin beta-gamma domain containing 1
hsa-miR-103a-3p	2.6E-02	-2.1	TargetScan Human	High (predicted)	GPC6	3.6E-03	4.0	glypican 6
hsa-miR-103a-3p	2.6E-02	-2.1	TargetScan Human	High (predicted)	GRAP2	2.9E-03	2.4	GRB2-related adaptor protein 2
hsa-miR-103a-3p	2.6E-02	-2.1	TargetScan Human	High (predicted)	HAUS5	2.7E-02	2.8	HAUS augmin like complex subunit 5
hsa-miR-103a-3p	2.6E-02	-2.1	TargetScan Human	High (predicted)	HELLS	2.3E-02	3.3	helicase, lymphoid-specific
hsa-miR-103a-3p	2.6E-02	-2.1	miRecords	Experimentally Observed	ICOS	6.7E-03	5.0	inducible T-cell costimulator
hsa-miR-103a-3p	2.6E-02	-2.1	TargetScan Human	High (predicted)	JAKMIP2	2.2E-03	5.4	janus kinase and microtubule interacting protein 2
hsa-miR-103a-3p	2.6E-02	-2.1	TargetScan Human	High (predicted)	KLRB1	5.8E-04	17.7	killer cell lectin like receptor B1
hsa-miR-103a-3p	2.6E-02	-2.1	TargetScan Human	High (predicted)	NPAS3	1.2E-02	2.3	neuronal PAS domain protein 3
hsa-miR-103a-3p	2.6E-02	-2.1	TargetScan Human	High (predicted)	P2RY12	1.9E-03	5.1	purinergic receptor P2Y12
hsa-miR-103a-3p	2.6E-02	-2.1	TargetScan Human	High (predicted)	PABPC1L	6.4E-03	3.9	poly(A) binding protein cytoplasmic 1 like
hsa-miR-103a-3p	2.6E-02	-2.1	TargetScan Human	High (predicted)	PPP6R2	3.4E-02	2.4	protein phosphatase 6 regulatory subunit 2
hsa-miR-103a-3p	2.6E-02	-2.1	TargetScan Human	High (predicted)	SH3BP5	3.8E-02	2.2	SH3 domain binding protein 5
hsa-miR-103a-3p	2.6E-02	-2.1	TargetScan Human	High (predicted)	SLA2	9.5E-03	5.0	Src like adaptor 2
hsa-miR-103a-3p	2.6E-02	-2.1	TargetScan Human	High (predicted)	TGFBR2	4.8E-02	3.3	transforming growth factor beta receptor 2
hsa-miR-103a-3p	2.6E-02	-2.1	TargetScan Human	High (predicted)	TMEM254	4.8E-02	2.3	transmembrane protein 254
hsa-miR-103a-3p	2.6E-02	-2.1	TargetScan Human	High (predicted)	TRANK1	3.4E-02	3.1	tetratricopeptide repeat and ankyrin repeat containing 1
hsa-miR-103a-3p	2.6E-02	-2.1	TargetScan Human	High (predicted)	TSPAN5	4.6E-03	3.5	tetraspanin 5
hsa-miR-1233-3p	7.6E-03	-2.5	TargetScan Human	High (predicted)	BMF	6.4E-03	3.1	Bcl2 modifying factor
hsa-miR-1233-3p	7.6E-03	-2.5	TargetScan Human	High (predicted)	C10orf105	1.5E-02	4.2	chromosome 10 open reading frame 105

hsa-miR-1233-3p	7.6E-03	-2.5	TargetScan Human	High (predicted)	CCL14	2.5E-02	4.1	C-C motif chemokine ligand 14
hsa-miR-1233-3p	7.6E-03	-2.5	TargetScan Human	High (predicted)	CCL28	3.5E-03	4.5	C-C motif chemokine ligand 28
hsa-miR-1233-3p	7.6E-03	-2.5	TargetScan Human	High (predicted)	CLEC4D	2.1E-03	7.9	C-type lectin domain family 4 member D
hsa-miR-1233-3p	7.6E-03	-2.5	TargetScan Human	High (predicted)	DENND2D	1.5E-03	6.2	DENN domain containing 2D
hsa-miR-1233-3p	7.6E-03	-2.5	TargetScan Human	High (predicted)	GRAP2	2.9E-03	2.4	GRB2-related adaptor protein 2
hsa-miR-1233-3p	7.6E-03	-2.5	TargetScan Human	High (predicted)	LRRC17	4.0E-04	5.4	leucine rich repeat containing 17
hsa-miR-1233-3p	7.6E-03	-2.5	TargetScan Human	High (predicted)	PIK3IP1	1.1E-03	5.6	phosphoinositide-3-kinase interacting protein 1
hsa-miR-1233-3p	7.6E-03	-2.5	TargetScan Human	High (predicted)	SH2D1A	5.7E-05	11.6	SH2 domain containing 1A
hsa-miR-1233-3p	7.6E-03	-2.5	TargetScan Human	High (predicted)	TIFAB	1.2E-03	16.9	TIFA inhibitor
hsa-miR-1233-3p	7.6E-03	-2.5	TargetScan Human	High (predicted)	UBE2L6	4.9E-02	2.1	ubiquitin conjugating enzyme E2 L6
hsa-miR-125b-5p	4.6E-02	-2.3	TargetScan Human	High (predicted)	ATP10D	2.2E-03	2.9	ATPase phospholipid transporting 10D (putative)
hsa-miR-125b-5p	4.6E-02	-2.3	TargetScan Human	High (predicted)	BIN2	3.4E-04	11.5	bridging integrator 2
hsa-miR-125b-5p	4.6E-02	-2.3	TargetScan Human,miRecords	Experimentally Observed,High (predicted)	BMF	6.4E-03	3.1	Bcl2 modifying factor
hsa-miR-125b-5p	4.6E-02	-2.3	Ingenuity Expert Findings,TargetScan Human	Experimentally Observed,High (predicted)	CCR5	7.6E-04	13.5	C-C motif chemokine receptor 5 (gene/pseudogene)
hsa-miR-125b-5p	4.6E-02	-2.3	TargetScan Human	High (predicted)	CD69	1.3E-05	20.0	CD69 molecule
hsa-miR-125b-5p	4.6E-02	-2.3	miRecords	Experimentally Observed	CDKN2A	4.8E-03	4.7	cyclin dependent kinase inhibitor 2A
hsa-miR-125b-5p	4.6E-02	-2.3	TargetScan Human	High (predicted)	GCNT1	4.5E-03	4.0	glucosaminyl (N-acetyl) transferase 1, core 2
hsa-miR-125b-5p	4.6E-02	-2.3	TargetScan Human	High (predicted)	GMIP	5.3E-03	4.1	GEM interacting protein
hsa-miR-125b-5p	4.6E-02	-2.3	TargetScan Human,miRecords	Experimentally Observed	GPR160	3.0E-03	2.7	G protein-coupled receptor 160
hsa-miR-125b-5p	4.6E-02	-2.3	TargetScan Human	High (predicted)	IRF4	3.5E-03	6.0	interferon regulatory factor 4
hsa-miR-125b-5p	4.6E-02	-2.3	TargetScan Human	High (predicted)	KLHL6	2.0E-02	4.5	kelch like family member 6
hsa-miR-125b-5p	4.6E-02	-2.3	TargetScan Human	High (predicted)	NPL	4.6E-02	2.8	N-acetylneuraminate pyruvate lyase
hsa-miR-125b-5p	4.6E-02	-2.3	TargetScan Human	High (predicted)	PRDM1	6.8E-05	26.8	PR/SET domain 1
hsa-miR-125b-5p	4.6E-02	-2.3	TargetScan Human	High (predicted)	PSTPIP2	8.7E-03	4.5	proline-serine-threonine phosphatase interacting protein 2
hsa-miR-125b-5p	4.6E-02	-2.3	TargetScan Human	High (predicted)	PTPN18	7.6E-03	2.4	protein tyrosine phosphatase, non-receptor type 18
hsa-miR-125b-5p	4.6E-02	-2.3	TarBase	Experimentally Observed	RHEBL1	9.1E-03	2.4	Ras homolog enriched in brain like 1

hsa-miR-125b-5p	4.6E-02	-2.3	TargetScan Human	High (predicted)	SEMA4D	3.0E-03	8.8	semaphorin 4D
hsa-miR-125b-5p	4.6E-02	-2.3	TargetScan Human	High (predicted)	SLA2	9.5E-03	5.0	Src like adaptor 2
hsa-miR-125b-5p	4.6E-02	-2.3	TargetScan Human	High (predicted)	THEMIS2	1.5E-02	4.0	thymocyte selection associated family member 2
hsa-miR-125b-5p	4.6E-02	-2.3	TargetScan Human	High (predicted)	TIFAB	1.2E-03	16.9	TIFA inhibitor
hsa-miR-125b-5p	4.6E-02	-2.3	TargetScan Human	High (predicted)	TNFAIP3	8.7E-03	4.5	TNF alpha induced protein 3
hsa-miR-125b-5p	4.6E-02	-2.3	TargetScan Human	High (predicted)	ZNF624	3.7E-02	2.2	zinc finger protein 624
hsa-miR-125b-5p	4.6E-02	-2.3	TargetScan Human	High (predicted)	ZSWIM5	5.5E-03	2.7	zinc finger SWIM-type containing 5
hsa-miR-127-3p	4.0E-02	-1.9	TargetScan Human	High (predicted)	HLA-DOA	5.4E-03	5.6	major histocompatibility complex, class II, DO alpha
hsa-miR-127-3p	4.0E-02	-1.9	TargetScan Human	High (predicted)	PITX1	4.7E-02	3.5	paired like homeodomain 1
hsa-miR-127-3p	4.0E-02	-1.9	miRecords	Experimentally Observed	PRDM1	6.8E-05	26.8	PR/SET domain 1
hsa-miR-127-3p	4.0E-02	-1.9	TargetScan Human	High (predicted)	SDC1	1.3E-02	3.7	syndecan 1
hsa-miR-127-3p	4.0E-02	-1.9	TargetScan Human	High (predicted)	SMPD3	2.7E-04	19.1	sphingomyelin phosphodiesterase 3
hsa-miR-127-3p	4.0E-02	-1.9	TargetScan Human	High (predicted)	XRCC3	1.6E-02	2.0	X-ray repair cross complementing 3
hsa-miR-212-3p	1.9E-02	-2.6	TargetScan Human	High (predicted)	FOLR2	4.5E-02	3.0	folate receptor beta
hsa-miR-212-3p	1.9E-02	-2.6	TargetScan Human	High (predicted)	MELK	2.4E-02	2.3	maternal embryonic leucine zipper kinase
hsa-miR-212-3p	1.9E-02	-2.6	miRecords	Experimentally Observed	MMP9	3.6E-02	7.3	matrix metallopeptidase 9
hsa-miR-133a-3p	1.5E-02	-2.4	TargetScan Human	High (predicted)	ANKRD44	1.0E-02	3.2	ankyrin repeat domain 44
hsa-miR-133a-3p	1.5E-02	-2.4	TargetScan Human	High (predicted)	CRTAM	3.2E-05	27.8	cytotoxic and regulatory T-cell molecule
hsa-miR-133a-3p	1.5E-02	-2.4	TargetScan Human	High (predicted)	CXCL11	2.1E-02	14.7	C-X-C motif chemokine ligand 11
hsa-miR-133a-3p	1.5E-02	-2.4	TargetScan Human	High (predicted)	EDEM1	6.4E-03	2.2	ER degradation enhancing alpha-mannosidase like protein 1
hsa-miR-133a-3p	1.5E-02	-2.4	TargetScan Human	High (predicted)	GZMM	2.4E-02	2.4	granzyme M
hsa-miR-133a-3p	1.5E-02	-2.4	TargetScan Human	High (predicted)	KLRC4-KLRK1/KLRK1	4.6E-05	14.5	killer cell lectin like receptor K1
hsa-miR-133a-3p	1.5E-02	-2.4	Ingenuity Expert Findings,miRecords	Experimentally Observed	RUNX2	4.4E-04	6.0	runt related transcription factor 2
hsa-miR-133a-3p	1.5E-02	-2.4	TargetScan Human	High (predicted)	SCRG1	4.5E-02	3.3	stimulator of chondrogenesis 1
hsa-miR-133a-3p	1.5E-02	-2.4	TargetScan Human	High (predicted)	SIMC1	1.4E-02	2.2	SUMO interacting motifs containing 1
hsa-miR-143-3p	9.5E-03	-3.6	TargetScan Human	High (predicted)	C1orf162	1.5E-03	4.3	chromosome 1 open reading frame 162
hsa-miR-143-3p	9.5E-03	-3.6	TargetScan Human	High (predicted)	DENND1C	1.3E-04	8.6	DENN domain containing 1C
hsa-miR-143-3p	9.5E-03	-3.6	TargetScan Human	High (predicted)	HLA-DOA	5.4E-03	5.6	major histocompatibility complex, class II, DO alpha

hsa-miR-143-3p	9.5E-03	-3.6	TargetScan Human	High (predicted)	IL18	1.3E-02	4.8	interleukin 18
hsa-miR-143-3p	9.5E-03	-3.6	TargetScan Human	High (predicted)	MS4A14	4.7E-02	2.9	membrane spanning 4-domains A14
hsa-miR-143-3p	9.5E-03	-3.6	TargetScan Human	High (predicted)	SELL	6.5E-03	8.1	selectin L
hsa-miR-143-3p	9.5E-03	-3.6	TargetScan Human	High (predicted)	SFRP4	1.7E-02	8.3	secreted frizzled related protein 4
hsa-miR-143-3p	9.5E-03	-3.6	TargetScan Human	High (predicted)	TMEM154	3.1E-02	2.2	transmembrane protein 154
hsa-miR-143-3p	9.5E-03	-3.6	TargetScan Human	High (predicted)	TSC22D3	3.1E-04	4.9	TSC22 domain family member 3
hsa-miR-145-3p	3.9E-03	-2.5	TargetScan Human	High (predicted)	CD226	1.5E-03	6.5	CD226 molecule
hsa-miR-145-3p	3.9E-03	-2.5	TargetScan Human	High (predicted)	GFI1	3.8E-06	40.1	growth factor independent 1 transcriptional repressor
hsa-miR-145-3p	3.9E-03	-2.5	TargetScan Human	High (predicted)	GZMK	1.1E-03	31.7	granzyme K
hsa-miR-145-5p	2.0E-03	-3.0	TargetScan Human	High (predicted)	GLIS1	1.0E-03	6.2	GLIS family zinc finger 1
hsa-miR-145-5p	2.0E-03	-3.0	TargetScan Human	High (predicted)	KCNA4	1.6E-02	3.8	potassium voltage-gated channel subfamily A member 4
hsa-miR-145-5p	2.0E-03	-3.0	TargetScan Human	High (predicted)	MYO5A	2.2E-03	2.1	myosin VA
hsa-miR-145-5p	2.0E-03	-3.0	TarBase,miRecords	Experimentally Observed	PARP8	9.0E-05	5.0	poly(ADP-ribose) polymerase family member 8
hsa-miR-145-5p	2.0E-03	-3.0	TargetScan Human	High (predicted)	SRGAP2	1.7E-02	2.1	SLIT-ROBO Rho GTPase activating protein 2
hsa-miR-145-5p	2.0E-03	-3.0	TargetScan Human	High (predicted)	ZNF169	1.3E-02	2.0	zinc finger protein 169
hsa-miR-146a-5p	3.3E-05	3.9	TargetScan Human,miRecords	Experimentally Observed	CDKN3	1.4E-03	-2.4	cyclin dependent kinase inhibitor 3
hsa-miR-146a-5p	3.3E-05	3.9	TargetScan Human	High (predicted)	INO80D	8.1E-03	-2.1	INO80 complex subunit D
hsa-miR-148a-3p	4.4E-02	-2.6	TargetScan Human	High (predicted)	ARRDC3	1.1E-02	3.0	arrestin domain containing 3
hsa-miR-148a-3p	4.4E-02	-2.6	TargetScan Human	High (predicted)	C11orf65	9.5E-03	2.4	chromosome 11 open reading frame 65
hsa-miR-148a-3p	4.4E-02	-2.6	TargetScan Human	High (predicted)	CCL14	2.5E-02	4.1	C-C motif chemokine ligand 14
hsa-miR-148a-3p	4.4E-02	-2.6	TargetScan Human	High (predicted)	CEBPE	4.3E-03	3.9	CCAAT/enhancer binding protein epsilon
hsa-miR-148a-3p	4.4E-02	-2.6	TargetScan Human	High (predicted)	HLA-A	5.4E-03	2.2	major histocompatibility complex, class I, A
hsa-miR-148a-3p	4.4E-02	-2.6	TargetScan Human	High (predicted)	HLA-DQB2	3.9E-02	3.6	major histocompatibility complex, class II, DQ beta 2
hsa-miR-148a-3p	4.4E-02	-2.6	TargetScan Human	High (predicted)	MAFB	2.7E-02	3.1	MAF bZIP transcription factor B
hsa-miR-148a-3p	4.4E-02	-2.6	TargetScan Human	High (predicted)	NFE2L3	4.0E-02	2.4	nuclear factor, erythroid 2 like 3
hsa-miR-148a-3p	4.4E-02	-2.6	TargetScan Human	High (predicted)	PABPC1L	6.4E-03	3.9	poly(A) binding protein cytoplasmic 1 like
hsa-miR-148a-3p	4.4E-02	-2.6	TargetScan Human	High (predicted)	PIK3IP1	1.1E-03	5.6	phosphoinositide-3-kinase interacting protein 1
hsa-miR-148a-3p	4.4E-02	-2.6	TargetScan Human	High (predicted)	TMSB10/TMSB4X	1.3E-02	2.0	thymosin beta 4, X-linked
hsa-miR-148a-3p	4.4E-02	-2.6	TargetScan Human	High (predicted)	ZNF226	4.1E-03	2.1	zinc finger protein 226
hsa-miR-151a-3p	2.5E-02	-4.9	TargetScan Human	High (predicted)	BTLA	2.4E-03	8.6	B and T lymphocyte associated

hsa-miR-151a-3p	2.5E-02	-4.9	TargetScan Human	High (predicted)	CD48	4.5E-05	25.7	CD48 molecule
hsa-miR-151a-3p	2.5E-02	-4.9	TargetScan Human	High (predicted)	HMGN2	1.9E-02	2.1	high mobility group nucleosomal binding domain 2
hsa-miR-151a-3p	2.5E-02	-4.9	TargetScan Human	High (predicted)	SLA	1.3E-03	7.8	Src like adaptor
hsa-miR-151a-3p	2.5E-02	-4.9	TargetScan Human	High (predicted)	ZMAT1	1.3E-02	3.3	zinc finger matrin-type 1
hsa-miR-151a-5p	7.9E-03	-2.3	TargetScan Human	High (predicted)	BEND6	8.1E-03	4.8	BEN domain containing 6
hsa-miR-151a-5p	7.9E-03	-2.3	TargetScan Human	High (predicted)	FANCA	1.4E-03	5.0	Fanconi anemia complementation group A
hsa-miR-151a-5p	7.9E-03	-2.3	TargetScan Human	High (predicted)	GGACT	2.9E-02	2.2	gamma-glutamylamine cyclotransferase
hsa-miR-151a-5p	7.9E-03	-2.3	TargetScan Human	High (predicted)	GNLY	5.1E-03	6.9	granulysin
hsa-miR-151a-5p	7.9E-03	-2.3	TargetScan Human	High (predicted)	SIGLEC1	1.7E-02	4.0	sialic acid binding Ig like lectin 1
hsa-miR-155-5p	6.5E-05	3.8	TargetScan Human	High (predicted)	CDX1	2.8E-02	-2.1	caudal type homeobox 1
hsa-miR-155-5p	6.5E-05	3.8	TarBase	Experimentally Observed	MET	3.4E-03	-2.5	MET proto-oncogene, receptor tyrosine kinase
hsa-miR-155-5p	6.5E-05	3.8	TarBase	Experimentally Observed	MSI2	1.5E-02	-2.3	musashi RNA binding protein 2
hsa-miR-155-5p	6.5E-05	3.8	TarBase,TargetScan Human,miRecords	Experimentally Observed	RHEB	2.7E-02	-2.1	Ras homolog enriched in brain
hsa-miR-155-5p	6.5E-05	3.8	TargetScan Human	High (predicted)	SLCO5A1	2.5E-02	-3.5	solute carrier organic anion transporter family member 5A1
hsa-miR-155-5p	6.5E-05	3.8	TarBase,TargetScan Human	Experimentally Observed,High (predicted)	TRIP13	1.4E-02	-2.3	thyroid hormone receptor interactor 13
hsa-miR-155-5p	6.5E-05	3.8	TargetScan Human	High (predicted)	WWC1	2.1E-02	-3.4	WW and C2 domain containing 1
hsa-miR-15a-5p	1.9E-02	-2.2	TargetScan Human	High (predicted)	BTLA	2.4E-03	8.6	B and T lymphocyte associated
hsa-miR-15a-5p	1.9E-02	-2.2	TargetScan Human	High (predicted)	CNOT6L	3.9E-02	2.1	CCR4-NOT transcription complex subunit 6 like
hsa-miR-15a-5p	1.9E-02	-2.2	TargetScan Human	High (predicted)	FCRL2	4.4E-02	3.4	Fc receptor like 2
hsa-miR-15a-5p	1.9E-02	-2.2	Ingenuity Expert Findings,TargetScan Human	Experimentally Observed	FLT3	1.0E-02	5.9	fms related tyrosine kinase 3
hsa-miR-15a-5p	1.9E-02	-2.2	TargetScan Human	High (predicted)	GPR171	8.8E-06	21.6	G protein-coupled receptor 171
hsa-miR-15a-5p	1.9E-02	-2.2	TargetScan Human	High (predicted)	HLA-DQB2	3.9E-02	3.6	major histocompatibility complex, class II, DQ beta 2
hsa-miR-15a-5p	1.9E-02	-2.2	TargetScan Human	High (predicted)	HLA-F	1.7E-02	2.7	major histocompatibility complex, class I, F
hsa-miR-15a-5p	1.9E-02	-2.2	Ingenuity Expert Findings	Experimentally Observed	IGF1	1.1E-02	2.7	insulin like growth factor 1

hsa-miR-15a-5p	1.9E-02	-2.2	TargetScan Human	High (predicted)	ISLR	2.3E-02	3.5	immunoglobulin superfamily containing leucine rich repeat
hsa-miR-15a-5p	1.9E-02	-2.2	TarBase,TargetScan Human	Experimentally Observed,High (predicted)	KCNN4	3.4E-02	3.8	potassium calcium-activated channel subfamily N member 4
hsa-miR-15a-5p	1.9E-02	-2.2	TargetScan Human	High (predicted)	LAMP3	1.1E-02	14.1	lysosomal associated membrane protein 3
hsa-miR-15a-5p	1.9E-02	-2.2	miRecords	Experimentally Observed	LDAH	4.1E-02	2.4	lipid droplet associated hydrolase
hsa-miR-15a-5p	1.9E-02	-2.2	TargetScan Human	High (predicted)	LY9	2.0E-05	12.7	lymphocyte antigen 9
hsa-miR-15a-5p	1.9E-02	-2.2	TargetScan Human	High (predicted)	MTMR11	3.3E-02	2.1	myotubularin related protein 11
hsa-miR-15a-5p	1.9E-02	-2.2	TargetScan Human,miRecords	Experimentally Observed,High (predicted)	MYB	3.2E-04	11.5	MYB proto-oncogene, transcription factor
hsa-miR-15a-5p	1.9E-02	-2.2	TarBase,TargetScan Human	Experimentally Observed	NOTCH2	2.4E-02	2.3	notch 2
hsa-miR-15a-5p	1.9E-02	-2.2	TarBase,TargetScan Human	Experimentally Observed,High (predicted)	RAB30	4.3E-03	2.0	RAB30, member RAS oncogene family
hsa-miR-15a-5p	1.9E-02	-2.2	TargetScan Human	High (predicted)	SLIT2	1.6E-03	2.8	slit guidance ligand 2
hsa-miR-15a-5p	1.9E-02	-2.2	TargetScan Human	High (predicted)	SYNRG	9.2E-03	2.1	synergin gamma
hsa-miR-15a-5p	1.9E-02	-2.2	TargetScan Human	High (predicted)	TFCP2L1	3.3E-02	3.0	transcription factor CP2 like 1
hsa-miR-15a-5p	1.9E-02	-2.2	TargetScan Human	High (predicted)	TNFSF13B	5.5E-03	5.4	TNF superfamily member 13b
hsa-miR-15a-5p	1.9E-02	-2.2	TargetScan Human	High (predicted)	TRANK1	3.4E-02	3.1	tetratricopeptide repeat and ankyrin repeat containing 1
hsa-miR-15a-5p	1.9E-02	-2.2	miRecords	Experimentally Observed	WIPF1	4.8E-03	2.8	WAS/WASL interacting protein family member 1
hsa-miR-106b-5p	3.0E-02	-2.3	TargetScan Human	High (predicted)	APCDD1	4.3E-02	2.0	APC down-regulated 1
hsa-miR-106b-5p	3.0E-02	-2.3	Ingenuity Expert Findings,miRecords	Experimentally Observed	BCL2L11	3.3E-02	2.4	BCL2 like 11
hsa-miR-106b-5p	3.0E-02	-2.3	TargetScan Human	High (predicted)	CCNG2	3.8E-02	2.9	cyclin G2
hsa-miR-106b-5p	3.0E-02	-2.3	TargetScan Human	High (predicted)	CD69	1.3E-05	20.0	CD69 molecule
hsa-miR-106b-5p	3.0E-02	-2.3	TargetScan Human	High (predicted)	CTSK	9.6E-03	3.0	cathepsin K
hsa-miR-106b-5p	3.0E-02	-2.3	Ingenuity Expert Findings,TargetScan Human	Experimentally Observed	E2F2	2.7E-02	4.3	E2F transcription factor 2
hsa-miR-106b-5p	3.0E-02	-2.3	TargetScan Human	High (predicted)	NABP1	2.5E-02	3.6	nucleic acid binding protein 1
hsa-miR-106b-5p	3.0E-02	-2.3	TargetScan Human	High (predicted)	PDCD1LG2	3.3E-02	2.7	programmed cell death 1 ligand 2

hsa-miR-106b-5p	3.0E-02	-2.3	TargetScan Human	High (predicted)	RASL11B	9.4E-03	6.0	RAS like family 11 member B
hsa-miR-106b-5p	3.0E-02	-2.3	TargetScan Human	High (predicted)	STK17B	3.0E-02	3.0	serine/threonine kinase 17b
hsa-miR-106b-5p	3.0E-02	-2.3	Ingenuity Expert Findings,TarBase,TargetScan Human	Experimentally Observed,High (predicted)	TGFBR2	4.8E-02	3.3	transforming growth factor beta receptor 2
hsa-miR-185-5p	2.7E-02	-2.6	TargetScan Human	High (predicted)	AIM2	2.7E-04	34.1	absent in melanoma 2
hsa-miR-185-5p	2.7E-02	-2.6	TargetScan Human	High (predicted)	BTLA	2.4E-03	8.6	B and T lymphocyte associated
hsa-miR-185-5p	2.7E-02	-2.6	TargetScan Human	High (predicted)	CD40LG	5.8E-05	37.9	CD40 ligand
hsa-miR-185-5p	2.7E-02	-2.6	TargetScan Human	High (predicted)	CD84	1.1E-04	11.1	CD84 molecule
hsa-miR-185-5p	2.7E-02	-2.6	TargetScan Human	High (predicted)	CTSK	9.6E-03	3.0	cathepsin K
hsa-miR-185-5p	2.7E-02	-2.6	TargetScan Human	High (predicted)	CXCL16	3.6E-02	3.0	C-X-C motif chemokine ligand 16
hsa-miR-185-5p	2.7E-02	-2.6	TargetScan Human	High (predicted)	CXCL9	3.9E-04	64.1	C-X-C motif chemokine ligand 9
hsa-miR-185-5p	2.7E-02	-2.6	TargetScan Human	High (predicted)	DOK3	3.1E-02	3.0	docking protein 3
hsa-miR-185-5p	2.7E-02	-2.6	TargetScan Human	High (predicted)	LAMP5	2.8E-02	6.3	lysosomal associated membrane protein family member 5
hsa-miR-185-5p	2.7E-02	-2.6	TargetScan Human	High (predicted)	NPL	4.6E-02	2.8	N-acetylneuraminate pyruvate lyase
hsa-miR-185-5p	2.7E-02	-2.6	TargetScan Human	High (predicted)	RAB30	4.3E-03	2.0	RAB30, member RAS oncogene family
hsa-miR-185-5p	2.7E-02	-2.6	TargetScan Human	High (predicted)	SAMD9L	2.9E-02	3.0	sterile alpha motif domain containing 9 like
hsa-miR-185-5p	2.7E-02	-2.6	TargetScan Human	High (predicted)	SIMC1	1.4E-02	2.2	SUMO interacting motifs containing 1
hsa-miR-185-5p	2.7E-02	-2.6	TargetScan Human	High (predicted)	SLAMF6	1.4E-05	15.6	SLAM family member 6
hsa-miR-185-5p	2.7E-02	-2.6	TargetScan Human	High (predicted)	TIFAB	1.2E-03	16.9	TIFA inhibitor
hsa-miR-185-5p	2.7E-02	-2.6	TargetScan Human	High (predicted)	TNFSF13	2.9E-02	2.2	TNF superfamily member 13
hsa-miR-185-5p	2.7E-02	-2.6	TargetScan Human	High (predicted)	TRIM14	6.7E-04	6.8	tripartite motif containing 14
hsa-miR-185-5p	2.7E-02	-2.6	TargetScan Human	High (predicted)	TRIM66	3.5E-02	3.1	tripartite motif containing 66
hsa-miR-185-5p	2.7E-02	-2.6	TargetScan Human	High (predicted)	ZNF226	4.1E-03	2.1	zinc finger protein 226
hsa-miR-185-5p	2.7E-02	-2.6	TargetScan Human	High (predicted)	ZNF831	1.7E-05	37.4	zinc finger protein 831
hsa-miR-192-5p	3.0E-02	-1.9	TargetScan Human	High (predicted)	ADGRG6	3.6E-03	3.7	adhesion G protein-coupled receptor G6
hsa-miR-192-5p	3.0E-02	-1.9	TargetScan Human	High (predicted)	CATSPER2	4.0E-02	2.1	cation channel sperm associated 2
hsa-miR-192-5p	3.0E-02	-1.9	TargetScan Human	High (predicted)	CD48	4.5E-05	25.7	CD48 molecule
hsa-miR-192-5p	3.0E-02	-1.9	TargetScan Human	High (predicted)	JCHAIN	1.9E-06	72.7	joining chain of multimeric IgA and IgM
hsa-miR-192-5p	3.0E-02	-1.9	TargetScan Human	High (predicted)	SLAMF7	3.0E-05	41.0	SLAM family member 7
hsa-miR-193a-5p	4.7E-02	-1.7	TargetScan Human	High (predicted)	BCL2L11	3.3E-02	2.4	BCL2 like 11
hsa-miR-193a-5p	4.7E-02	-1.7	TargetScan Human	High (predicted)	C1orf186	5.4E-03	5.9	chromosome 1 open reading frame 186
hsa-miR-193a-5p	4.7E-02	-1.7	TargetScan Human	High (predicted)	CD27	6.7E-05	14.9	CD27 molecule
hsa-miR-193a-5p	4.7E-02	-1.7	TargetScan Human	High (predicted)	CSF2RA	2.1E-05	6.1	colony stimulating factor 2 receptor alpha subunit

hsa-miR-193a-5p	4.7E-02	-1.7	Ingenuity Expert Findings	Experimentally Observed	IL2RG	1.9E-05	14.9	interleukin 2 receptor subunit gamma
hsa-miR-193a-5p	4.7E-02	-1.7	TargetScan Human	High (predicted)	KLRD1	5.0E-05	16.5	killer cell lectin like receptor D1
hsa-miR-193a-5p	4.7E-02	-1.7	TargetScan Human	High (predicted)	OMD	8.6E-03	4.8	osteomodulin
hsa-miR-193a-5p	4.7E-02	-1.7	TargetScan Human	High (predicted)	TRAT1	1.2E-04	18.8	T-cell receptor associated transmembrane adaptor 1
hsa-miR-19b-1-5p	1.6E-02	-2.5	TargetScan Human	High (predicted)	CSF2RA	2.1E-05	6.1	colony stimulating factor 2 receptor alpha subunit
hsa-miR-19b-1-5p	1.6E-02	-2.5	TargetScan Human	High (predicted)	CYTIP	3.8E-05	12.1	cytohesin 1 interacting protein
hsa-miR-19b-1-5p	1.6E-02	-2.5	TargetScan Human	High (predicted)	GPR174	5.4E-03	4.6	G protein-coupled receptor 174
hsa-miR-19b-1-5p	1.6E-02	-2.5	TargetScan Human	High (predicted)	HELLS	2.3E-02	3.3	helicase, lymphoid-specific
hsa-miR-19b-1-5p	1.6E-02	-2.5	TargetScan Human	High (predicted)	P2RY12	1.9E-03	5.1	purinergic receptor P2Y12
hsa-miR-19b-1-5p	1.6E-02	-2.5	TargetScan Human	High (predicted)	PIWIL4	3.5E-04	3.1	piwi like RNA-mediated gene silencing 4
hsa-miR-19b-1-5p	1.6E-02	-2.5	TargetScan Human	High (predicted)	SLC18B1	9.9E-03	2.1	solute carrier family 18 member B1
hsa-miR-203a-3p	4.2E-03	-3.4	Ingenuity Expert Findings	Experimentally Observed	RUNX2	4.4E-04	6.0	runt related transcription factor 2
hsa-miR-20a-3p	1.5E-02	-2.1	TargetScan Human	High (predicted)	ALOX5AP	3.0E-02	4.6	arachidonate 5-lipoxygenase activating protein
hsa-miR-20a-3p	1.5E-02	-2.1	TargetScan Human	High (predicted)	BATF	1.0E-03	13.3	basic leucine zipper ATF-like transcription factor
hsa-miR-20a-3p	1.5E-02	-2.1	TargetScan Human	High (predicted)	CCL4	6.3E-06	20.7	C-C motif chemokine ligand 4
hsa-miR-20a-3p	1.5E-02	-2.1	TargetScan Human	High (predicted)	CD160	2.5E-02	3.4	CD160 molecule
hsa-miR-20a-3p	1.5E-02	-2.1	TargetScan Human	High (predicted)	CFHR3	4.0E-02	3.0	complement factor H related 3
hsa-miR-20a-3p	1.5E-02	-2.1	TargetScan Human	High (predicted)	HMGN2	1.9E-02	2.1	high mobility group nucleosomal binding domain 2
hsa-miR-20a-3p	1.5E-02	-2.1	TargetScan Human	High (predicted)	TRAF1	8.0E-04	5.1	TNF receptor associated factor 1
hsa-miR-22-3p	7.3E-03	-4.7	TargetScan Human	High (predicted)	APCDD1L	4.5E-02	6.2	APC down-regulated 1 like
hsa-miR-22-3p	7.3E-03	-4.7	TargetScan Human	High (predicted)	CSF1R	3.3E-03	4.7	colony stimulating factor 1 receptor
hsa-miR-22-3p	7.3E-03	-4.7	TargetScan Human	High (predicted)	DDIT4	6.6E-03	4.0	DNA damage inducible transcript 4
hsa-miR-22-3p	7.3E-03	-4.7	TargetScan Human	High (predicted)	DERL3	1.9E-03	3.6	derlin 3
hsa-miR-22-3p	7.3E-03	-4.7	TargetScan Human	High (predicted)	GPR132	7.4E-03	3.3	G protein-coupled receptor 132
hsa-miR-22-3p	7.3E-03	-4.7	TargetScan Human	High (predicted)	NELL2	2.5E-03	9.5	neural EGFL like 2
hsa-miR-22-3p	7.3E-03	-4.7	TargetScan Human	High (predicted)	NLRP3	1.4E-02	4.0	NLR family pyrin domain containing 3
hsa-miR-22-3p	7.3E-03	-4.7	TargetScan Human	High (predicted)	OGN	1.2E-02	7.0	osteoglycin
hsa-miR-22-3p	7.3E-03	-4.7	TargetScan Human	High (predicted)	PRKCB	4.1E-04	10.0	protein kinase C beta
hsa-miR-22-3p	7.3E-03	-4.7	TargetScan Human	High (predicted)	RAB11FIP4	4.8E-02	3.2	RAB11 family interacting protein 4
hsa-miR-22-3p	7.3E-03	-4.7	TargetScan Human	High (predicted)	RGS10	1.8E-02	3.4	regulator of G protein signaling 10

hsa-miR-22-3p	7.3E-03	-4.7	TargetScan Human	High (predicted)	TLR8	7.0E-03	7.1	toll like receptor 8
hsa-miR-22-3p	7.3E-03	-4.7	TargetScan Human	High (predicted)	ZNF594	2.2E-02	2.0	zinc finger protein 594
hsa-miR-22-3p	7.3E-03	-4.7	TargetScan Human	High (predicted)	ZNF599	2.4E-02	2.3	zinc finger protein 599
hsa-miR-22-5p	3.8E-03	-2.6	TargetScan Human	High (predicted)	JAKMIP2	2.2E-03	5.4	janus kinase and microtubule interacting protein 2
hsa-miR-22-5p	3.8E-03	-2.6	TargetScan Human	High (predicted)	LAX1	9.8E-05	17.6	lymphocyte transmembrane adaptor 1
hsa-miR-22-5p	3.8E-03	-2.6	TargetScan Human	High (predicted)	RGS1	6.3E-04	19.4	regulator of G protein signaling 1
hsa-miR-22-5p	3.8E-03	-2.6	TargetScan Human	High (predicted)	SYNRG	9.2E-03	2.1	synergin gamma
hsa-miR-22-5p	3.8E-03	-2.6	TargetScan Human	High (predicted)	ZBTB37	1.4E-02	2.4	zinc finger and BTB domain containing 37
hsa-miR-221-3p	6.5E-03	-4.3	Ingenuity Expert Findings,miRecords	Experimentally Observed	BCL2L11	3.3E-02	2.4	BCL2 like 11
hsa-miR-221-3p	6.5E-03	-4.3	TargetScan Human,miRecords	Experimentally Observed	BMF	6.4E-03	3.1	Bcl2 modifying factor
hsa-miR-221-3p	6.5E-03	-4.3	TargetScan Human	High (predicted)	CXCL11	2.1E-02	14.7	C-X-C motif chemokine ligand 11
hsa-miR-221-3p	6.5E-03	-4.3	Ingenuity Expert Findings,TargetScan Human,miRecords	Experimentally Observed	DDIT4	6.6E-03	4.0	DNA damage inducible transcript 4
hsa-miR-221-3p	6.5E-03	-4.3	TargetScan Human	High (predicted)	EVI2A	2.0E-03	6.7	ecotropic viral integration site 2A
hsa-miR-221-3p	6.5E-03	-4.3	TargetScan Human	High (predicted)	HNMT	1.9E-03	2.5	histamine N-methyltransferase
hsa-miR-221-3p	6.5E-03	-4.3	TargetScan Human	High (predicted)	MS4A1	1.2E-02	5.1	membrane spanning 4-domains A1
hsa-miR-221-3p	6.5E-03	-4.3	TargetScan Human	High (predicted)	NRK	4.6E-03	10.1	Nik related kinase
hsa-miR-221-3p	6.5E-03	-4.3	Ingenuity Expert Findings,TargetScan Human	Experimentally Observed,High (predicted)	PIK3R1	4.1E-02	2.7	phosphoinositide-3-kinase regulatory subunit 1
hsa-miR-221-3p	6.5E-03	-4.3	TargetScan Human	High (predicted)	TSC22D3	3.1E-04	4.9	TSC22 domain family member 3
hsa-miR-221-3p	6.5E-03	-4.3	TargetScan Human	High (predicted)	ZBTB37	1.4E-02	2.4	zinc finger and BTB domain containing 37
hsa-miR-224-5p	1.8E-02	-3.2	Ingenuity Expert Findings	Experimentally Observed	CD53	2.1E-03	8.2	CD53 molecule
hsa-miR-224-5p	1.8E-02	-3.2	TargetScan Human	High (predicted)	NPFFR2	7.1E-03	6.3	neuropeptide FF receptor 2
hsa-miR-224-5p	1.8E-02	-3.2	TargetScan Human	High (predicted)	PARVG	3.9E-03	5.1	parvin gamma
hsa-miR-224-5p	1.8E-02	-3.2	TargetScan Human	High (predicted)	ZNF226	4.1E-03	2.1	zinc finger protein 226
hsa-miR-23b-3p	1.0E-02	-3.2	TargetScan Human	High (predicted)	CCSAP	1.4E-02	2.5	centriole, cilia and spindle associated protein
hsa-miR-23b-3p	1.0E-02	-3.2	TargetScan Human	High (predicted)	CLDN14	5.3E-03	4.0	claudin 14
hsa-miR-23b-3p	1.0E-02	-3.2	TargetScan Human	High (predicted)	FUT4	4.2E-03	2.2	fucosyltransferase 4
hsa-miR-23b-3p	1.0E-02	-3.2	TargetScan Human	High (predicted)	GALNT12	2.3E-02	2.9	polypeptide N-acetylgalactosaminyltransferase 12

hsa-miR-23b-3p	1.0E-02	-3.2	TargetScan Human	High (predicted)	HMGN2	1.9E-02	2.1	high mobility group nucleosomal binding domain 2
hsa-miR-23b-3p	1.0E-02	-3.2	TargetScan Human	High (predicted)	IL18	1.3E-02	4.8	interleukin 18
hsa-miR-23b-3p	1.0E-02	-3.2	TargetScan Human	High (predicted)	KLRF1	1.6E-02	2.1	killer cell lectin like receptor F1
hsa-miR-23b-3p	1.0E-02	-3.2	TargetScan Human	High (predicted)	MRC1	1.6E-02	4.0	mannose receptor C-type 1
hsa-miR-23b-3p	1.0E-02	-3.2	TargetScan Human	High (predicted)	NAP1L3	2.9E-02	8.7	nucleosome assembly protein 1 like 3
hsa-miR-23b-3p	1.0E-02	-3.2	TargetScan Human	High (predicted)	RAB39B	9.2E-05	14.8	RAB39B, member RAS oncogene family
hsa-miR-23b-3p	1.0E-02	-3.2	TargetScan Human	High (predicted)	RGS18	1.6E-03	7.4	regulator of G protein signaling 18
hsa-miR-23b-3p	1.0E-02	-3.2	TargetScan Human	High (predicted)	SCG5	3.6E-02	2.9	secretogranin V
hsa-miR-23b-3p	1.0E-02	-3.2	TargetScan Human	High (predicted)	ZNF287	3.5E-02	3.3	zinc finger protein 287
hsa-miR-24-2-5p	2.3E-02	-2.1	TargetScan Human	High (predicted)	BTK	3.4E-04	7.8	Bruton tyrosine kinase
hsa-miR-24-2-5p	2.3E-02	-2.1	TargetScan Human	High (predicted)	C16orf89	1.1E-02	5.4	chromosome 16 open reading frame 89
hsa-miR-24-2-5p	2.3E-02	-2.1	TargetScan Human	High (predicted)	GPC6	3.6E-03	4.0	glypican 6
hsa-miR-24-2-5p	2.3E-02	-2.1	TargetScan Human	High (predicted)	NUAK2	4.8E-02	2.1	NUAK family kinase 2
hsa-miR-24-2-5p	2.3E-02	-2.1	TargetScan Human	High (predicted)	PIF1	8.2E-03	3.0	PIF1 5'-to-3' DNA helicase
hsa-miR-24-2-5p	2.3E-02	-2.1	TargetScan Human	High (predicted)	TRIM14	6.7E-04	6.8	tripartite motif containing 14
hsa-miR-24-2-5p	2.3E-02	-2.1	TargetScan Human	High (predicted)	UBA7	8.6E-03	2.4	ubiquitin like modifier activating enzyme 7
hsa-miR-24-3p	3.9E-02	-1.8	TargetScan Human	High (predicted)	BCL2L11	3.3E-02	2.4	BCL2 like 11
hsa-miR-24-3p	3.9E-02	-1.8	TargetScan Human	High (predicted)	BHLHE22	3.9E-03	7.5	basic helix-loop-helix family member e22
hsa-miR-24-3p	3.9E-02	-1.8	TarBase,miRecords	Experimentally Observed	CDKN2A	4.8E-03	4.7	cyclin dependent kinase inhibitor 2A
hsa-miR-24-3p	3.9E-02	-1.8	TargetScan Human	High (predicted)	CXCR2	3.1E-02	2.4	C-X-C motif chemokine receptor 2
hsa-miR-24-3p	3.9E-02	-1.8	TargetScan Human	High (predicted)	DCANP1	4.4E-03	11.1	dendritic cell associated nuclear protein
hsa-miR-24-3p	3.9E-02	-1.8	TargetScan Human	High (predicted)	DNASE1L3	3.9E-02	4.1	deoxyribonuclease 1 like 3
hsa-miR-24-3p	3.9E-02	-1.8	miRecords	Experimentally Observed	E2F2	2.7E-02	4.3	E2F transcription factor 2
hsa-miR-24-3p	3.9E-02	-1.8	TargetScan Human	High (predicted)	EDA2R	3.6E-02	3.4	ectodysplasin A2 receptor
hsa-miR-24-3p	3.9E-02	-1.8	TargetScan Human	High (predicted)	FASLG	1.5E-05	17.7	Fas ligand
hsa-miR-24-3p	3.9E-02	-1.8	TargetScan Human	High (predicted)	IFNG	6.9E-03	5.9	interferon gamma
hsa-miR-24-3p	3.9E-02	-1.8	TargetScan Human	High (predicted)	PIM2	1.2E-03	7.4	Pim-2 proto-oncogene, serine/threonine kinase
hsa-miR-24-3p	3.9E-02	-1.8	TargetScan Human	High (predicted)	SPN	2.0E-02	2.4	sialophorin
hsa-miR-24-3p	3.9E-02	-1.8	TargetScan Human	High (predicted)	TPSD1	2.3E-02	2.1	tryptase delta 1
hsa-miR-24-3p	3.9E-02	-1.8	TargetScan Human	High (predicted)	ZBTB37	1.4E-02	2.4	zinc finger and BTB domain containing 37
hsa-miR-26a-5p	3.0E-02	-1.9	TargetScan Human	High (predicted)	DUSP18	7.9E-03	2.1	dual specificity phosphatase 18
hsa-miR-26a-5p	3.0E-02	-1.9	TargetScan Human	High (predicted)	HLA-F	1.7E-02	2.7	major histocompatibility complex, class I, F

hsa-miR-26a-5p	3.0E-02	-1.9	miRecords	Experimentally Observed	TGFBR2	4.8E-02	3.3	transforming growth factor beta receptor 2
hsa-miR-26a-5p	3.0E-02	-1.9	TargetScan Human	High (predicted)	ZBTB37	1.4E-02	2.4	zinc finger and BTB domain containing 37
hsa-miR-27b-3p	6.1E-03	-4.1	TargetScan Human	High (predicted)	GOLM1	1.8E-02	2.1	golgi membrane protein 1
hsa-miR-27b-3p	6.1E-03	-4.1	Ingenuity Expert Findings	Experimentally Observed	IGF1	1.1E-02	2.7	insulin like growth factor 1
hsa-miR-27b-3p	6.1E-03	-4.1	TargetScan Human	High (predicted)	KCNA4	1.6E-02	3.8	potassium voltage-gated channel subfamily A member 4
hsa-miR-27b-3p	6.1E-03	-4.1	TargetScan Human	High (predicted)	MS4A7	3.2E-02	2.8	membrane spanning 4-domains A7
hsa-miR-27b-3p	6.1E-03	-4.1	TargetScan Human	High (predicted)	NABP1	2.5E-02	3.6	nucleic acid binding protein 1
hsa-miR-27b-3p	6.1E-03	-4.1	TargetScan Human	High (predicted)	RIPOR2	3.8E-03	4.3	RHO family interacting cell polarization regulator 2
hsa-miR-27b-3p	6.1E-03	-4.1	TargetScan Human	High (predicted)	STYK1	1.6E-05	14.7	serine/threonine/tyrosine kinase 1
hsa-miR-27b-3p	6.1E-03	-4.1	TargetScan Human	High (predicted)	TMSB10/TMSB4X	1.3E-02	2.0	thymosin beta 4, X-linked
hsa-miR-27b-3p	6.1E-03	-4.1	TargetScan Human	High (predicted)	TRAF3IP3	9.9E-06	30.7	TRAF3 interacting protein 3
hsa-miR-27b-3p	6.1E-03	-4.1	TargetScan Human	High (predicted)	YPEL1	1.0E-02	2.1	yippee like 1
hsa-miR-27b-3p	6.1E-03	-4.1	TargetScan Human	High (predicted)	ZBTB37	1.4E-02	2.4	zinc finger and BTB domain containing 37
hsa-miR-302d-3p	2.7E-02	-2.4	TargetScan Human	High (predicted)	LAMP5	2.8E-02	6.3	lysosomal associated membrane protein family member 5
hsa-miR-302d-3p	2.7E-02	-2.4	TargetScan Human	High (predicted)	PDCD1LG2	3.3E-02	2.7	programmed cell death 1 ligand 2
hsa-miR-302d-3p	2.7E-02	-2.4	TargetScan Human	High (predicted)	RTN1	8.4E-04	7.6	reticulon 1
hsa-miR-302d-3p	2.7E-02	-2.4	TarBase,miRecords	Experimentally Observed	STK4	9.1E-04	2.5	serine/threonine kinase 4
hsa-miR-302d-3p	2.7E-02	-2.4	TargetScan Human	High (predicted)	TGFBR2	4.8E-02	3.3	transforming growth factor beta receptor 2
hsa-miR-302d-3p	2.7E-02	-2.4	TarBase,miRecords	Experimentally Observed	ZNF226	4.1E-03	2.1	zinc finger protein 226
hsa-miR-296-5p	4.1E-02	-2.2	TargetScan Human	High (predicted)	BMF	6.4E-03	3.1	Bcl2 modifying factor
hsa-miR-296-5p	4.1E-02	-2.2	TargetScan Human	High (predicted)	C10orf105	1.5E-02	4.2	chromosome 10 open reading frame 105
hsa-miR-296-5p	4.1E-02	-2.2	TargetScan Human	High (predicted)	CCL28	3.5E-03	4.5	C-C motif chemokine ligand 28
hsa-miR-296-5p	4.1E-02	-2.2	TargetScan Human	High (predicted)	CD300LF	5.6E-03	4.9	CD300 molecule like family member f
hsa-miR-296-5p	4.1E-02	-2.2	TargetScan Human	High (predicted)	CD6	9.1E-06	40.4	CD6 molecule
hsa-miR-296-5p	4.1E-02	-2.2	TargetScan Human	High (predicted)	CD8A	1.6E-05	25.0	CD8a molecule
hsa-miR-296-5p	4.1E-02	-2.2	TargetScan Human	High (predicted)	CLDN3	4.2E-02	2.1	claudin 3
hsa-miR-296-5p	4.1E-02	-2.2	TargetScan Human	High (predicted)	CXCL10	1.7E-02	12.8	C-X-C motif chemokine ligand 10
hsa-miR-296-5p	4.1E-02	-2.2	TargetScan Human	High (predicted)	DERL3	1.9E-03	3.6	derlin 3
hsa-miR-296-5p	4.1E-02	-2.2	TargetScan Human	High (predicted)	E2F2	2.7E-02	4.3	E2F transcription factor 2

hsa-miR-296-5p	4.1E-02	-2.2	TargetScan Human	High (predicted)	ESPNL	2.9E-02	5.8	espin-like
hsa-miR-296-5p	4.1E-02	-2.2	TargetScan Human	High (predicted)	GDF10	4.1E-02	5.3	growth differentiation factor 10
hsa-miR-296-5p	4.1E-02	-2.2	TargetScan Human	High (predicted)	KCNN4	3.4E-02	3.8	potassium calcium-activated channel subfamily N member 4
hsa-miR-296-5p	4.1E-02	-2.2	TargetScan Human	High (predicted)	KRTCAP3	8.4E-03	3.8	keratinocyte associated protein 3
hsa-miR-296-5p	4.1E-02	-2.2	TargetScan Human	High (predicted)	MARVELD1	1.1E-02	2.2	MARVEL domain containing 1
hsa-miR-296-5p	4.1E-02	-2.2	TargetScan Human	High (predicted)	NFAM1	2.6E-02	4.3	NFAT activating protein with ITAM motif 1
hsa-miR-296-5p	4.1E-02	-2.2	TargetScan Human	High (predicted)	NUAK2	4.8E-02	2.1	NUAK family kinase 2
hsa-miR-296-5p	4.1E-02	-2.2	TargetScan Human	High (predicted)	RAB37	1.2E-04	4.5	RAB37, member RAS oncogene family
hsa-miR-296-5p	4.1E-02	-2.2	TargetScan Human	High (predicted)	SNX20	1.5E-03	4.3	sorting nexin 20
hsa-miR-296-5p	4.1E-02	-2.2	TargetScan Human	High (predicted)	TBC1D10C	2.0E-06	30.3	TBC1 domain family member 10C
hsa-miR-296-5p	4.1E-02	-2.2	TargetScan Human	High (predicted)	TNFSF13	2.9E-02	2.2	TNF superfamily member 13
hsa-miR-296-5p	4.1E-02	-2.2	TargetScan Human	High (predicted)	TRPM2	2.3E-03	3.8	transient receptor potential cation channel subfamily M member 2
hsa-miR-29c-3p	1.1E-02	-3.1	TargetScan Human	High (predicted)	ARRDC3	1.1E-02	3.0	arrestin domain containing 3
hsa-miR-29c-3p	1.1E-02	-3.1	TargetScan Human	High (predicted)	BMF	6.4E-03	3.1	Bcl2 modifying factor
hsa-miR-29c-3p	1.1E-02	-3.1	TargetScan Human	High (predicted)	CASP8	3.3E-04	6.2	caspase 8
hsa-miR-29c-3p	1.1E-02	-3.1	TargetScan Human	High (predicted)	CCSAP	1.4E-02	2.5	centriole, cilia and spindle associated protein
hsa-miR-29c-3p	1.1E-02	-3.1	Ingenuity Expert Findings,TarBase,TargetScan Human,miRecords	Experimentally Observed,High (predicted)	COL1A2	2.3E-02	2.9	collagen type I alpha 2 chain
hsa-miR-29c-3p	1.1E-02	-3.1	TarBase,TargetScan Human,miRecords	Experimentally Observed,High (predicted)	COL3A1	9.4E-03	3.5	collagen type III alpha 1 chain
hsa-miR-29c-3p	1.1E-02	-3.1	TargetScan Human	High (predicted)	COL4A4	3.9E-02	2.4	collagen type IV alpha 4 chain
hsa-miR-29c-3p	1.1E-02	-3.1	TargetScan Human	High (predicted)	COL5A1	2.1E-02	2.2	collagen type V alpha 1 chain
hsa-miR-29c-3p	1.1E-02	-3.1	Ingenuity Expert Findings,TargetScan Human	Experimentally Observed,High (predicted)	COL5A2	1.1E-02	2.4	collagen type V alpha 2 chain
hsa-miR-29c-3p	1.1E-02	-3.1	TargetScan Human	High (predicted)	ICOS	6.7E-03	5.0	inducible T-cell costimulator
hsa-miR-29c-3p	1.1E-02	-3.1	TargetScan Human	High (predicted)	N4BP2L1	3.7E-02	2.1	NEDD4 binding protein 2 like 1
hsa-miR-29c-3p	1.1E-02	-3.1	TargetScan Human	High (predicted)	NAP1L3	2.9E-02	8.7	nucleosome assembly protein 1 like 3
hsa-miR-29c-3p	1.1E-02	-3.1	TargetScan Human	High (predicted)	NAV1	3.1E-02	2.3	neuron navigator 1
hsa-miR-29c-3p	1.1E-02	-3.1	TargetScan Human	High (predicted)	NPAS3	1.2E-02	2.3	neuronal PAS domain protein 3

hsa-miR-29c-3p	1.1E-02	-3.1	Ingenuity Expert Findings,TargetScan Human,miRecords	Experimentally Observed	PIK3R1	4.1E-02	2.7	phosphoinositide-3-kinase regulatory subunit 1
hsa-miR-29c-3p	1.1E-02	-3.1	TargetScan Human	High (predicted)	RAB30	4.3E-03	2.0	RAB30, member RAS oncogene family
hsa-miR-29c-3p	1.1E-02	-3.1	TargetScan Human	High (predicted)	RGS1	6.3E-04	19.4	regulator of G protein signaling 1
hsa-miR-29c-3p	1.1E-02	-3.1	TargetScan Human	High (predicted)	TMEM236	1.4E-02	4.0	transmembrane protein 236
hsa-miR-29c-3p	1.1E-02	-3.1	Ingenuity Expert Findings,TargetScan Human	Experimentally Observed,High (predicted)	TNFAIP3	8.7E-03	4.5	TNF alpha induced protein 3
hsa-miR-29c-3p	1.1E-02	-3.1	TargetScan Human	High (predicted)	TNFRSF9	6.8E-03	2.3	TNF receptor superfamily member 9
hsa-miR-29c-3p	1.1E-02	-3.1	TargetScan Human	High (predicted)	ZBTB37	1.4E-02	2.4	zinc finger and BTB domain containing 37
hsa-miR-30c-5p	4.3E-02	-1.9	TarBase	Experimentally Observed	ANPEP	7.3E-03	4.5	alanyl aminopeptidase, membrane
hsa-miR-30c-5p	4.3E-02	-1.9	TargetScan Human	High (predicted)	CCL19	1.8E-02	16.1	C-C motif chemokine ligand 19
hsa-miR-30c-5p	4.3E-02	-1.9	TargetScan Human	High (predicted)	CFHR3	4.0E-02	3.0	complement factor H related 3
hsa-miR-30c-5p	4.3E-02	-1.9	TargetScan Human	High (predicted)	FAM133A	4.8E-03	4.8	family with sequence similarity 133 member A
hsa-miR-30c-5p	4.3E-02	-1.9	TargetScan Human	High (predicted)	FAM49A	5.5E-03	2.1	family with sequence similarity 49 member A
hsa-miR-30c-5p	4.3E-02	-1.9	TargetScan Human	High (predicted)	GCSAM	8.1E-03	4.0	germinal center associated signaling and motility
hsa-miR-30c-5p	4.3E-02	-1.9	TargetScan Human	High (predicted)	LRRC17	4.0E-04	5.4	leucine rich repeat containing 17
hsa-miR-30c-5p	4.3E-02	-1.9	TargetScan Human	High (predicted)	PGBD2	1.3E-02	2.1	piggyBac transposable element derived 2
hsa-miR-30c-5p	4.3E-02	-1.9	TargetScan Human	High (predicted)	PIP4K2A	2.0E-02	2.8	phosphatidylinositol-5-phosphate 4-kinase type 2 alpha
hsa-miR-30c-5p	4.3E-02	-1.9	TargetScan Human	High (predicted)	PRDM1	6.8E-05	26.8	PR/SET domain 1
hsa-miR-30c-5p	4.3E-02	-1.9	TarBase,TargetScan Human	Experimentally Observed,High (predicted)	RAB27B	4.6E-03	3.0	RAB27B, member RAS oncogene family
hsa-miR-30c-5p	4.3E-02	-1.9	TargetScan Human	High (predicted)	RAB38	2.5E-02	3.1	RAB38, member RAS oncogene family
hsa-miR-30c-5p	4.3E-02	-1.9	TargetScan Human	High (predicted)	RHEBL1	9.1E-03	2.4	Ras homolog enriched in brain like 1
hsa-miR-30c-5p	4.3E-02	-1.9	Ingenuity Expert Findings,TargetScan Human	Experimentally Observed,High (predicted)	RUNX2	4.4E-04	6.0	runt related transcription factor 2
hsa-miR-30c-5p	4.3E-02	-1.9	TargetScan Human	High (predicted)	SCN9A	2.2E-02	2.7	sodium voltage-gated channel alpha subunit 9
hsa-miR-30c-5p	4.3E-02	-1.9	TargetScan Human	High (predicted)	SOCS1	2.0E-03	3.9	suppressor of cytokine signaling 1

hsa-miR-30c-5p	4.3E-02	-1.9	TargetScan Human	High (predicted)	STIM2	3.6E-02	2.0	stromal interaction molecule 2
hsa-miR-30c-5p	4.3E-02	-1.9	TargetScan Human	High (predicted)	TNFSF13B	5.5E-03	5.4	TNF superfamily member 13b
hsa-miR-324-5p	2.0E-02	-2.9	TargetScan Human	High (predicted)	C10orf105	1.5E-02	4.2	chromosome 10 open reading frame 105
hsa-miR-324-5p	2.0E-02	-2.9	TargetScan Human	High (predicted)	CD84	1.1E-04	11.1	CD84 molecule
hsa-miR-324-5p	2.0E-02	-2.9	TargetScan Human	High (predicted)	CRTAM	3.2E-05	27.8	cytotoxic and regulatory T-cell molecule
hsa-miR-324-5p	2.0E-02	-2.9	TargetScan Human	High (predicted)	CTLA4	3.8E-03	10.7	cytotoxic T-lymphocyte associated protein 4
hsa-miR-324-5p	2.0E-02	-2.9	TargetScan Human	High (predicted)	FAM19A2	2.9E-02	2.2	family with sequence similarity 19 member A2, C-C motif chemokine like
hsa-miR-324-5p	2.0E-02	-2.9	TargetScan Human	High (predicted)	KIAA0513	1.3E-02	2.0	KIAA0513
hsa-miR-324-5p	2.0E-02	-2.9	TargetScan Human	High (predicted)	P2RY13	3.4E-02	3.6	purinergic receptor P2Y13
hsa-miR-324-5p	2.0E-02	-2.9	TargetScan Human	High (predicted)	RAB27B	4.6E-03	3.0	RAB27B, member RAS oncogene family
hsa-miR-324-5p	2.0E-02	-2.9	TargetScan Human	High (predicted)	REEP4	1.1E-02	2.2	receptor accessory protein 4
hsa-miR-324-5p	2.0E-02	-2.9	TargetScan Human	High (predicted)	S1PR4	1.8E-04	7.1	sphingosine-1-phosphate receptor 4
hsa-miR-324-5p	2.0E-02	-2.9	TargetScan Human	High (predicted)	THEMIS2	1.5E-02	4.0	thymocyte selection associated family member 2
hsa-miR-328-3p	3.0E-02	-2.1	TargetScan Human	High (predicted)	APBB1IP	5.8E-04	6.2	amyloid beta precursor protein binding family B member 1 interacting protein
hsa-miR-328-3p	3.0E-02	-2.1	TargetScan Human	High (predicted)	C16orf89	1.1E-02	5.4	chromosome 16 open reading frame 89
hsa-miR-328-3p	3.0E-02	-2.1	TargetScan Human	High (predicted)	FRAT1	4.4E-02	2.2	FRAT1, WNT signaling pathway regulator
hsa-miR-328-3p	3.0E-02	-2.1	TargetScan Human	High (predicted)	HKDC1	5.3E-03	3.3	hexokinase domain containing 1
hsa-miR-328-3p	3.0E-02	-2.1	TargetScan Human	High (predicted)	KY	4.6E-02	3.3	kyphoscoliosis peptidase
hsa-miR-328-3p	3.0E-02	-2.1	TargetScan Human	High (predicted)	LOC100996763/NOTCH2NL	1.8E-02	2.7	notch 2 N-terminal like
hsa-miR-328-3p	3.0E-02	-2.1	TargetScan Human	High (predicted)	PRF1	1.5E-04	6.1	perforin 1
hsa-miR-328-3p	3.0E-02	-2.1	TargetScan Human	High (predicted)	PSTPIP2	8.7E-03	4.5	proline-serine-threonine phosphatase interacting protein 2
hsa-miR-328-3p	3.0E-02	-2.1	TargetScan Human	High (predicted)	RASL11B	9.4E-03	6.0	RAS like family 11 member B
hsa-miR-33a-3p	1.9E-02	-2.2	TargetScan Human	High (predicted)	ENKUR	4.9E-02	2.6	enkurin, TRPC channel interacting protein
hsa-miR-335-5p	2.1E-02	-2.2	TargetScan Human	High (predicted)	ADAMDEC1	4.9E-02	2.7	ADAM like decysin 1
hsa-miR-335-5p	2.1E-02	-2.2	TargetScan Human	High (predicted)	ALOX5AP	3.0E-02	4.6	arachidonate 5-lipoxygenase activating protein
hsa-miR-335-5p	2.1E-02	-2.2	TargetScan Human	High (predicted)	NBPF10 (includes others)	9.4E-04	2.2	NBPF member 20
hsa-miR-335-5p	2.1E-02	-2.2	TargetScan Human	High (predicted)	PTPN22	7.4E-05	16.7	protein tyrosine phosphatase, non-receptor type 22

hsa-miR-335-5p	2.1E-02	-2.2	TargetScan Human	High (predicted)	UCMA	2.0E-02	4.2	upper zone of growth plate and cartilage matrix associated
hsa-miR-345-5p	1.4E-02	-2.1	TargetScan Human	High (predicted)	MAP3K7CL	3.5E-02	4.8	MAP3K7 C-terminal like
hsa-miR-345-5p	1.4E-02	-2.1	TargetScan Human	High (predicted)	RGS1	6.3E-04	19.4	regulator of G protein signaling 1
hsa-miR-345-5p	1.4E-02	-2.1	TargetScan Human	High (predicted)	SAPCD1	1.5E-02	2.6	suppressor APC domain containing 1
hsa-miR-345-5p	1.4E-02	-2.1	TargetScan Human	High (predicted)	TGM1	1.8E-02	2.8	transglutaminase 1
hsa-miR-345-5p	1.4E-02	-2.1	TargetScan Human	High (predicted)	UBE2L6	4.9E-02	2.1	ubiquitin conjugating enzyme E2 L6
hsa-miR-361-5p	3.1E-02	-2.9	TargetScan Human	High (predicted)	ERI2	3.6E-02	2.1	ERI1 exoribonuclease family member 2
hsa-miR-361-5p	3.1E-02	-2.9	TargetScan Human	High (predicted)	OGN	1.2E-02	7.0	osteoglycin
hsa-miR-361-5p	3.1E-02	-2.9	TargetScan Human	High (predicted)	RAB27B	4.6E-03	3.0	RAB27B, member RAS oncogene family
hsa-miR-361-5p	3.1E-02	-2.9	TargetScan Human	High (predicted)	RAB30	4.3E-03	2.0	RAB30, member RAS oncogene family
hsa-miR-361-5p	3.1E-02	-2.9	TargetScan Human	High (predicted)	RMI2	2.3E-02	3.6	RecQ mediated genome instability 2
hsa-miR-365a-3p	2.1E-03	-2.7	TargetScan Human	High (predicted)	ARRDC3	1.1E-02	3.0	arrestin domain containing 3
hsa-miR-365a-3p	2.1E-03	-2.7	TargetScan Human	High (predicted)	DOK2	1.4E-02	5.4	docking protein 2
hsa-miR-365a-3p	2.1E-03	-2.7	TargetScan Human	High (predicted)	E2F2	2.7E-02	4.3	E2F transcription factor 2
hsa-miR-365a-3p	2.1E-03	-2.7	TargetScan Human	High (predicted)	HELLS	2.3E-02	3.3	helicase, lymphoid-specific
hsa-miR-365a-3p	2.1E-03	-2.7	TargetScan Human	High (predicted)	ITGAD	3.9E-03	13.9	integrin subunit alpha D
hsa-miR-365a-3p	2.1E-03	-2.7	TargetScan Human	High (predicted)	MCOLN2	7.1E-06	24.1	mucolipin 2
hsa-miR-365a-3p	2.1E-03	-2.7	TargetScan Human	High (predicted)	NABP1	2.5E-02	3.6	nucleic acid binding protein 1
hsa-miR-365a-3p	2.1E-03	-2.7	TargetScan Human	High (predicted)	RHEBL1	9.1E-03	2.4	Ras homolog enriched in brain like 1
hsa-miR-376a-3p	2.6E-03	-2.6	TargetScan Human	High (predicted)	CASP8	3.3E-04	6.2	caspase 8
hsa-miR-376a-3p	2.6E-03	-2.6	TargetScan Human	High (predicted)	CATSPER2	4.0E-02	2.1	cation channel sperm associated 2
hsa-miR-376a-3p	2.6E-03	-2.6	TargetScan Human	High (predicted)	GIMAP2	3.2E-02	2.8	GTPase, IMAP family member 2
hsa-miR-376a-3p	2.6E-03	-2.6	TargetScan Human	High (predicted)	IL7	2.2E-03	6.2	interleukin 7
hsa-miR-376a-3p	2.6E-03	-2.6	TargetScan Human	High (predicted)	LILRA2	3.9E-02	3.1	leukocyte immunoglobulin like receptor A2
hsa-miR-376a-3p	2.6E-03	-2.6	TargetScan Human	High (predicted)	RGS1	6.3E-04	19.4	regulator of G protein signaling 1
hsa-miR-376c-3p	2.0E-02	-2.0	TargetScan Human	High (predicted)	PARP4	2.1E-02	2.0	poly(ADP-ribose) polymerase family member 4
hsa-miR-376c-3p	2.0E-02	-2.0	TargetScan Human	High (predicted)	THEMIS	6.9E-06	32.9	thymocyte selection associated
hsa-miR-376c-3p	2.0E-02	-2.0	TargetScan Human	High (predicted)	TMEM154	3.1E-02	2.2	transmembrane protein 154
hsa-miR-422a	9.6E-03	-2.6	TargetScan Human	High (predicted)	ARHGAP15	6.5E-03	2.6	Rho GTPase activating protein 15
hsa-miR-422a	9.6E-03	-2.6	TargetScan Human	High (predicted)	CCR7	1.8E-03	14.7	C-C motif chemokine receptor 7
hsa-miR-422a	9.6E-03	-2.6	TargetScan Human	High (predicted)	CD226	1.5E-03	6.5	CD226 molecule
hsa-miR-422a	9.6E-03	-2.6	TargetScan Human	High (predicted)	DHX58	1.9E-02	2.2	DExH-box helicase 58
hsa-miR-422a	9.6E-03	-2.6	TargetScan Human	High (predicted)	NUAK2	4.8E-02	2.1	NUAK family kinase 2
hsa-miR-422a	9.6E-03	-2.6	TargetScan Human	High (predicted)	PIK3CG	1.1E-03	5.4	phosphatidylinositol-4,5-bisphosphate 3-kinase catalytic subunit gamma

hsa-miR-378a-5p	2.8E-03	-3.1	TargetScan Human	High (predicted)	CCNG2	3.8E-02	2.9	cyclin G2
hsa-miR-378a-5p	2.8E-03	-3.1	TargetScan Human	High (predicted)	CD33	2.4E-02	3.8	CD33 molecule
hsa-miR-378a-5p	2.8E-03	-3.1	TargetScan Human	High (predicted)	FRZB	1.6E-03	5.9	frizzled-related protein
hsa-miR-378a-5p	2.8E-03	-3.1	TargetScan Human	High (predicted)	LDLRAD2	7.0E-03	6.6	low density lipoprotein receptor class A domain containing 2
hsa-miR-379-5p	1.3E-02	-2.4	TargetScan Human	High (predicted)	CXCL11	2.1E-02	14.7	C-X-C motif chemokine ligand 11
hsa-miR-379-5p	1.3E-02	-2.4	TargetScan Human	High (predicted)	HLA-E	1.6E-02	2.1	major histocompatibility complex, class I, E
hsa-miR-379-5p	1.3E-02	-2.4	TargetScan Human	High (predicted)	ICOS	6.7E-03	5.0	inducible T-cell costimulator
hsa-miR-379-5p	1.3E-02	-2.4	TargetScan Human	High (predicted)	IL18RAP	1.3E-02	6.0	interleukin 18 receptor accessory protein
hsa-miR-379-5p	1.3E-02	-2.4	TargetScan Human	High (predicted)	LINC01588	8.1E-04	7.0	long intergenic non-protein coding RNA 1588
hsa-miR-379-5p	1.3E-02	-2.4	TargetScan Human	High (predicted)	OGN	1.2E-02	7.0	osteoglycin
hsa-miR-379-5p	1.3E-02	-2.4	TargetScan Human	High (predicted)	RAB30	4.3E-03	2.0	RAB30, member RAS oncogene family
hsa-miR-379-5p	1.3E-02	-2.4	TargetScan Human	High (predicted)	SIT1	1.6E-04	13.2	signaling threshold regulating transmembrane adaptor 1
hsa-miR-379-5p	1.3E-02	-2.4	TargetScan Human	High (predicted)	SPEF2	3.4E-03	5.0	sperm flagellar 2
hsa-miR-409-3p	4.2E-02	-1.8	TargetScan Human	High (predicted)	CCL28	3.5E-03	4.5	C-C motif chemokine ligand 28
hsa-miR-409-3p	4.2E-02	-1.8	TargetScan Human	High (predicted)	TNFSF14	6.0E-03	8.9	TNF superfamily member 14
hsa-miR-411-5p	1.5E-02	-2.2	TargetScan Human	High (predicted)	METTL7A	2.3E-02	2.9	methyltransferase like 7A
hsa-miR-423-5p	2.2E-02	-2.2	TargetScan Human	High (predicted)	ANPEP	7.3E-03	4.5	alanyl aminopeptidase, membrane
hsa-miR-423-5p	2.2E-02	-2.2	TargetScan Human	High (predicted)	ARHGAP4	8.1E-03	2.7	Rho GTPase activating protein 4
hsa-miR-423-5p	2.2E-02	-2.2	TargetScan Human	High (predicted)	BMF	6.4E-03	3.1	Bcl2 modifying factor
hsa-miR-423-5p	2.2E-02	-2.2	TargetScan Human	High (predicted)	C10orf105	1.5E-02	4.2	chromosome 10 open reading frame 105
hsa-miR-423-5p	2.2E-02	-2.2	TargetScan Human	High (predicted)	CD72	7.2E-03	7.3	CD72 molecule
hsa-miR-423-5p	2.2E-02	-2.2	TargetScan Human	High (predicted)	CD8A	1.6E-05	25.0	CD8a molecule
hsa-miR-423-5p	2.2E-02	-2.2	TargetScan Human	High (predicted)	CXCL16	3.6E-02	3.0	C-X-C motif chemokine ligand 16
hsa-miR-423-5p	2.2E-02	-2.2	TargetScan Human	High (predicted)	KLHDC7B	5.5E-03	8.2	kelch domain containing 7B
hsa-miR-423-5p	2.2E-02	-2.2	TargetScan Human	High (predicted)	METTL27	4.6E-02	5.7	methyltransferase like 27
hsa-miR-423-5p	2.2E-02	-2.2	TargetScan Human	High (predicted)	MYCL	1.1E-02	6.2	MYCL proto-oncogene, bHLH transcription factor
hsa-miR-423-5p	2.2E-02	-2.2	TargetScan Human	High (predicted)	NFAM1	2.6E-02	4.3	NFAT activating protein with ITAM motif 1
hsa-miR-423-5p	2.2E-02	-2.2	TargetScan Human	High (predicted)	NKG7	6.1E-06	12.4	natural killer cell granule protein 7
hsa-miR-423-5p	2.2E-02	-2.2	TargetScan Human	High (predicted)	PTK2B	3.1E-03	3.4	protein tyrosine kinase 2 beta
hsa-miR-423-5p	2.2E-02	-2.2	TargetScan Human	High (predicted)	RAC2	1.0E-03	4.1	ras-related C3 botulinum toxin substrate 2 (rho family, small GTP binding protein Rac2)
hsa-miR-423-5p	2.2E-02	-2.2	TargetScan Human	High (predicted)	RGS1	6.3E-04	19.4	regulator of G protein signaling 1

hsa-miR-423-5p	2.2E-02	-2.2	TargetScan Human	High (predicted)	RIMBP3 (includes others)	1.3E-03	5.7	RIMS binding protein 3
hsa-miR-423-5p	2.2E-02	-2.2	TargetScan Human	High (predicted)	SELL	6.5E-03	8.1	selectin L
hsa-miR-423-5p	2.2E-02	-2.2	TargetScan Human	High (predicted)	SRCIN1	3.4E-03	5.0	SRC kinase signaling inhibitor 1
hsa-miR-423-5p	2.2E-02	-2.2	TargetScan Human	High (predicted)	THBS3	4.8E-02	2.2	thrombospondin 3
hsa-miR-423-5p	2.2E-02	-2.2	TargetScan Human	High (predicted)	THY1	2.8E-02	6.8	Thy-1 cell surface antigen
hsa-miR-423-5p	2.2E-02	-2.2	TargetScan Human	High (predicted)	TIFAB	1.2E-03	16.9	TIFA inhibitor
hsa-miR-423-5p	2.2E-02	-2.2	TargetScan Human	High (predicted)	TMED8	1.5E-02	2.3	transmembrane p24 trafficking protein family member 8
hsa-miR-423-5p	2.2E-02	-2.2	TargetScan Human	High (predicted)	TRABD2A	2.6E-03	2.8	TraB domain containing 2A
hsa-miR-423-5p	2.2E-02	-2.2	TargetScan Human	High (predicted)	TRIM11	5.4E-03	2.3	tripartite motif containing 11
hsa-miR-451a	1.7E-02	-3.7	TargetScan Human	High (predicted)	BATF	1.0E-03	13.3	basic leucine zipper ATF-like transcription factor
hsa-miR-451a	1.7E-02	-3.7	TargetScan Human	High (predicted)	CXCL16	3.6E-02	3.0	C-X-C motif chemokine ligand 16
hsa-miR-451a	1.7E-02	-3.7	TargetScan Human	High (predicted)	CXorf21	4.5E-03	5.0	chromosome X open reading frame 21
hsa-miR-451a	1.7E-02	-3.7	TargetScan Human	High (predicted)	HELLS	2.3E-02	3.3	helicase, lymphoid-specific
hsa-miR-455-3p	1.5E-02	-3.3	TargetScan Human	High (predicted)	FAM105A	2.1E-03	5.5	family with sequence similarity 105 member A
hsa-miR-455-3p	1.5E-02	-3.3	TargetScan Human	High (predicted)	PITX1	4.7E-02	3.5	paired like homeodomain 1
hsa-miR-455-3p	1.5E-02	-3.3	TargetScan Human	High (predicted)	RGS10	1.8E-02	3.4	regulator of G protein signaling 10
hsa-miR-455-3p	1.5E-02	-3.3	TargetScan Human	High (predicted)	STK17B	3.0E-02	3.0	serine/threonine kinase 17b
hsa-miR-455-3p	1.5E-02	-3.3	TargetScan Human	High (predicted)	UBE2L6	4.9E-02	2.1	ubiquitin conjugating enzyme E2 L6
hsa-miR-455-3p	1.5E-02	-3.3	TargetScan Human	High (predicted)	XCL1	4.4E-04	20.0	X-C motif chemokine ligand 1
hsa-miR-455-5p	1.2E-02	-3.0	TargetScan Human	High (predicted)	CCSAP	1.4E-02	2.5	centriole, cilia and spindle associated protein
hsa-miR-455-5p	1.2E-02	-3.0	TargetScan Human	High (predicted)	CD200R1	2.0E-02	2.6	CD200 receptor 1
hsa-miR-455-5p	1.2E-02	-3.0	TargetScan Human	High (predicted)	FCAR	1.6E-02	3.0	Fc fragment of IgA receptor
hsa-miR-455-5p	1.2E-02	-3.0	TargetScan Human	High (predicted)	MS4A7	3.2E-02	2.8	membrane spanning 4-domains A7
hsa-miR-455-5p	1.2E-02	-3.0	TargetScan Human	High (predicted)	NCK2	3.5E-03	3.0	NCK adaptor protein 2
hsa-miR-455-5p	1.2E-02	-3.0	TargetScan Human	High (predicted)	TSPAN5	4.6E-03	3.5	tetraspanin 5
hsa-miR-486-5p	4.6E-02	-2.0	TargetScan Human	High (predicted)	CD247	3.2E-05	17.9	CD247 molecule
hsa-miR-486-5p	4.6E-02	-2.0	TargetScan Human	High (predicted)	LRRC25	3.8E-02	3.3	leucine rich repeat containing 25
hsa-miR-486-5p	4.6E-02	-2.0	TargetScan Human	High (predicted)	PTGDR	3.0E-03	5.2	prostaglandin D2 receptor
hsa-miR-494-3p	8.2E-03	-3.5	TargetScan Human	High (predicted)	ADAM28	5.4E-03	4.1	ADAM metallopeptidase domain 28
hsa-miR-494-3p	8.2E-03	-3.5	TargetScan Human	High (predicted)	ANXA2R	1.6E-02	2.2	annexin A2 receptor
hsa-miR-494-3p	8.2E-03	-3.5	TargetScan Human	High (predicted)	KLRC4	3.3E-06	17.8	killer cell lectin like receptor C4

hsa-miR-494-3p	8.2E-03	-3.5	TargetScan Human	High (predicted)	MNDA	9.7E-03	5.0	myeloid cell nuclear differentiation antigen
hsa-miR-494-3p	8.2E-03	-3.5	TargetScan Human	High (predicted)	SLAMF1	2.9E-02	4.9	signaling lymphocytic activation molecule family member 1
hsa-miR-494-3p	8.2E-03	-3.5	TargetScan Human	High (predicted)	UBE2L6	4.9E-02	2.1	ubiquitin conjugating enzyme E2 L6
hsa-miR-494-3p	8.2E-03	-3.5	TargetScan Human	High (predicted)	ZBTB37	1.4E-02	2.4	zinc finger and BTB domain containing 37
hsa-miR-499a-5p	6.2E-03	-3.8	TargetScan Human	High (predicted)	CXCL11	2.1E-02	14.7	C-X-C motif chemokine ligand 11
hsa-miR-499a-5p	6.2E-03	-3.8	TargetScan Human	High (predicted)	ENKUR	4.9E-02	2.6	enkurin, TRPC channel interacting protein
hsa-miR-499a-5p	6.2E-03	-3.8	TargetScan Human	High (predicted)	SCRG1	4.5E-02	3.3	stimulator of chondrogenesis 1
hsa-miR-499a-5p	6.2E-03	-3.8	TargetScan Human	High (predicted)	VAV3	6.1E-03	2.6	vav guanine nucleotide exchange factor 3
hsa-miR-532-5p	3.6E-02	-1.9	TargetScan Human	High (predicted)	MS4A14	4.7E-02	2.9	membrane spanning 4-domains A14
hsa-miR-532-5p	3.6E-02	-1.9	TargetScan Human	High (predicted)	NCF2	2.2E-02	3.6	neutrophil cytosolic factor 2
hsa-miR-532-5p	3.6E-02	-1.9	TargetScan Human,miRecords	Experimentally Observed	RUNX3	1.5E-05	16.8	runt related transcription factor 3
hsa-miR-532-5p	3.6E-02	-1.9	TargetScan Human	High (predicted)	TFEC	4.1E-02	4.4	transcription factor EC
hsa-miR-532-5p	3.6E-02	-1.9	TargetScan Human	High (predicted)	TTC14	3.3E-04	2.2	tetratricopeptide repeat domain 14
hsa-miR-532-5p	3.6E-02	-1.9	TargetScan Human	High (predicted)	UTS2B	3.0E-02	2.5	urotensin 2B
hsa-miR-532-5p	3.6E-02	-1.9	TargetScan Human	High (predicted)	ZBTB37	1.4E-02	2.4	zinc finger and BTB domain containing 37
hsa-miR-642a-5p	7.8E-03	-2.5	TargetScan Human	High (predicted)	ARHGAP25	4.8E-03	2.8	Rho GTPase activating protein 25
hsa-miR-642a-5p	7.8E-03	-2.5	TargetScan Human	High (predicted)	FIBIN	6.4E-03	4.8	fin bud initiation factor homolog (zebrafish)
hsa-miR-642a-5p	7.8E-03	-2.5	TargetScan Human	High (predicted)	GCSAM	8.1E-03	4.0	germinal center associated signaling and motility
hsa-miR-642a-5p	7.8E-03	-2.5	TargetScan Human	High (predicted)	LTB	1.4E-02	8.6	lymphotoxin beta
hsa-miR-28-5p	2.9E-02	-2.2	TargetScan Human	High (predicted)	CCL28	3.5E-03	4.5	C-C motif chemokine ligand 28
hsa-miR-28-5p	2.9E-02	-2.2	TargetScan Human	High (predicted)	EOMES	3.8E-06	27.5	eomesodermin
hsa-miR-28-5p	2.9E-02	-2.2	TargetScan Human	High (predicted)	N4BP2L1	3.7E-02	2.1	NEDD4 binding protein 2 like 1
hsa-miR-28-5p	2.9E-02	-2.2	TargetScan Human	High (predicted)	SCRG1	4.5E-02	3.3	stimulator of chondrogenesis 1
hsa-miR-28-5p	2.9E-02	-2.2	TargetScan Human	High (predicted)	SNX20	1.5E-03	4.3	sorting nexin 20
hsa-miR-744-5p	3.1E-02	-2.1	TargetScan Human	High (predicted)	B3GAT1	8.3E-03	6.5	beta-1,3-glucuronyltransferase 1
hsa-miR-744-5p	3.1E-02	-2.1	TargetScan Human	High (predicted)	BCL11B	1.5E-04	13.7	B-cell CLL/lymphoma 11B
hsa-miR-744-5p	3.1E-02	-2.1	TargetScan Human	High (predicted)	BLK	8.1E-04	12.3	BLK proto-oncogene, Src family tyrosine kinase
hsa-miR-744-5p	3.1E-02	-2.1	TargetScan Human	High (predicted)	CD37	3.1E-04	9.6	CD37 molecule
hsa-miR-744-5p	3.1E-02	-2.1	TargetScan Human	High (predicted)	CORO7/CORO7-PAM16	3.3E-02	2.0	coronin 7
hsa-miR-744-5p	3.1E-02	-2.1	TargetScan Human	High (predicted)	GFRA2	2.3E-03	3.4	GDNF family receptor alpha 2
hsa-miR-744-5p	3.1E-02	-2.1	TargetScan Human	High (predicted)	HAAO	1.1E-02	3.9	3-hydroxyanthranilate 3,4-dioxygenase
hsa-miR-744-5p	3.1E-02	-2.1	TargetScan Human	High (predicted)	HDAC10	5.9E-03	2.9	histone deacetylase 10

hsa-miR-744-5p	3.1E-02	-2.1	TargetScan Human	High (predicted)	KIF21B	1.2E-02	2.7	kinesin family member 21B
hsa-miR-744-5p	3.1E-02	-2.1	TargetScan Human	High (predicted)	MMP25	1.6E-03	6.1	matrix metallopeptidase 25
hsa-miR-744-5p	3.1E-02	-2.1	TargetScan Human	High (predicted)	MYO1G	5.5E-03	7.7	myosin IG
hsa-miR-744-5p	3.1E-02	-2.1	TargetScan Human	High (predicted)	OSBPL7	3.5E-03	2.3	oxysterol binding protein like 7
hsa-miR-744-5p	3.1E-02	-2.1	TargetScan Human	High (predicted)	PCNX2	5.7E-03	4.8	pecanex homolog 2 (Drosophila)
hsa-miR-744-5p	3.1E-02	-2.1	TargetScan Human	High (predicted)	PLD4	3.9E-03	2.9	phospholipase D family member 4
hsa-miR-744-5p	3.1E-02	-2.1	TargetScan Human	High (predicted)	SFMBT2	3.1E-02	2.0	Scm-like with four mbt domains 2
hsa-miR-744-5p	3.1E-02	-2.1	TargetScan Human	High (predicted)	SRCIN1	3.4E-03	5.0	SRC kinase signaling inhibitor 1
hsa-miR-744-5p	3.1E-02	-2.1	TargetScan Human	High (predicted)	TCF7	1.7E-02	3.0	transcription factor 7 (T-cell specific, HMG-box)
hsa-miR-744-5p	3.1E-02	-2.1	TargetScan Human	High (predicted)	THY1	2.8E-02	6.8	Thy-1 cell surface antigen
hsa-miR-9-3p	3.9E-03	-2.5	TargetScan Human	High (predicted)	IL4	2.9E-02	2.3	interleukin 4
hsa-miR-9-3p	3.9E-03	-2.5	TargetScan Human	High (predicted)	MDK	4.1E-02	2.4	midkine (neurite growth-promoting factor 2)
hsa-miR-9-3p	3.9E-03	-2.5	TargetScan Human	High (predicted)	RGS10	1.8E-02	3.4	regulator of G protein signaling 10
hsa-miR-9-3p	3.9E-03	-2.5	TargetScan Human	High (predicted)	USP51	1.9E-02	3.0	ubiquitin specific peptidase 51
hsa-miR-9-3p	3.9E-03	-2.5	TargetScan Human	High (predicted)	ZNF214	1.2E-02	3.1	zinc finger protein 214
hsa-miR-9-5p	3.5E-03	-2.9	TargetScan Human	High (predicted)	CLDN14	5.3E-03	4.0	claudin 14
hsa-miR-9-5p	3.5E-03	-2.9	TargetScan Human	High (predicted)	CXCL11	2.1E-02	14.7	C-X-C motif chemokine ligand 11
hsa-miR-9-5p	3.5E-03	-2.9	TargetScan Human	High (predicted)	EMB	4.6E-04	6.6	embigin
hsa-miR-9-5p	3.5E-03	-2.9	TargetScan Human	High (predicted)	FOXN2	6.3E-03	2.4	forkhead box N2
hsa-miR-9-5p	3.5E-03	-2.9	TargetScan Human	High (predicted)	FOXP2	1.4E-02	3.4	forkhead box P2
hsa-miR-9-5p	3.5E-03	-2.9	TargetScan Human	High (predicted)	GCSAM	8.1E-03	4.0	germinal center associated signaling and motility
hsa-miR-9-5p	3.5E-03	-2.9	Ingenuity Expert Findings,TargetScan Human,miRecords	Experimentally Observed,High (predicted)	PRDM1	6.8E-05	26.8	PR/SET domain 1
hsa-miR-9-5p	3.5E-03	-2.9	TargetScan Human	High (predicted)	RANBP17	2.4E-02	3.4	RAN binding protein 17
hsa-miR-9-5p	3.5E-03	-2.9	TargetScan Human	High (predicted)	VAV3	6.1E-03	2.6	vav guanine nucleotide exchange factor 3
hsa-miR-25-3p	4.5E-02	-2.0	TargetScan Human	High (predicted)	ARRDC3	1.1E-02	3.0	arrestin domain containing 3
hsa-miR-25-3p	4.5E-02	-2.0	TargetScan Human	High (predicted)	ASPN	5.6E-03	5.0	asporin
hsa-miR-25-3p	4.5E-02	-2.0	Ingenuity Expert Findings,TargetScan Human,miRecords	Experimentally Observed	BCL2L11	3.3E-02	2.4	BCL2 like 11
hsa-miR-25-3p	4.5E-02	-2.0	TargetScan Human	High (predicted)	BTLA	2.4E-03	8.6	B and T lymphocyte associated
hsa-miR-25-3p	4.5E-02	-2.0	TargetScan Human	High (predicted)	CD69	1.3E-05	20.0	CD69 molecule
hsa-miR-25-3p	4.5E-02	-2.0	TargetScan Human	High (predicted)	FOXN2	6.3E-03	2.4	forkhead box N2

hsa-miR-25-3p	4.5E-02	-2.0	TargetScan Human	High (predicted)	IFIT2	8.5E-03	3.2	interferon induced protein with tetratricopeptide repeats 2
hsa-miR-25-3p	4.5E-02	-2.0	Ingenuity Expert Findings	Experimentally Observed	IKZF1	1.5E-05	17.4	IKAROS family zinc finger 1
hsa-miR-25-3p	4.5E-02	-2.0	TargetScan Human	High (predicted)	KLRF1	1.6E-02	2.1	killer cell lectin like receptor F1
hsa-miR-25-3p	4.5E-02	-2.0	TargetScan Human	High (predicted)	MCOLN2	7.1E-06	24.1	mucolipin 2
hsa-miR-25-3p	4.5E-02	-2.0	TargetScan Human	High (predicted)	SCRG1	4.5E-02	3.3	stimulator of chondrogenesis 1
hsa-miR-25-3p	4.5E-02	-2.0	TargetScan Human	High (predicted)	TAGAP	2.7E-04	17.9	T-cell activation RhoGTPase activating protein
hsa-miR-25-3p	4.5E-02	-2.0	TargetScan Human	High (predicted)	ZNF287	3.5E-02	3.3	zinc finger protein 287
hsa-miR-95-3p	4.0E-02	-2.1	TargetScan Human	High (predicted)	KMO	2.3E-02	3.0	kynurenine 3-monooxygenase
hsa-miR-95-3p	4.0E-02	-2.1	TargetScan Human	High (predicted)	PLVAP	4.0E-02	3.6	plasmalemma vesicle associated protein
