# Supplementary material for: miRNAs may play a major role in the control of gene expression in key pathobiological processes in Chagas disease cardiomyopathy
Source: PLoS Negl Trop Dis. 2020 Dec 22;14(12):e0008889. doi: 10.1371/journal.pntd.0008889 (PMC7787679; doi:10.1371/journal.pntd.0008889)
Supplement: S10 Table — (PDF) [file pntd.0008889.s010.pdf]

S10 table. DEM-DEG interaction in each pathobiological function or process in CCC myocardium.

DEM-DEG interaction classification

Mitochondrial genes

DEM ID	Expr p-value	Expr Fold Change	Confidence	DEG ID	Expr p-value	Expr Fold Change
hsa-miR-101-3p	4.17E-02	-2.891	High (predicted)	DDIT4	6.61E-03	4.020
hsa-miR-103a-3p	2.61E-02	-2.121	High (predicted)	SH3BP5	3.81E-02	2.158
hsa-miR-125b-5p	4.58E-02	-2.252	Experimentally Observed	CDKN2A	4.77E-03	4.656
hsa-miR-127-3p	4.01E-02	-1.909	High (predicted)	XRCC3	1.55E-02	2.016
hsa-miR-106b-5p	3.04E-02	-2.348	Experimentally Observed	BCL2L11	3.29E-02	2.359
hsa-miR-193a-5p	4.68E-02	-1.699	High (predicted)	BCL2L11	3.29E-02	2.359
hsa-miR-22-3p	7.27E-03	-4.686	High (predicted)	DDIT4	6.61E-03	4.020
hsa-miR-221-3p	6.50E-03	-4.286	Experimentally Observed	BCL2L11	3.29E-02	2.359
hsa-miR-221-3p	6.50E-03	-4.286	Experimentally Observed	DDIT4	6.61E-03	4.020
hsa-miR-24-2-5p	2.27E-02	-2.073	High (predicted)	PIF1	8.20E-03	2.975
hsa-miR-24-3p	3.88E-02	-1.832	High (predicted)	BCL2L11	3.29E-02	2.359
hsa-miR-24-3p	3.88E-02	-1.832	Experimentally Observed	CDKN2A	4.77E-03	4.656
hsa-miR-29c-3p	1.12E-02	-3.142	High (predicted)	CASP8	3.27E-04	6.203
hsa-miR-376a-3p	2.56E-03	-2.627	High (predicted)	CASP8	3.27E-04	6.203
hsa-miR-25-3p	4.50E-02	-2.008	Experimentally Observed	BCL2L11	3.29E-02	2.359

Arrythmia

DEM ID	Expr p-value	Expr Fold Change	Confidence	DEG ID	Expr p-value	Expr Fold Change
hsa-miR-145-5p	6	2.04E-03	High (predicted)	KCNA4	1.59E-02	3.751
hsa-miR-19b-1-5p	7	1.60E-02	High (predicted)	P2RY12	1.88E-03	5.073
hsa-miR-212-3p	3	1.92E-02	Experimentally Observed	MMP9	3.63E-02	7.262
hsa-miR-27b-3p	11	6.08E-03	High (predicted)	KCNA4	1.59E-02	3.751
hsa-miR-30c-5p	18	4.34E-02	High (predicted)	SCN9A	2.16E-02	2.735
hsa-miR-422a	6	9.63E-03	High (predicted)	PIK3CG	1.09E-03	5.372

Nrf2 induced genes and oxidative stress

DEM ID	Expr p-value	Expr Fold Change	Confidence	DEG ID	Expr p-value	Expr Fold Change
hsa-miR-99a-5p	1.43E-02	-2.885	High (predicted)	VNN1	3.60E-02	4.331
hsa-miR-212-3p	1.92E-02	-2.587	Experimentally Observed	MMP9	3.63E-02	7.262
hsa-miR-155-5p	6.53E-05	3.781	Experimentally Observed	MET	3.40E-03	-2.488
hsa-miR-193a-5p	4.68E-02	-1.699	High (predicted)	C1orf186	5.38E-03	5.864
hsa-miR-221-3p	6.50E-03	-4.286	High (predicted)	HNMT	1.89E-03	2.539
hsa-miR-221-3p	6.50E-03	-4.286	Experimentally Observed.High (predicted)	PIK3R1	4.08E-02	2.670
hsa-miR-302d-3p	2.65E-02	-2.399	Experimentally Observed	STK4	9.12E-04	2.480
hsa-miR-296-5p	4.12E-02	-2.217	High (predicted)	TRPM2	2.25E-03	3.771
hsa-miR-29c-3p	1.12E-02	-3.142	Experimentally Observed	PIK3R1	4.08E-02	2.670
hsa-miR-532-5p	3.58E-02	-1.943	High (predicted)	NCF2	2.16E-02	3.550

Fibrosis

DEM ID	Expr p-value	Expr Fold Change	Confidence	DEG ID	Expr p-value	Expr Fold Change
hsa-miR-212-3p	1.92E-02	-2.587	Experimentally Observed	MMP9	3.63E-02	7.262
hsa-miR-302d-3p	2.65E-02	-2.399	Experimentally Observed	STK4	9.12E-04	2.480
hsa-miR-125b-5p	4.58E-02	-2.252	Experimentally Observed	CDKN2A	4.77E-03	4.656
hsa-miR-24-3p	3.88E-02	-1.832	Experimentally Observed	CDKN2A	4.77E-03	4.656
hsa-miR-1-3p	4.96E-03	-2.715	Experimentally Observed.High (predicted)	IGF1	1.09E-02	2.716
hsa-miR-103a-3p	2.61E-02	-2.121	High (predicted)	P2RY12	1.88E-03	5.073
hsa-miR-103a-3p	2.61E-02	-2.121	High (predicted)	TGFBR2	4.78E-02	3.280
hsa-miR-125b-5p	4.58E-02	-2.252	Experimentally Observed.High (predicted)	CCR5	7.62E-04	13.487

hsa-miR-125b-5p	4.58E-02	-2.252	High (predicted)	TNFAIP3	8.71E-03	4.491
hsa-miR-133a-3p	1.45E-02	-2.375	Experimentally Observed	RUNX2	4.37E-04	5.975
hsa-miR-143-3p	9.54E-03	-3.585	High (predicted)	SELL	6.50E-03	8.128
hsa-miR-143-3p	9.54E-03	-3.585	High (predicted)	SFRP4	1.69E-02	8.336
hsa-miR-15a-5p	1.90E-02	-2.166	Experimentally Observed	FLT3	1.02E-02	5.912
hsa-miR-15a-5p	1.90E-02	-2.166	Experimentally Observed	IGF1	1.09E-02	2.716
hsa-miR-15a-5p	1.90E-02	-2.166	Experimentally Observed.High (predicted)	KCNN4	3.41E-02	3.789
hsa-miR-15a-5p	1.90E-02	-2.166	High (predicted)	SLIT2	1.59E-03	2.813
hsa-miR-106b-5p	3.04E-02	-2.348	High (predicted)	CTSK	9.64E-03	2.968
hsa-miR-106b-5p	3.04E-02	-2.348	Experimentally Observed.High (predicted)	TGFBR2	4.78E-02	3.280
hsa-miR-185-5p	2.67E-02	-2.574	High (predicted)	CD40LG	5.81E-05	37.872
hsa-miR-185-5p	2.67E-02	-2.574	High (predicted)	CTSK	9.64E-03	2.968
hsa-miR-185-5p	2.67E-02	-2.574	High (predicted)	CXCL9	3.94E-04	64.052
hsa-miR-193a-5p	4.68E-02	-1.699	Experimentally Observed	IL2RG	1.92E-05	14.873
hsa-miR-19b-1-5p	1.60E-02	-2.461	High (predicted)	P2RY12	1.88E-03	5.073
hsa-miR-203a-3p	4.17E-03	-3.385	Experimentally Observed	RUNX2	4.37E-04	5.975
hsa-miR-22-3p	7.27E-03	-4.686	High (predicted)	CSF1R	3.28E-03	4.699
hsa-miR-22-3p	7.27E-03	-4.686	High (predicted)	NLRP3	1.43E-02	4.021
hsa-miR-22-3p	7.27E-03	-4.686	High (predicted)	PRKCB	4.05E-04	10.044
hsa-miR-24-3p	3.88E-02	-1.832	High (predicted)	FASLG	1.52E-05	17.708
hsa-miR-24-3p	3.88E-02	-1.832	High (predicted)	IFNG	6.86E-03	5.864
hsa-miR-26a-5p	2.95E-02	-1.913	Experimentally Observed	TGFBR2	4.78E-02	3.280
hsa-miR-27b-3p	6.08E-03	-4.055	High (predicted)	GOLM1	1.77E-02	2.101
hsa-miR-27b-3p	6.08E-03	-4.055	Experimentally Observed	IGF1	1.09E-02	2.716
hsa-miR-302d-3p	2.65E-02	-2.399	High (predicted)	TGFBR2	4.78E-02	3.280
hsa-miR-296-5p	4.12E-02	-2.217	High (predicted)	KCNN4	3.41E-02	3.789

hsa-miR-29c-3p	1.12E-02	-3.142	Experimentally Observed.High (predicted)	COL1A2	2.30E-02	2.879
hsa-miR-29c-3p	1.12E-02	-3.142	Experimentally Observed.High (predicted)	COL3A1	9.35E-03	3.543
hsa-miR-29c-3p	1.12E-02	-3.142	Experimentally Observed.High (predicted)	TNFAIP3	8.71E-03	4.491
hsa-miR-30c-5p	4.34E-02	-1.909	Experimentally Observed.High (predicted)	RUNX2	4.37E-04	5.975
hsa-miR-30c-5p	4.34E-02	-1.909	High (predicted)	SOCS1	1.97E-03	3.929
hsa-miR-328-3p	3.01E-02	-2.110	High (predicted)	PRF1	1.53E-04	6.099
hsa-miR-376a-3p	2.56E-03	-2.627	High (predicted)	IL7	2.24E-03	6.228
hsa-miR-409-3p	4.21E-02	-1.773	High (predicted)	TNFSF14	6.00E-03	8.943
hsa-miR-423-5p	2.15E-02	-2.171	High (predicted)	SELL	6.50E-03	8.128
hsa-miR-423-5p	2.15E-02	-2.171	High (predicted)	THY1	2.81E-02	6.811
hsa-miR-499a-5p	6.18E-03	-3.810	High (predicted)	VAV3	6.05E-03	2.641
hsa-miR-744-5p	3.05E-02	-2.097	High (predicted)	THY1	2.81E-02	6.811
hsa-miR-9-3p	3.90E-03	-2.488	High (predicted)	IL4	2.90E-02	2.336
hsa-miR-9-5p	3.53E-03	-2.877	High (predicted)	VAV3	6.05E-03	2.641

Extracellular matrix

DEM ID	Expr p-value	Expr Fold Change	Confidence	DEG ID	Expr p-value	Expr Fold Change
hsa-miR-212-3p	1.92E-02	-2.587	Experimentally Observed	MMP9	3.63E-02	7.262
hsa-miR-125b-5p	4.58E-02	-2.252	Experimentally Observed	CDKN2A	4.77E-03	4.656
hsa-miR-24-3p	3.88E-02	-1.832	Experimentally Observed	CDKN2A	4.77E-03	4.656
hsa-miR-1-3p	4.96E-03	-2.715	Experimentally Observed.High (predicted)	IGF1	1.09E-02	2.716
hsa-miR-15a-5p	1.90E-02	-2.166	Experimentally Observed	IGF1	1.09E-02	2.716
hsa-miR-106b-5p	3.04E-02	-2.348	High (predicted)	CTSK	9.64E-03	2.968
hsa-miR-185-5p	2.67E-02	-2.574	High (predicted)	CTSK	9.64E-03	2.968

hsa-miR-22-3p	7.27E-03	-4.686	High (predicted)	CSF1R	3.28E-03	4.699
hsa-miR-24-3p	3.88E-02	-1.832	High (predicted)	IFNG	6.86E-03	5.864
hsa-miR-27b-3p	6.08E-03	-4.055	Experimentally Observed	IGF1	1.09E-02	2.716
hsa-miR-29c-3p	1.12E-02	-3.142	Experimentally Observed.High (predicted)	COL3A1	9.35E-03	3.543
hsa-miR-376a-3p	2.56E-03	-2.627	High (predicted)	IL7	2.24E-03	6.228
hsa-miR-423-5p	2.15E-02	-2.171	High (predicted)	THY1	2.81E-02	6.811
hsa-miR-744-5p	3.05E-02	-2.097	High (predicted)	THY1	2.81E-02	6.811
hsa-miR-9-3p	3.90E-03	-2.488	High (predicted)	IL4	2.90E-02	2.336
hsa-miR-155-5p	6.53E-05	3.781	Experimentally Observed	MET	3.40E-03	-2.488
hsa-miR-221-3p	6.50E-03	-4.286	Experimentally Observed.High (predicted)	PIK3R1	4.08E-02	2.670
hsa-miR-29c-3p	1.12E-02	-3.142	Experimentally Observed	PIK3R1	4.08E-02	2.670
hsa-miR-106b-5p	3.04E-02	-2.348	Experimentally Observed	BCL2L11	3.29E-02	2.359
hsa-miR-193a-5p	4.68E-02	-1.699	High (predicted)	BCL2L11	3.29E-02	2.359
hsa-miR-221-3p	6.50E-03	-4.286	Experimentally Observed	BCL2L11	3.29E-02	2.359
hsa-miR-24-3p	3.88E-02	-1.832	High (predicted)	BCL2L11	3.29E-02	2.359
hsa-miR-29c-3p	1.12E-02	-3.142	High (predicted)	CASP8	3.27E-04	6.203
hsa-miR-376a-3p	2.56E-03	-2.627	High (predicted)	CASP8	3.27E-04	6.203
hsa-miR-25-3p	4.50E-02	-2.008	Experimentally Observed	BCL2L11	3.29E-02	2.359
hsa-miR-1-3p	4.96E-03	-2.715	Experimentally Observed	ANPEP	7.34E-03	4.510
hsa-miR-127-3p	4.01E-02	-1.909	High (predicted)	SMPD3	2.70E-04	19.071
hsa-miR-106b-5p	3.04E-02	-2.348	High (predicted)	CCNG2	3.80E-02	2.907
hsa-miR-224-5p	1.81E-02	-3.246	High (predicted)	PARVG	3.85E-03	5.101
hsa-miR-30c-5p	4.34E-02	-1.909	Experimentally Observed	ANPEP	7.34E-03	4.510
hsa-miR-378a-5p	2.80E-03	-3.147	High (predicted)	CCNG2	3.80E-02	2.907
hsa-miR-423-5p	2.15E-02	-2.171	High (predicted)	ANPEP	7.34E-03	4.510

Hypertrophy

DEM ID	Expr p-value	Expr Fold Change	Confidence	DEG ID	Expr p-value	Expr Fold Change
hsa-miR-212-3p	1.92E-02	-2.587	Experimentally Observed	MMP9	3.63E-02	7.262
hsa-miR-1-3p	4.96E-03	-2.715	Experimentally Observed.High (predicted)	IGF1	1.09E-02	2.716
hsa-miR-15a-5p	1.90E-02	-2.166	Experimentally Observed	IGF1	1.09E-02	2.716
hsa-miR-27b-3p	6.08E-03	-4.055	Experimentally Observed	IGF1	1.09E-02	2.716
hsa-miR-376a-3p	2.56E-03	-2.627	High (predicted)	IL7	2.24E-03	6.228
hsa-miR-9-3p	3.90E-03	-2.488	High (predicted)	IL4	2.90E-02	2.336
hsa-miR-221-3p	6.50E-03	-4.286	Experimentally Observed.High (predicted)	PIK3R1	4.08E-02	2.670
hsa-miR-29c-3p	1.12E-02	-3.142	Experimentally Observed	PIK3R1	4.08E-02	2.670
hsa-miR-103a-3p	2.61E-02	-2.121	High (predicted)	TGFBR2	4.78E-02	3.280
hsa-miR-125b-5p	4.58E-02	-2.252	High (predicted)	TNFAIP3	8.71E-03	4.491
hsa-miR-133a-3p	1.45E-02	-2.375	Experimentally Observed	RUNX2	4.37E-04	5.975
hsa-miR-15a-5p	1.90E-02	-2.166	High (predicted)	SLIT2	1.59E-03	2.813
hsa-miR-106b-5p	3.04E-02	-2.348	Experimentally Observed.High (predicted)	TGFBR2	4.78E-02	3.280
hsa-miR-203a-3p	4.17E-03	-3.385	Experimentally Observed	RUNX2	4.37E-04	5.975
hsa-miR-22-3p	7.27E-03	-4.686	High (predicted)	PRKCB	4.05E-04	10.044
hsa-miR-24-3p	3.88E-02	-1.832	High (predicted)	FASLG	1.52E-05	17.708
hsa-miR-26a-5p	2.95E-02	-1.913	Experimentally Observed	TGFBR2	4.78E-02	3.280
hsa-miR-302d-3p	2.65E-02	-2.399	High (predicted)	TGFBR2	4.78E-02	3.280
hsa-miR-29c-3p	1.12E-02	-3.142	Experimentally Observed.High (predicted)	TNFAIP3	8.71E-03	4.491
hsa-miR-30c-5p	4.34E-02	-1.909	Experimentally Observed.High (predicted)	RUNX2	4.37E-04	5.975

hsa-miR-499a-5p	6.18E-03	-3.810	High (predicted)	VAV3	6.05E-03	2.641
hsa-miR-9-5p	3.53E-03	-2.877	High (predicted)	VAV3	6.05E-03	2.641
hsa-miR-1-3p	4.96E-03	-2.715	High (predicted)	NFATC2	1.62E-02	3.036
hsa-miR-143-3p	9.54E-03	-3.585	High (predicted)	IL18	1.27E-02	4.807
hsa-miR-155-5p	6.53E-05	3.781	Experimentally Observed	RHEB	2.65E-02	-2.088
hsa-miR-106b-5p	3.04E-02	-2.348	Experimentally Observed	E2F2	2.72E-02	4.283
hsa-miR-23b-3p	1.01E-02	-3.178	High (predicted)	IL18	1.27E-02	4.807
hsa-miR-24-3p	3.88E-02	-1.832	Experimentally Observed	E2F2	2.72E-02	4.283
hsa-miR-296-5p	4.12E-02	-2.217	High (predicted)	E2F2	2.72E-02	4.283
hsa-miR-365a-3p	2.14E-03	-2.687	High (predicted)	E2F2	2.72E-02	4.283
hsa-miR-422a	9.63E-03	-2.624	High (predicted)	PIK3CG	1.09E-03	5.372

Contraction of heart and of cardiac muscle and contractility of heart

DEM ID	Expr p-value	Expr Fold Change	Confidence	DEG ID	Expr p-value	Expr Fold Change
hsa-miR-212-3p	1.92E-02	-2.587	Experimentally Observed	MMP9	3.63E-02	7.262
hsa-miR-22-3p	7.27E-03	-4.686	High (predicted)	PRKCB	4.05E-04	10.044
hsa-miR-24-3p	3.88E-02	-1.832	High (predicted)	FASLG	1.52E-05	17.708
hsa-miR-499a-5p	6.18E-03	-3.810	High (predicted)	VAV3	6.05E-03	2.641
hsa-miR-9-5p	3.53E-03	-2.877	High (predicted)	VAV3	6.05E-03	2.641
hsa-miR-143-3p	9.54E-03	-3.585	High (predicted)	IL18	1.27E-02	4.807
hsa-miR-23b-3p	1.01E-02	-3.178	High (predicted)	IL18	1.27E-02	4.807
hsa-miR-422a	9.63E-03	-2.624	High (predicted)	PIK3CG	1.09E-03	5.372
hsa-miR-24-3p	3.88E-02	-1.832	High (predicted)	IFNG	6.86E-03	5.864
hsa-miR-155-5p	6.53E-05	3.781	Experimentally Observed	MET	3.40E-03	-2.488
hsa-miR-296-5p	4.12E-02	-2.217	High (predicted)	TRPM2	2.25E-03	3.771

Inflammation

DEM ID	Expr p-value	Expr Fold Change	Confidence	DEG ID	Expr p-value	Expr Fold Change
hsa-miR-212-3p	1.92E-02	-2.587	Experimentally Observed	MMP9	3.63E-02	7.262
hsa-miR-22-3p	7.27E-03	-4.686	High (predicted)	PRKCB	4.05E-04	10.044
hsa-miR-24-3p	3.88E-02	-1.832	High (predicted)	FASLG	1.52E-05	17.708
hsa-miR-499a-5p	6.18E-03	-3.810	High (predicted)	VAV3	6.05E-03	2.641

hsa-miR-9-5p	3.53E-03	-2.877	High (predicted)	VAV3	6.05E-03	2.641
hsa-miR-143-3p	9.54E-03	-3.585	High (predicted)	IL18	1.27E-02	4.807
hsa-miR-23b-3p	1.01E-02	-3.178	High (predicted)	IL18	1.27E-02	4.807
hsa-miR-422a	9.63E-03	-2.624	High (predicted)	PIK3CG	1.09E-03	5.372
hsa-miR-24-3p	3.88E-02	-1.832	High (predicted)	IFNG	6.86E-03	5.864
hsa-miR-296-5p	4.12E-02	-2.217	High (predicted)	TRPM2	2.25E-03	3.771
hsa-miR-1-3p	4.96E-03	-2.715	Experimentally Observed.High (predicted)	IGF1	1.09E-02	2.716
hsa-miR-15a-5p	1.90E-02	-2.166	Experimentally Observed	IGF1	1.09E-02	2.716
hsa-miR-27b-3p	6.08E-03	-4.055	Experimentally Observed	IGF1	1.09E-02	2.716
hsa-miR-376a-3p	2.56E-03	-2.627	High (predicted)	IL7	2.24E-03	6.228
hsa-miR-9-3p	3.90E-03	-2.488	High (predicted)	IL4	2.90E-02	2.336
hsa-miR-221-3p	6.50E-03	-4.286	Experimentally Observed.High (predicted)	PIK3R1	4.08E-02	2.670
hsa-miR-29c-3p	1.12E-02	-3.142	Experimentally Observed	PIK3R1	4.08E-02	2.670
hsa-miR-103a-3p	2.61E-02	-2.121	High (predicted)	TGFBR2	4.78E-02	3.280
hsa-miR-125b-5p	4.58E-02	-2.252	High (predicted)	TNFAIP3	8.71E-03	4.491
hsa-miR-15a-5p	1.90E-02	-2.166	High (predicted)	SLIT2	1.59E-03	2.813
hsa-miR-106b-5p	3.04E-02	-2.348	Experimentally Observed.High (predicted)	TGFBR2	4.78E-02	3.280
hsa-miR-26a-5p	2.95E-02	-1.913	Experimentally Observed	TGFBR2	4.78E-02	3.280
hsa-miR-302d-3p	2.65E-02	-2.399	High (predicted)	TGFBR2	4.78E-02	3.280
hsa-miR-29c-3p	1.12E-02	-3.142	Experimentally Observed.High (predicted)	TNFAIP3	8.71E-03	4.491
hsa-miR-1-3p	4.96E-03	-2.715	High (predicted)	NFATC2	1.62E-02	3.036
hsa-miR-106b-5p	3.04E-02	-2.348	Experimentally Observed	E2F2	2.72E-02	4.283
hsa-miR-24-3p	3.88E-02	-1.832	Experimentally Observed	E2F2	2.72E-02	4.283
hsa-miR-296-5p	4.12E-02	-2.217	High (predicted)	E2F2	2.72E-02	4.283
hsa-miR-365a-3p	2.14E-03	-2.687	High (predicted)	E2F2	2.72E-02	4.283

hsa-miR-125b-5p	4.58E-02	-2.252	Experimentally Observed	CDKN2A	4.77E-03	4.656
hsa-miR-24-3p	3.88E-02	-1.832	Experimentally Observed	CDKN2A	4.77E-03	4.656
hsa-miR-22-3p	7.27E-03	-4.686	High (predicted)	CSF1R	3.28E-03	4.699
hsa-miR-29c-3p	1.12E-02	-3.142	Experimentally Observed.High (predicted)	COL3A1	9.35E-03	3.543
hsa-miR-106b-5p	3.04E-02	-2.348	Experimentally Observed	BCL2L11	3.29E-02	2.359
hsa-miR-193a-5p	4.68E-02	-1.699	High (predicted)	BCL2L11	3.29E-02	2.359
hsa-miR-221-3p	6.50E-03	-4.286	Experimentally Observed	BCL2L11	3.29E-02	2.359
hsa-miR-24-3p	3.88E-02	-1.832	High (predicted)	BCL2L11	3.29E-02	2.359
hsa-miR-29c-3p	1.12E-02	-3.142	High (predicted)	CASP8	3.27E-04	6.203
hsa-miR-376a-3p	2.56E-03	-2.627	High (predicted)	CASP8	3.27E-04	6.203
hsa-miR-25-3p	4.50E-02	-2.008	Experimentally Observed	BCL2L11	3.29E-02	2.359
hsa-miR-103a-3p	2.61E-02	-2.121	High (predicted)	P2RY12	1.88E-03	5.073
hsa-miR-125b-5p	4.58E-02	-2.252	Experimentally Observed.High (predicted)	CCR5	7.62E-04	13.487
hsa-miR-143-3p	9.54E-03	-3.585	High (predicted)	SELL	6.50E-03	8.128
hsa-miR-15a-5p	1.90E-02	-2.166	Experimentally Observed	FLT3	1.02E-02	5.912
hsa-miR-15a-5p	1.90E-02	-2.166	Experimentally Observed.High (predicted)	KCNN4	3.41E-02	3.789
hsa-miR-185-5p	2.67E-02	-2.574	High (predicted)	CD40LG	5.81E-05	37.872
hsa-miR-185-5p	2.67E-02	-2.574	High (predicted)	CXCL9	3.94E-04	64.052
hsa-miR-193a-5p	4.68E-02	-1.699	Experimentally Observed	IL2RG	1.92E-05	14.873
hsa-miR-19b-1-5p	1.60E-02	-2.461	High (predicted)	P2RY12	1.88E-03	5.073
hsa-miR-22-3p	7.27E-03	-4.686	High (predicted)	NLRP3	1.43E-02	4.021
hsa-miR-296-5p	4.12E-02	-2.217	High (predicted)	KCNN4	3.41E-02	3.789
hsa-miR-29c-3p	1.12E-02	-3.142	Experimentally Observed.High (predicted)	COL1A2	2.30E-02	2.879
hsa-miR-30c-5p	4.34E-02	-1.909	High (predicted)	SOCS1	1.97E-03	3.929

hsa-miR-328-3p	3.01E-02	-2.110	High (predicted)	PRF1	1.53E-04	6.099
hsa-miR-409-3p	4.21E-02	-1.773	High (predicted)	TNFSF14	6.00E-03	8.943
hsa-miR-423-5p	2.15E-02	-2.171	High (predicted)	SELL	6.50E-03	8.128
hsa-miR-99a-5p	1.43E-02	-2.885	High (predicted)	VNN1	3.60E-02	4.331
hsa-miR-221-3p	6.50E-03	-4.286	High (predicted)	HNMT	1.89E-03	2.539
hsa-miR-101-3p	4.17E-02	-2.891	High (predicted)	DDIT4	6.61E-03	4.020
hsa-miR-22-3p	7.27E-03	-4.686	High (predicted)	DDIT4	6.61E-03	4.020
hsa-miR-221-3p	6.50E-03	-4.286	Experimentally Observed	DDIT4	6.61E-03	4.020
hsa-miR-1-3p	4.96E-03	-2.715	Experimentally Observed	NOTCH2	2.41E-02	2.302
hsa-miR-1-3p	4.96E-03	-2.715	High (predicted)	TNFAIP8L2	2.55E-02	4.121
hsa-miR-1-3p	4.96E-03	-2.715	Experimentally Observed	UNC93B1	1.33E-02	2.316
hsa-miR-99a-5p	1.43E-02	-2.885	High (predicted)	SPN	2.00E-02	2.393
hsa-miR-101-3p	4.17E-02	-2.891	High (predicted)	ASPN	5.56E-03	5.012
hsa-miR-101-3p	4.17E-02	-2.891	High (predicted)	COTL1	1.84E-02	3.905
hsa-miR-101-3p	4.17E-02	-2.891	Experimentally Observed	ICOS	6.66E-03	5.007
hsa-miR-103a-3p	2.61E-02	-2.121	High (predicted)	BTLA	2.37E-03	8.616
hsa-miR-103a-3p	2.61E-02	-2.121	Experimentally Observed	ICOS	6.66E-03	5.007
hsa-miR-103a-3p	2.61E-02	-2.121	High (predicted)	KLRB1	5.82E-04	17.677
hsa-miR-1233-3p	7.57E-03	-2.523	High (predicted)	BMF	6.39E-03	3.138
hsa-miR-1233-3p	7.57E-03	-2.523	High (predicted)	CCL14	2.45E-02	4.110
hsa-miR-1233-3p	7.57E-03	-2.523	High (predicted)	CCL28	3.46E-03	4.514
hsa-miR-1233-3p	7.57E-03	-2.523	High (predicted)	CLEC4D	2.08E-03	7.891
hsa-miR-1233-3p	7.57E-03	-2.523	High (predicted)	SH2D1A	5.71E-05	11.640
hsa-miR-1233-3p	7.57E-03	-2.523	High (predicted)	UBE2L6	4.94E-02	2.116
hsa-miR-125b-5p	4.58E-02	-2.252	Experimentally Observed.High (predicted)	BMF	6.39E-03	3.138
hsa-miR-125b-5p	4.58E-02	-2.252	High (predicted)	CD69	1.27E-05	20.025
hsa-miR-125b-5p	4.58E-02	-2.252	High (predicted)	GCNT1	4.48E-03	3.983
hsa-miR-125b-5p	4.58E-02	-2.252	High (predicted)	IRF4	3.49E-03	5.957
hsa-miR-125b-5p	4.58E-02	-2.252	High (predicted)	PRDM1	6.82E-05	26.789
hsa-miR-125b-5p	4.58E-02	-2.252	High (predicted)	PSTPIP2	8.71E-03	4.463
hsa-miR-125b-5p	4.58E-02	-2.252	High (predicted)	SEMA4D	3.00E-03	8.821
hsa-miR-127-3p	4.01E-02	-1.909	High (predicted)	HLA-DOA	5.44E-03	5.601

hsa-miR-127-3p	4.01E-02	-1.909	Experimentally Observed	PRDM1	6.82E-05	26.789
hsa-miR-127-3p	4.01E-02	-1.909	High (predicted)	SDC1	1.32E-02	3.663
hsa-miR-133a-3p	1.45E-02	-2.375	High (predicted)	CXCL11	2.07E-02	14.705
hsa-miR-143-3p	9.54E-03	-3.585	High (predicted)	HLA-DOA	5.44E-03	5.601
hsa-miR-145-3p	3.93E-03	-2.508	High (predicted)	GFI1	3.78E-06	40.106
hsa-miR-148a-3p	4.41E-02	-2.648	High (predicted)	CCL14	2.45E-02	4.110
hsa-miR-148a-3p	4.41E-02	-2.648	High (predicted)	CEBPE	4.33E-03	3.871
hsa-miR-148a-3p	4.41E-02	-2.648	High (predicted)	HLA-A	5.37E-03	2.185
hsa-miR-148a-3p	4.41E-02	-2.648	High (predicted)	MAFB	2.66E-02	3.069
hsa-miR-151a-3p	2.50E-02	-4.893	High (predicted)	BTLA	2.37E-03	8.616
hsa-miR-151a-3p	2.50E-02	-4.893	High (predicted)	CD48	4.49E-05	25.693
hsa-miR-151a-5p	7.94E-03	-2.319	High (predicted)	FANCA	1.44E-03	4.996
hsa-miR-151a-5p	7.94E-03	-2.319	High (predicted)	GNLY	5.12E-03	6.940
hsa-miR-15a-5p	1.90E-02	-2.166	High (predicted)	BTLA	2.37E-03	8.616
hsa-miR-15a-5p	1.90E-02	-2.166	Experimentally Observed	NOTCH2	2.41E-02	2.302
hsa-miR-15a-5p	1.90E-02	-2.166	High (predicted)	TNFSF13B	5.53E-03	5.399
hsa-miR-15a-5p	1.90E-02	-2.166	Experimentally Observed	WIPF1	4.75E-03	2.815
hsa-miR-106b-5p	3.04E-02	-2.348	High (predicted)	CD69	1.27E-05	20.025
hsa-miR-106b-5p	3.04E-02	-2.348	High (predicted)	PDCD1LG2	3.30E-02	2.707
hsa-miR-106b-5p	3.04E-02	-2.348	High (predicted)	STK17B	2.96E-02	3.004
hsa-miR-185-5p	2.67E-02	-2.574	High (predicted)	AIM2	2.70E-04	34.077
hsa-miR-185-5p	2.67E-02	-2.574	High (predicted)	BTLA	2.37E-03	8.616
hsa-miR-185-5p	2.67E-02	-2.574	High (predicted)	CD84	1.14E-04	11.117
hsa-miR-185-5p	2.67E-02	-2.574	High (predicted)	CXCL16	3.62E-02	3.047
hsa-miR-185-5p	2.67E-02	-2.574	High (predicted)	SAMD9L	2.88E-02	2.984
hsa-miR-185-5p	2.67E-02	-2.574	High (predicted)	TNFSF13	2.93E-02	2.186
hsa-miR-192-5p	3.00E-02	-1.896	High (predicted)	CD48	4.49E-05	25.693
hsa-miR-193a-5p	4.68E-02	-1.699	High (predicted)	CD27	6.67E-05	14.856
hsa-miR-193a-5p	4.68E-02	-1.699	High (predicted)	CSF2RA	2.14E-05	6.063
hsa-miR-19b-1-5p	1.60E-02	-2.461	High (predicted)	CSF2RA	2.14E-05	6.063
hsa-miR-19b-1-5p	1.60E-02	-2.461	High (predicted)	CYTIP	3.82E-05	12.099
hsa-miR-19b-1-5p	1.60E-02	-2.461	High (predicted)	GPR174	5.37E-03	4.586
hsa-miR-20a-3p	1.49E-02	-2.092	High (predicted)	ALOX5AP	2.96E-02	4.646
hsa-miR-20a-3p	1.49E-02	-2.092	High (predicted)	BATF	1.04E-03	13.327
hsa-miR-20a-3p	1.49E-02	-2.092	High (predicted)	CCL4	6.28E-06	20.663
hsa-miR-20a-3p	1.49E-02	-2.092	High (predicted)	TRAF1	7.95E-04	5.095

hsa-miR-22-3p	7.27E-03	-4.686	High (predicted)	GPR132	7.40E-03	3.341
hsa-miR-22-3p	7.27E-03	-4.686	High (predicted)	TLR8	6.97E-03	7.087
hsa-miR-22-5p	3.81E-03	-2.591	High (predicted)	RGS1	6.34E-04	19.413
hsa-miR-221-3p	6.50E-03	-4.286	Experimentally Observed	BMF	6.39E-03	3.138
hsa-miR-221-3p	6.50E-03	-4.286	High (predicted)	CXCL11	2.07E-02	14.705
hsa-miR-221-3p	6.50E-03	-4.286	High (predicted)	MS4A1	1.19E-02	5.117
hsa-miR-23b-3p	1.01E-02	-3.178	High (predicted)	MRC1	1.63E-02	4.006
hsa-miR-23b-3p	1.01E-02	-3.178	High (predicted)	SCG5	3.55E-02	2.851
hsa-miR-24-3p	3.88E-02	-1.832	High (predicted)	CXCR2	3.05E-02	2.404
hsa-miR-24-3p	3.88E-02	-1.832	High (predicted)	PIM2	1.18E-03	7.447
hsa-miR-24-3p	3.88E-02	-1.832	High (predicted)	SPN	2.00E-02	2.393
hsa-miR-27b-3p	6.08E-03	-4.055	High (predicted)	MS4A7	3.15E-02	2.802
hsa-miR-302d-3p	2.65E-02	-2.399	High (predicted)	PDCD1LG2	3.30E-02	2.707
hsa-miR-296-5p	4.12E-02	-2.217	High (predicted)	BMF	6.39E-03	3.138
hsa-miR-296-5p	4.12E-02	-2.217	High (predicted)	CCL28	3.46E-03	4.514
hsa-miR-296-5p	4.12E-02	-2.217	High (predicted)	CD300LF	5.57E-03	4.946
hsa-miR-296-5p	4.12E-02	-2.217	High (predicted)	CD6	9.05E-06	40.352
hsa-miR-296-5p	4.12E-02	-2.217	High (predicted)	CD8A	1.55E-05	25.013
hsa-miR-296-5p	4.12E-02	-2.217	High (predicted)	CXCL10	1.71E-02	12.848
hsa-miR-296-5p	4.12E-02	-2.217	High (predicted)	TNFSF13	2.93E-02	2.186
hsa-miR-29c-3p	1.12E-02	-3.142	High (predicted)	BMF	6.39E-03	3.138
hsa-miR-29c-3p	1.12E-02	-3.142	High (predicted)	COL4A4	3.91E-02	2.390
hsa-miR-29c-3p	1.12E-02	-3.142	High (predicted)	ICOS	6.66E-03	5.007
hsa-miR-29c-3p	1.12E-02	-3.142	High (predicted)	RGS1	6.34E-04	19.413
hsa-miR-29c-3p	1.12E-02	-3.142	High (predicted)	TNFRSF9	6.78E-03	2.343
hsa-miR-30c-5p	4.34E-02	-1.909	High (predicted)	CCL19	1.76E-02	16.140
hsa-miR-30c-5p	4.34E-02	-1.909	High (predicted)	PRDM1	6.82E-05	26.789
hsa-miR-30c-5p	4.34E-02	-1.909	High (predicted)	SCN9A	2.16E-02	2.735
hsa-miR-30c-5p	4.34E-02	-1.909	High (predicted)	STIM2	3.64E-02	2.026
hsa-miR-30c-5p	4.34E-02	-1.909	High (predicted)	TNFSF13B	5.53E-03	5.399
hsa-miR-324-5p	2.03E-02	-2.855	High (predicted)	CD84	1.14E-04	11.117
hsa-miR-324-5p	2.03E-02	-2.855	High (predicted)	CTLA4	3.83E-03	10.680
hsa-miR-324-5p	2.03E-02	-2.855	High (predicted)	P2RY13	3.36E-02	3.597
hsa-miR-324-5p	2.03E-02	-2.855	High (predicted)	S1PR4	1.78E-04	7.144
hsa-miR-328-3p	3.01E-02	-2.110	High (predicted)	PSTPIP2	8.71E-03	4.463
hsa-miR-335-5p	2.05E-02	-2.221	High (predicted)	ALOX5AP	2.96E-02	4.646
hsa-miR-335-5p	2.05E-02	-2.221	High (predicted)	PTPN22	7.35E-05	16.747
hsa-miR-345-5p	1.38E-02	-2.108	High (predicted)	RGS1	6.34E-04	19.413

hsa-miR-345-5p	1.38E-02	-2.108	High (predicted)	UBE2L6	4.94E-02	2.116
hsa-miR-376a-3p	2.56E-03	-2.627	High (predicted)	LILRA2	3.91E-02	3.122
hsa-miR-376a-3p	2.56E-03	-2.627	High (predicted)	RGS1	6.34E-04	19.413
hsa-miR-376c-3p	1.95E-02	-1.990	High (predicted)	PARP4	2.14E-02	2.002
hsa-miR-422a	9.63E-03	-2.624	High (predicted)	CCR7	1.76E-03	14.696
hsa-miR-378a-5p	2.80E-03	-3.147	High (predicted)	FRZB	1.62E-03	5.883
hsa-miR-379-5p	1.27E-02	-2.367	High (predicted)	CXCL11	2.07E-02	14.705
hsa-miR-379-5p	1.27E-02	-2.367	High (predicted)	HLA-E	1.56E-02	2.118
hsa-miR-379-5p	1.27E-02	-2.367	High (predicted)	ICOS	6.66E-03	5.007
hsa-miR-379-5p	1.27E-02	-2.367	High (predicted)	IL18RAP	1.28E-02	5.958
hsa-miR-379-5p	1.27E-02	-2.367	High (predicted)	SIT1	1.59E-04	13.192
hsa-miR-409-3p	4.21E-02	-1.773	High (predicted)	CCL28	3.46E-03	4.514
hsa-miR-423-5p	2.15E-02	-2.171	High (predicted)	BMF	6.39E-03	3.138
hsa-miR-423-5p	2.15E-02	-2.171	High (predicted)	CD72	7.17E-03	7.274
hsa-miR-423-5p	2.15E-02	-2.171	High (predicted)	CD8A	1.55E-05	25.013
hsa-miR-423-5p	2.15E-02	-2.171	High (predicted)	CXCL16	3.62E-02	3.047
hsa-miR-423-5p	2.15E-02	-2.171	High (predicted)	PTK2B	3.13E-03	3.355
hsa-miR-423-5p	2.15E-02	-2.171	High (predicted)	RAC2	1.02E-03	4.144
hsa-miR-423-5p	2.15E-02	-2.171	High (predicted)	RGS1	6.34E-04	19.413
hsa-miR-423-5p	2.15E-02	-2.171	High (predicted)	SRCIN1	3.40E-03	5.005
hsa-miR-451a	1.69E-02	-3.669	High (predicted)	BATF	1.04E-03	13.327
hsa-miR-451a	1.69E-02	-3.669	High (predicted)	CXCL16	3.62E-02	3.047
hsa-miR-455-3p	1.49E-02	-3.286	High (predicted)	STK17B	2.96E-02	3.004
hsa-miR-455-3p	1.49E-02	-3.286	High (predicted)	UBE2L6	4.94E-02	2.116
hsa-miR-455-3p	1.49E-02	-3.286	High (predicted)	XCL1	4.38E-04	19.986
hsa-miR-455-5p	1.18E-02	-2.969	High (predicted)	CD200R1	2.04E-02	2.552
hsa-miR-455-5p	1.18E-02	-2.969	High (predicted)	FCAR	1.64E-02	2.964
hsa-miR-455-5p	1.18E-02	-2.969	High (predicted)	MS4A7	3.15E-02	2.802
hsa-miR-486-5p	4.58E-02	-1.997	High (predicted)	CD247	3.23E-05	17.934
hsa-miR-486-5p	4.58E-02	-1.997	High (predicted)	PTGDR	3.00E-03	5.175
hsa-miR-494-3p	8.19E-03	-3.473	High (predicted)	SLAMF1	2.85E-02	4.949
hsa-miR-494-3p	8.19E-03	-3.473	High (predicted)	UBE2L6	4.94E-02	2.116
hsa-miR-499a-5p	6.18E-03	-3.810	High (predicted)	CXCL11	2.07E-02	14.705
hsa-miR-532-5p	3.58E-02	-1.943	Experimentally Observed	RUNX3	1.48E-05	16.778
hsa-miR-642a-5p	7.76E-03	-2.482	High (predicted)	LTB	1.35E-02	8.556
hsa-miR-28-5p	2.88E-02	-2.216	High (predicted)	CCL28	3.46E-03	4.514
hsa-miR-28-5p	2.88E-02	-2.216	High (predicted)	EOMES	3.78E-06	27.454
hsa-miR-744-5p	3.05E-02	-2.097	High (predicted)	BCL11B	1.45E-04	13.713

hsa-miR-744-5p	3.05E-02	-2.097	High (predicted)	BLK	8.11E-04	12.334
hsa-miR-744-5p	3.05E-02	-2.097	High (predicted)	CD37	3.12E-04	9.603
hsa-miR-744-5p	3.05E-02	-2.097	High (predicted)	MMP25	1.56E-03	6.090
hsa-miR-744-5p	3.05E-02	-2.097	High (predicted)	SRCIN1	3.40E-03	5.005
hsa-miR-744-5p	3.05E-02	-2.097	High (predicted)	TCF7	1.70E-02	2.991
hsa-miR-9-3p	3.90E-03	-2.488	High (predicted)	MDK	4.11E-02	2.352
hsa-miR-9-5p	3.53E-03	-2.877	High (predicted)	CXCL11	2.07E-02	14.705
hsa-miR-9-5p	3.53E-03	-2.877	Experimentally Observed.High (predicted)	PRDM1	6.82E-05	26.789
hsa-miR-25-3p	4.50E-02	-2.008	High (predicted)	ASPN	5.56E-03	5.012
hsa-miR-25-3p	4.50E-02	-2.008	High (predicted)	BTLA	2.37E-03	8.616
hsa-miR-25-3p	4.50E-02	-2.008	High (predicted)	CD69	1.27E-05	20.025
hsa-miR-25-3p	4.50E-02	-2.008	Experimentally Observed	IKZF1	1.50E-05	17.415
hsa-miR-25-3p	4.50E-02	-2.008	High (predicted)	TAGAP	2.66E-04	17.919
hsa-miR-95-3p	3.98E-02	-2.056	High (predicted)	KMO	2.30E-02	3.037
hsa-miR-155-5p	6.53E-05	3.781	Experimentally Observed	MET	3.40E-03	-2.488

IFNG induced genes and Th1 response

DEM ID	Expr p-value	Expr Fold Change	Confidence	DEG ID	Expr p-value	Expr Fold Change
hsa-miR-24-3p	3.88E-02	-1.832	High (predicted)	FASLG	1.52E-05	17.708
hsa-miR-143-3p	9.54E-03	-3.585	High (predicted)	IL18	1.27E-02	4.807
hsa-miR-23b-3p	1.01E-02	-3.178	High (predicted)	IL18	1.27E-02	4.807
hsa-miR-422a	9.63E-03	-2.624	High (predicted)	PIK3CG	1.09E-03	5.372
hsa-miR-24-3p	3.88E-02	-1.832	High (predicted)	IFNG	6.86E-03	5.864
hsa-miR-9-3p	3.90E-03	-2.488	High (predicted)	IL4	2.90E-02	2.336
hsa-miR-221-3p	6.50E-03	-4.286	Experimentally Observed.High (predicted)	PIK3R1	4.08E-02	2.670
hsa-miR-29c-3p	1.12E-02	-3.142	Experimentally Observed	PIK3R1	4.08E-02	2.670
hsa-miR-1-3p	4.96E-03	-2.715	High (predicted)	NFATC2	1.62E-02	3.036
hsa-miR-125b-5p	4.58E-02	-2.252	Experimentally Observed	CDKN2A	4.77E-03	4.656
hsa-miR-24-3p	3.88E-02	-1.832	Experimentally Observed	CDKN2A	4.77E-03	4.656

hsa-miR-125b-5p	4.58E-02	-2.252	Experimentally Observed.High (predicted)	CCR5	7.62E-04	13.487
hsa-miR-143-3p	9.54E-03	-3.585	High (predicted)	SELL	6.50E-03	8.128
hsa-miR-185-5p	2.67E-02	-2.574	High (predicted)	CD40LG	5.81E-05	37.872
hsa-miR-185-5p	2.67E-02	-2.574	High (predicted)	CXCL9	3.94E-04	64.052
hsa-miR-193a-5p	4.68E-02	-1.699	Experimentally Observed	IL2RG	1.92E-05	14.873
hsa-miR-30c-5p	4.34E-02	-1.909	High (predicted)	SOCS1	1.97E-03	3.929
hsa-miR-409-3p	4.21E-02	-1.773	High (predicted)	TNFSF14	6.00E-03	8.943
hsa-miR-423-5p	2.15E-02	-2.171	High (predicted)	SELL	6.50E-03	8.128
hsa-miR-1-3p	4.96E-03	-2.715	Experimentally Observed	NOTCH2	2.41E-02	2.302
hsa-miR-101-3p	4.17E-02	-2.891	Experimentally Observed	ICOS	6.66E-03	5.007
hsa-miR-103a-3p	2.61E-02	-2.121	High (predicted)	BTLA	2.37E-03	8.616
hsa-miR-103a-3p	2.61E-02	-2.121	Experimentally Observed	ICOS	6.66E-03	5.007
hsa-miR-1233-3p	7.57E-03	-2.523	High (predicted)	CLEC4D	2.08E-03	7.891
hsa-miR-1233-3p	7.57E-03	-2.523	High (predicted)	SH2D1A	5.71E-05	11.640
hsa-miR-1233-3p	7.57E-03	-2.523	High (predicted)	UBE2L6	4.94E-02	2.116
hsa-miR-125b-5p	4.58E-02	-2.252	High (predicted)	GCNT1	4.48E-03	3.983
hsa-miR-125b-5p	4.58E-02	-2.252	High (predicted)	IRF4	3.49E-03	5.957
hsa-miR-127-3p	4.01E-02	-1.909	High (predicted)	HLA-DOA	5.44E-03	5.601
hsa-miR-133a-3p	1.45E-02	-2.375	High (predicted)	CXCL11	2.07E-02	14.705
hsa-miR-143-3p	9.54E-03	-3.585	High (predicted)	HLA-DOA	5.44E-03	5.601
hsa-miR-148a-3p	4.41E-02	-2.648	High (predicted)	HLA-A	5.37E-03	2.185
hsa-miR-148a-3p	4.41E-02	-2.648	High (predicted)	MAFB	2.66E-02	3.069
hsa-miR-151a-3p	2.50E-02	-4.893	High (predicted)	BTLA	2.37E-03	8.616
hsa-miR-15a-5p	1.90E-02	-2.166	High (predicted)	BTLA	2.37E-03	8.616
hsa-miR-15a-5p	1.90E-02	-2.166	Experimentally Observed	NOTCH2	2.41E-02	2.302
hsa-miR-185-5p	2.67E-02	-2.574	High (predicted)	BTLA	2.37E-03	8.616
hsa-miR-20a-3p	1.49E-02	-2.092	High (predicted)	CCL4	6.28E-06	20.663
hsa-miR-20a-3p	1.49E-02	-2.092	High (predicted)	TRAF1	7.95E-04	5.095
hsa-miR-22-5p	3.81E-03	-2.591	High (predicted)	RGS1	6.34E-04	19.413
hsa-miR-221-3p	6.50E-03	-4.286	High (predicted)	CXCL11	2.07E-02	14.705
hsa-miR-23b-3p	1.01E-02	-3.178	High (predicted)	MRC1	1.63E-02	4.006
hsa-miR-24-3p	3.88E-02	-1.832	High (predicted)	CXCR2	3.05E-02	2.404

hsa-miR-296-5p	4.12E-02	-2.217	High (predicted)	CXCL10	1.71E-02	12.848
hsa-miR-29c-3p	1.12E-02	-3.142	High (predicted)	ICOS	6.66E-03	5.007
hsa-miR-29c-3p	1.12E-02	-3.142	High (predicted)	RGS1	6.34E-04	19.413
hsa-miR-324-5p	2.03E-02	-2.855	High (predicted)	CTLA4	3.83E-03	10.680
hsa-miR-324-5p	2.03E-02	-2.855	High (predicted)	S1PR4	1.78E-04	7.144
hsa-miR-335-5p	2.05E-02	-2.221	High (predicted)	PTPN22	7.35E-05	16.747
hsa-miR-345-5p	1.38E-02	-2.108	High (predicted)	RGS1	6.34E-04	19.413
hsa-miR-345-5p	1.38E-02	-2.108	High (predicted)	UBE2L6	4.94E-02	2.116
hsa-miR-376a-3p	2.56E-03	-2.627	High (predicted)	RGS1	6.34E-04	19.413
hsa-miR-379-5p	1.27E-02	-2.367	High (predicted)	CXCL11	2.07E-02	14.705
hsa-miR-379-5p	1.27E-02	-2.367	High (predicted)	HLA-E	1.56E-02	2.118
hsa-miR-379-5p	1.27E-02	-2.367	High (predicted)	ICOS	6.66E-03	5.007
hsa-miR-379-5p	1.27E-02	-2.367	High (predicted)	SIT1	1.59E-04	13.192
hsa-miR-423-5p	2.15E-02	-2.171	High (predicted)	RGS1	6.34E-04	19.413
hsa-miR-455-3p	1.49E-02	-3.286	High (predicted)	UBE2L6	4.94E-02	2.116
hsa-miR-486-5p	4.58E-02	-1.997	High (predicted)	CD247	3.23E-05	17.934
hsa-miR-494-3p	8.19E-03	-3.473	High (predicted)	UBE2L6	4.94E-02	2.116
hsa-miR-499a-5p	6.18E-03	-3.810	High (predicted)	CXCL11	2.07E-02	14.705
hsa-miR-9-5p	3.53E-03	-2.877	High (predicted)	CXCL11	2.07E-02	14.705
hsa-miR-25-3p	4.50E-02	-2.008	High (predicted)	BTLA	2.37E-03	8.616
hsa-miR-106b-5p	3.04E-02	-2.348	High (predicted)	CCNG2	3.80E-02	2.907
hsa-miR-378a-5p	2.80E-03	-3.147	High (predicted)	CCNG2	3.80E-02	2.907
hsa-miR-27b-3p	6.08E-03	-4.055	High (predicted)	GOLM1	1.77E-02	2.101
hsa-miR-1-3p	4.96E-03	-2.715	High (predicted)	SAMSN1	9.63E-03	7.424
hsa-miR-133a-3p	1.45E-02	-2.375	High (predicted)	CRTAM	3.17E-05	27.814
hsa-miR-145-3p	3.93E-03	-2.508	High (predicted)	CD226	1.47E-03	6.472
hsa-miR-145-5p	2.04E-03	-3.042	Experimentally Observed	PARP8	9.04E-05	4.981
hsa-miR-15a-5p	1.90E-02	-2.166	High (predicted)	CNOT6L	3.85E-02	2.125
hsa-miR-193a-5p	4.68E-02	-1.699	High (predicted)	KLRD1	5.02E-05	16.503
hsa-miR-23b-3p	1.01E-02	-3.178	High (predicted)	FUT4	4.21E-03	2.243
hsa-miR-24-2-5p	2.27E-02	-2.073	High (predicted)	UBA7	8.60E-03	2.356
hsa-miR-27b-3p	6.08E-03	-4.055	High (predicted)	STYK1	1.55E-05	14.721
hsa-miR-29c-3p	1.12E-02	-3.142	High (predicted)	COL5A1	2.13E-02	2.232
hsa-miR-29c-3p	1.12E-02	-3.142	High (predicted)	NAV1	3.06E-02	2.271
hsa-miR-324-5p	2.03E-02	-2.855	High (predicted)	CRTAM	3.17E-05	27.814
hsa-miR-422a	9.63E-03	-2.624	High (predicted)	CD226	1.47E-03	6.472
hsa-miR-455-3p	1.49E-02	-3.286	High (predicted)	FAM105A	2.11E-03	5.505
hsa-miR-744-5p	3.05E-02	-2.097	High (predicted)	PLD4	3.94E-03	2.853

hsa-miR-25-3p	4.50E-02	-2.008	High (predicted)	IFIT2	8.54E-03	3.203

Left. experimental fold change and P value of each DEM; right. experimental fold change and P value of each DEG
