# Supplementary material for: miRNAs may play a major role in the control of gene expression in key pathobiological processes in Chagas disease cardiomyopathy
Source: PLoS Negl Trop Dis. 2020 Dec 22;14(12):e0008889. doi: 10.1371/journal.pntd.0008889 (PMC7787679; doi:10.1371/journal.pntd.0008889)
Supplement: S11 Table — (PDF) [file pntd.0008889.s011.pdf]

S11 table. Five DEMs that control a large number of DEGS and processes in CCC myocardium.

DEM ID	# DEG TARGETS	# PROCESS	DEG TARGETS	PROCESSES
hsa-miR-125b-5p	23	6	ATP10D BIN2 GMIP GPR160 KLHL6 NPL PTPN18 RHEBL1 SLA2 C1orf38 TIFAB ZNF624 ZSWIM5 BMF CD69 PRDM1 PSTPIP2 SEMA4D GCNT1 IRF4 CDKN2A CCR5 TNFAIP3	Inflammation, Th1 response, fibrosis, extracellular matrix, hypertrophy, mitochondria,
hsa-miR-15a-5p	23	5	FCRL2 GPR171 HLA-DQB2 HLA-F ISLR LAMP3 C2orf43 LY9 MTMR11 MYB RAB30 SYNRG TFCP2L1 TRANK1 CNOT6L TNFSF13B WIPF1 BTLA NOTCH2 IGF1 FLT3 SLIT2 KCNN4	Inflammation, Th1 response, fibrosis, extracellular matrix, hypertrophy,
hsa-miR-296-5p	22	6	C10orf105 CLDN3 DERL3 ESPNL GDF10 KRTCAP3 MARVELD1 NFAM1 NUAK2 RAB37 SNX20 TBC1D10C BMF CCL28 CD300LF CD6 CD8A TNFSF13 CXCL10 E2F2 KCNN4 TRPM2	Inflammation, Th1 response, fibrosis, hypertrophy, oxidative stress, contraction and contractility of heart muscle
hsa-miR-29c-3p	21	8	ARRDC3 C1orf96 COL5A2 N4BP2L1 NAP1L3 NPAS3 RAB30 TMEM236 ZBTB37 COL5A1 NAV1 BMF COL4A4 TNFRSF9	Inflammation, Th1 response, fibrosis, extracellular matrix, hypertrophy,

			ICOS RGS1 COL1A2 TNFAIP3 PIK3R1 CASP8	oxydative stress, mitochondria, Arrythmia
hsa-miR-103a-3p	20	6	DOK3 C20orf103 NPL RAB30 C5orf25 SLAMF6 TIFAB TRIM14 TRIM66 ZNF226 ZNF831 AIM2 CD84 CXCL16 SAMD9L TNFSF13 BTLA CTSK CD40LG CXCL9	Inflammation, Th1 response, fibrosis, hypertrophy, mitochondria, Arrythmia
