# Supplementary figures and images for: miRNAs may play a major role in the control of gene expression in key pathobiological processes in Chagas disease cardiomyopathy
Source: PLoS Negl Trop Dis. 2020 Dec 22;14(12):e0008889. doi: 10.1371/journal.pntd.0008889 (PMC7787679; doi:10.1371/journal.pntd.0008889)

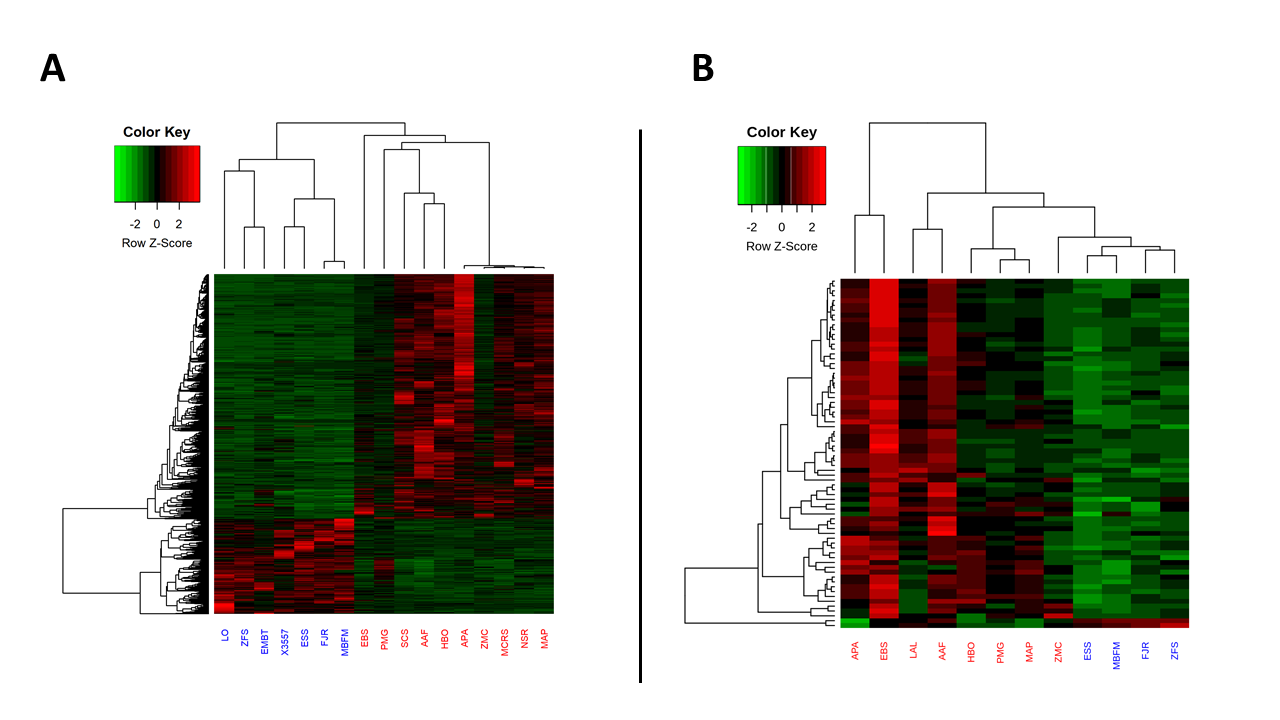

Supplement: S1 Fig — A. Unsupervised hierarchical clustering based on the 1535 DEGs. B. Unsupervised hierarchical clustering based on the 80 DEMs. (TIF) [file pntd.0008889.s012.tif]

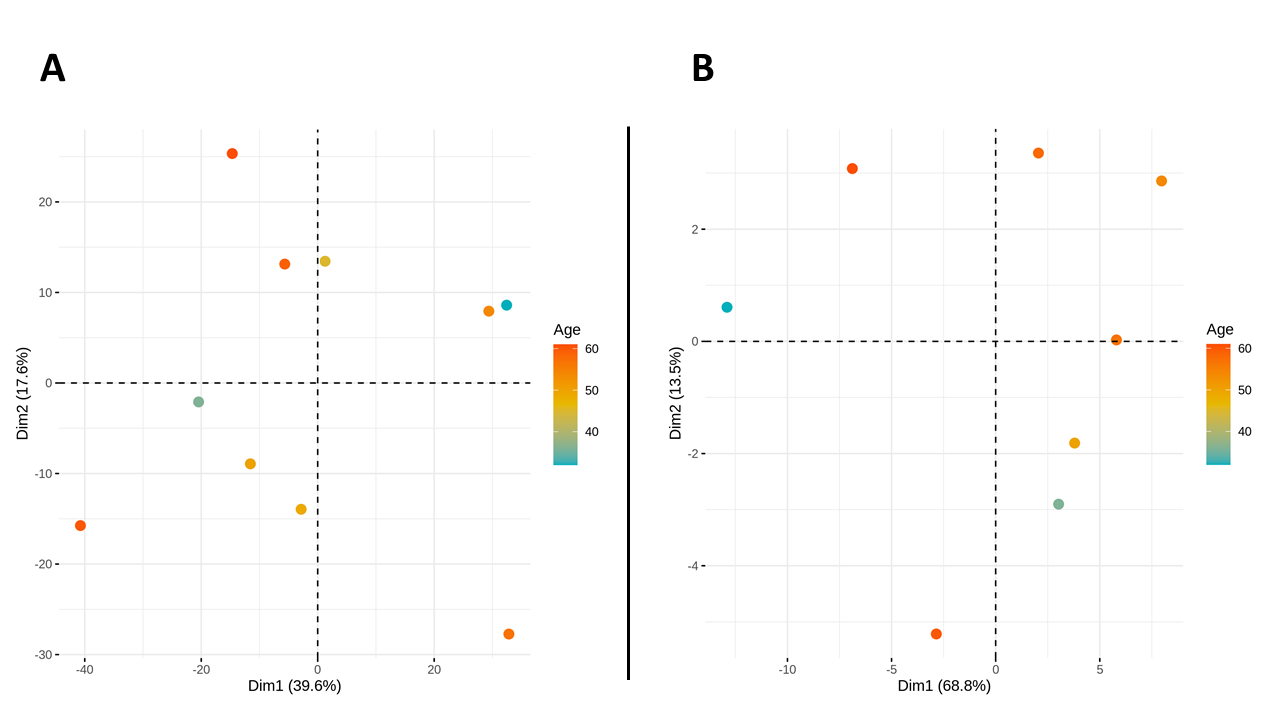

Supplement: S2 Fig — Principal component analysis (PCA) plot of samples was performed based A. on 1535 differentially expressed genes (DEGs) between CCC and controls. B. on 80 differentially expressed miRNAs (DEMs) between CCC and controls. Each plot was generated only on cases and the age of of the patients was overlaid. (TIF) [file pntd.0008889.s013.tif]

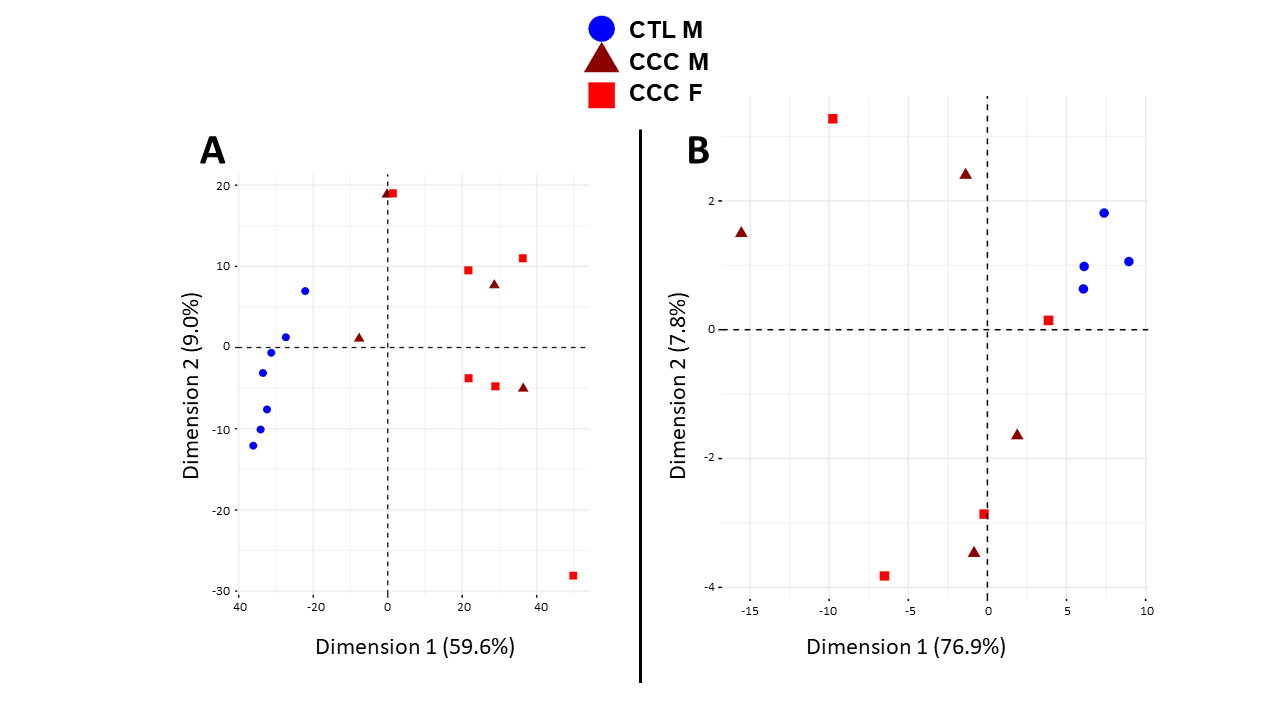

Supplement: S3 Fig — Principal component analysis (PCA) plot of samples was performed based A. on 1535 differentially expressed genes (DEGs) between CCC and controls. B. on 80 differentially expressed miRNAs (DEMs) between CCC and controls. On each plot cases and controls are indicated according to their sex. (TIF) [file pntd.0008889.s014.tif]
